# Electrosynthesis of bridged or fused sulfonamides through complex radical cascade reactions: divergence in medium-sized ring formation†

**DOI:** 10.1039/d2sc07045f

**Published:** 2023-02-21

**Authors:** Yan Zhang, Zhenzhi Cai, Chunhang Zhao, Hanliang Zheng, Lutz Ackermann

**Affiliations:** a Key Laboratory of the Ministry of Education for Advanced Catalysis Materials, Drug Discovery & Innovation Center, Department of Chemistry, Zhejiang Normal University Jinhua China zhangyan001@zjnu.edu.cn; b Institut für Organische und Biomolekulare Chemie, Georg-August-Universität Göttingen Germany Lutz.Ackermann@chemie.uni-goettingen.de

## Abstract

Radical cascade addition is one of the most important and efficient strategies for the synthesis of valuable heterocycles with structural diversity and complexity. Organic electrochemistry has emerged as an effective tool for the sustainable molecular synthesis. Herein, we describe an electrooxidative radical cascade cyclization of 1,6-enynes to access two new classes of sulfonamides, containing medium-sized rings. Differences in the activation barrier for radical addition between alkynyl and alkenyl moieties contribute to the chemo-selective addition and regioselective 7- and 9-membered ring-formation. Our finding features good substrate scope, mild conditions, and high efficiency under metal-free and chemical oxidant-free conditions. In addition, the electrochemical cascade reaction allows for the concise synthesis of sulfonamides with bridged or fused ring systems containing medium-sized heterocycles.

## Abstract 2

Radical cascade addition is one of the most important and efficient strategies for the synthesis of valuable heterocycles with structural diversity and complexity.

## Graphical abstract

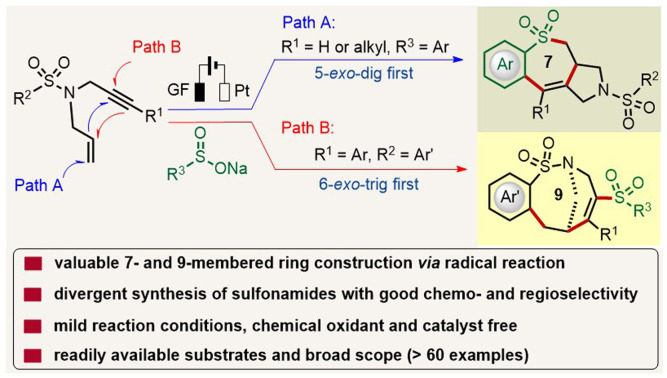

## Introduction

**Fig. 1:**
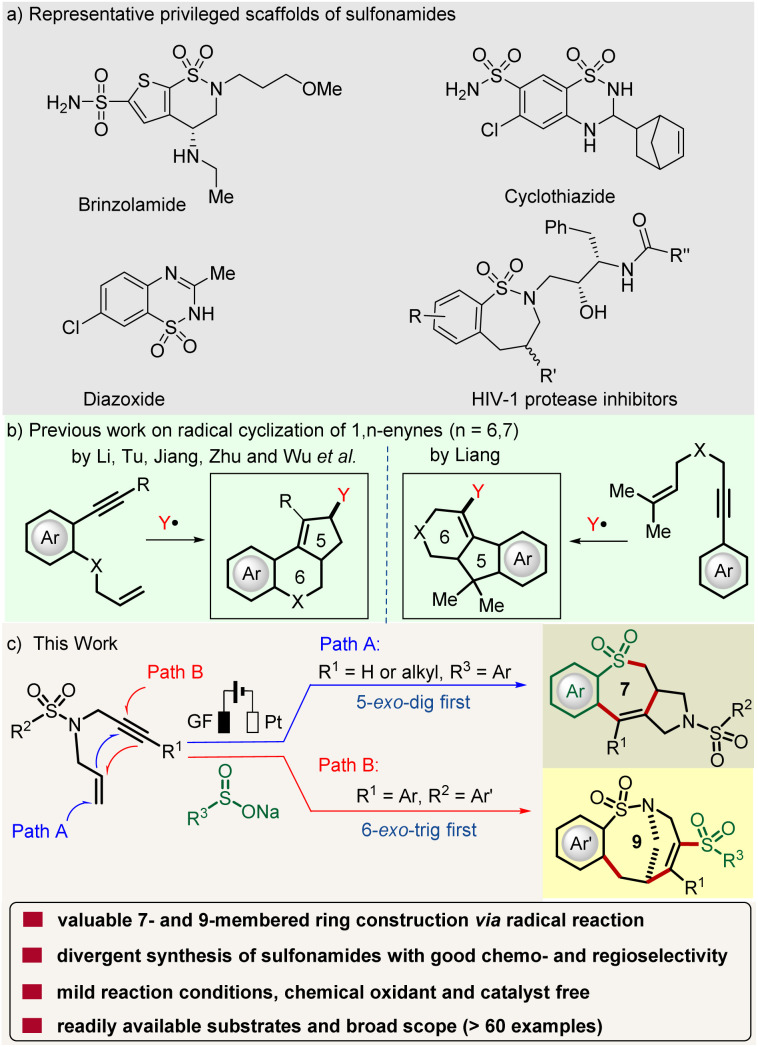
Bridged and fused sulfonamide construction: (a) selected examples of bioactive cyclic sulfonamides, (b) previous work and (c) reaction design.

Sulfonamides, especially cyclic sulfonamides are important pharmacophores and represent one of the essential structural motifs which are integral part of drugs such as brinzolamide, cyclothiazide and inhibitors of HIV-1 protease. The incorporation of such building blocks is well-known for a variety of pharmacological effects such as antibiotic, hypoglycemic, diuretic, antitumor and antihypertensive activities (Fig. 1a, top).^1,2^ Due to unfavorable transannular interaction and entropic factors, medium-sized rings (8- to 11-membered) represent a class of compounds with a synthetically challenging scaffold.^3^ Thus, in contrast to five- or six-membered rings, the synthesis of a sulfonamide containing medium-sized ring remains rare. While transition-metal-catalyzed intramolecular coupling reactions provide a common strategy, they suffer from insurmountable limitations, such as the need for a complex substrate structure and harsh reaction conditions.^4^ Notably, examples of [5 + 2] and [4 + 3] strategies have been reported by Gulías, Li and others.^5,6^ However, direct seven or medium-sized ring formation by radical cascade addition is rare, especially for sulfonamide-fused macrocyclic systems, such as the synthetically appealing nine-membered sultam core, for which no radical protocol has been reported yet. Therefore, approaches to access macrocyclic sulfonamides, and especially their divergent synthesis from simple materials under environmentally friendly conditions are in high demand.

Electrochemistry, a green and operationally simple method, has surfaced as an attractive technique used in organic synthesis.^7^ The generation of reactive radicals by electron and proton transfer taking place at the electrodes was widely exploited for versatile transformations.^8^ Specifically, electrooxidative radical cascade cyclizations proved to be efficient for constructing complex molecular scaffolds,^9^ exhibiting several distinct characteristics, such as being transition-metal-free, external chemical oxidant-free and the direct introduction of carbon/heteroatom-containing radicals.

1,6-Enynes, classical polyfunctionalized radical receptors, have attracted considerable attention and elegantly been applied in organic syntheses,^10^ which enable access to highly complex and often polycyclic ring systems through consecutive steps. However, 1,*n*-enynes (*n* = 6 or 7) are mostly limited to the construction of simple five- or six-membered cyclic scaffolds by radical reactions (Fig. 1b).^11–13^ To the best of our knowledge, there is, thus far, no reported example for medium-sized ring formation by a cascade radical reaction of 1,*n*-enynes. This is mainly due to the fact that it is challenging to control their reactivity and selectivity, in terms of chemo- and regio-selectivity, when involving various radical cyclization modes. Although DFT calculations and experimental results show that intermolecular electrophilic radical addition to alkenes is easier than addition to an alkyne,^14,15^ we believe that the chemo-selectivity in the radical addition to 1,*n*-enynes can be controlled by electronic effects on the substrates and the reaction conditions.^16^ Based on our interest in electrooxidative radical reactions,^17^ herein, we report on an unprecedented cascade cyclization of 1,6-enynes under operationally simple electrochemical conditions, which can be performed under water-tolerant radical reaction conditions (Fig. 1c). Notable features of our strategy include (a) the synthesis of two classes of 7- and 9-membered sulfonamides, (b) a traceless removal of the thus-obtained C(sp^2^)–S-motif, (c) catalyst- and chemical oxidant-free conditions, (d) controllable radical addition to 1,6-enynes, (e) mild reaction conditions, and (f) the use of readily available substrates with a broad scope.

## Results and discussion

We initiated our studies by probing various reaction conditions for the envisioned domino cyclization of *N*-allyl-*N*-(prop-2-yn-1-yl)benzenesulfonamide (2a) as an easily available model substrate (Table 1 and Table S-2 in the ESI†), using the inexpensive *p*-toluenesulfinate (1a) as the sulfonyl radical source. After considerable preliminary experimentation, we observed that the desired 6–7–5 tricyclic fused product 3aa was isolated in 71% yield with a mixed solvent system consisting of 1,1,1,3,3,3-hexafluoro-2-propanol (HFIP)/H_2_O (20 : 1) and Et_4_NClO_4_ as the electrolyte at room temperature (entry 1). Different solvents and a series of supporting electrolytes were tested, but proved not to be beneficial (entries 2–9). We found that the addition of Na_2_CO_3_ could facilitate cyclization (entry 10). Increasing the reaction temperature and the current failed to improve the yield of 3aa (entries 11 and 12). Changing the electrode material to a nickel cathode, as well as a platinum anode led to a decrease in the yield (entry 13). Control experiments confirmed the essential role of electricity in the cascade electrooxidative cyclization (entries 14 and 15).

**Table 1:** Optimization of the electrooxidative radical cyclization for the fused product synthesis^a^

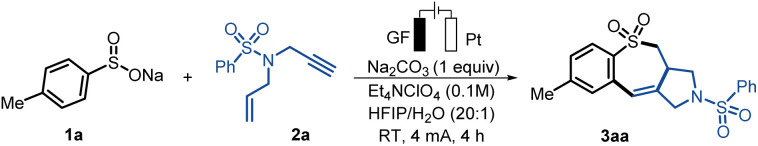		
Entry	Deviation from standard conditions	Yield/%
1	No change	71
2	MeCN	0
3	MeCN/H_2_O (3 : 1)	33
4	HFIP	38
5	HFIP/H_2_O (10 : 1)	42
6	LiClO_4_ as the electrolyte	24
7	*n*-Bu_4_NBF_4_ as the electrolyte	28
8	*n*-Bu_4_NClO_4_ as the electrolyte	20
9	Et_4_NClO_4_ (0.05 M)	41
10	Without Na_2_CO_3_	50
11	Reaction at 50 °C	60
12	CCE = 6 mA	54
13	GF(+)|Ni(−) instead of GF(+)|Pt(−)	50
14	No electricity	0
15^b^	No electricity, Mn(OAc)_3_ as the oxidant	16

a Standard conditions: undivided cell, GF anode, Pt cathode, constant current = 4 mA, 1a (0.60 mmol, 2.0 equiv.), 2a (0.30 mmol, 1.0 equiv.), electrolyte (0.1 M), solvent (4 mL), in air, 4 h, 2.0 F mol^−1^. Yield of the isolated product.

b HFIP as the solvent.

**Scheme 1:**
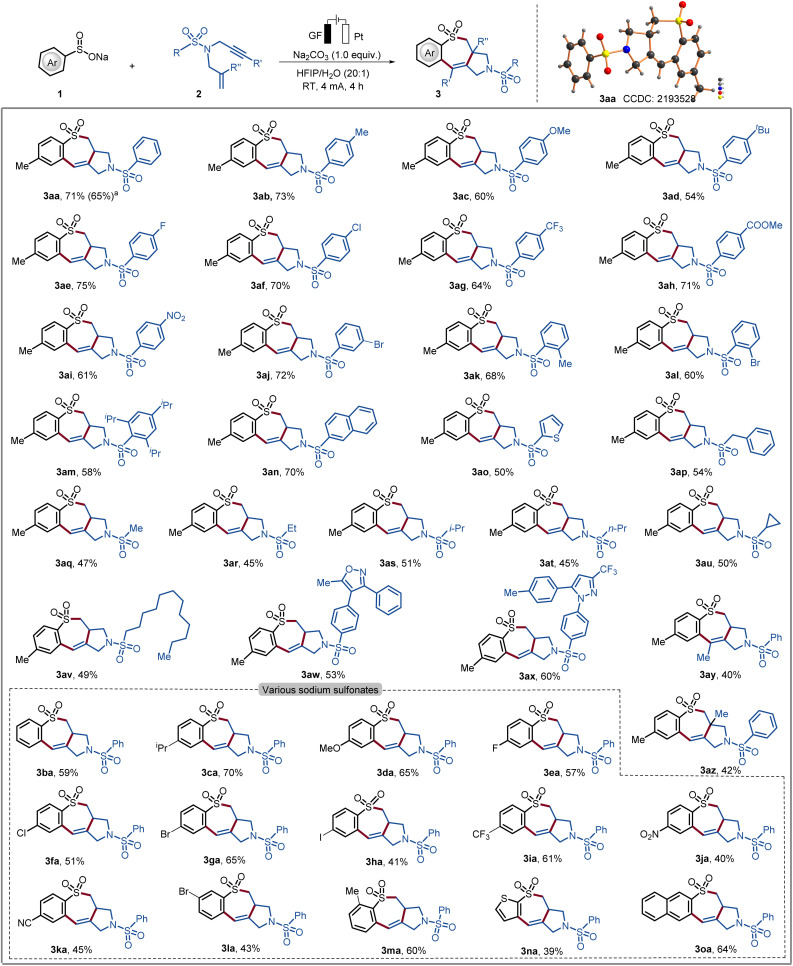
Scope of electrooxidative 7-*endo*-trig cyclization. Reaction conditions from Table 1, entry 1, sodium sulfinate (1, 0.60 mmol), 1,6-enynes (2, 0.30 mmol) and Na_2_CO_3_ (0.30 mmol) in HFIP/H_2_O = 20 : 1 (v/v, 4.0 mL), 4 mA, 4 h. Isolated yield. ^a^1 mmol scale.

With the optimized reaction conditions in hand, we explored the scope of the cascade electrochemical radical cyclization of sodium sulfinate 1 and 1,6-enynes 2 to access seven-membered heterocycles 3 (Scheme 1). We first examined the transformation of a diverse range of 1,6-enynes 2 with different substitution patterns. The reaction tolerated a variety of substituents with diverse electronic properties in all positions of the phenyl (3aa–3am). Substrates 2 decorated with both electron-withdrawing and electron-donating groups on the aryl ring did not have a significant effect on the yield of the reaction. In addition, heterocyclic substrates bearing thiophene as well as naphthyl as a substituent were also tolerated in the electrooxidative cyclization (3an and 3ao). A heterocyclic substrate proved to also be applicable in the electrooxidative transformation to selectively afford the corresponding product 3ao in good yield. Having demonstrated the broad applicability with arene sulfonamides, substrates derived from alkyl sulfonamides were tested. The corresponding products 3aq–3av were synthesized in comparatively low yields, indicating that the steric hindrance and/or electronic effect of the amide part plays an important role in the reaction. Furthermore, celecoxib and vadecoxib derivatives were also applicable in the radical cascade reaction and formed the corresponding products 3ax and 3aw in 60% and 53% yield, respectively. It is noteworthy that, internal alkynes and alkenes were tolerated and furnished the 7-membered cyclic sulfonamides 3ay and 3az. The reaction of 1a with 2a was scaled up to 1.0 mmol for practical application, generating 3aa in 65% yield. Moreover, the structure of product 3aa was further confirmed by single-crystal X-ray analysis (Scheme 1).^18*a*^

To further examine the efficacy of the present electrochemical domino cyclization scope in terms of various sodium sulfinates, 1 with 1,6-enynes 2a was (3ba–3oa) was probed. To our delight, both common electron-donating substituents and electron-withdrawing functional groups, namely chloro, bromo, trifluoromethyl, nitro and cyano groups, were well tolerated in radical cyclization and formed the desired products. In addition, naphthalene sulfonate and thiophene sulfonate also reacted well with substrate 2a to form the annulated products 3na and 3oa in good yields. However, we sought to examine the reactivity for various substituted ethenes and ethynes, in terms of diverse electronic properties (see Table S-4†). We assumed that the order of radical addition to 1,6-enynes can be shifted, enabling the synthesis of medium-sized ring-containing sulfonamides.

**Scheme 2:**
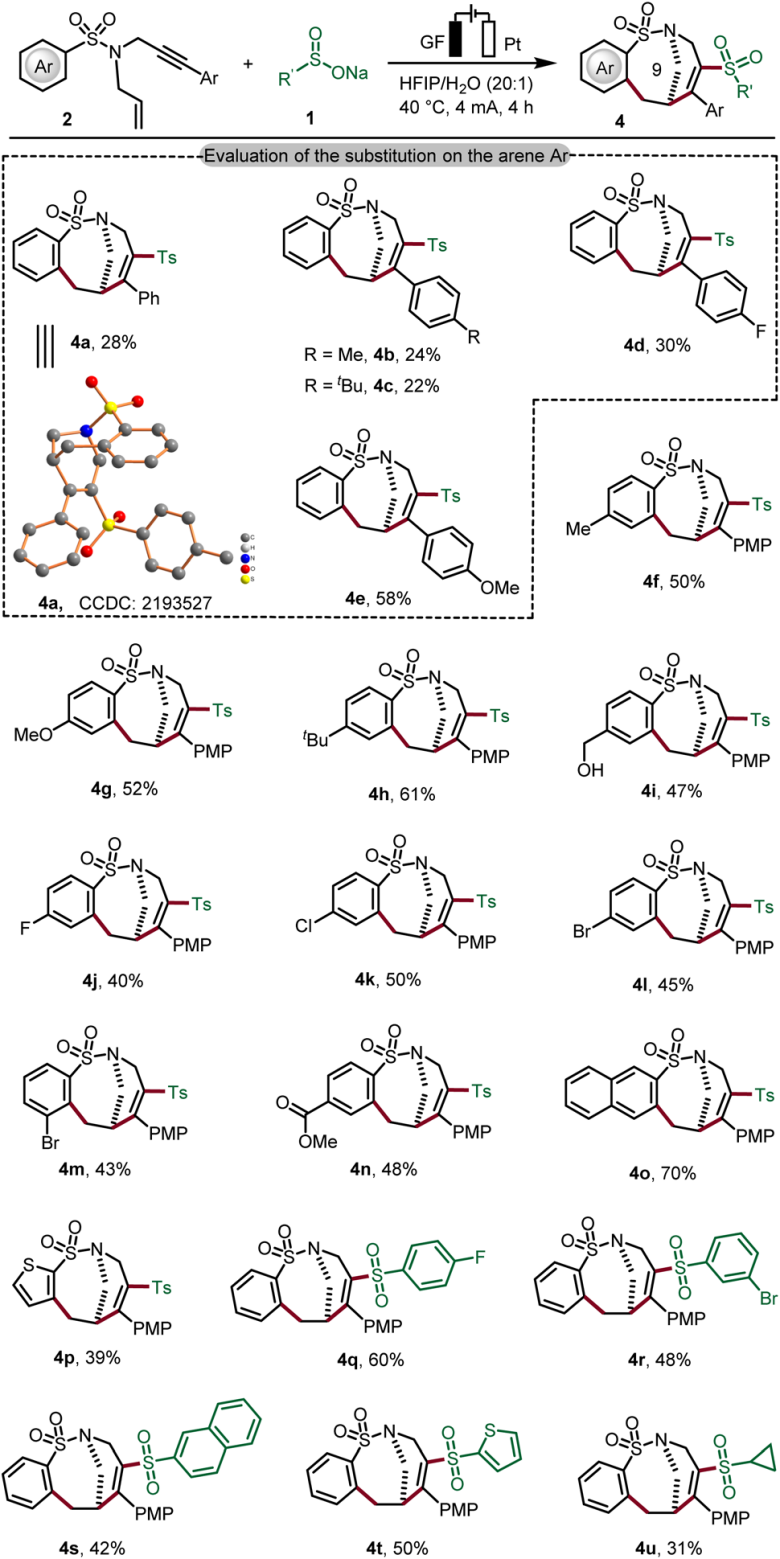
Scope for preparing the bridged ring products containing a 9-member-ring. Reaction conditions from Table 1, entry 1, sodium sulfinate (1, 0.60 mmol), 1,6-enynes (2, 0.30 mmol) in HFIP/H_2_O = 20 : 1 (v/v, 4.0 mL), 4 mA, 4 h. Isolated yield. (PMP = *para*-methoxyphenyl).

Although electrophilic free radical addition to alkenes is typically more facile than addition to alkynes, our experiments highlight that the chemo-selectivity in the radical addition to 1,*n*-enynes could be controlled by electronic effects on the substrates. We then examined the generality of this new type of cascade electrooxidative transformation for preparing bridged cyclic products containing 9-membered cyclic sulfonamides (Scheme 2). As expected, we observed a substitution effect of the arene Ar under the optimized electrooxidative conditions (4a–4e). We were pleased to find that 4-(methoxyphenyl) enyne 2e' gave the best result and the desired product 4e was thereby obtained in 58% yield (Table S-3 in the ESI†). We believe that the *para*-methoxyphenyl (PMP) group can thus stabilize the thus-formed vinyl radical upon Ts˙ addition. To our delight, common electron-donating substituents and electron-withdrawing functional groups were well tolerated in cyclization and the desired products 4f–4l were formed efficiently. In addition, naphthalene sulfinate and thiophene sulfinate also reacted well with substrate 1a to assemble tricyclic products 4o and 4p in good yields. Likewise, the mild electrooxidative radical cyclization approach was found to be generally applicable to various sodium sulfinates, furnishing the expected 9-membered bridged cycles (4q–4u). The structure of bridged sulfonamide 4a was unambiguously verified by X-ray crystallographic analysis.^18*b*^

**Scheme 3:**
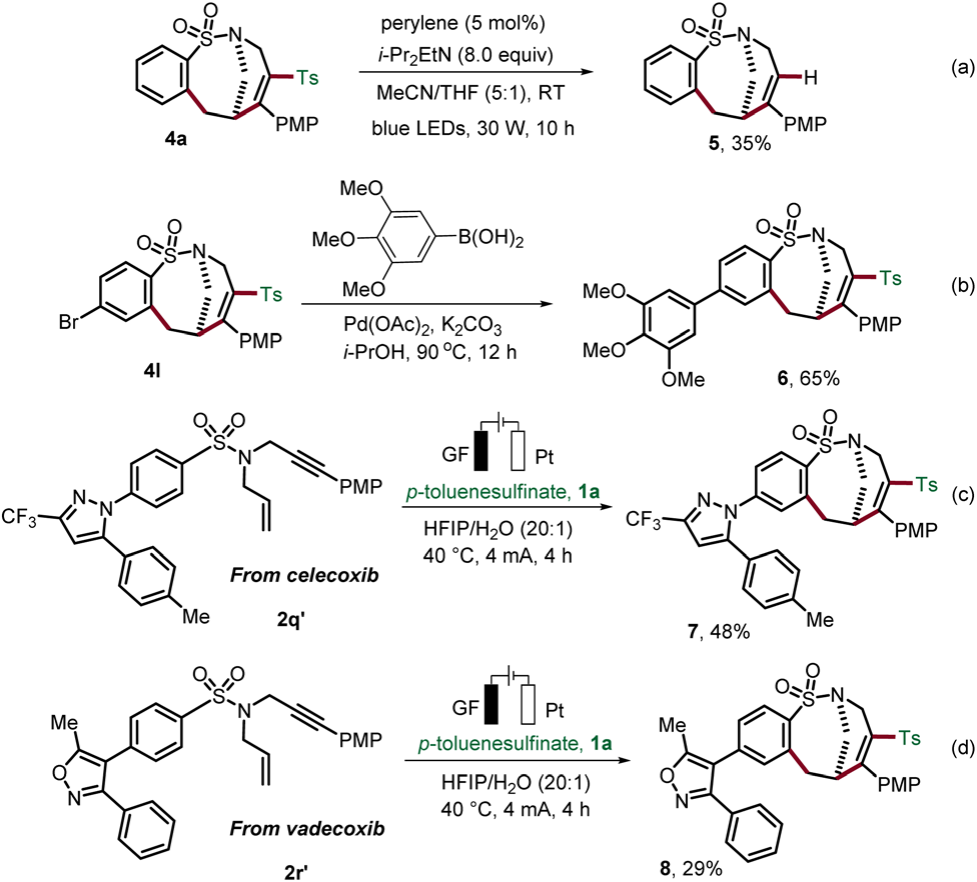
Derivatization of compound 4 and synthetic application.

To illustrate the synthetic utility of the obtained sulfonamides 4, we investigated their further diversification (Scheme 3). Continuing our concept of the removable radical motif strategy,^19^ we removed the incorporated sulfonyl motif on the product. Photochemical desulfonylation delivered sulfonamide 5 (Scheme 3a). In addition, trimethoxyphenyl, a common motif in many biologically active compounds, was successfully attached to the medium-sized sulfonamide 4l by Suzuki cross-coupling in i-PrOH (Scheme 3b).^20^ Our strategy for the construction of bridged rings was further expanded by applying it to complex substrates derived from the anti-inflammatory drugs celecoxib and vadecoxib, thus, providing the medium-sized products 7 and 8 (Scheme 3c and d).

**Fig. 2:**
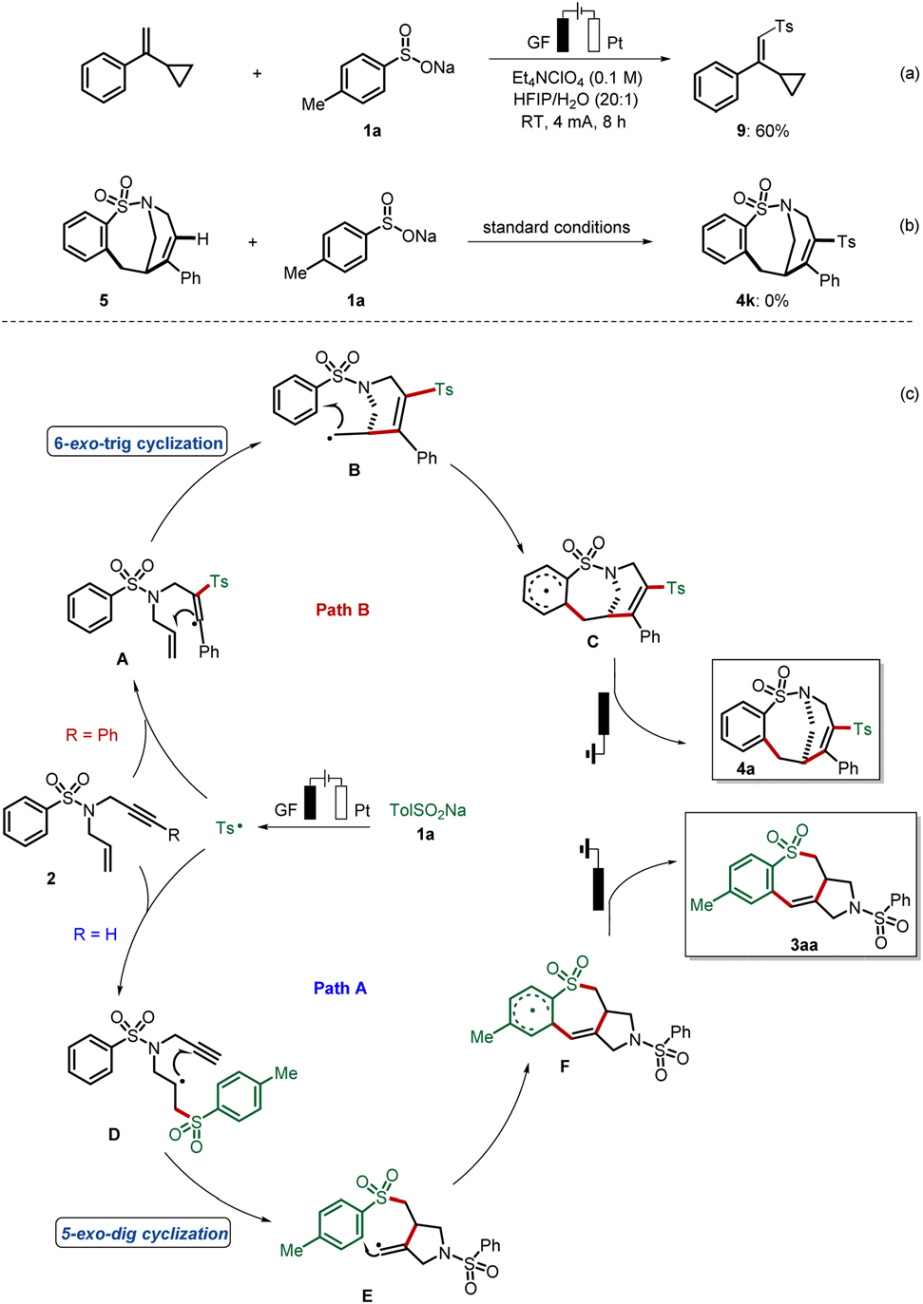
Mechanistic investigation on radical annulation: the (a) radical trapping experiment, (b) control experiment, and (c) proposed mechanism.

Control experiments were conducted to verify the radical nature of this cascade electrosynthesis. The Ts˙ that was proven to be easily generated as shown by our previous cyclic voltammetry (CV) experiment,^19^ could be trapped by (1-cyclopropylvinyl)benzene to form the adduct 9 in 60% yield (Fig. 2a). This radical trapping experiment shows that the addition of the Ts˙ radical onto the alkenyl part occurred, and that the formed benzyl radical is easily oxidized without cyclopropyl ring opening, a notable difference to our previous report.^19^ What is more, the addition of Ts˙ is not possible after formation of the medium-sized ring, which has been revealed in our second control experiment (Fig. 2b). Overall, these experimental results strongly supported the occurrence of a radical cyclization cascade leading to the formation of a tricyclic scaffold and bridged cyclic sulfonamides, which is illustrated in the proposed mechanistic pathway in Fig. 2c. First, the Ts is generated from *p*-toluenesulfinate 1a through anodic oxidation. Selective radical addition of Ts˙ to the C–C triple bond of 1,6-enynes 1 affords a vinyl radical A, which undergoes 6-*exo*-trig cyclization to form intermediate B. Then the key intramolecular cyclization of B results in the formation of radical intermediate C. Finally, C undergoes further single-electron-transfer (SET) oxidation and deprotonation to form the bridged ring product 4a. Alternatively, with respect to the formation of the 6–7–5 fused-system, the Ts radical chemo-selectively reacts with the C–C double bond of 2 affording an alkyl radical D. Then, D undergoes 5-*exo*-dig cyclization to form a vinyl radical E, which subsequently reacts with the radical intermediate F through radical cyclization. Radical F next undergoes another SET oxidation, followed by deprotonation, to afford the tricyclic product 3aa.

## Conclusion

In summary, we have presented an electrooxidative method for selective radical cascade addition to 1,6-enynes giving access to 7- and 9-membered sulfonamide containing rings. This method relies on the use of easily available sulfonamides. Key to the desired transformations is the chemo- and regioselective radical addition to the C–C double or triple bond controlled by the different activation barriers, depending on the alkyne substitution. It is worth mentioning that the generated sulfonyl radical which is used to construct medium-sized heterocycles can be removed in the presence of a photocatalyst. The developed electrooxidative strategy represents a rare cascade radical cyclization containing a macrocyclic system and is a useful method for the concise assembly of a variety of bridged and fused sulfur-containing rings with structural novelty and complexity.

## Data availability

The datasets supporting this article have been uploaded as part of the ESI material.†

## Author contributions

Y. Z. and L. A. conceptualized the project. Z. C., C. Z. and H. Z. performed the experimental studies. Y. Z. and L. A. prepared the manuscript.

## Conflicts of interest

There are no conflicts to declare.

## Supplementary Material

SC-014-D2SC07045F-s001

SC-014-D2SC07045F-s002
