# Supplementary material for: Electrosynthesis of bridged or fused sulfonamides through complex radical cascade reactions: divergence in medium-sized ring formation
Source: Chem Sci. 2023 Feb 21;14(13):3541–7. doi: 10.1039/d2sc07045f (PMC10055698; doi:10.1039/d2sc07045f)
Supplement: SC-014-D2SC07045F-s001 [file SC-014-D2SC07045F-s001.pdf]

Electronic Supplementary Material (ESI) for Chemical Science.
This journal is © The Royal Society of Chemistry 2023

Supporting Information for

Electrosynthesis of Bridged or Fused Sulfonamides through Complex Radical Cascade Reactions: Divergence in Medium-Sized Ring Formation

Yan Zhang,[a] Zhenzhi Cai,[a] Chunhang Zhao,[a] Hanliang Zheng,[a] Lutz Ackermann*[b]*

[a] Key Laboratory of the Ministry of Education for Advanced Catalysis Materials, and Drug Discovery & Innovation Center, Department of Chemistry, Zhejiang Normal University, 688 Yingbin Road, 321004 Jinhua P. R. China
*zhangyan001@zjnu.edu.cn

[b] Institut für Organische und Biomolekulare Chemie, Georg–August–Universität Göttingen
Tammannstraße 2, 37077 Göttingen, Germany
*Lutz.Ackermann@chemie.uni–goettingen.de

Table of Contents

1. General Remarks S2
2. Scope S3
3. Optimization S4
4. General Procedures S6
5. Characterization Data S8
6. Derivatization of the products **4**..S40
5. Mechanistic Studies S41
6. Density Functional Theory (DFT) Computations S42
7. NMR Spectra S52

1. General Remarks

NMR spectra were recorded on BRUKER AVANCE III 400 or BRUKER AVANCE III 600. $CDCl_3$ was used as the solvent. Chemical shifts were referenced relative to residual solvent signal ($CDCl_3$: ^{1}H NMR: δ 7.26 ppm, ^{13}C NMR: δ 77.16 ppm). The following abbreviations are used to describe peak patterns where appropriate: br = broad, s = singlet, d = doublet, t = triplet, q = quartet, m = multiplet. Coupling constants (*J*) are reported in Hertz (Hz). Electrospray-ionization (ESI) mass spectra were obtained on AB Sciex LC 30A-Triple TOF 4600 apparatus. All systems are equipped with time-of-flight (TOF) analyzers. Melting points were measured with micro melting point apparatus. Platinum electrodes (10 mm × 25 mm × 0.25 mm, 99.9%; obtained from ChemPur® Karlsruhe, Germany) and graphite felt (GF) electrodes (10 mm × 25 mm × 6 mm, SIGRACELL® GFA 6 EA, obtained from SGL Carbon, Wiesbaden, Germany) were connected using stainless steel adapters. Unless otherwise noted, some materials (or alternatively chemicals) obtained from commercial suppliers were used directly without further purification. 1,6-enynes **1** were prepared according to the literatures.[1,2]

2. Scope

Table S–1. Scope of 1,6-Enynes 2 and Sodium Sulfinate 1.

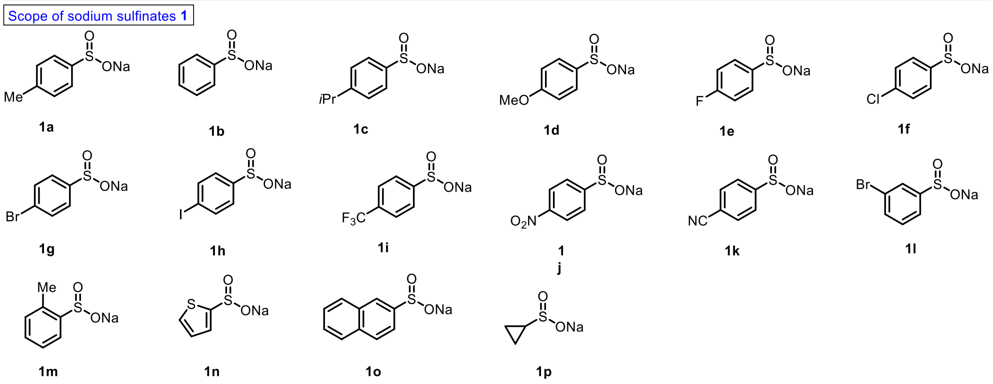

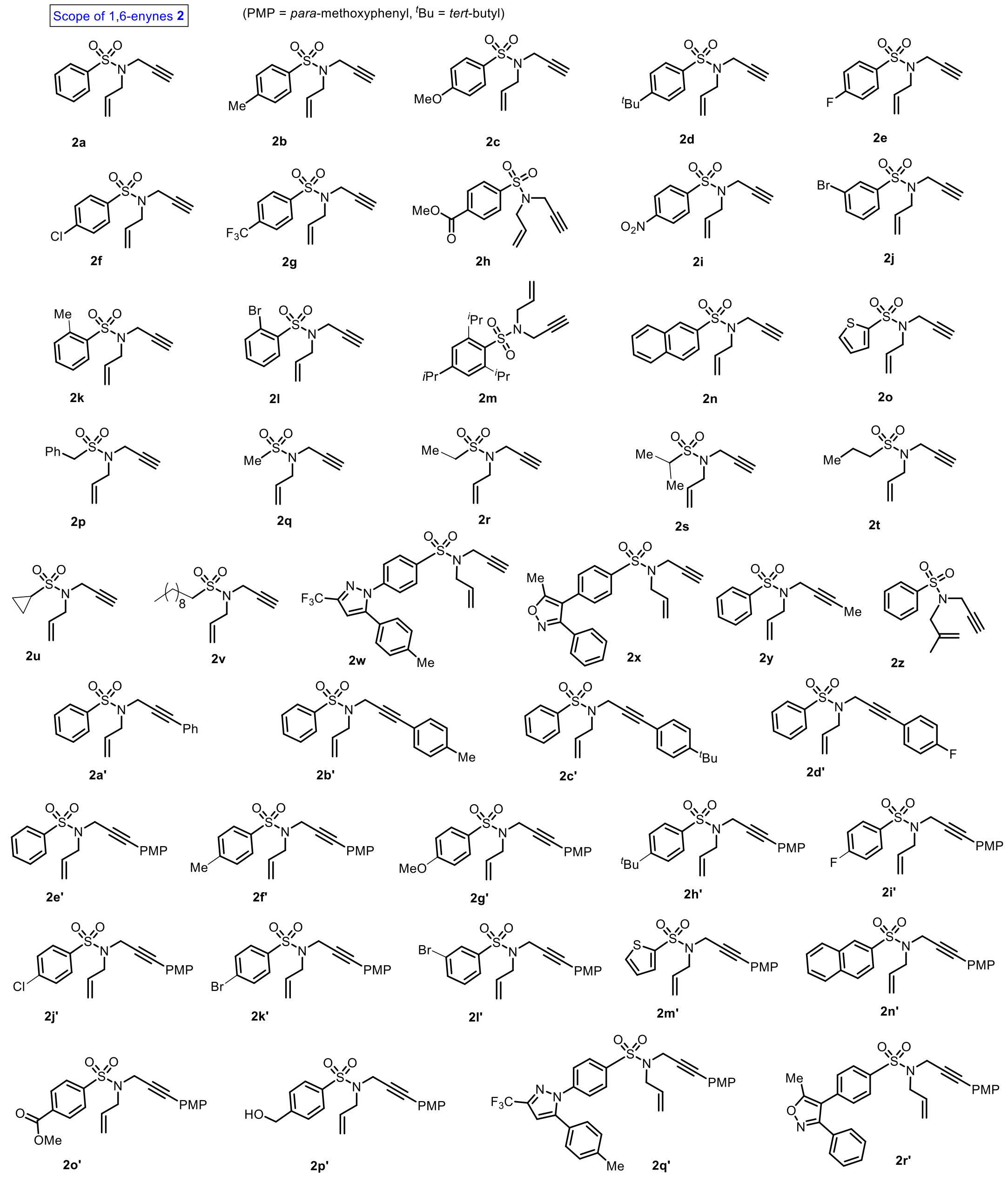

3. Optimization

Table S–2. Optimization of the electrooxidative radical cyclization for the synthesis of the fused sulfonamide **3aa**.

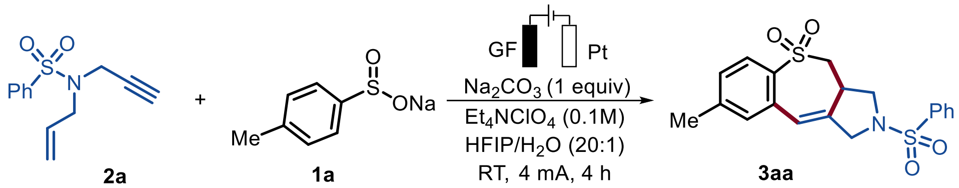

Entry	Solvent	Electrolyte	T / °C	additive	Yield/%
1	MeCN	Et_4NClO_4	rt	Na_2CO_3	0
2	HFIP	Et_4NClO_4	rt	Na_2CO_3	38
3	MeCN/H_2O (3:1)	Et_4NClO_4	rt	-	13
4	MeCN/H_2O (3:1)	Et_4NClO_4	rt	Na_2CO_3	33
5	MeCN/HFIP (3:1:)	Et_4NClO_4	rt	Na_2CO_3	60
6	MeCN/HFIP/H_2O (3:1:0.2)	Et_4NClO_4	rt	Na_2CO_3	69
7	HFIP/H_2O (20:1)	Et_4NClO_4	rt	Na_2CO_3	71
8	HFIP/H_2O (10:1)	Et_4NClO_4	rt	Na_2CO_3	42
9	HFIP/H_2O (20:1)	Et_4NClO_4	rt	-	46
10	HFIP/H_2O (20:1)	Et_4NClO_4	rt	K_2CO_3	28
11	HFIP/H_2O (20:1)	Et_4NClO_4	rt	Na_2HPO_4	30
12	HFIP/H_2O (20:1)	Et_4NClO_4	40	Na_2CO_3	63
13	HFIP/H_2O (20:1)	Et_4NClO_4	60	Na_2CO_3	23
14	HFIP/H_2O (20:1)	LiOTf	rt	Na_2CO_3	23
15	HFIP/H_2O (20:1)	$LiClO_4$	rt	Na_2CO_3	24
16	HFIP/H_2O (20:1)	*n*-Bu_4NBF_4	rt	Na_2CO_3	28
17	HFIP/H_2O (20:1)	*n*-Bu_4NBF_6	rt	Na_2CO_3	21
18	HFIP/H_2O (20:1)	*n*-Bu_4NClO_4	rt	Na_2CO_3	20
19[a]	HFIP/H_2O (20:1)	Et_4NClO_4	rt	Na_2CO_3	41
20[b]	HFIP/H_2O (20:1)	Et_4NClO_4	rt	Na_2CO_3	22
21[c]	HFIP/H_2O (20:1)	Et_4NClO_4	rt	Na_2CO_3	0

Standard conditions: Undivided cell, GF anode, Pt cathode, constant current = 4 mA, **2a** (0.30 mmol), **1a** (0.60 mmol, 2.0 equiv), eletrolyte (0.1 M), solvent (4 mL), additive (1.0 equiv), under 30 °C, 4 h, 2.0 $Fmol^{-1}$. Yield of the isolated product. [a] eletrolyte (0.05 M). [b] GF(+)|Ni(−) instead of GF(+)|Pt(−). [c] Pt(+)|Pt(−) instead of GF(+)|Pt(−).

Table S–3. Optimization of electrooxidative synthesis of the product containing 9-membered ring **4e**.[a]

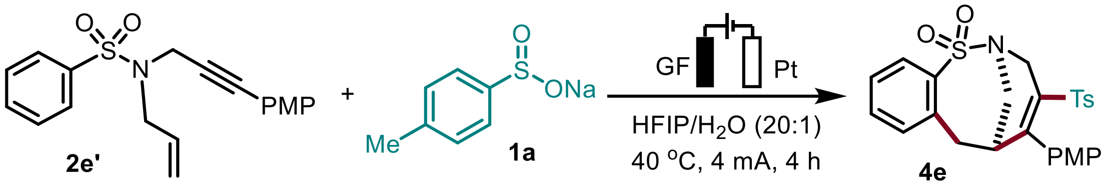

Entry	Solvent	Electrolyte	T /℃	additive	Yield/%
1	MeCN	Et_4NClO_4	rt	Na_2CO_3	trace
2	HFIP	Et_4NClO_4	rt	Na_2CO_3	20
3	MeCN/H_2O (10:1)	Et_4NClO_4	rt	Na_2CO_3	26
4	HFIP/H_2O (20:1)	Et_4NClO_4	rt	Na_2CO_3	51
5	MeCN/HFIP/H_2O (3:1:10.0 eq.)	Et_4NClO_4	rt	Na_2CO_3	24
6	HFIP/H_2O (20:1)	Et_4NClO_4	40	Na_2CO_3	43
7	HFIP/H_2O (20:1)	Et_4NClO_4	60	Na_2CO_3	34
8	HFIP/H_2O (20:1)	Et_4NClO_4	40	-	58
9	HFIP/H_2O (20:1)	Et_4NClO_4	40	K_2CO_3	28
10	HFIP/H_2O (20:1)	Et_4NClO_4	40	Na_2HPO_4	20
11	HFIP/H_2O (20:1)	*n*-Bu_4NBF_4	40	-	25
12	HFIP/H_2O (20:1)	*n*-Bu_4NBF_6	40	-	21
13	HFIP/H_2O (20:1)	*n*-Bu_4NClO_4	40	-	26
14	HFIP/H_2O (20:1)	-	40	-	20
15[b]	HFIP/H_2O (20:1)	Et_4NClO_4	40	-	14

[a] Standard conditions: Undivided cell, GF anode, Pt cathode, constant current = 4 mA, **2a** (0.30 mmol), **1a** (0.60 mmol, 2.0 equiv), eletrolyte (0.1 M), solvent (4 mL), under 40 °C, 4 h. Yield of the isolated product. [b] GF(+)|Ni(−) instead of GF(+)|Pt(−).

4. General Procedures

(A) General Procedure for Electrooxidative Radical Cyclization: Access to Fused Cyclic Sulfonamides.

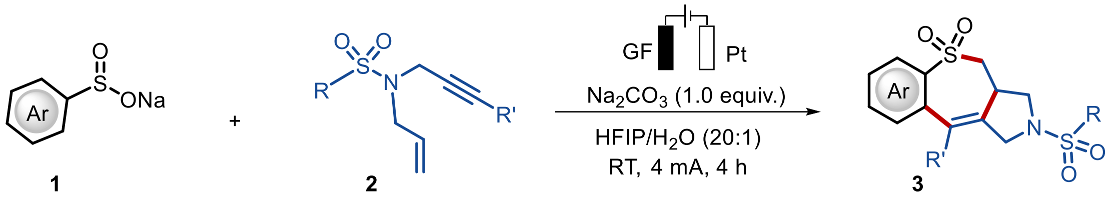

The electrocatalysis was carried out in an undivided cell under air with a graphite felt (GF) anode (10 mm × 15 mm × 6 mm) and a platinum cathode (10 mm × 15 mm × 0.25 mm). Sodium sulfonates $ArSO_2Na$ **1** (0.6 mmol, 2.0 equiv), 1,6-enynes **2** (0.3 mmol, 1.0 equiv), Na_2CO_3 (0.3 mmol, 1.0 equiv) and Et_4NClO_4 (92 mg, 0.1 M) were dissolved in a mixture of HFIP and H_2O (20:1, 4 mL). Electrocatalysis was performed at room temperature with a constant current of 4.0 mA maintained for 4 h. The GF anode was washed with ethyl acetate (3 × 3 mL) in an ultrasonic bath and transfered to the round bottom flask with the crude reaction solution. Silica was added to the flask and all volatiles were evaporated under vacuum. Purification was performed by flash column chromatography on silica gel using petroleum ether/ EtOAc as the eluent to give the corresponding products **3**.

(B) General Procedure for Electrooxidative Radical Cyclization: Access to Bridged Sulfonamides.

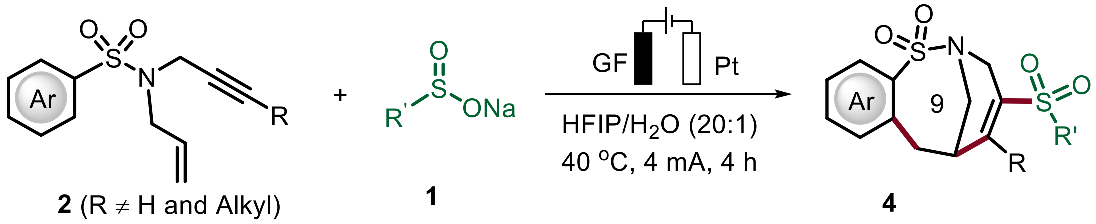

The electrocatalysis was carried out in an undivided cell under air with a graphite felt (GF) anode (10 mm × 15 mm × 6 mm) and a platinum cathode (10 mm × 15 mm × 0.25 mm). Sodium sulfonates $ArSO_2Na$ **1** (0.6 mmol, 2.0 equiv), 1,6-enynes **2** (0.3 mmol, 1.0 equiv) and Et_4NClO_4 (92 mg, 0.1 M) were dissolved in a mixture of HFIP and H_2O (20:1, 4 mL). Electrocatalysis was performed at room temperature with a constant current of 4.0 mA maintained for 4 h. The GF anode was washed with ethyl acetate (3 × 3 mL) in an ultrasonic bath and transfered to the round bottom flask with the crude reaction solution. Silica was added to the flask and all volatiles were evaporated under

vacuum. Purification was performed by flash column chromatography on silica gel using petroleum ether/ EtOAc as the eluent to give the corresponding products **4**.

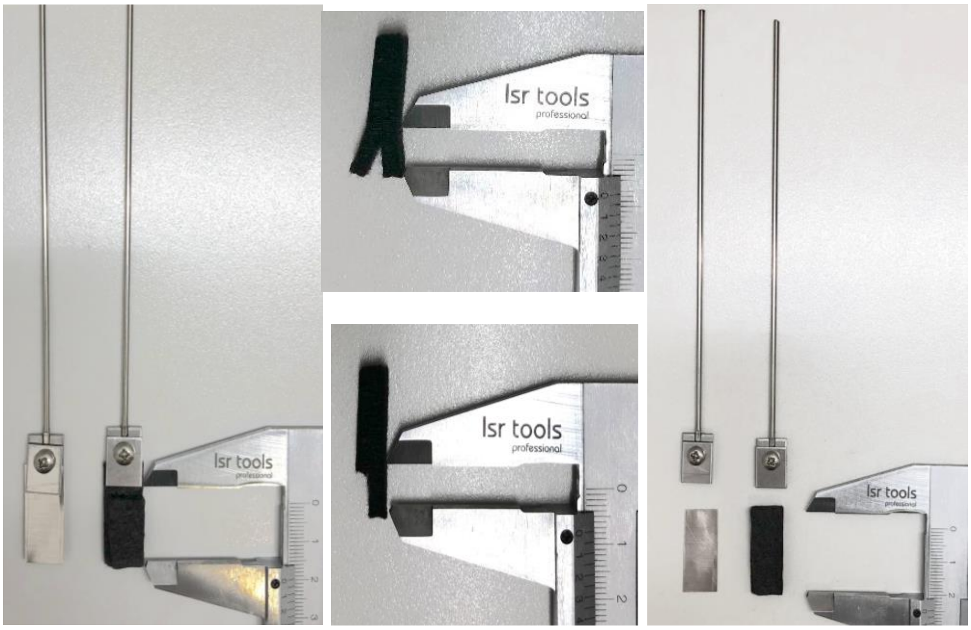

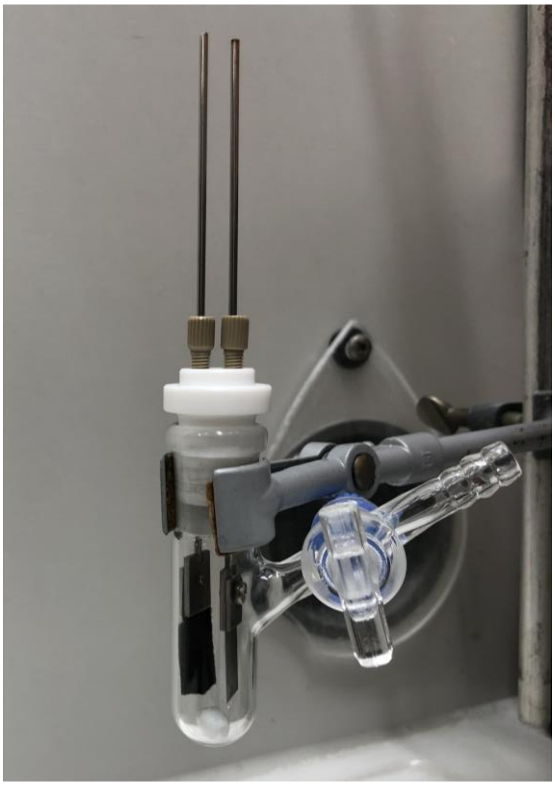

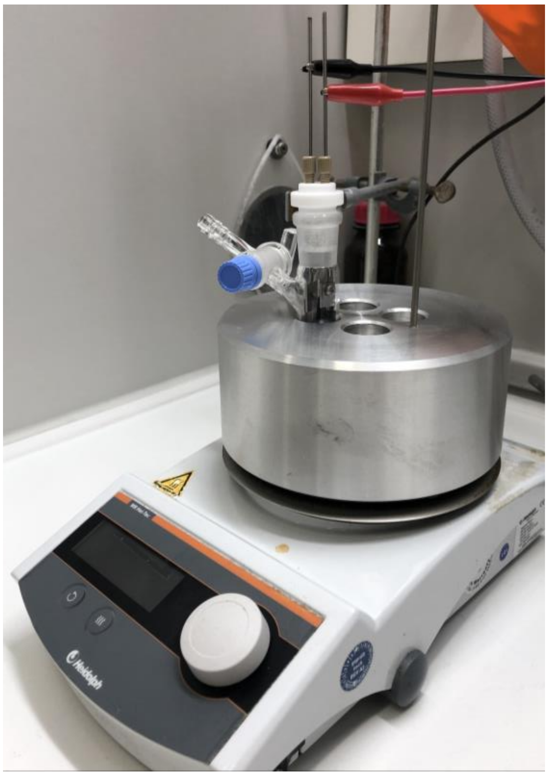

5. Characterization Data

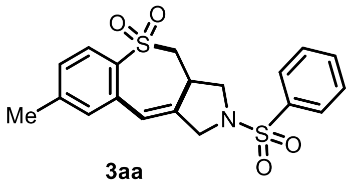

3aa

8-Methyl-2-(phenylsulfonyl)-2,3,3a,4-tetrahydro-1*H*-benzo[6,7]thiepino[3,4-*c*]pyrrole 5,5-dioxide (3aa)

The general procedure (A) was followed using **1a** (107 mg, 0.60 mmol), **2a** (70.5 mg, 0.3 mmol) and Na_2CO_3 (31.8 mg, 0.30 mmol). Purification by column chromatography on silica gel (petroleum ether/ EtOAc = 3:1) yielded **3aa** (82.8 mg, 71%) as a white solid **M.p.**: 184–186 °C. **^{1}H NMR** (400 MHz, $CDCl_3$) δ 8.00 (d, J = 8.1 Hz, 1H), 7.88–7.86 (m, 2H), 7.68–7.64 (m, 1H), 7.61– (d, J = 14.8 Hz, 2H), 7.22 (d, J = 8.1 Hz, 1H), 7.10 (s, 1H), 6.44 (d, J = 1.9 Hz, 1H), 4.23 (dt, J = 14.4, 1.6 Hz, 1H), 4.07 (dt, J = 14.5, 1.6 Hz, 1H), 3.75 (dd, J = 9.4, 7.5 Hz, 1H), 3.55 (dd, J = 13.2, 4.2 Hz, 1H), 3.45–3.31 (m, 2H), 3.10 (t, J = 9.1 Hz, 1H), 2.40 (s, 3H). **^{13}C NMR** (151 MHz, $CDCl_3$) δ 144.6, 142.0, 136.6, 135.8, 133.5, 133.5, 132.4, 129.5, 128.4, 127.8, 127.4, 123.2, 55.7, 54.1, 52.3, 38.8, 21.4. **HR–MS** (ESI) *m/z* calc. for $C_{19}H_{20}NO_4S_2$ $[M+H]^+$: 390.0828, found: 390.0826.

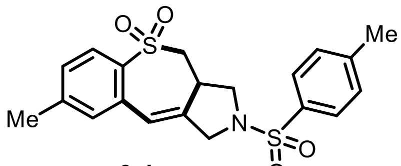

3ab

8-Methyl-2-tosyl-2,3,3a,4-tetrahydro-1*H*-benzo[6,7]thiepino[3,4-*c*]pyrrole 5,5-dioxide (3ab)

The general procedure (A) was followed using **1a** (107 mg, 0.60 mmol), **2b** (74.7 mg, 0.3 mmol) and Na_2CO_3 (31.8 mg, 0.30 mmol). Purification by column chromatography on silica gel (petroleum ether/ EtOAc = 3:1) yielded **3ab** (88.3 mg, 73%) as a white solid. **M.p.**: 193–195 °C. **^{1}H NMR** (400 MHz, $CDCl_3$) δ 8.01 (d, J = 8.1 Hz, 1H), 7.76 (d, J = 8.2 Hz, 2H), 7.38 (d, J = 8.1 Hz, 2H), 7.23 (d, J = 8.0 Hz, 1H), 7.10 (s, 1H), 6.44 (s, 1H), 4.21 (d, J = 14.5 Hz, 1H), 4.06 (d, J = 14.5 Hz, 1H), 3.73 (dd, J = 9.3, 7.5 Hz, 1H), 3.55 (dd, J = 12.7, 3.6 Hz, 1H), 3.43–3.32 (m, 2H), 3.08 (t, J = 9.0 Hz, 1H), 2.46 (s, 3H), 2.41 (s, 3H). **^{13}C NMR** (151 MHz, $CDCl_3$) δ 144.6, 144.4, 142.3, 136.6, 133.5, 132.8, 132.5, 130.1, 128.4, 127.9, 127.5, 123.1, 55.9, 54.2, 52.4, 38.8, 21.7, 21.5. **HR–MS** (ESI) *m/z* calc. for $C_{20}H_{22}NO_4S_2$ $[M+H]^+$: 404.0985, found: 404.0984.

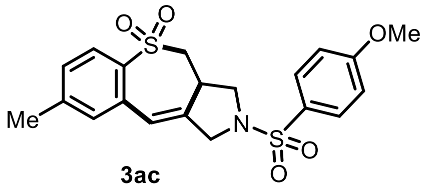

2-((4-Methoxyphenyl)sulfonyl)-8-methyl-2,3,3a,4-tetrahydro-1*H*-benzo[6,7]thiepino[3,4-*c*]pyrrole 5,5-dioxide (3ac)

The general procedure (A) was followed using **1a** (107 mg, 0.60 mmol), **2c** (79.5 mg, 0.3 mmol) and Na_2CO_3 (31.8 mg, 0.30 mmol). Purification by column chromatography on silica gel (petroleum ether/ EtOAc = 3:1) yielded **3ac** (75.4 mg, 60%) as a yellow soild. **M.P.**: 201–203 °C. **^{1}H NMR** (400 MHz, $CDCl_3$) δ 8.01 (d, J = 8.1 Hz, 1H), 7.81 (d, J = 8.8 Hz, 2H), 7.22 (d, J = 8.1 Hz, 1H), 7.10 (s, 1H), 7.04 (d, J = 8.9 Hz, 2H), 6.44 (d, J = 1.3 Hz, 1H), 4.20 (d, J = 14.5 Hz, 1H), 4.05 (d, J = 14.5 Hz, 1H), 3.90 (s, 3H), 3.72 (dd, J = 9.3, 7.5 Hz, 1H), 3.55 (dd, J = 12.7, 3.6 Hz, 1H), 3.44–3.36 (m, 2H), 3.07 (t, J = 9.0 Hz, 1H), 2.41 (s, 3H). **^{13}C NMR** (151 MHz, $CDCl_3$) δ 163.6, 144.6, 142.4, 136.7, 133.4, 132.6, 130.0, 128.4, 127.5, 127.4, 123.1, 114.7, 56.0, 55.8, 54.2, 52.4, 38.8, 21.5. **HR–MS** (ESI) *m/z* calc. for $C_{20}H_{22}NO_5S_2$ $[M+H]^+$: 420.0934, found: 420.0928.

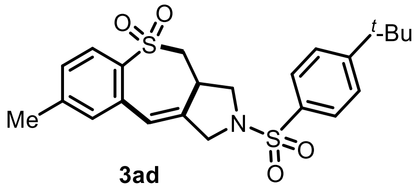

2-((4-(Tert-butyl)phenyl)sulfonyl)-8-methyl-2,3,3a,4-tetrahydro-1*H*-benzo[6,7]thiepino[3,4-*c*]pyrrole 5,5-dioxide (3ad)

The general procedure (A) was followed using **1a** (107 mg, 0.60 mmol), **2d** (87.3 mg, 0.3 mmol) and Na_2CO_3 (31.8 mg, 0.30 mmol). Purification by column chromatography on silica gel (petroleum ether/ EtOAc = 3:1) yielded **3ad** (72.1 mg, 54%) as a white solid. **M.p.**: 232–234 °C. **^{1}H NMR** (400 MHz, $CDCl_3$) δ 8.00 (d, J = 8.1 Hz, 1H), 7.78 (d, J = 8.4 Hz, 2H), 7.58 (d, J = 8.5 Hz, 2H), 7.22 (d, J = 8.1 Hz, 1H), 7.10 (s, 1H), 6.45 (d, J = 1.3 Hz, 1H), 4.22 (d, J = 14.4 Hz, 1H), 4.08 (d, J = 14.5 Hz, 1H), 3.74 (dd, J = 9.3, 7.5 Hz, 1H), 3.56 (dd, J = 12.8, 3.7 Hz, 1H), 3.44–3.35 (m, 2H), 3.11 (t, J = 9.2 Hz, 1H), 2.40 (s, 3H), 1.36 (s, 9H). **^{13}C NMR** (151 MHz, $CDCl_3$) δ 157.3, 144.6, 142.4, 136.6, 133.4, 132.6, 130.3, 128.4, 127.7, 127.5, 126.5, 123.1, 56.0, 54.2, 52.3, 38.8, 35.4, 31.2, 21.4. **HR–MS** (ESI) *m/z* calc. for $C_{23}H_{28}NO_4S_2$ $[M+H]^+$: 446.1454, found: 446.1451.

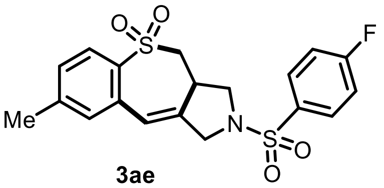

2-((4-Fluorophenyl)sulfonyl)-8-methyl-2,3,3a,4-tetrahydro-1*H*-benzo[6,7]thiepino[3,4-*c*]pyrrole 5,5-dioxide (3ae)

The general procedure (A) was followed using **1a** (107 mg, 0.60 mmol), **2e** (75.9 mg, 0.3 mmol) and Na_2CO_3 (31.8 mg, 0.30 mmol). Purification by column chromatography on silica gel (petroleum ether/ EtOAc = 3:1) yielded **3ae** (91.6 mg, 75%) as a white solid. **M.p.**: 201–203 °C. **^{1}H NMR** (400 MHz, $CDCl_3$) δ 8.00 (d, J = 8.0 Hz, 1H), 7.89 (dd, J = 8.8, 5.0 Hz, 2H), 7.25(dd, J = 17.6, 9.2 Hz, 3H), 7.10 (s, 1H), 6.44 (d, J = 1.7 Hz, 1H), 4.22 (d, J = 14.4 Hz, 1H), 4.05 (d, J = 14.5 Hz, 1H), 3.74 (dd, J = 9.3, 7.6 Hz, 1H), 3.56 (dd, J = 13.2, 4.3 Hz, 1H), 3.47–3.41 (m, 1H), 3.38 (d, J = 13.2 Hz, 1H), 3.09 (t, J = 9.1 Hz, 1H), 2.40 (s, 3H). **^{13}C NMR** (101 MHz, $CDCl_3$) δ 165.6 (d, J = 256.5 Hz), 144.7, 141.8, 136.6, 133.5, 132.4, 130.5, 130.4, 128.4, 127.4, 123.3, 116.8 (d, J = 22.2 Hz), 55.5, 54.1, 52.3, 38.9, 21.4. **^{19}F NMR** (377 MHz, $CDCl_3$) δ –103.99. **HR–MS** (ESI) *m/z* calc. for $C_{19}H_{19}FNO_4S_2$ $[M+H]^+$:408.0734, found: 408.0734.

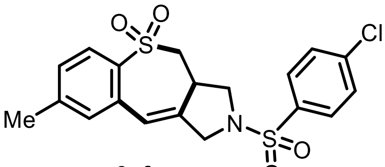

2-((4-Chlorophenyl)sulfonyl)-8-methyl-2,3,3a,4-tetrahydro-1*H*-benzo[6,7]thiepino[3,4-*c*]pyrrole 5,5-dioxide (3af)

The general procedure (A) was followed using **1a** (107 mg, 0.60 mmol), **2f** (80.7 mg, 0.3 mmol) and Na_2CO_3 (31.8 mg, 0.30 mmol). Purification by column chromatography on silica gel (petroleum ether/ EtOAc = 3:1) yielded **3af** (88.8 mg, 70%) as a white solid. **M.p.**: 235–237 °C. **^{1}H NMR** (400 MHz, $CDCl_3$) δ 8.02 (d, J = 8.1 Hz, 1H), 7.82 (d, J = 8.5 Hz, 2H), 7.57 (d, J = 8.4 Hz, 2H), 7.24 (d, J = 8.0 Hz, 1H), 7.11 (s, 1H), 6.45 (s, 1H), 4.24 (d, J = 14.4 Hz, 1H), 4.07 (d, J = 14.4 Hz, 1H), 3.74 (dd, J = 8.0, 8.0 Hz, 1H), 3.56 (dd, J = 13.3, 4.4 Hz, 1H), 3.52–3.47 (m, 1H), 3.39 (d, J = 12.8 Hz, 1H), 3.10 (t, J = 9.1 Hz, 1H), 2.42 (s, 3H). **^{13}C NMR** (101 MHz, $CDCl_3$) δ 144.7, 141.7, 140.2, 136.7, 134.6, 133.6, 132.4, 129.9, 129.2, 128.5, 127.6, 123.4, 55.6, 54.1, 52.3, 38.9, 21.5. **HR–MS** (ESI) *m/z* calc. for $C_{19}H_{19}ClNO_4S_2$ $[M+H]^+$: 424.0439, found: 424.0435.

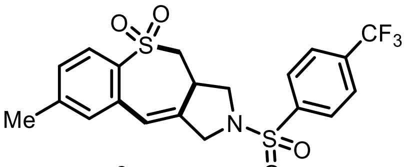

8-Methyl-2-((4-(trifluoromethyl)phenyl)sulfonyl)-2,3,3a,4-tetrahydro-1*H*-benzo[6,7]thiepino[3,4-*c*]pyrrole 5,5-dioxide (3ag)

The general procedure (A) was followed using **1a** (107 mg, 0.60 mmol), **2g** (90.9 mg, 0.3 mmol) and Na_2CO_3 (31.8 mg, 0.30 mmol). Purification by column chromatography on silica gel (petroleum ether/ EtOAc = 3:1) yielded **3ag** (87.7 mg, 64%) as a white solid. **M.p.**: 252–254 °C. **^{1}H NMR** (400 MHz, DMSO) *δ* 8.09–8.02 (m, 4H), 7.85 (d, *J* = 8.0 Hz, 1H), 7.30 (d, *J* = 8.2 Hz, 1H), 7.26 (s, 1H), 6.55 (s, 1H), 4.30 (d, *J* = 14.9 Hz, 1H), 4.02 (d, *J* = 14.9 Hz, 1H), 3.84 (dd, *J* = 8.7, 8.7 Hz, 1H), 3.73 (dd, *J* = 14.6, 4.9 Hz, 1H), 3.57 (dd, *J* = 14.5, 12.0 Hz, 1H), 3.24–3.17 (m, 1H), 3.06 (t, *J* = 9.9 Hz, 1H), 2.35 (s, 3H). **^{13}C NMR** (151 MHz, DMSO) *δ* 144.4, 142.7, 140.0, 137.4, 134.1, 133.4 (q, *J* = 33.2 Hz), 132.2, 129.0, 128.5, 127.2 (q, *J* = 3..0 Hz), 126.6, 123.9 (q, *J* = 273.3 Hz), 122.7, 54.6, 52.7, 52.2, 39.1, 21.2. **^{19}F NMR** (565 MHz, DMSO) *δ* –61.68. **HR–MS** (ESI) *m/z* calc. for $C_{20}H_{19}F_3NO_4S_2$ $[M+H]^+$: 458.0702, found: 458.0700.

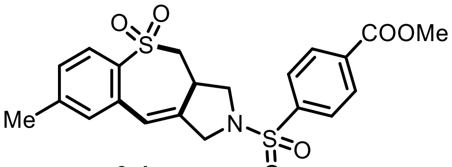

Methyl-4-((8-methyl-5,5-dioxido-3a,4-dihydro-1*H*-benzo[6,7]thiepino[3,4-*c*]pyrrol-2(3*H*)-yl)sulfonyl)benzoate (3ah)

The general procedure (A) was followed using **1a** (107 mg, 0.60 mmol), **2h** (87.9 mg, 0.3 mmol) and Na_2CO_3 (31.8 mg, 0.30 mmol). Purification by column chromatography on silica gel (petroleum ether/ EtOAc = 3:1) yielded **3ah** (95.2 mg, 71%) as a white solid. **M.p.**: 95–97 °C. **^{1}H NMR** (400 MHz, $CDCl_3$) *δ* 8.23 (d, *J* = 8.2 Hz, 2H), 7.91 (d, *J* = 8.2 Hz, 2H), 7.79 (d, *J* = 8.0 Hz, 2H), 7.40 (d, *J* = 8.0 Hz, 2H), 3.99 (s, 3H), 3.89 (d, *J* = 14.3 Hz, 1H), 3.79 (d, *J* = 16.0 Hz, 1H), 3.75 (dd, *J* = 10.4, 7.2 Hz, 1H), 3.24–3.20 (m, 2H), 3.11–3.00 (m, 2H), 2.49 (s, 3H). **^{13}C NMR** (151 MHz, $CDCl_3$) *δ* 165.7, 145.5, 145.0, 139.6, 136.1, 134.4, 130.5, 130.3, 128.1, 127.9, 109.1, 58.6, 53.0, 52.9, 51.5, 37.4, 21.8. **HR–MS** (ESI) *m/z* calc. for $C_{21}H_{22}NO_6S_2$ $[M+H]^+$: 448.0883, found: 448.0880.

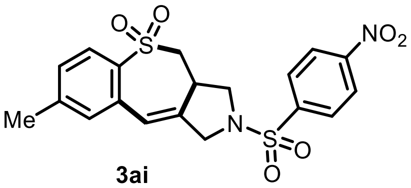

8-Methyl-2-((4-nitrophenyl)sulfonyl)-2,3,3a,4-tetrahydro-1*H*-benzo[6,7]thiepino[3,4-*c*]pyrrole 5,5-dioxide (3ai)

The general procedure (A) was followed using **1a** (107 mg, 0.60 mmol), **2i** (74.0 mg, 0.3 mmol) and Na_2CO_3 (31.8 mg, 0.30 mmol). Purification by column chromatography on silica gel (petroleum ether/ EtOAc = 3:1) yielded **3ai** (83.3 mg, 61%) as a white solid. **M.p.**: >250 °C. **^{1}H NMR** (400 MHz, DMSO) δ 8.44 (d, J = 8.7 Hz, 1H), 8.18 (d, J = 8.3 Hz, 1H), 8.12 (d, J = 8.7 Hz, 1H), 7.99(d, J = 8.4 Hz, 1H), 7.84 (dd, J = 8.0, 2.4 Hz, 1H), 7.30 (d, J = 8.1 Hz, 1H), 7.25 (s, 1H), 6.54 (s, 1H), 4.29 (t, J = 12.8 Hz, 1H), 4.02 (t, J = 15.4 Hz, 1H), 3.84 (dd, J = 17.1, 8.1 Hz, 1H), 3.74 (dd, J = 14.6, 4.6 Hz, 1H), 3.60–3.52 (m, 1H), 3.26–3.14 (m, 1H), 3.10–3.01 (m, 1H), 2.35 (s, 3H). **^{13}C NMR** (101 MHz, DMSO) δ 150.6, 144.4, 142.5, 137.4, 134.1, 132.2, 130.8, 129.6, 128.5, 126.6, 125.3, 122.8, 54.6, 53.2, 52.8, 39.1, 21.2. **HR–MS** (ESI) *m/z* calc. for $C_{19}H_{19}N_2O_6S_2$ [M+H]$^+$: 435.0679, found: 435.0680.

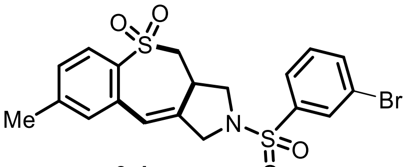

2-((3-Bromophenyl)sulfonyl)-8-methyl-2,3,3a,4-tetrahydro-1*H*-benzo[6,7]thiepino[3,4-*c*]pyrrole 5,5-dioxide (3aj)

The general procedure (A) was followed using **1a** (107 mg, 0.60 mmol), **2j** (93.6 mg, 0.3 mmol) and Na_2CO_3 (31.8 mg, 0.30 mmol). Purification by column chromatography on silica gel (petroleum ether/ EtOAc = 3:1) yielded **3aj** (100.7 mg, 72%) as a yellow solid. **M.p.**: 184–186 °C. **^{1}H NMR** (400 MHz, $CDCl_3$) δ 8.02–8.00 (m, 2H), 7.80 (dd, J = 7.9, 1.3 Hz, 2H), 7.46 (dd, J = 7.9, 7.9 Hz, 1H), 7.23 (d, J = 8.0 Hz, 1H), 7.12 (s, 1H), 6.46 (d, J = 1.8 Hz, 1H), 4.25 (d, J = 14.4 Hz, 1H), 4.08 (d, J = 14.4 Hz, 1H), 3.76 (dd, J = 9.3, 7.6 Hz, 1H), 3.57 (dd, J = 13.4, 4.6 Hz, 1H), 3.51–3.46 (m, 1H), 3.37 (dd, J = 13.3, 11.9 Hz, 1H), 3.11 (t, J = 9.1 Hz, 1H), 2.41 (s, 3H). **^{13}C NMR** (101 MHz, $CDCl_3$) δ 144.7, 141.6, 137.9, 136.6, 136.5, 133.6, 132.3, 131.0, 130.6, 128.5, 127.5, 126.2, 123.6, 123.4, 55.5, 54.1, 52.3, 38.9, 21.5. **HR–MS** (ESI) *m/z* calc. for $C_{19}H_{19}BrNO_4S_2$ [M+H]$^+$: 467.9937, found: 467.9930.

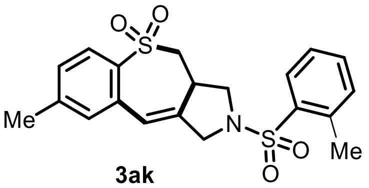

3ak

8-Methyl-2-(o-tolylsulfonyl)-2,3,3a,4-tetrahydro-1*H*-benzo[6,7]thiepino[3,4-*c*]pyrrole 5,5-dioxide (3ak)

The general procedure (A) was followed using **1a** (107 mg, 0.60 mmol), **2k** (74.7 mg, 0.3 mmol) and Na_2CO_3 (31.8 mg, 0.30 mmol). Purification by column chromatography on silica gel (petroleum ether/ EtOAc = 3:1) yielded **3ak** (82.2 mg, 68%) as a white solid. **M.p.**: 154–156 °C. **^{1}H NMR** (400 MHz, $CDCl_3$) δ 8.02 (d, J = 8.1 Hz, 1H), 7.97 (d, J = 7.4 Hz, 1H), 7.52 (dd, J = 7.4, 7.4 Hz, 1H), 7.37 (d, J = 6.7 Hz, 2H), 7.23 (d, J = 8.1 Hz, 1H), 7.13 (s, 1H), 6.48 (d, J = 1.3 Hz, 1H), 4.26 (d, J = 14.5 Hz, 1H), 4.16 (d, J = 14.6 Hz, 1H), 3.74 (dd, J = 7.2, 7.2 Hz, 1H), 3.68 (dd, J = 15.2, 4.4 Hz, 2H), 3.41 (dd, J = 15.1, 13.5 Hz, 1H), 3.20 (t, J = 8.8 Hz, 1H), 2.68 (s, 3H), 2.42 (s, 3H). **^{13}C NMR** (151 MHz, $CDCl_3$) δ 144.6, 142.4, 138.3, 136.6, 135.9, 133.5, 133.5, 133.0, 132.5, 130.0, 128.4, 127.4, 126.4, 123.1, 55.6, 53.5, 51.6, 39.2, 21.4, 20.7. **HR–MS** (ESI) *m/z* calc. for $C_{20}H_{22}NO_4S_2$ $[M+H]^+$: 404.0985, found: 404.0984.

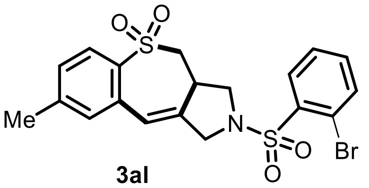

3al

2-((2-Bromophenyl)sulfonyl)-8-methyl-2,3,3a,4-tetrahydro-1*H*-benzo[6,7]thiepino[3,4-*c*]pyrrole 5,5-dioxide (3al)

The general procedure (A) was followed using **1a** (107 mg, 0.60 mmol), **2l** (93.6 mg, 0.3 mmol) and Na_2CO_3 (31.8 mg, 0.30 mmol). Purification by column chromatography on silica gel (petroleum ether/EtOAc = 3:1) yielded **3al** (83.9 mg, 60%) as a yellow solid. **M.p.**: 141–143 °C. **^{1}H NMR** (400 MHz, $CDCl_3$) δ 8.15 (d, J = 7.7 Hz, 1H), 8.02 (d, J = 8.1 Hz, 1H), 7.78 (d, J = 7.7 Hz, 1H), 7.50–7.45 (m, 2H), 7.23 (d, J = 8.1 Hz, 1H), 7.14 (s, 1H), 6.48 (s, 1H), 4.37 (d, J = 14.4 Hz, 1H), 4.25 (d, J = 14.4 Hz, 1H), 3.92 (dd, J = 8.8, 8.8 Hz, 1H),, 3.63–3.55 (m, 2H), 3.43 (t, J = 13.3 Hz, 1H), 3.33 (t, J = 9.3 Hz, 1H), 2.41 (s, 3H). **^{13}C NMR** (101 MHz, $CDCl_3$) δ 144.6, 142.2, 137.7, 136.7, 135.9, 134.2, 133.7, 132.4, 132.3, 128.4, 127.8, 127.4, 123.1, 120.7, 55.0, 53.8, 52.0, 39.4, 21.4. **HR–MS** (ESI) *m/z* calc. for $C_{20}H_{22}NO_4S_2$ $[M+H]^+$: 404.0985, found: 404.0984.

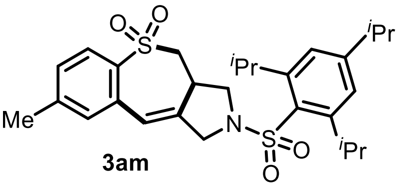

3am

8-Methyl-2-((2,4,6-triisopropylphenyl)sulfonyl)-2,3,3a,4-tetrahydro-1*H*-benzo[6,7]thiepino[3,4-*c*]pyrrole 5,5-dioxide (3am)

The general procedure (A) was followed using **1a** (107 mg, 0.60 mmol), **2m** (108.3 mg, 0.3 mmol) and Na_2CO_3 (31.8 mg, 0.30 mmol). Purification by column chromatography on silica gel (petroleum ether/EtOAc = 3:1) yielded **3am** (89.6 mg, 58%) as a white solid. **M.p.**: 60–62 °C. **^{1}H NMR** (400 MHz, $CDCl_3$) δ 8.04 (d, J = 8.1 Hz, 1H), 7.26–7.20 (m, 3H), 7.14 (s, 1H), 6.50 (s, 1H), 4.26–4.19 (m, 3H), 4.15 (d, J = 14.5 Hz, 1H), 3.68–3.50 (m, 3H), 3.52–3.44 (m, 1H), 3.23 (t, J = 8.3 Hz, 1H), 2.94 (d, J = 6.8 Hz, 1H), 2.43 (s, 3H), 1.29 (dd, J = 6.8, 2.9 Hz, 18H). **^{13}C NMR** (151 MHz, $CDCl_3$) δ 153.8, 151.7, 144.6, 142.8, 136.6, 133.4, 132.9, 130.3, 128.3, 127.5, 124.1, 123.0, 56.6, 52.8, 51.0, 39.0, 34.3, 29.6, 25.1, 25.0, 23.7, 21.5. **HR–MS** (ESI) *m/z* calc. for $C_{28}H_{38}NO_4S_2$ $[M+H]^+$: 516.2237, found: 516.2237.

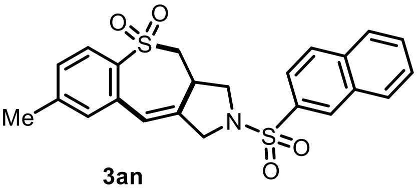

3an

8-Methyl-2-(naphthalen-2-ylsulfonyl)-2,3,3a,4-tetrahydro-1*H*-benzo[6,7]thiepino[3,4-*c*]pyrrole 5,5-dioxide (3an)

The general procedure (A) was followed using **1a** (107 mg, 0.60 mmol), **2n** (85.5 mg, 0.3 mmol) and Na_2CO_3 (31.8 mg, 0.30 mmol). Purification by column chromatography on silica gel (petroleum ether/ EtOAc = 3:1) yielded **3an** (92.2 mg, 70%) as a white solid. **M.p.**: 232–234 °C. **^{1}H NMR** (400 MHz, DMSO) δ 8.54 (s, 1H), 8.24 (d, J = 7.7 Hz, 1H), 8.17 (d, J = 8.7 Hz, 1H), 8.07 (d, J = 7.8 Hz, 1H), 7.87 (dd, J = 8.6, 1.3 Hz, 1H), 7.81 (d, J = 8.0 Hz, 1H), 7.75–7.68 (m, 2H), 7.25 (d, J = 8.1 Hz, 1H), 7.14 (s, 1H), 6.50 (s, 1H), 4.31 (d, J = 15.1 Hz, 1H), 4.03 (d, J = 15.1 Hz, 1H), 3.89 (dd, J = 8.4, 8.4 Hz, 1H), 3.73 (dd, J = 14.6, 4.9 Hz, 1H), 3.54 (dd, J = 14.5, 12.1 Hz, 1H), 3.19–3.17 (m, 1H), 3.06 (t, J = 9.9 Hz, 1H), 2.30 (s, 3H). **^{13}C NMR** (101 MHz, DMSO) δ 144.3, 143.0, 137.4, 135.1, 134.0, 133.1, 132.3, 132.2, 130.1, 129.9, 129.6, 129.4, 128.4, 128.3, 128.2, 126.5, 123.3, 122.5, 54.6, 52.8, 52.4, 39.2, 21.2. **HR–MS** (ESI) *m/z* calc. for $C_{23}H_{22}NO_4S_2$ $[M+H]^+$: 440.0985, found: 440.0985.

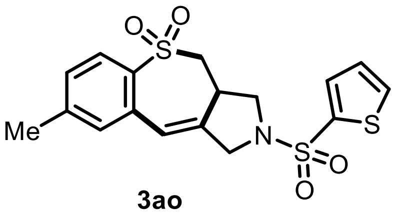

3ao

8-Methyl-2-(thiophen-2-ylsulfonyl)-2,3,3a,4-tetrahydro-1*H*-benzo[6,7]thiepino[3,4-*c*]pyrrole 5,5-dioxide (3ao)

The general procedure (A) was followed using **1a** (107 mg, 0.60 mmol), **2o** (72.3 mg, 0.3 mmol) and Na_2CO_3 (31.8 mg, 0.30 mmol). Purification by column chromatography on silica gel (petroleum ether/ EtOAc = 3:1) yielded **3ao** (59.3 mg, 50%) as a white solid. **M.p.**: 235–237 °C. **^{1}H NMR** (400 MHz, $CDCl_3$) δ 8.03 (d, *J* = 8.1 Hz, 1H), 7.68 (d, *J* = 5.0 Hz, 1H), 7.67 (d, *J* = 3.7 Hz, 1H), 7.24 (d, *J* = 8.1 Hz, 1H), 7.21 (dd, *J* = 4.0, 4.0 Hz, 1H), 7.13 (s, 1H), 6.48 (d, *J* = 1.3 Hz, 1H), 4.30 (d, *J* = 14.6 Hz, 1H), 4.14 (d, *J* = 14.6 Hz, 1H), 3.81 (dd, *J* = 9.5, 7.8 Hz, 1H), 3.56 (dd, *J* = 13.4, 4.3 Hz, 1H), 3.47–3.33 (m, 2H), 3.17 (t, *J* = 9.4 Hz, 1H), 2.42 (s, 3H). **^{13}C NMR** (151 MHz, $CDCl_3$) δ 144.7, 141.8, 136.7, 135.8, 133.7, 133.1, 132.8, 132.3, 128.5, 128.0, 127.5, 123.3. 55.3, 54.5, 52.5, 38.9, 21.5. **HR–MS** (ESI) *m/z* calc. for $C_{17}H_{18}NO_4S_3$ $[M+H]^+$: 396.0393, found: 396.0392.

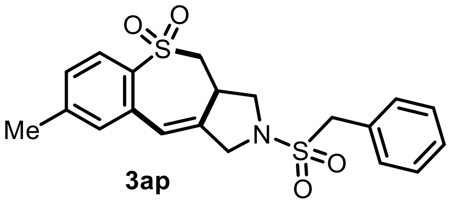

3ap

2-(Benzylsulfonyl)-8-methyl-2,3,3a,4-tetrahydro-1*H*-benzo[6,7]thiepino[3,4-*c*]pyrrole 5,5-dioxide (3ap)

The general procedure (A) was followed using **1a** (107 mg, 0.60 mmol), **2p** (74.7 mg, 0.3 mmol) and Na_2CO_3 (31.8 mg, 0.30 mmol). Purification by column chromatography on silica gel (petroleum ether/ EtOAc = 3:1) yielded **3ap** (65.3 mg, 54%) as a white solid. **M.p.**: 158–160 °C. **^{1}H NMR** (400 MHz, $CDCl_3$) δ 8.02 (d, *J* = 8.1 Hz, 1H), 7.43–7.39 (m, 5H), 7.24 (d, *J* = 8.2 Hz, 1H), 7.14 (s, 1H), 6.39 (s, 1H), 4.34 (s, 2H), 4.17 (d, *J* = 14.6 Hz, 1H), 4.00 (d, *J* = 14.4 Hz, 1H), 3.57 (dd, *J* = 7.6, 7.6 Hz, 1H), 3.49–3.42 (m, 2H), 3.28 (dd, *J* = 15.2, 12.8 Hz,, 1H), 3.06 (t, *J* = 9.0 Hz, 1H), 2.42 (s, 3H). **^{13}C NMR** (101 MHz, $CDCl_3$) δ 144.6, 142.2, 136.8, 133.8, 132.2, 130.8, 129.1, 129.0, 128.9, 128.4, 127.3, 122.9, 57.6, 54.5, 54.4, 52.5, 39.3, 21.4. **HR–MS** (ESI) *m/z* calc. for $C_{20}H_{22}NO_4S_2$ $[M+H]^+$: 404.0985, found: 404.0985.

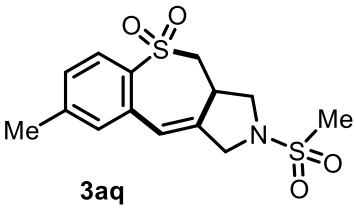

3aq

8-Methyl-2-(methylsulfonyl)-2,3,3a,4-tetrahydro-1*H*-benzo[6,7]thiepino[3,4-*c*]pyrrole 5,5-dioxide (3aq)

The general procedure (A) was followed using **1a** (107 mg, 0.60 mmol), **2q** (51.9 mg, 0.3 mmol) and Na_2CO_3 (31.8 mg, 0.30 mmol). Purification by column chromatography on silica gel (petroleum ether/ EtOAc = 3:1) yielded **3aq** (46.1 mg, 47%) as a white solid. **M.p.**: 148–150 °C. **^{1}H NMR** (400 MHz, $CDCl_3$) δ 8.05 (d, J = 8.1 Hz, 1H), 7.26 (d, J = 8.1 Hz, 1H), 7.18 (s, 1H), 6.54 (d, J = 1.2 Hz, 1H), 4.36 (d, J = 14.6 Hz, 1H), 4.20 (d, J = 14.6 Hz, 1H), 3.84 (dd, J = 9.6, 7.6 Hz, 1H), 3.64–3.58 (m, 2H), 3.45 (dd, J = 15.2, 12.8 Hz, 1H), 3.28 (t, J = 9.2 Hz. 1H), 2.94 (s, 3H), 2.44 (s, 3H). **^{13}C NMR** (151 MHz, DMSO) δ 144.5, 143.9, 137.5, 134.3, 132.3, 128.5, 126.6, 122.3, 54.6, 52.5, 52.0, 39.5, 34.1, 21.2. **HR–MS** (ESI) *m/z* calc. for $C_{14}H_{18}NO_4S_2$ $[M+H]^+$:328.0672, found: 328.0682.

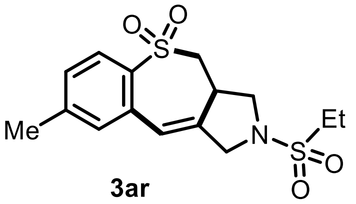

3ar

2-(Ethylsulfonyl)-8-methyl-2,3,3a,4-tetrahydro-1*H*-benzo[6,7]thiepino[3,4-*c*]pyrrole 5,5-dioxide (3ar)

The general procedure (A) was followed using **1a** (107 mg, 0.60 mmol), **2r** (56.1 mg, 0.3 mmol) and Na_2CO_3 (31.8 mg, 0.30 mmol). Purification by column chromatography on silica gel (petroleum ether/ EtOAc = 3:1) yielded **3ar** (46.0 mg, 45%) as a white solid. **M.p.**: 122–124 °C. **^{1}H NMR** (400 MHz, $CDCl_3$) δ 8.04 (d, J = 8.1 Hz, 1H), 7.25 (d, J = 8.1 Hz, 1H), 7.17 (s, 1H), 6.52 (s, 1H), 4.38 (d, J = 14.4 Hz, 1H), 4.23 (d, J = 14.5 Hz, 1H), 3.86 (dd, J = 9.4, 7.7 Hz, 1H), 3.67–3.57 (m, 2H), 3.43 (dd, J = 14.1, 11.9 Hz, 1H), 3.30 (t, J = 9.4 Hz, 1H), 3.09 (q, J = 7.4 Hz, 2H), 2.43 (s, 3H), 1.42 (t, J = 7.4 Hz, 3H). **^{13}C NMR** (151 MHz, $CDCl_3$) δ 144.7, 142.5, 136.8, 133.7, 132.3, 128.5, 127.4, 123.0, 54.9, 54.2, 52.2, 45.5, 39.6, 21.5, 8.1. **HR–MS** (ESI) *m/z* calc. for $C_{15}H_{20}NO_4S_2$ $[M+H]^+$: 342.0828, found: 342.0828.

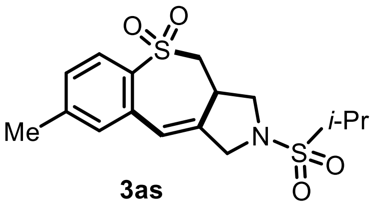

3as

2-(Isopropylsulfonyl)-8-methyl-2,3,3a,4-tetrahydro-1*H*-benzo[6,7]thiepino[3,4-*c*]pyrrole 5,5-dioxide (3as)

The general procedure (A) was followed using **1a** (107 mg, 0.60 mmol), **2s** (60.3 mg, 0.3 mmol) and Na_2CO_3 (31.8 mg, 0.30 mmol). Purification by column chromatography on silica gel (petroleum ether/ EtOAc = 3:1) yielded **3as** (54.3 mg, 51%) as a white solid. **M.p.**: 168–170 °C. **^{1}H NMR** (400 MHz, $CDCl_3$) δ 8.03 (d, J = 8.1 Hz, 1H), 7.24 (d, J = 8.2 Hz, 1H), 7.17 (s, 1H), 6.50 (d, J = 1.1 Hz, 1H), 4.41 (d, J = 14.4 Hz, 1H), 4.25 (d, J = 14.5 Hz, 1H), 3.87 (dd, J = 9.6, 7.6 Hz, 1H), 3.64–3.56 (m, 2H), 3.42 (dd, J = 14.0, 12.0 Hz, 1H), 3.32 (t, J = 9.6 Hz, 1H), 3.28 (t, J = 6.8 Hz, 1H), 2.43 (s, 3H), 1.41 (s, 3H), 1.40 (s, 3H). **^{13}C NMR** (101 MHz, $CDCl_3$) δ 144.6, 142.8, 136.7, 133.8, 132.3, 128.4, 127.3, 122.8, 54.6, 54.0, 52.7, 39.7, 21.4, 16.7. **HR–MS** (ESI) *m/z* calc. for $C_{16}H_{22}NO_4S_2$ $[M+H]^+$: 356.0985 found: 356.0983.

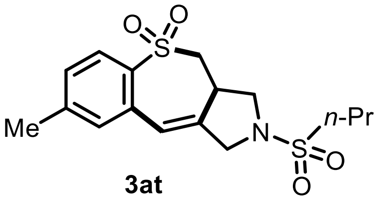

3at

8-Methyl-2-(propylsulfonyl)-2,3,3a,4-tetrahydro-1*H*-benzo[6,7]thiepino[3,4-*c*]pyrrole 5,5-dioxide (3at)

The general procedure (A) was followed using **1a** (107 mg, 0.60 mmol), **2t** (60.3 mg, 0.3 mmol) and Na_2CO_3 (31.8 mg, 0.30 mmol). Purification by column chromatography on silica gel (petroleum ether/ EtOAc = 3:1) yielded **3at** (47.9 mg, 45%) as a yellow solid. **M.p.**: 142–144 °C. **^{1}H NMR** (400 MHz, $CDCl_3$) δ 8.03 (d, J = 8.1 Hz, 1H), 7.25 (d, J = 8.1 Hz, 1H), 7.17 (s, 1H), 6.51 (d, J = 1.6 Hz, 1H), 4.36 (d, J = 14.5 Hz, 1H), 4.21 (d, J = 14.5 Hz, 1H), 3.84 (dd, J = 9.3, 7.7 Hz, 1H), 3.62–3.57 (m, 2H), 3.43 (dd, J = 15.3, 13.0 Hz, 1H), 3.28 (t, J = 9.4 Hz, 1H), 3.05–3.01 (m, 2H), 2.42 (s, 3H), 1.94–1.84 (m, 2H), 1.09 (t, J = 7.4 Hz, 3H). **^{13}C NMR** (151 MHz, $CDCl_3$) δ 144.7, 142.5, 136.7, 133.7, 132.2, 128.4, 127.3, 123.0, 54.8, 54.0, 52.4, 52.0, 39.5, 21.4, 17.1, 13.1. **HR–MS** (ESI) *m/z* calc. for $C_{16}H_{22}NO_4S_2$ $[M+H]^+$: 356.0985 found: 356.0983.

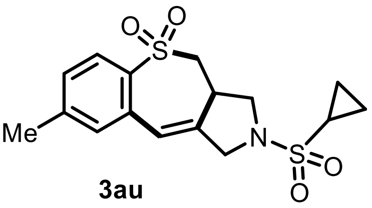

3au

2-(Cyclopropylsulfonyl)-8-methyl-2,3,3a,4-tetrahydro-1*H*-benzo[6,7]thiepino[3,4-*c*]pyrrole 5,5-dioxide (3au)

The general procedure (A) was followed using **1a** (107 mg, 0.60 mmol), **2u** (59.7 mg, 0.3 mmol) and Na_2CO_3 (31.8 mg, 0.30 mmol). Purification by column chromatography on silica gel (petroleum ether/ EtOAc = 3:1) yielded **3au** (53.0 mg, 50%) as a white solid. **M.p.**: 177–179 °C. **^{1}H NMR** (400 MHz, $CDCl_3$) δ 8.05 (d, *J* = 8.1 Hz, 1H), 7.25 (d, *J* = 8.1 Hz, 1H), 7.17 (s, 1H), 6.53 (d, *J* = 1.4 Hz, 1H), 4.39 (d, *J* = 14.6 Hz, 1H), 4.25 (d, *J* = 14.6 Hz, 1H), 3.85 (dd, *J* = 7.6, 7.6 Hz, 1H), 3.65–3.60 (m, 2H), 3.47 (dd, *J* = 15.2, 13,2 Hz,, 1H), 3.31 (t, *J* = 9.3 Hz, 1H), 2.43 (s, 3H), 1.27–1.22 (m, 3H), 1.08–1.03 (m, 2H). **^{13}C NMR** (151 MHz, $CDCl_3$) δ 144.7, 142.6, 136.7, 133.6, 132.5, 128.4, 127.4, 123.0, 55.4, 54.2, 52.3, 39.3, 26.9, 21.5, 4.9, 4.9. **HR–MS** (ESI) *m/z* calc. for $C_{16}H_{20}NO_4S_2$ $[M+H]^+$: 354.0838, found: 354.0830.

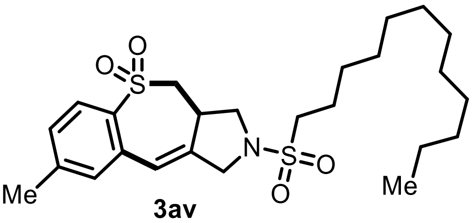

3av

2-(Dodecylsulfonyl)-8-methyl-2,3,3a,4-tetrahydro-1*H*-benzo[6,7]thiepino[3,4-*c*]pyrrole 5,5-dioxide (3av)

The general procedure (A) was followed using **1a** (107 mg, 0.60 mmol), **2v** (98.1 mg, 0.3 mmol) and Na_2CO_3 (31.8 mg, 0.30 mmol). Purification by column chromatography on silica gel (petroleum ether/ EtOAc = 3:1) yielded **3av** (71.1 mg, 49%) as a white solid. **M.p.**: 97–99 °C. **^{1}H NMR** (400 MHz, $CDCl_3$) δ 8.04 (d, *J* = 8.1 Hz, 1H), 7.25 (d, *J* = 8.1 Hz, 1H), 7.17 (s, 1H), 6.51 (s, 1H), 4.37 (d, *J* = 14.5 Hz, 1H), 4.22 (d, *J* = 14.5 Hz, 1H), 3.85 (dd, *J* = 7.6, 7.6 Hz, 1H), 3.64–3.57 (m, 2H), 3.43 (dd, *J* = 15.2, 12.8 Hz, 1H), 3.29 (t, *J* = 9.5 Hz, 1H), 3.04 (dd, *J* = 9.4, 6.5 Hz, 2H), 2.43 (s, 3H), 1.87–1.81 (m, 2H), 1.45–1.40 (m, 2H), 1.27 (s, 16H), 0.88 (t, *J* = 6.4 Hz, 3H). **^{13}C NMR** (151 MHz, $CDCl_3$) δ 144.6, 142.5, 136.8, 133.7, 132.3, 128.4, 127.4, 123.0, 54.8, 54.1, 52.1, 50.8, 39.5, 32.0, 29.7, 29.6, 29.4, 29.2, 28.5, 23.4, 22.8, 21.4, 14.2. **HR–MS** (ESI) *m/z* calc. for $C_{25}H_{38}NO_4S_2$ $[M+H]^+$: 482.2394, found: 482.2392.

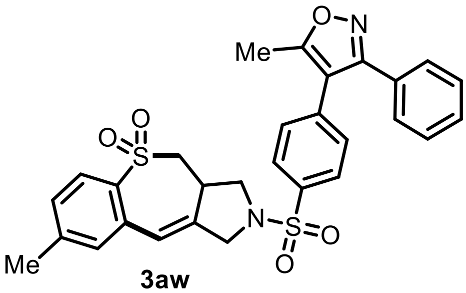

3aw

8-Methyl-2-((4-(5-methyl-3-phenylisoxazol-4-yl)phenyl)sulfonyl)-2,3,3a,4-tetrahydro-1*H*-benzo[6,7]thiepino[3,4-*c*]pyrrole 5,5-dioxide (3aw)

The general procedure (A) was followed using **1a** (107 mg, 0.60 mmol), **2w** (117.6 mg, 0.3 mmol) and Na_2CO_3 (31.8 mg, 0.30 mmol). Purification by column chromatography on silica gel (petroleum ether/ EtOAc = 3:1) yielded **3aw** (86.8 mg, 53%) as a yellow solid. **M.p.**: 207–209 °C. **^{1}H NMR** (400 MHz, $CDCl_3$) δ 8.00 (d, *J* = 8.1 Hz, 1H), 7.85 (d, *J* = 8.5 Hz, 2H), 7.38–7.35 (m, 7H), 7.23 (d, *J* = 8.1 Hz, 1H), 7.12 (s, 1H), 6.46 (d, *J* = 1.5 Hz, 1H), 4.26 (d, *J* = 14.6 Hz, 1H), 4.12 (d, *J* = 14.6 Hz, 1H), 3.80 (dd, *J* = 9.6, 7.4 Hz, 1H), 3.55 (dd, *J* = 12.7, 3.5 Hz, 1H), 3.40–3.30 (m, 2H), 3.17 (t, *J* = 9.3 Hz, 1H), 2.51 (s, 3H), 2.40 (s, 3H). **^{13}C NMR** (101 MHz, $CDCl_3$) δ 167.5, 161.2, 144.7, 142.0, 136.6, 136.0, 135.4, 133.5, 132.3, 130.6, 129.9, 128.8, 128.6, 128.5, 128.4, 128.1, 127.4, 123.2, 114.4, 55.4, 54.2, 52.3, 38.9, 21.4, 11.9. **HR–MS** (ESI) *m/z* calc. for $C_{29}H_{27}N_2O_5S_2$ $[M+H]^+$: 547.1356, found: 547.1353.

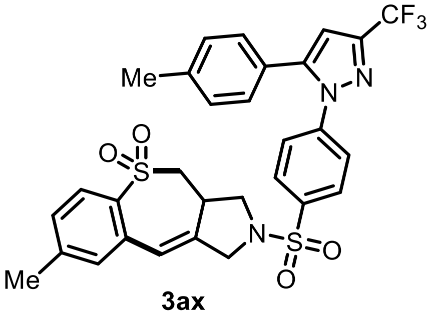

3ax

8-Methyl-2-((4-(5-(*p*-tolyl)-3-(trifluoromethyl)-1*H*-pyrazol-1-yl)phenyl)sulfonyl)-2,3,3a,4-tetrahydro-1*H*-benzo[6,7]thiepino[3,4-*c*]pyrrole 5,5-dioxide (3ax)

The general procedure (A) was followed using **1a** (107 mg, 0.60 mmol), **2x** (137.7 mg, 0.3 mmol) and Na_2CO_3 (31.8 mg, 0.30 mmol). Purification by column chromatography on silica gel (petroleum ether/ EtOAc = 3:1) yielded **3ax** (110.3 mg, 60%) as a yellow solid. **M.p.**: 77–79 °C. **^{1}H NMR** (400 MHz, $CDCl_3$) δ 8.01 (d, *J* = 8.1 Hz, 1H), 7.85 (d, *J* = 8.7 Hz, 2H), 7.54 (d, *J* = 8.7 Hz, 2H), 7.22–7.17 (m, 3H), 7.12 (d, *J* = 8.4 Hz, 3H), 6.76 (s, 1H), 6.42 (d, *J* = 1.7 Hz, 1H), 4.24 (d, *J* = 14.6 Hz, 1H), 4.03 (d, *J* = 14.6 Hz, 1H), 3.74 (dd, *J* = 9.2, 7.6 Hz, 1H), 3.54 (dd, *J* = 13.5, 4.6 Hz, 1H), 3.48–3.43 (m, 1H),

3.33 (dd, J = 13.4, 11.8 Hz, 1H), 3.06 (t, J = 9.2 Hz, 1H), 2.40 (s, 3H), 2.39 (s, 3H). **^{13}C NMR** (101 MHz, $CDCl_3$) δ 145.5, 144.7, 143.2, 141.5, 140.0, 136.7, 135.2, 133.6, 132.2, 129.9, 128.8, 128.8, 128.5, 127.4, 125.8, 125.7, 123.4, 121.1 (q, J = 270.7 Hz), 106.5, 55.2, 54.1, 52.3, 39.0, 27.0, 21.4. **^{19}F NMR** (377 MHz, $CDCl_3$) δ –62.45. **HR–MS** (ESI) *m/z* calc. for $C_{30}H_{27}F_3N_3O_4S_2$ $[M+H]^+$: 614,1392, found: 614.1390.

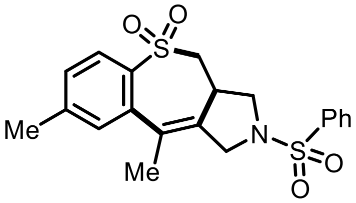

3ay

8,10-Dimethyl-2-(phenylsulfonyl)-2,3,3a,4-tetrahydro-1*H*-benzo[6,7]thiepino[3,4-*c*]pyrrole 5,5-dioxide (3ay)

The general procedure (A) was followed using **1a** (107 mg, 0.60 mmol), **2y** (74.7 mg, 0.3 mmol) and Na_2CO_3 (31.8 mg, 0.30 mmol). Purification by column chromatography on silica gel (petroleum ether/ EtOAc = 3:1) yielded **3ay** (48.4 mg, 40%) as a yellow oil. **^{1}H NMR** (400 MHz, $CDCl_3$) δ 7.93 (d, J = 8.1 Hz, 1H), 7.87 (d, J = 7.3 Hz, 2H), 7.66 (d, J = 7.3 Hz, 1H), 7.61 (d, J = 7.8 Hz, 2H), 7.23 (d, J = 7.0 Hz, 1H), 7.11 (s, 1H), 4.36 (d, J = 14.6 Hz, 1H), 3.77(dd, J = 8.4, 6.0 Hz, 1H), 3.75 (d, J = 6.9 Hz, 1H), 3.67 (d, J = 14.8 Hz, 1H), 3.31 (d, J = 9.4 Hz, 1H), 3.03 (d, J = 9.4 Hz, 1H), 2.45 (s, 1H), 2.42 (s, 3H), 2.05 (s, 3H). **^{13}C NMR** (151 MHz, $CDCl_3$) δ 144.7, 141.8, 135.0, 133.4, 130.0, 129.9, 129.4, 128.8, 128.3, 128.2, 128.0, 126.9, 68.4, 52.1, 50.7, 37.8, 21.7, 19.5. **HR–MS** (ESI) *m/z* calc. for $C_{20}H_{22}NO_4S_2$ $[M+H]^+$: 404.0985, found: 404.0982.

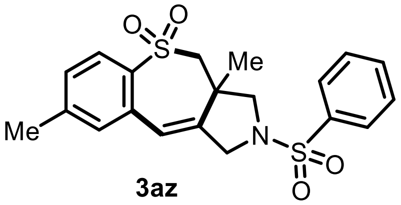

3az

3a,8-Dimethyl-2-(phenylsulfonyl)-2,3,3a,4-tetrahydro-1*H*-benzo[6,7]thiepino[3,4-*c*]pyrrole 5,5-dioxide (3az)

The general procedure (A) was followed using **1a** (107 mg, 0.60 mmol), **2z** (74.7 mg, 0.3 mmol) and Na_2CO_3 (31.8 mg, 0.30 mmol). Purification by column chromatography on silica gel (petroleum ether/ EtOAc = 3:1) yielded **3az** (50.8 mg, 42%) as a yellow oil. **^{1}H NMR** (400 MHz, $CDCl_3$) δ 7.91 (d, J = 8.1 Hz, 1H), 7.86 (d, J = 7.7 Hz, 2H), 7.65 (d, J = 7.0 Hz, 1H), 7.60 (d, J = 7.6 Hz, 2H), 7.19 (d, J = 8.1 Hz, 1H), 7.06 (s, 1H), 6.39 (s, 1H), 4.27 (d, J = 14.2 Hz, 1H), 3.98 (d, J = 14.3 Hz, 1H), 3.62 (q, J = 11.5 Hz,

2H), 3.37 (q, J = 8.9 Hz, 2H), 2.41 (s, 3H), 1.22 (s, 3H). **^{13}C NMR** (151 MHz, $CDCl_3$) δ 146.7, 144.9, 136.2, 135.6, 134.1, 133.4, 131.4, 129.4, 128.2, 127.8, 128.7, 121.3, 67.4, 60.8, 53.9, 42.9, 26.5, 21.5. **HR–MS** (ESI) m/z calc. for $C_{20}H_{22}NO_4S_2$ $[M+H]^+$: 404.0985, found: 404.0986.

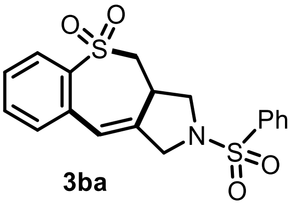

3ba

2-(Phenylsulfonyl)-2,3,3a,4-tetrahydro-1*H*-benzo[6,7]thiepino[3,4-*c*]pyrrole 5,5-dioxide (3ba)

The general procedure (A) was followed using **1b** (98.4 mg, 0.60 mmol), **2a** (70.5 mg, 0.3 mmol) and Na_2CO_3 (31.8 mg, 0.30 mmol). Purification by column chromatography on silica gel (petroleum ether/ EtOAc = 3:1) yielded **3ba** (66.4 mg, 59%) as a white solid. **M.p.**: 139–141 °C. **^{1}H NMR** (400 MHz, $CDCl_3$) δ 8.13 (d, J = 7.9 Hz, 1H), 7.88 (d, J = 7.5 Hz, 2H), 7.66 (d, J = 7.2 Hz, 1H), 7.60 (dd, J = 7.6, 7.6 Hz, 3H), 7.43 (dd, J = 7.7, 7.7 Hz, 1H), 7.30 (d, J = 7.8 Hz, 1H), 6.49 (d, J = 1.3 Hz, 1H), 4.24 (d, J = 14.9 Hz, 1H), 4.09 (d, J = 14.6 Hz, 1H), 3.76 (dd, J = 9.2, 7.5 Hz, 1H), 3.58 (dd, J = 12.9, 3.8 Hz, 1H), 3.44–3.35 (m, 2H), 3.11 (t, J = 9.0 Hz, 1H). **^{13}C NMR** (151 MHz, $CDCl_3$) δ 142.3, 139.3, 135.8, 133.8, 133.5, 132.9, 132.6, 129.5, 127.8, 127.8, 127.3, 123.1, 55.6, 54.1, 52.3, 38.8. **HR–MS** (ESI) m/z calc. for $C_{18}H_{18}NO_4S_2$ $[M+H]^+$: 376.0672, found: 376.0673.

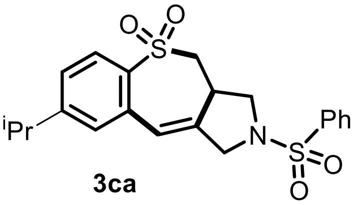

3ca

8-Isopropyl-2-(phenylsulfonyl)-2,3,3a,4-tetrahydro-1*H*-benzo[6,7]thiepino[3,4-*c*]pyrrole 5,5-dioxide (3ca)

The general procedure (A) was followed using **1c** (123.6 mg, 0.60 mmol), **2a** (70.5 mg, 0.3 mmol) and Na_2CO_3 (31.8 mg, 0.30 mmol). Purification by column chromatography on silica gel (petroleum ether/ EtOAc = 3:1) yielded **3ca** (87.6 mg, 70%) as a white solid. **M.p.**: 160–162 °C. **^{1}H NMR** (400 MHz, $CDCl_3$) δ 8.03 (d, J = 8.2 Hz, 1H), 7.87 (d, J = 7.5 Hz, 2H), 7.64 (d, J = 7.3 Hz, 1H), 7.59 (d, J = 7.8 Hz, 2H), 7.26 (d, J = 9.2 Hz, 1H), 7.13 (s, 1H), 6.48 (d, J = 1.2 Hz, 1H), 4.23 (d, J = 14.5 Hz, 1H), 4.08 (d, J =

14.5 Hz, 1H), 3.74 (dd, J = 9.2, 7.6 Hz, 1H), 3.56 (dd, J = 13.1, 4.0 Hz, 1H), 3.44–3.32 (m, 2H), 3.09 (t, J = 9.0 Hz, 1H), 2.98–2.91 (m, 1H), 1.25 (d, J = 6.9 Hz, 6H). **^{13}C NMR** (151 MHz, $CDCl_3$) δ 155.3, 141.9, 136.8, 135.8, 133.4, 132.6, 131.1, 129.5, 127.7, 127.5, 125.9, 123.3, 55.8, 54.1, 52.3, 38.8, 34.1, 23.6, 23.6. **HR–MS** (ESI) *m/z* calc. for $C_{21}H_{24}NO_4S_2$ [M+H]$^+$: 418.1141, found: 418.1142.

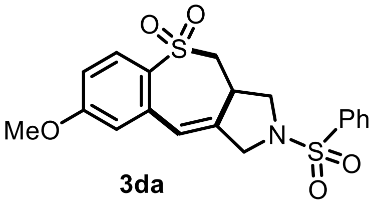

3da

8-Methoxy-2-(phenylsulfonyl)-2,3,3a,4-tetrahydro-1*H*-benzo[6,7]thiepino[3,4-*c*]pyrrole 5,5-dioxide (3da)

The general procedure (A) was followed using **1d** (116.4 mg, 0.60 mmol), **2a** (70.5 mg, 0.3 mmol) and Na_2CO_3 (31.8 mg, 0.30 mmol). Purification by column chromatography on silica gel (petroleum ether/ EtOAc = 3:1) yielded **3da** (79.0 mg, 65%) as a white solid. **M.p.**: 152–154 °C. **^{1}H NMR** (400 MHz, $CDCl_3$) δ 8.05 (d, J = 8.8 Hz, 1H), 7.87 (d, J = 7.4 Hz, 2H), 7.65 (d, J = 7.4 Hz, 1H), 7.60 (d, J = 7.8 Hz, 2H), 6.88 (dd, J = 8.8, 2.5 Hz, 1H), 6.77 (d, J = 2.5 Hz, 1H), 6.44 (d, J = 1.2 Hz, 1H), 4.20 (d, J = 14.7 Hz, 1H), 4.09 (d, J = 14.6 Hz, 1H), 3.86 (s, 3H), 3.71 (dd, J = 9.6, 7.2 Hz, 1H), 3.57 (d, J = 8.9 Hz, 1H), 3.40–3.34 (m, 2H), 3.12 (t, J = 8.8 Hz, 1H). **^{13}C NMR** (151 MHz, $CDCl_3$) δ 163.5, 142.6, 135.8, 135.0, 133.5, 131.3, 129.8, 129.5, 127.8, 123.1, 118.3, 112.2, 56.9, 55.8, 54.0, 52.3, 38.8. **HR–MS** (ESI) *m/z* calc. for $C_{19}H_{20}NO_5S_2$ [M+H]$^+$: 406.0778, found: 406.0777.

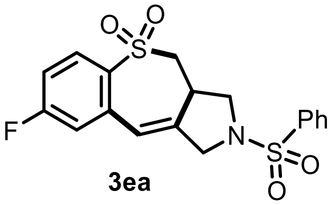

3ea

8-Fluoro-2-(phenylsulfonyl)-2,3,3a,4-tetrahydro-1*H*-benzo[6,7]thiepino[3,4-*c*]pyrrole 5,5-dioxide (3ea)

The general procedure (A) was followed using **1e** (109.2 mg, 0.60 mmol), **2a** (70.5 mg, 0.3 mmol) and Na_2CO_3 (31.8 mg, 0.30 mmol). Purification by column chromatography on silica gel (petroleum ether/ EtOAc = 3:1) yielded **3ea** (67.2 mg, 57%) as a white solid. **M.p.**: 160–162 °C. **^{1}H NMR** (400 MHz, $CDCl_3$) δ 8.15 (dd, J = 8.8, 5.6 Hz, 1H), 7.88 (d, J = 7.3 Hz, 2H), 7.67 (d, J = 7.4 Hz, 1H), 7.61 (d, J = 7.8 Hz, 2H), 7.14–7.09

(m, 1H), 7.00 (dd, *J* = 9.3, 2.5 Hz, 1H), 6.45 (d, *J* = 1.4 Hz, 1H), 4.23 (d, *J* = 14.9 Hz, 1H), 4.11 (d, *J* = 14.9 Hz, 1H), 3.74 (dd, *J* = 9.5, 7.3 Hz, 1H), 3.61 (d, *J* = 9.0 Hz, 1H), 3.45–3.37 (m, 2H), 3.12 (t, *J* = 8.6 Hz, 1H). ^{13}**C NMR** (101 MHz, $CDCl_3$) *δ* 165.5 (d, *J* = 256.5 Hz), 144.1, 136.0 (d, *J* = 9.1 Hz), 135.8, 133.6, 130.5 (d, *J* = 9.8 Hz), 129.6, 128.2, 127.8, 122.2, 119.3 (d, *J* = 23.2 Hz), 114.8 (d, *J* = 22.2 Hz), 56.4, 54.0, 52.2, 38.9. ^{19}**F NMR** (377 MHz, $CDCl_3$) *δ* –104.31. **HR–MS** (ESI) *m/z* calc. for $C_{18}H_{17}FNO_4S_2$ [M+H]$^+$: 354.0578, found: 354.0576.

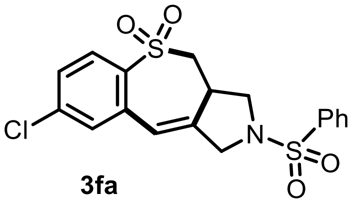

3fa

8-Chloro-2-(phenylsulfonyl)-2,3,3a,4-tetrahydro-1*H*-benzo[6,7]thiepino[3,4-*c*]pyrrole 5,5-dioxide (3fa)

The general procedure (A) was followed using **1f** (118.8 mg, 0.60 mmol), **2b** (70.5 mg, 0.3 mmol) and Na_2CO_3 (31.8 mg, 0.30 mmol). Purification by column chromatography on silica gel (petroleum ether/ EtOAc = 3:1) yielded **3fa** (62.6 mg, 51%) as a yellow solid. **M.p.**: 182–184 °C. ^{1}**H NMR** (600 MHz, $CDCl_3$) *δ* 8.07 (d, *J* = 8.5 Hz, 1H), 7.88 (d, *J* = 7.3 Hz, 2H), 7.67 (d, *J* = 7.5 Hz, 1H), 7.61 (d, *J* = 7.9 Hz, 2H), 7.40 (dd, *J* = 8.5, 2.0 Hz, 1H), 7.29 (d, *J* = 1.9 Hz, 1H), 6.43 (d, *J* = 1.7 Hz, 1H), 4.24 (d, *J* = 14.8 Hz, 1H), 4.10 (d, *J* = 14.8 Hz, 1H), 3.75 (dd, *J* = 9.5, 7.6 Hz, 1H), 3.59 (dd, *J* = 13.5, 4.2 Hz, 1H), 3.47–3.39 (m, 2H), 3.13 (t, *J* = 9.0 Hz, 1H). ^{13}**C NMR** (101 MHz, $CDCl_3$) *δ* 144.2, 140.2, 137.8, 135.8, 134.5, 133.6, 132.4, 129.6, 129.0, 127.8, 122.1, 56.1, 54.1, 52.2, 38.9. **HR–MS** (ESI) *m/z* calc. for $C_{18}H_{17}ClNO_4S_2$ [M+H]$^+$: 410.0282, found: 410.0264.

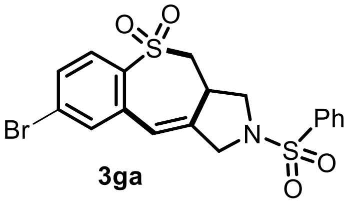

3ga

8-Bromo-2-(phenylsulfonyl)-2,3,3a,4-tetrahydro-1*H*-benzo[6,7]thiepino[3,4-*c*]pyrrole 5,5-dioxide (3ga)

The general procedure (A) was followed using **1g** (145.2 mg, 0.60 mmol), **2b** (70.5 mg, 0.3 mmol) and Na_2CO_3 (31.8 mg, 0.30 mmol). Purification by column chromatography on silica gel (petroleum ether/ EtOAc = 3:1) yielded **3ga** (88.3 mg, 65%) as a yellow

solid. **M.p.**: 212–214 °C. **^{1}H NMR** (400 MHz, $CDCl_3$) δ 7.99 (d, *J* = 8.4 Hz, 1H), 7.88 (d, *J* = 7.5 Hz, 2H), 7.67 (d, *J* = 7.1 Hz, 1H), 7.60 (dd, *J* = 7.5, 7.5 Hz, 2H), 7.56 (d, *J* = 8.5 Hz, 1H), 7.46 (s, 1H), 6.42 (s, 1H), 4.24 (d, *J* = 14.9 Hz, 1H), 4.10 (d, *J* = 14.9 Hz, 1H), 3.75 (dd, *J* = 9.2, 7.5 Hz, 1H), 3.59 (dd, *J* = 12.9, 3.7 Hz, 1H), 3.46–3.34 (m, 2H), 3.12 (t, *J* = 8.9 Hz, 1H). **^{13}C NMR** (151 MHz, $CDCl_3$) δ 144.2, 138.2, 135.8, 135.3, 134.5, 133.6, 130.8, 129.56, 129.0, 128.6, 127.8, 122.0, 56.0, 54.1, 52.2, 38.9. **HR–MS** (ESI) *m/z* calc. for $C_{18}H_{17}BrNO_4S_2$ $[M+H]^+$: 453.9777, found: 453.9774.

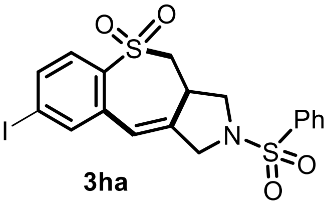

3ha

8-Iodo-2-(phenylsulfonyl)-2,3,3a,4-tetrahydro-1*H*-benzo[6,7]thiepino[3,4-*c*]pyrrole 5,5-dioxide (3ha)

The general procedure (A) was followed using **1h** (174.0 mg, 0.60 mmol), **2b** (70.5 mg, 0.3 mmol) and Na_2CO_3 (31.8 mg, 0.30 mmol). Purification by column chromatography on silica gel (petroleum ether/ EtOAc = 3:1) yielded **3ha** (61.6 mg, 41%) as a yellow solid. **M.p.**: 176–178 °C. **^{1}H NMR** (400 MHz, $CDCl_3$) δ 7.89–7.86 (m, 2H), 7.81 (dd, *J* = 8.4, 8.4 Hz, 2H), 7.67 (d, *J* = 6.9 Hz, 2H), 7.61 (dd, *J* = 8.4, 1.6 Hz, 2H), 7.70–7.66 (m, 2H), 7.62–7.58 (m, 2H), 6.39 (d, *J* = 2.0 Hz, 1H), 4.24 (dt, *J* = 14.8, 1.6 Hz, 1H). 4.09 (dt, *J* = 14.8, 1.6 Hz, 1H), 3.75 (dd, *J* = 9.5, 7.4 Hz, 1H), 3.58 (dd, *J* = 13.1, 3.9 Hz, 1H), 3.47–3.34 (m, 2H), 3.11 (t, *J* = =9.6 Hz, 1H). **^{13}C NMR** (151 MHz, $CDCl_3$) δ 144.0, 141.3, 139.0, 138.9, 136.8, 134.1, 133.6, 129.6, 128.6, 127.8, 121.9, 101.2, 56.0, 54.1, 52.2, 38.9. **HR–MS** (ESI) *m/z* calc. for $C_{18}H_{17}INO_4S_2$ $[M+H]^+$: 501.9638, found: 501.9633.

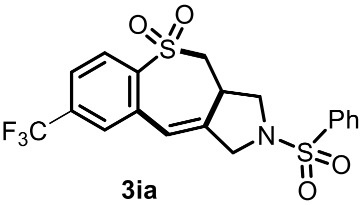

3ia

2-(Phenylsulfonyl)-8-(trifluoromethyl)-2,3,3a,4-tetrahydro-1*H*-benzo[6,7]thiepino[3,4-*c*]pyrrole 5,5-dioxide (3ia)

The general procedure (A) was followed using **1i** (139.2 mg, 0.60 mmol), **2b** (70.5 mg, 0.3 mmol) and Na_2CO_3 (31.8 mg, 0.30 mmol). Purification by column chromatography on silica gel (petroleum ether/ EtOAc = 3:1) yielded **3ia** (81.1 mg, 61%) as a white

solid. **M.p.**: 198–200 °C. **^{1}H NMR** (400 MHz, $CDCl_3$) *δ* 8.27 (d, *J* = 8.2 Hz, 1H), 7.88 (d, *J* = 7.4 Hz, 2H), 7.68 (dd, *J* = 7.7, 5.4 Hz, 2H), 7.61 (dd, *J* = 7.5, 7.5 Hz, 2H), 7.56 (s, 1H), 6.55 (d, *J* = 1.5 Hz, 1H), 4.27 (d, *J* = 15.0 Hz, 1H), 4.12 (d, *J* = 15.0 Hz, 1H), 3.78 (dd, *J* = 9.4, 7.4 Hz, 1H), 3.63 (dd, *J* = 12.9, 3.5 Hz, 1H), 3.49–3.38(m, 2H), 3.13 (t, *J* = 9.0 Hz, 1H). **^{13}C NMR** (151 MHz, $CDCl_3$) *δ* 144.7, 142.5, 135.8, 135.5, 133.7, 133.6, 129.6, 129.5, 128.3, 127.8, 124.5 (q, *J* = 3.0 Hz), 122.2, 55.7, 54.1, 52.2, 38.9. **^{19}F NMR** (377 MHz, $CDCl_3$) *δ* –63.45. **HR–MS** (ESI) *m/z* calc. for $C_{19}H_{17}F_3NO_4S_2$ $[M+H]^+$: 444.0546, found: 444.0543.

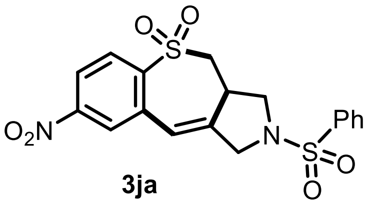

3ja

8-Nitro-2-(phenylsulfonyl)-2,3,3a,4-tetrahydro-1*H*-benzo[6,7]thiepino[3,4-*c*]pyrrole 5,5-dioxide (3ja)

The general procedure (A) was followed using **1j** (125.4 mg, 0.60 mmol), **2b** (70.5 mg, 0.3 mmol) and Na_2CO_3 (31.8 mg, 0.30 mmol). Purification by column chromatography on silica gel (petroleum ether/ EtOAc = 3:1) yielded **3ja** (50.4 mg, 40%) as a white solid. **M.p.**: 231–233 °C. **^{1}H NMR** (400 MHz, DMSO) *δ* 8.33 (d, *J* = 2.0 Hz, 1H), 8.26 (dd, *J* = 8.7, 2.0 Hz, 1H), 8.21 (d, *J* = 8.7 Hz, 1H), 7.87 (d, *J* = 7.5 Hz, 2H), 7.75 (dd, *J* = 7.3, 7.3 Hz, 1H), 7.67 (dd, *J* = 7.5, 7.5 Hz, 2H), 6.85 (s, 1H), 4.30 (d, *J* = 15.4 Hz, 1H), 3.99 (d, *J* = 15.4 Hz, 1H), 3.91–3.83 (m, 2H), 3.76 (dd, *J* = 14.8, 12.1 Hz, 1H), 3.29–3.21 (m, 1H), 3.00 (t, *J* = 10.0 Hz, 1H). **^{13}C NMR** (151 MHz, DMSO) *δ* 150.7, 146.0, 144.8, 135.8, 134.2, 134.0, 130.1, 128.5, 128.1, 128.0, 122.5, 121.4, 54.5, 52.1, 51.5, 39.3. **HR–MS** (ESI) *m/z* calc. for $C_{18}H_{17}N_2O_6S_2$ $[M+H]^+$: 421.0523, found: 421.0515.

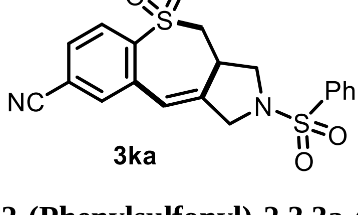

3ka

2-(Phenylsulfonyl)-2,3,3a,4-tetrahydro-1*H*-benzo[6,7]thiepino[3,4-*c*]pyrrole-8-carbonitrile 5,5-dioxide (3ka)

The general procedure (A) was followed using **1k** (113.4 mg, 0.60 mmol), **2b** (70.5 mg, 0.3 mmol) and Na_2CO_3 (31.8 mg, 0.30 mmol). Purification by column chromatography

on silica gel (petroleum ether/ EtOAc = 3:1) yielded **3ka** (54.0 mg, 45%) as a white solid. **M.p.**: 215–217 °C. **^{1}H NMR** (600 MHz, $CDCl_3$) δ 8.26 (d, J = 8.1 Hz, 1H), 7.88 (dd, J = 8.4, 1.2 Hz, 2H), 7.71 (dd, J = 8.2, 1.5 Hz, 1H), 7.68 (dd, J = 2.5, 1.6 Hz, 1H), 7.63–7.59 (m, 3H), 6.49 (d, J = 2.0 Hz, 1H), 4.27 (dt, J = 15.0, 1.2 Hz, 1H), 4.13 (dt, J = 15.1, 1.6 Hz, 1H), 3.77 (dd, J = 9.5, 7.5 Hz, 1H), 3.64 (dd, J = 13.6, 4.1 Hz, 1H), 3.49–3.40 (m, 2H), 3.14 (t, J = 9.0 Hz, 1H). **^{13}C NMR** (151 MHz, DMSO) δ 145.9, 143.6, 137.2, 135.7, 134.0, 133.4, 131.4, 130.1, 128.0, 127.4, 121.1, 117.7, 116.6, 54.6, 52.2, 51.6, 39.3. **HR–MS** (ESI) *m/z* calc. for $C_{19}H_{17}N_2O_4S_2$ $[M+H]^+$: 401.0624, found: 401.0601.

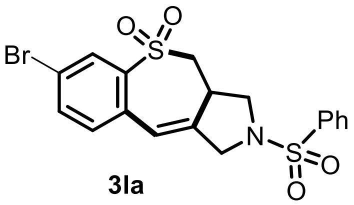

3la

7-Bromo-2-(phenylsulfonyl)-2,3,3a,4-tetrahydro-1*H*-benzo[6,7]thiepino[3,4-*c*]pyrrole 5,5-dioxide (3la)

The general procedure (A) was followed using **1l** (145.2 mg, 0.60 mmol), **2b** (70.5 mg, 0.3 mmol) and Na_2CO_3 (31.8 mg, 0.30 mmol). Purification by column chromatography on silica gel (petroleum ether/ EtOAc = 3:1) yielded **3la** (58.4 mg, 43%) as a white solid. **M.p.**: 62–64 °C. **^{1}H NMR** (600 MHz, $CDCl_3$) δ 8.09 (dd, J = 7.9, 0.9 Hz, 1H), 7.88 (dd, J = 8.3, 1.1 Hz, 2H), 7.84 (dd, J = 8.0, 1.1 Hz, 1H), 7.71–7.68 (m, 1H), 7.62 (dd, J = 7.7, 7.7 Hz, 2H), 7.30 (dd, J = 7.8, 7.8 Hz, 1H), 6.73 (d, J = 2.0 Hz, 1H), 4.37 (dt, J = 15.0, 1.8 Hz, 1H), 3.95 (dd, J = 15.4, 2.4 Hz, 1H), 3.92 (dd, J = 13.8, 6.6 Hz, 1H), 3.78 (dd, J = 13.8, 11.4 Hz, 1H), 3.30 (d, J = 1.5 Hz, 1H), 3.29 (s, 1H), 3.25–3.21 (m, 1H). **^{13}C NMR** (101 MHz, $CDCl_3$) δ 143.7, 140.5, 138. 2, 135.8, 135.1, 133.6, 129.6, 128.7, 128.0, 127.7, 125.3, 123.1, 66.5, 52.4, 52.3, 37.1. **HR–MS** (ESI) *m/z* calc. for $C_{18}H_{17}BrNO_4S_2$ $[M+H]^+$: 453.9777, found: 453.9782.

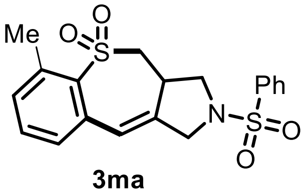

3ma

6-Methyl-2-(phenylsulfonyl)-2,3,3a,4-tetrahydro-1*H*-benzo[6,7]thiepino[3,4-*c*]pyrrole 5,5-dioxide (3ma)

The general procedure (A) was followed using **1m** (106.8 mg, 0.60 mmol), **2b** (70.5

mg, 0.3 mmol) and Na_2CO_3 (31.8 mg, 0.30 mmol). Purification by column chromatography on silica gel (petroleum ether/ EtOAc = 3:1) yielded **3ma** (70.0 mg, 60%) as a pink solid. **M.p.**: 149–151 °C. **^{1}H NMR** (400 MHz, $CDCl_3$) δ 8.01 (d, *J* = 8.1 Hz, 1H), 7.88 (d, *J* = 7.9 Hz, 2H), 7.66 (d, *J* = 6.7 Hz, 1H), 7.61 (d, *J* = 7.7 Hz, 2H), 7.23 (d, *J* = 8.1 Hz, 1H), 7.10 (s, 1H), 6.44 (s, 1H), 4.24 (d, *J* = 14.5 Hz, 1H), 4.08 (d, *J* = 14.4 Hz, 1H), 3.77 (dd, J = 9.2, 7.6 Hz, 1H), 3.55 (dd, *J* = 13.2, 4.2 Hz, 1H), 3.45–3.32 (m, 2H), 3.11 (t, *J* = 9.1 Hz, 1H), 2.41 (s, 3H). **^{13}C NMR** (151 MHz, $CDCl_3$) δ 144.7, 142.1, 136.6, 133.5, 133.5, 132.5, 129.5, 128.4, 127.9, 127.8, 127.5, 123.2, 55.8, 54.2, 52.3, 38.9, 21.5. **HR–MS** (ESI) *m/z* calc. for $C_{19}H_{20}NO_4S_2$ $[M+H]^+$: 390.0828, found: 390.0836.

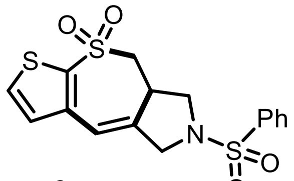

3na

6-(Phenylsulfonyl)-6,7,7a,8-tetrahydro-5*H*-thieno[3',2':6,7]thiepino[3,4-*c*]pyrrole 9,9-dioxide (3na)

The general procedure (A) was followed using **1n** (102.0 mg, 0.60 mmol), **2b** (70.5 mg, 0.3 mmol) and Na_2CO_3 (31.8 mg, 0.30 mmol). Purification by column chromatography on silica gel (petroleum ether/ EtOAc = 3:1) yielded **3na** (44.6 mg, 39%) as a yellow solid. **M.p.**: 180–182 °C. **^{1}H NMR** (400 MHz, $CDCl_3$) δ 7.88 (d, *J* = 7.3 Hz, 2H), 7.67 (d, *J* = 7.3 Hz, 1H), 7.61 (d, *J* = 7.8 Hz, 2H), 7.52 (d, *J* = 5.0 Hz, 1H), 6.95 (d, *J* = 5.0 Hz, 1H), 6.50 (d, *J* = 2.1 Hz, 1H), 4.33 (dt, *J* = 15.0, 1.7 Hz, 1H), 3.99 (dt, *J* = 15.0, 2.0 Hz, 1H), 3.95(t, *J* = 9.2 Hz, 1H), 3.60–3.55 (m, 1H), 3.40 (dd, *J* = 14.4, 3.8 Hz, 1H), 3.30 (t, *J* = 12.8 Hz, 1H), 3.03 (t, *J* = 10.4 Hz, 1H). **^{13}C NMR** (151 MHz, $CDCl_3$) δ 142.3, 137.9, 136.2, 135.9, 133.6, 131.6, 129.8, 129.6, 127.8, 116.8, 54.2, 52.6, 52.1, 39.2. **HR–MS** (ESI) *m/z* calc. for $C_{16}H_{16}NO_4S_3$ $[M+H]^+$: 382.0236, found: 382.0230.

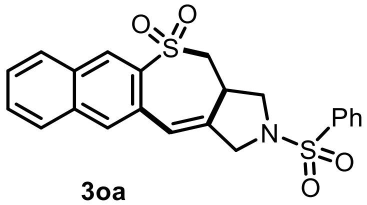

3oa

2-(Phenylsulfonyl)-2,3,3a,4-tetrahydro-1*H*-naphtho[2',3':6,7]thiepino[3,4-*c*]pyrrole 5,5-dioxide (3oa)

The general procedure (A) was followed using **1o** (128.4 mg, 0.60 mmol), **2b** (70.5 mg,

0.3 mmol) and Na_2CO_3 (31.8 mg, 0.30 mmol). Purification by column chromatography on silica gel (petroleum ether/ EtOAc = 3:1) yielded **3oa** (81.6 mg, 64%) as a yellow solid. **M.p.**: 93–95 °C. **^{1}H NMR** (600 MHz, $CDCl_3$) δ 8.15 (d, J = 8.7 Hz, 1H), 8.00 (d, J = 8.4 Hz, 1H), 7.93 (dd, J = 6,0, 6.0 Hz, 2H), 7.90 (d, J = 7.6 Hz, 2H), 7.68 (dd, J = 14.5, 7.3 Hz, 2H), 7.62 (d, J = 7.4 Hz, 3H), 7.21 (s, 1H), 4.48 (d, J = 14.9 Hz, 1H), 4.03 (dd, J = 13.8, 6.3 Hz, 2H), 3.86 (dd, J = 7.6, 7.6 Hz, 1H), 3.31–3.25 (m, 3H). **^{13}C NMR** (151 MHz, $CDCl_3$) δ 144.6, 136.1, 135.5, 135.2, 133.6, 131.2, 129.5, 129.1, 128.8, 128.4, 128.0, 127.9, 127.4, 125.3, 122.9, 120.4, 68.9, 52.4, 52.3, 37.3. **HR–MS** (ESI) *m/z* calc. for $C_{22}H_{20}NO_4S_2$ $[M+H]^+$: 426.0828, found: 426.0831.

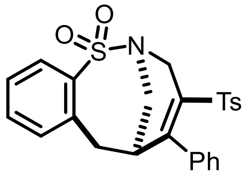

4a

5-Phenyl-4-tosyl-6,7-dihydro-3*H*-2,6-methanobenzo[*h*][1,2]thiazonine 1,1-dioxide (4a)

The general procedure (B) was followed using **1a** (107 mg, 0.60 mmol), **2a'** (93.3 mg, 0.3 mmol). Purification by column chromatography on silica gel (petroleum ether/ EtOAc = 3:1) yielded **4a** (39.1 mg, 28%) as a yellow solid. **M.p.**: 108–110 °C. **^{1}H NMR** (600 MHz, $CDCl_3$) δ 8.00 (dd, J = 7.7, 1.1 Hz, 1H), 7.49–7.46 (m, 1H), 7.38 (dd, J = 7.6, 7.6 Hz, 1H), 7.32 (dd, J = 7.4, 7.4 Hz, 1H), 7.23 (dd, J = 7.8, 7.8 Hz, 2H), 7.03 (s, 4H), 7.00 (d, J = 7.5 Hz, 1H), 6.68 (s, 2H), 4.47 (s, 1H), 4.44 (d, J = 5.5 Hz, 1H), 4.29 (dd, J = 19.7, 2.0 Hz, 1H), 3.61 (d, J = 14.9 Hz, 1H), 3.48 (d, J = 15.4 Hz, 1H), 2.91–2.85 (m, 2H), 2.37 (s, 3H). **^{13}C NMR** (151 MHz, $CDCl_3$) δ 149.6, 144.1, 139.7, 137.5, 136.7, 135.2, 134.8, 133.5, 132.7, 129.7, 129.4, 128.6, 127.8, 127.4, 127.3, 50.0, 45.6, 38.9, 38.6, 21.6. **HR–MS** (ESI) *m/z* calc. for $C_{25}H_{24}NO_4S_2$ $[M+H]^+$: 466.1141, found: 466.1144.

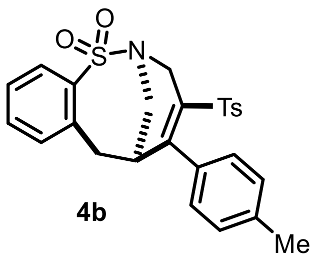

4b

5-(*p*-Tolyl)-4-tosyl-6,7-dihydro-3*H*-2,6-methanobenzo[*h*][1,2]thiazonine 1,1-dioxide (4b)

The general procedure (B) was followed using **1a** (107 mg, 0.60 mmol), **2b'** (97.5 mg, 0.3 mmol). Purification by column chromatography on silica gel (petroleum ether/ EtOAc = 3:1) yielded **4b** (34.5 mg, 24%) as a yellow solid. **M.p.**: 193–195 °C. **^{1}H NMR** (600 MHz, $CDCl_3$) δ 7.99 (d, *J* = 7.7 Hz, 1H), 7.47–7.44 (m, 1H), 7.37 (dd, *J* = 7.6, 7.6 Hz, 1H), 7.09 (d, *J* = 8.3 Hz, 2H), 7.05 (d, *J* = 7.9 Hz, 4H), 6.98 (d, *J* = 7.5 Hz, 1H), 6.59 (s, 2H), 4.45 (d, *J* = 14.4 Hz, 1H), 4.42 (d, *J* = 16.8 Hz, 1H), 4.26 (dd, *J* = 19.5, 1.9 Hz, 1H), 3.61 (d, *J* = 14.7 Hz, 1H), 3.46 (dd, *J* = 14.4, 1.2 Hz, 1H), 2.91–2.85 (m, 2H), 2.39 (s, 3H), 2.38 (s, 3H). **^{13}C NMR** (151 MHz, $CDCl_3$) δ 149.8, 144.1, 139.8, 138.7, 137.7, 135.2, 134.5, 133.9, 133.5, 132.7, 129.7, 129.3, 128.5, 127.4, 127.4, 50.1, 45.8, 39.0, 38.7, 21.7, 21.4. **HR–MS** (ESI) *m/z* calc. for $C_{26}H_{26}NO_4S_2$ $[M+H]^+$: 480.1298, found: 480.1303.

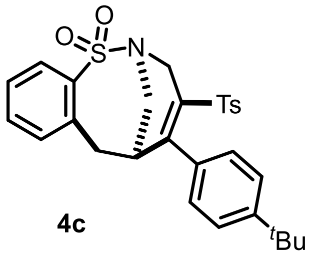

4c

5-(4-(Tert-butyl)phenyl)-4-tosyl-6,7-dihydro-3*H*-2,6-methanobenzo[*h*][1,2]thiazonine 1,1-dioxide (4c)

The general procedure (B) was followed using **1a** (107 mg, 0.60 mmol), **2c'** (110.1 mg, 0.3 mmol). Purification by column chromatography on silica gel (petroleum ether/ EtOAc = 3:1) yielded **4c** (34.4 mg, 22%) as a white solid. **M.p.**: 221–223 °C. **^{1}H NMR** (400 MHz, $CDCl_3$) δ 8.05 (dd, *J* = 7.7, 1.3 Hz, 1H), 7.52–7.48 (m, 1H), 7.42 (dd, *J* = 7.5, 7.5 Hz, 1H), 7.19 (d, *J* = 8.6 Hz, 2H), 7.02 (d, *J* = 7.4 Hz, 1H), 6.97 (s, 4H), 6.57 (d, *J* = 8.2 Hz, 2H), 4.51 (d, *J* = 7.8 Hz, 1H), 4.47 (s, 1H), 4.32 (dd, *J* = 19.6, 2.2 Hz, 1H), 3.63 (d, *J* = 15.4 Hz, 1H), 3.49 (dd, *J* = 14.4, 1.7 Hz, 1H), 2.92 (dd, *J* = 14.9, 6.5 Hz, 1H), 2.83 (d, *J* = 5.8 Hz, 1H), 2.35 (s, 3H), 1.34 (s, 9H). **^{13}C NMR** (101 MHz, $CDCl_3$) δ 151.8, 149.6, 143.7, 139.8, 137.7, 135.3, 135.1, 133.6, 133.5, 132.8, 129.9, 129.2, 128.0, 127.5, 127.3, 124.7, 50.1, 45.6, 39.0, 38.5, 34.7, 31.4, 21.6. **HR–MS** (ESI) *m/z* calc. for $C_{29}H_{32}NO_4S_2$ $[M+H]^+$: 522.1767, found: 522.1771.

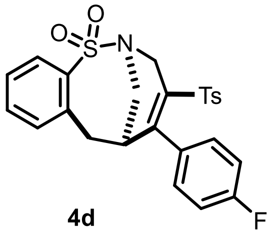

4d

5-(4-Fluorophenyl)-4-tosyl-6,7-dihydro-3*H*-2,6-methanobenzo[*h*][1,2]thiazonine 1,1-dioxide (4d)

The general procedure (B) was followed using **1a** (107 mg, 0.60 mmol), **2d'** (98.7 mg, 0.3 mmol). Purification by column chromatography on silica gel (petroleum ether/ EtOAc = 3:1) yielded **4d** (40.6 mg, 28%) as a yellow solid. **M.p.**: 180–182 °C. **^{1}H NMR** (400 MHz, $CDCl_3$) δ 8.02 (dd, J = 7.7, 1.2 Hz, 1H), 7.50–7.46 (m, 1H), 7.40 (dd, J = 7.6, 7.6 Hz, 1H), 7.08 (s, 4H), 6.98–6.91 (m, 3H), 6.68 (s, 2H), 4.48 (d, J = 7.7 Hz, 1H), 4.44 (d, J = 12.8 Hz, 1H), 4.26 (dd, J = 19.7, 2.0 Hz, 1H), 3.65 (d, J = 14.6 Hz, 1H), 3.47 (dd, J = 14.5, 1.2 Hz, 1H), 2.90–2.82 (m, 2H), 2.40 (s, 3H). **^{13}C NMR** (151 MHz, $CDCl_3$) δ 162.9 (d, J = 249.2 Hz), 148.5, 144.4, 139.9, 137.5, 135.7, 135.0, 133.3, 132.8, 132.6, 129.9, 129.5, 127.6, 127.3, 115.0 (d, J= 25.7 Hz), 50.0, 45.8, 39.0, 38.7, 21.7. **^{19}F NMR** (377 MHz, $CDCl_3$) δ –112.08. **HR–MS** (ESI) *m/z* calc. for $C_{25}H_{23}FNO_4S_2$ $[M+H]^+$: 484.1047, found: 484.1045.

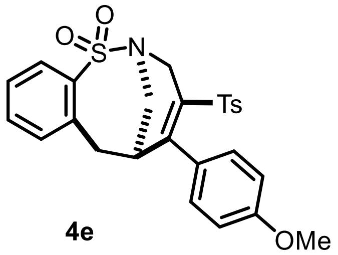

4e

5-(4-Methoxyphenyl)-4-tosyl-6,7-dihydro-3*H*-2,6-methanobenzo[*h*][1,2]thiazonine 1,1-dioxide (4e)

The general procedure (B) was followed using **1a** (107 mg, 0.60 mmol), **2e'** (102.3 mg, 0.3 mmol). Purification by column chromatography on silica gel (petroleum ether/ EtOAc = 3:1) yielded **4e** (81.7 mg, 55%) as a white solid. **M.p.**: 128–130 °C. **^{1}H NMR** (400 MHz, $CDCl_3$) δ 8.02 (dd, J = 7.7, 1.4 Hz, 1H), 7.48–7.44 (m, 1H), 7.39 (dd, J = 7.2, 7.2 Hz, 1H), 7.07 (dd, J = 8.5, 8.5 Hz, 4H), 6.95 (d, J = 7.3 Hz, 1H), 6.76 (d, J = 8.9 Hz, 2H), 6.61 (d, J = 8.3 Hz, 2H), 4.48–4.42 (m, 2H), 4.25 (dd, J = 19.5, 1.7 Hz, 1H), 3.85 (s, 3H), 3.62 (q, J = 5.0 Hz, 1H), 3.45 (dt, J = 14.0, 2.0 Hz, 1H), 2.90–2.85 (m, 2H), 2.38 (s, 3H). **^{13}C NMR** (151 MHz, $CDCl_3$) δ 160.0, 149.6, 144.0, 139.9, 137.8, 135.2, 134.8, 133.5, 132.7, 129.8, 129.4, 128.7, 127.5, 127.4, 113.3, 55.4, 50.1, 45.9, 39.1, 38.7, 21.7. **HR–MS** (ESI) *m/z* calc. for $C_{26}H_{26}NO_5S_2$ $[M+H]^+$: 496.1247, found: 496.1266.

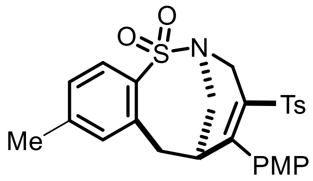

4f

5-(4-Methoxyphenyl)-9-methyl-4-tosyl-6,7-dihydro-3*H*-2,6-methanobenzo[*h*][1,2]thiazonine 1,1-dioxide (4f)

The general procedure (B) was followed using **1a** (107 mg, 0.60 mmol), **2f'** (107.1 mg, 0.3 mmol). Purification by column chromatography on silica gel (petroleum ether/ EtOAc = 3:1) yielded **4f** (76.4 mg, 50%) as a white solid. **M.p.**: 212–214 °C. **^{1}H NMR** (600 MHz, $CDCl_3$) δ 7.89 (d, *J* = 7.9 Hz, 1H), 7.16 (d, *J* = 7.8 Hz, 1H), 7.10 (d, *J* = 8.4 Hz, 2H), 7.07 (d, *J* = 8.3 Hz, 2H), 6.76 (d, *J* = 8.9 Hz, 2H), 6.70 (s, 1H), 6.60 (d, *J* = 4.8 Hz, 2H), 4.45 (s, 1H), 4.42 (d, *J* = 4.9 Hz, 1H), 4.23 (dd, *J* = 19.7, 2.2 Hz, 1H), 3.85 (s, 3H), 3.58 (d, *J* = 15.1 Hz, 1H), 3.43 (dd, *J* = 14.1, 1.7 Hz, 1H), 2.83 (d, *J* = 6.5 Hz, 1H), 2.77 (dd, *J* = 14.7, 6.5 Hz, 1H), 2.39 (s, 3H), 2.37 (s, 3H). **^{13}C NMR** (151 MHz, $CDCl_3$) δ 160.0, 149.6, 144.0, 143.2, 138.0, 136.9, 135.0, 134.8, 134.4, 131.5, 129.8, 129.3, 128.7, 127.8, 127.4, 113.2, 55.4, 50.1, 45.9, 39.0, 38.6, 21.6, 21.4. **HR–MS** (ESI) *m/z* calc. for $C_{27}H_{28}NO_5S_2$ $[M+H]^+$: 510.1404, found: 510.1413.

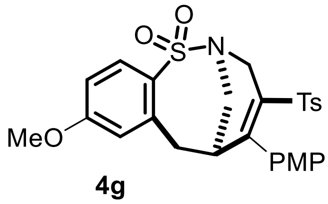

4g

9-Methoxy-5-(4-methoxyphenyl)-4-tosyl-6,7-dihydro-3*H*-2,6-methanobenzo[*h*][1,2]thiazonine 1,1-dioxide (4g)

The general procedure (B) was followed using **1a** (107 mg, 0.60 mmol), **2g'** (111.3 mg, 0.3 mmol). Purification by column chromatography on silica gel (petroleum ether/ EtOAc = 3:1) yielded **4g** (75.6 mg, 48%) as a yellow solid. **M.p.**: 95–97 °C. **^{1}H NMR** (400 MHz, $CDCl_3$) δ 7.95 (d, *J* = 8.7 Hz, 1H), 7.12–7.06 (m, 4H), 6.83 (dd, *J* = 8.7, 2.4 Hz, 1H), 6.77 (d, *J* = 8.8 Hz, 2H), 6.66 (d, *J* = 8.5 Hz, 2H), 6.43 (d, *J* = 2.4 Hz, 1H), 4.43 (d, *J* = 18.0 Hz, 2H), 4.22 (dd, *J* = 19.7, 1.9 Hz, 1H), 3.86 (s, 3H), 3.85 (s, 3H), 3.59 (d, *J* = 14.4 Hz, 1H), 3.42 (d, *J* = 13.0 Hz, 1H), 2.83–2.75 (m, 2H), 2.39 (s, 3H). **^{13}C NMR** (151 MHz, $CDCl_3$) δ 162.6, 160.1, 149.5, 144.0, 138.0, 137.3, 135.0, 132.0, 131.5, 129.3, 128.8, 127.4, 119.7, 114.9, 113.3, 111.3, 55.7, 55.5, 50.1, 45.9, 39.2, 38.8, 21.7. **HR–MS** (ESI) *m/z* calc. for $C_{27}H_{28}NO_6S_2$ $[M+H]^+$: 526.1353, found: 526.1352.

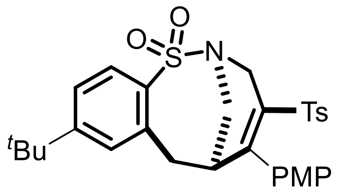

4h

9-(Tert-butyl)-5-(4-methoxyphenyl)-4-tosyl-6,7-dihydro-3*H*-2,6-methanobenzo[*h*][1,2]thiazonine 1,1-dioxide (4h)

The general procedure (B) was followed using **1a** (107 mg, 0.60 mmol), **2h'** (119.1 mg, 0.3 mmol). Purification by column chromatography on silica gel (petroleum ether/ EtOAc = 3:1) yielded **4h** (106.3 mg, 61%) as a yellow solid. **M.p.**: 83–85 °C. ^{1}**H NMR** (400 MHz, $CDCl_3$) δ 7.90 (d, *J* = 8.2 Hz, 1H), 7.31 (dd, *J* = 8.2, 1.7 Hz, 1H), 7.13 (d, *J* = 8.3 Hz, 2H), 7.08 (d, *J* = 8.3 Hz, 2H), 6.89 (d, *J* = 1.2 Hz, 1H), 6.73 (d, *J* = 7.7 Hz, 2H), 6.56 (s, 2H), 4.46 (d, *J* = 11.5 Hz, 1H), 4.43 (s, 1H), 4.30 (dd, *J* = 19.6, 2.1 Hz, 1H), 3.83 (s, 3H), 3.59 (d, *J* = 15.1 Hz, 1H), 3.46 (dd, *J* = 14.3, 2.3 Hz, 1H), 2.89 (d, *J* = 6.5 Hz, 1H), 2.80 (dd, *J* = 15.1, 6.8 Hz, 1H), 2.39 (s, 3H), 1.31 (s, 9H). ^{13}**C NMR** (151 MHz, $CDCl_3$) δ 160.0, 156.0, 150.1, 144.0, 138.2, 136.8, 134.6, 134.6, 131.2, 129.6, 129.4, 128.8, 127.3, 123.9, 113.2, 55.4, 50.1, 46.1, 39.5, 38.8, 35.0, 31.0, 21.7. **HR–MS** (ESI) *m/z* calc. for $C_{30}H_{34}NO_5S_2$ $[M+H]^+$: 552.1873, found: 582.1874.

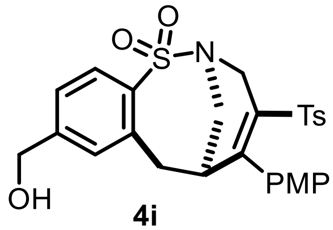

4i

9-(Hydroxymethyl)-5-(4-methoxyphenyl)-4-tosyl-6,7-dihydro-3*H*-2,6-methanobenzo[*h*][1,2]thiazonine 1,1-dioxide (4i)

The general procedure (B) was followed using **1a** (107 mg, 0.60 mmol), **2p'** (111.3 mg, 0.3 mmol). Purification by column chromatography on silica gel (petroleum ether/ EtOAc = 1:2) yielded **4i** (74.0 mg, 47%) as a white solid. **M.p.**: 58–60 °C. ^{1}**H NMR** (400 MHz, $CDCl_3$) δ 7.95 (d, *J* = 8.0 Hz, 1H), 7.32 (d, *J* = 7.9 Hz, 1H), 7.10–7.04 (m, 4H), 6.92 (s, 1H), 6.74 (d, *J* = 8.3 Hz, 2H), 6.58 (d, *J* = 7.9 Hz, 2H), 4.70 (s, 2H), 4.47–4.37 (m, 2H), 4.22 (d, *J* = 19.5 Hz, 1H), 3.83 (s, 3H), 3.59 (d, *J* = 13.7 Hz, 1H), 3.43 (d, *J* = 14.2 Hz, 1H), 2.88–2.77 (m, 2H), 2.37 (s, 3H). ^{13}**C NMR** (151 MHz, $CDCl_3$) δ 160.1, 149.7, 146.1, 144.2, 138.4, 137.6, 135.4, 134.6, 131.8, 129.9, 129.3, 128.5, 127.6, 127.4, 125.2, 113.3, 64.1, 55.5, 50.1, 45.8, 39.1, 38.5, 21.7. **HR–MS** (ESI) *m/z* calc. for

$C_{27}H_{28}NO_6S_2$ $[M+H]^+$: 526.1353, found: 526.1349.

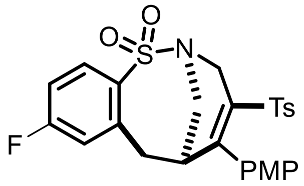

4j

9-Fluoro-5-(4-methoxyphenyl)-4-tosyl-6,7-dihydro-3*H*-2,6-methanobenzo[*h*][1,2]thiazonine 1,1-dioxide (4j)

The general procedure (B) was followed using **1a** (107 mg, 0.60 mmol), **2i'** (107.7 mg, 0.3 mmol). Purification by column chromatography on silica gel (petroleum ether/ EtOAc = 3:1) yielded **4j** (61.6 mg, 40%) as a yellow oil. **^{1}H NMR** (400 MHz, $CDCl_3$) δ 8.03 (dd, J = 8.6, 5.7 Hz, 1H), 7.31 (d, J = 8.1 Hz, 1H), 7.18 (dd, J = 6.8, 6.8 Hz, 1H), 7.09 (dd, J = 18.5, 8.3 Hz, 4H), 6.77 (d, J = 8.6 Hz, 2H), 6.63 (d, J = 8.2 Hz, 2H), 4.46 (d, J = 6.8 Hz, 1H), 4.41 (s, 1H), 4.25 (dd, J = 19.6, 1.6 Hz, 1H), 3.84 (s, 3H), 3.59 (d, J = 14.5 Hz, 1H), 3.44 (d, J = 14.8 Hz, 1H), 2.85–2.77 (m, 2H), 2.38 (s, 3H). **^{13}C NMR** (151 MHz, $CDCl_3$) δ 164.5 (d, J = 255.2 Hz), 160.2, 149.1, 144.2, 138.60 (d, J = 8.7 Hz), 137.6, 135.9, 135.1, 132.45 (d, J = 9.6 Hz), 129.3, 128.4, 127.4, 120.6 (d, J = 22.7 Hz), 114.1 (d, J = 21.1 H)z, 113.4, 55.4, 50.0, 45.8, 39.0, 38.3, 21.6. ^{19}F NMR (565 MHz, $CDCl_3$) δ –105.67. **HR–MS** (ESI) *m/z* calc. for $C_{26}H_{25}FNO_5S_2$ $[M+H]^+$: 513.1153, found: 514.1148.

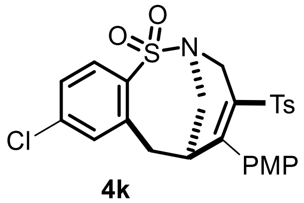

4k

9-Chloro-5-(4-methoxyphenyl)-4-tosyl-6,7-dihydro-3*H*-2,6-methanobenzo[*h*][1,2]thiazonine 1,1-dioxide (4k)

The general procedure (B) was followed using **1a** (107 mg, 0.60 mmol), **2j'** (112.5 mg, 0.3 mmol). Purification by column chromatography on silica gel (petroleum ether/ EtOAc = 3:1) yielded **4k** (76.2 mg, 48%) as a yellow oil. **H NMR** (600 MHz, $CDCl_3$) δ 7.93 (d, J = 8.3 Hz, 1H), 7.31 (d, J = 8.4 Hz, 1H), 7.13 (d, J = 8.3 Hz, 2H), 7.10 (d, J = 8.3 Hz, 2H), 6.89 (d, J = 1.9 Hz, 1H), 6.79 (d, J = 8.9 Hz, 2H), 6.61 (d, J = 5.9 Hz, 2H), 4.44 (d, J = 7.1 Hz, 1H), 4.41 (s, 1H), 4.26 (dd, J = 19.6, 2.3 Hz, 1H), 3.86 (s, 3H), 3.58 (d, J = 15.3 Hz, 1H), 3.46 (dd, J = 14.3, 1.8 Hz, 1H), 2.85 (d, J = 6.3 Hz, 1H), 2.78 (dd, J = 15.3, 7.0 Hz, 1H), 2.40 (s, 3H). **^{13}C NMR** (151 MHz, $CDCl_3$) δ 160.2, 149.1,

144.3, 138.7, 137.2, 135.0, 133.4, 131.1, 129.4, 128.4, 127.4, 127.4, 55.5, 50.1, 45.9, 38.9, 38.2, 21.7. **HR–MS** (ESI) *m/z* calc. for $C_{26}H_{25}ClNO_5S_2$ [M+H]$^+$: 530.0857, found: 530.0860.

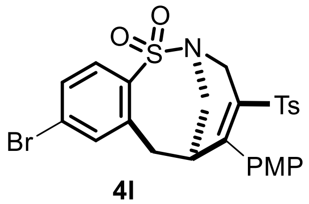

4l

9-Bromo-5-(4-methoxyphenyl)-4-tosyl-6,7-dihydro-3*H*-2,6-methanobenzo[*h*][1,2]thiazonine 1,1-dioxide (4l)

The general procedure (B) was followed using **1a** (107 mg, 0.60 mmol), **2k'** (125.7 mg, 0.3 mmol). Purification by column chromatography on silica gel (petroleum ether/ EtOAc = 3:1) yielded **4l** (77.4 mg, 45%) as a yellow oil. **^{1}H NMR** (400 MHz, $CDCl_3$) δ 7.85 (d, *J* = 8.3 Hz, 1H), 7.48 (dd, *J* = 8.3, 1.8 Hz, 1H), 7.16–7.10 (m, 4H), 7.05 (d, *J* = 1.8 Hz, 1H), 6.80 (d, *J* = 9.0 Hz, 2H), 6.61 (d, *J* = 7.7 Hz, 2H), 4.46–4.40 (m, 2H), 4.27 (dd, *J* = 19.8, 2.3 Hz, 1H), 3.86 (s, 3H), 3.58 (d, *J* = 15.3 Hz, 1H), 3.46 (dd, *J* = 14.2, 1.8 Hz, 1H), 2.86 (d, *J* = 6.7 Hz, 1H), 2.76 (dd, *J* = 15.4, 7.1 Hz, 1H), 2.42 (s, 3H). **^{13}C NMR** (101 MHz, $CDCl_3$) δ 160.2, 149.2, 144.3, 138.9, 137.7, 137.2, 136.4, 135.0, 132.8, 131.1, 130.4, 129.4, 128.3, 127.4, 127.2, 113.4, 55.5, 50.1, 45.9, 38.8, 38.1, 21.7. **HR–MS** (ESI) *m/z* calc. for $C_{26}H_{25}BrNO_5S_2$ [M+H]$^+$: 574.0352, found: 574.0363.

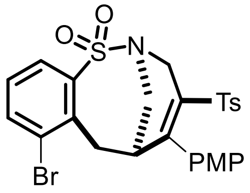

4m

8-Bromo-5-(4-methoxyphenyl)-4-tosyl-6,7-dihydro-3*H*-2,6-methanobenzo[*h*][1,2]thiazonine 1,1-dioxide (4m)

The general procedure (B) was followed using **1a** (107 mg, 0.60 mmol), **2r'** (125.7 mg, 0.3 mmol). Purification by column chromatography on silica gel (petroleum ether/ EtOAc = 3:1) yielded **4m** (73.9 mg, 43%) as a yellow oil. **^{1}H NMR** (600 MHz, $CDCl_3$) δ 8.04 (dd, *J* = 7.8, 1.2 Hz, 1H), 7.74 (dd, *J* = 8.0, 1.3 Hz, 1H), 7.24 (dd, *J* = 7.9, 7.9 Hz, 1H), 7.14 (d, *J* = 8.3 Hz, 2H), 7.06 (d, *J* = 8.0 Hz, 2H), 6.74 (d, *J* = 8.9 Hz, 2H), 6.54 (d, *J* = 8.5 Hz, 2H), 4.55 (d, *J* = 19.6 Hz, 1H), 4.40 (d, *J* = 14.1 Hz, 1H), 4.24 (dd, *J* = 19.6, 2.5 Hz, 1H), 3.83 (s, 3H), 3.65 (dd, *J* = 16.1, 7.5 Hz, 1H), 3.46 (dd, *J* = 13.1, 3.3 Hz, 1H), 3.39 (d, *J* = 16.2 Hz, 1H), 3.01–2.97 (m, 1H), 2.37 (s, 3H). **^{13}C NMR** (151

MHz, $CDCl_3$) δ 160.2, 149.4, 144.1, 142.0, 137.8, 137.6, 135.1, 135.0, 129.4, 129.3, 128.6, 128.3, 127.8, 127.5, 127.2, 113.5, 55.5, 50.6, 46.1, 38.7, 37.4, 21.7. **HR–MS** (ESI) *m/z* calc. for $C_{26}H_{25}BrNO_5S_2$ $[M+H]^+$: 574.0352, found: 574.0343.

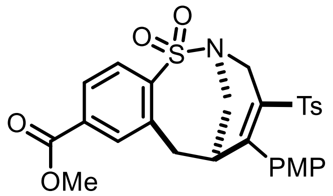

4n

Methyl-5-(4-methoxyphenyl)-4-tosyl-6,7-dihydro-3*H*-2,6-methanobenzo[*h*][1,2]thiazonine-9-carboxylate 1,1-dioxide (4n)

The general procedure (B) was followed using **1a** (107 mg, 0.60 mmol), **2o'** (119.7 mg, 0.3 mmol). Purification by column chromatography on silica gel (petroleum ether/ EtOAc = 3:1) yielded **4n** (79.6 mg, 48%) as a yellow oil. **^{1}H NMR** (600 MHz, $CDCl_3$) δ 8.07 (d, *J* = 8.1 Hz, 1H), 7.98 (dd, *J* = 8.1, 1.2 Hz, 1H), 7.59 (s, 1H), 7.08 (d, *J* = 8.3 Hz, 2H), 7.04 (d, *J* = 8.2 Hz, 2H), 6.75 (d, *J* = 8.9 Hz, 2H), 6.55–6.53 (m, 2H), 4.45 (d, *J* = 3.5 Hz, 1H), 4.42 (d, *J* = 9.1 Hz, 1H), 4.27 (dd, *J* = 19.7, 2.2 Hz, 1H), 4.00 (s, 3H), 3.84 (s, 3H), 3.62 (d, *J* = 15.2 Hz, 1H), 3.47 (dd, *J* = 14.5, 1.8 Hz, 1H), 2.92 (dd, *J* = 15.0, 6.6 Hz, 1H), 2.86 (d, *J* = 6.2 Hz, 1H), 2.37 (s, 3H). **^{13}C NMR** (151 MHz, $CDCl_3$) δ 165.5, 160.15, 149.4, 144.2, 143.7, 137.6, 135.6, 134.8, 134.4, 133.5, 129.9, 129.3, 128.7, 128.4, 128.3, 127.4, 113.2, 55.4, 52.8, 50.1, 45.8, 39.0, 38.3, 21.6. **HR–MS** (ESI) *m/z* calc. for $C_{28}H_{28}NO_7S_2$ $[M+H]^+$: 554.1302, found: 554.1311.

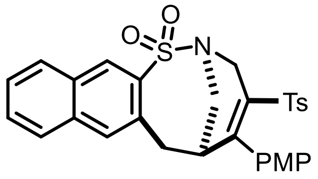

4o

5-(4-Methoxyphenyl)-4-tosyl-6,7-dihydro-3*H*-2,6-methanonaphtho[2,3-*h*][1,2]thiazonine 1,1-dioxide (4o)

The general procedure (B) was followed using **1a** (107 mg, 0.60 mmol), **2n'** (117.3 mg, 0.3 mmol). Purification by column chromatography on silica gel (petroleum ether/ EtOAc = 3:1) yielded **4o** (115.5 mg, 70%) as a yellow oil. **^{1}H NMR** (400 MHz, $CDCl_3$) δ 8.11 (d, *J* = 12.9 Hz, 1H), 7.90 (d, *J* = 8.1 Hz, 1H), 7.85 (d, *J* = 8.7 Hz, 1H), 7.58 (dd, *J* = 7.2, 7.2 Hz, 1H), 7.54 (d, *J* = 8.8 Hz, 1H), 7.36 (dd, *J* = 7.2, 7.2Hz, 1H), 7.14 (d, *J* = 11.0 Hz, 1H), 7.05–6.90 (m, 4H), 6.42 (d, *J* = 7.3 Hz, 2H), 6.06 (d, *J* = 2.5 Hz, 1H), 4.59–4.50 (m, 2H), 4.28 (dd, *J* = 19.6, 2.1 Hz, 1H), 3.95 (dd, *J* = 15.9, 7.6 Hz, 1H), 3.76

(s, 3H), 3.68 (d, *J* = 16.0 Hz, 1H), 3.50 (dd, *J* = 14.2, 2.7 Hz, 1H), 3.08 (d, *J* = 7.2 Hz, 1H), 2.34 (s, 3H). **^{13}C NMR** (151 MHz, $CDCl_3$) δ 159.9, 149.5, 143.9, 138.3, 137.6, 135.6, 134.3, 134.1, 132.4, 129.2, 129.0, 128.3, 128.1, 128.0, 127.7, 127.2, 127.0, 125.2, 124.8, 112.9, 55.3, 50.7, 46.2, 38.3, 31.0, 21.6. **HR–MS** (ESI) *m/z* calc. for $C_{30}H_{28}NO_5S_2$ $[M+H]^+$: 546.1404, found: 546.1394.

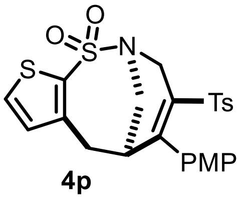

4p

5-(4-Methoxyphenyl)-4-tosyl-6,7-dihydro-3*H*-2,6-methanothieno[3,2-*h*][1,2]thiazonine 1,1-dioxide (4p)

The general procedure (B) was followed using **1a** (107 mg, 0.60 mmol), **2m'** (104.1 mg, 0.3 mmol). Purification by column chromatography on silica gel (petroleum ether/ EtOAc = 3:1) yielded **4p** (58.6 mg, 39%) as a yellow oil. **^{1}H NMR** (400 MHz, $CDCl_3$) δ 7.41 (d, *J* = 4.9 Hz, 1H), 7.19 (d, *J* = 8.2 Hz, 2H), 7.11 (d, *J* = 8.2 Hz, 2H), 6.77 (d, *J* = 8.8 Hz, 2H), 6.65 (d, *J* = 8.0 Hz, 2H), 6.62 (d, *J* = 4.9 Hz, 1H), 4.69 (d, *J* = 19.7 Hz, 1H), 4.51 (d, *J* = 14.8 Hz, 1H), 4.31 (dd, *J* = 19.6, 2.4 Hz, 1H), 3.84 (s, 3H), 3.58 (dd, *J* = 14.4 , 2.4 Hz, 1H), 3.19 (dd, *J* = 15.7, 1.8 Hz, 1H), 2.92 (dd, *J* = 15.2, 5.2 Hz, 1H), 2.72 (d, *J* = 2.3 Hz, 1H), 2.39 (s, 3H). **^{13}C NMR** (151 MHz, $CDCl_3$) δ 156.0, 148.9, 144.2, 139.7, 137.9, 137.8, 135.2, 132.0, 129.6, 129.4, 128.5, 127.4, 113.3, 55.4, 49.7, 45.9, 37.1, 33.2, 21.7. **HR–MS** (ESI) *m/z* calc. for $C_{24}H_{24}NO_5S_3$ $[M+H]^+$: 502.0811, found: 502,0818.

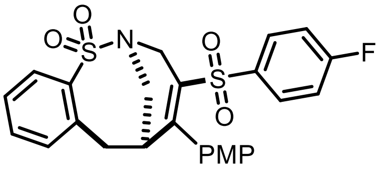

4q

4-((4-Fluorophenyl)sulfonyl)-5-(4-methoxyphenyl)-6,7-dihydro-3*H*-2,6-methanobenzo[*h*][1,2]thiazonine 1,1-dioxide (4q)

The general procedure (B) was followed using **1e** (109.2 mg, 0.60 mmol), **2e'** (102.3 mg, 0.3 mmol). Purification by column chromatography on silica gel (petroleum ether/ EtOAc = 3:1) yielded **4q** (77.8 mg, 52%) as a yellow oil. **^{1}H NMR** (600 MHz, $CDCl_3$) δ 8.04 (d, *J* = 7.7 Hz, 1H), 7.47 (dd, *J* = 7.5, 7.5 Hz, 1H), 7.42 (dd, *J* = 7.5, 7.5 Hz, 1H), 7.16 (dd, *J* = 8.7, 5.1 Hz, 2H), 6.95 (d, *J* = 7.5 Hz, 1H), 6.90 (dd, *J* = 8.5, 8.5 Hz, 2H),

6.75 (d, *J* = 8.7 Hz, 2H), 6.57 (d, *J* = 7.7 Hz, 2H), 4.48 (d, *J* = 14.0 Hz, 1H), 4.46 (d, *J* = 8.4 Hz, 1H), 4.31 (dd, *J* = 19.7, 1.0 Hz, 1H), 3.83 (s, 3H), 3.62 (d, *J* = 14.2 Hz, 1H), 3.47 (d, *J* = 14.3 Hz, 1H), 2.88–2.83 (m, 2H). **^{13}C NMR** (151 MHz, $CDCl_3$) *δ* 165.2 (d, *J* = 273.7 Hz), 160.1, 150.3, 139.8, 136.8 (d, *J* = 2.5 Hz), 135.1, 134.8, 133.5, 132.8, 130.05 (d, *J* = 9.6 Hz), 129.8, 128.3, 127.59, 115.7 (d, *J* = 22.7 Hz), 113.4, 55.4, 50.0, 45.7, 39.1, 38.5. **^{19}F NMR** (565 MHz, $CDCl_3$) *δ* –103.99. **HR–MS** (ESI) *m/z* calc. for $C_{25}H_{23}FNO_5S_2$ $[M+H]^+$: 500.0996, found: 500.1015.

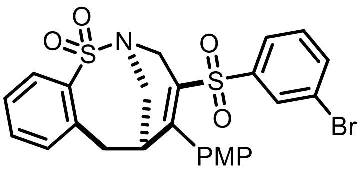

4r

4-((3-Bromophenyl)sulfonyl)-5-(4-methoxyphenyl)-6,7-dihydro-3*H*-2,6-methanobenzo[*h*][1,2]thiazonine 1,1-dioxide (4r)

The general procedure (B) was followed using **1l** (145.2 mg, 0.60 mmol), **2e'** (102.3 mg, 0.3 mmol). Purification by column chromatography on silica gel (petroleum ether/ EtOAc = 3:1) yielded **4r** (80.5 mg, 48%) as a yellow oil. **^{1}H NMR** (600 MHz, $CDCl_3$) *δ* 8.06 (d, *J* = 7.6 Hz, 1H), 7.54–7.53 (m, 1H), 7.49–7.47 m, 1H), 7.44 (dd, *J* = 7.4, 7.4 Hz, 1H), 7.17–7.12 (m, 3H), 6.95 (d, *J* = 7.3 Hz, 1H), 6.74 (d, *J* = 8.8 Hz, 2H), 6.53 (d, *J* = 7.9 Hz, 2H), 4.51 (d, *J* = 19.8 Hz, 1H), 4.47 (d, *J* = 14.3 Hz, 1H), 4.34 (dd, *J* = 19.7, 2.0 Hz, 1H), 3.85 (s, 3H), 3.63 (d, *J* = 14.6 Hz, 1H), 3.48 (d, *J* = 14.3 Hz, 1H), 2.89–2.83 (m, 2H). **^{13}C NMR** (151 MHz, $CDCl_3$) *δ* 160.3, 150.8, 142.4, 139.7, 136.0, 135.0, 134.9, 133.4, 132.8, 130.6, 130.1, 129.8, 127.8, 127.7, 125.6, 122.5, 55.4, 50.0, 45.6, 39.1, 38.5. **HR–MS** (ESI) *m/z* calc. for $C_{25}H_{23}BrNO_5S_2$ $[M+H]^+$: 560.0196, found: 560.1292.

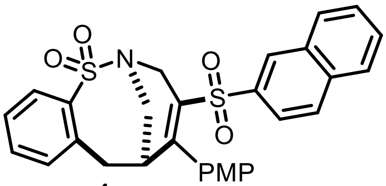

4s

5-(4-Methoxyphenyl)-4-(naphthalen-2-ylsulfonyl)-6,7-dihydro-3*H*-2,6-methanobenzo[*h*][1,2]thiazonine 1,1-dioxide (4s)

The general procedure (B) was followed using **1o** (128.4 mg, 0.60 mmol), **2e'** (102.3 mg, 0.3 mmol). Purification by column chromatography on silica gel (petroleum ether/ EtOAc = 3:1) yielded **4s** (66.9 mg, 42%) as a yellow oil. **^{1}H NMR** (600 MHz, $CDCl_3$)

δ 8.02 (dd, J = 7.6, 0.9 Hz, 1H), 7.84 (d, J = 8.0 Hz, 1H), 7.72 (d, J = 8.6 Hz, 1H), 7.68 (d, J = 8.2 Hz, 1H), 7.65 (dd, J = 7.1, 7.1 Hz, 1H), 7.62 (s, 1H), 7.60–7.54 m, 2H), 7.42–7.39 (m, 1H), 7.30 (d, J = 7.6 Hz, 1H), 7.22 (dd, J = 8.6, 1.8 Hz, 1H), 6.92 (d, J = 7.6 Hz, 1H), 6.55 (s, 2H), 6.51 (d, J = 9.0 Hz, 1H), 4.58 (d, J = 19.7 Hz, 1H), 4.47 (d, J = 14.2 Hz, 1H), 4.38 (dd, J = 19.8, 2.2 Hz, 1H), 3.69 (s, 3H), 3.62 (d, J = 15.4 Hz, 1H), 3.47 (dd, J = 14.2, 1.6 Hz, 1H), 2.87–2.80 (m, 2H). **^{13}C NMR** (151 MHz, $CDCl_3$) δ 159.9, 149.9, 139.8, 137.1, 135.1, 135.1, 134.8, 133.4, 132.7, 131.8, 129.8, 129.7, 129.3, 129.2, 129.0, 128.3, 127.8, 127.5, 121.8, 113.1, 55.2, 50.1, 45.8, 39.1, 38.5. **HR–MS** (ESI) *m/z* calc. for $C_{29}H_{26}NO_5S_2$ $[M+H]^+$: 532.1247, found: 532.1253.

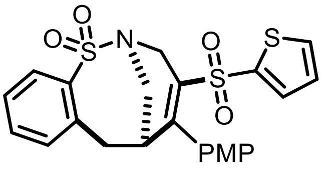

4t

5-(4-Methoxyphenyl)-4-(thiophen-2-ylsulfonyl)-6,7-dihydro-3*H*-2,6-methanobenzo[*h*][1,2]thiazonine 1,1-dioxide (4t)

The general procedure (B) was followed using **1n** (102.0 mg, 0.60 mmol), **2e'** (102.3 mg, 0.3 mmol). Purification by column chromatography on silica gel (petroleum ether/ EtOAc = 3:1) yielded **4t** (70.1 mg, 50%) as a yellow solid. **M.p.**: 202–204 °C. **^{1}H NMR** (600 MHz, $CDCl_3$) δ 7.97 (d, J = 7.7 Hz, 1H), 7.54 (d, J = 4.8 Hz, 1H), 7.44 (dd, J = 7.4, 7.4 Hz, 1H), 7.35 (d, J = 7.6 Hz, 1H), 7.01 (d, J = 3.5 Hz, 1H), 6.94 (d, J = 7.4 Hz, 1H), 6.89 (dd, J = 4.2, 4.2 Hz, 1H), 6.83 (d, J = 8.6 Hz, 2H), 6.73 (d, J = 0.6 Hz, 2H), 4.47 (d, J = 14.3 Hz, 1H), 4.43 (d, J = 19.7 Hz, 1H), 4.30 (dd, J = 19.4, 1.9 Hz, 1H), 3.85 (s, 3H), 3.63 (d, J = 15.1 Hz, 1H), 3.50 (d, J = 14.2 Hz, 1H), 2.92 (s, 1H), 2.87 (dd, J = 15.1, 6.8 Hz, 1H). **^{13}C NMR** (151 MHz, $CDCl_3$) δ 160.1, 150.3, 141.6, 139.8, 135.1, 134.4, 134.0, 133.8, 133.5, 132.6, 129.7, 128.6, 127.5, 127.3, 113.4, 55.4, 50.1, 45.6, 39.2, 38.7. **HR–MS** (ESI) *m/z* calc. for $C_{23}H_{22}NO_5S_3$ $[M+H]^+$: 488.0655, found: 488.0673.

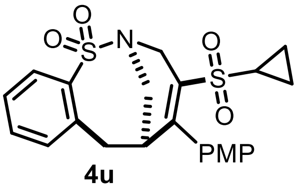

4u

4-(Cyclopropylsulfonyl)-5-(4-methoxyphenyl)-6,7-dihydro-3*H*-2,6-methanobenzo[*h*][1,2]thiazonine 1,1-dioxide (4u)

The general procedure (B) was followed using **1p** (76.8 mg, 0.60 mmol), **2e'** (102.3 mg, 0.3 mmol). Purification by column chromatography on silica gel (petroleum ether/ EtOAc = 3:1) yielded **4u** (41.4 mg, 31%) as a yellow oil. **^{1}H NMR** (600 MHz, $CDCl_3$) δ 8.11 (dd, *J* = 7.2, 0.6 Hz, 1H), 7.53–7.50 (m, 1H), 7.46 (dd *J* = 7.5, 7.5 Hz, 1H), 7.01 (d, *J* = 7.4 Hz, 1H), 6.90–6.89 (m, 4H), 4.52 (d, *J* = 14.3 Hz, 1H), 4.37 (d, *J* = 19.6 Hz, 1H), 4.21 (dd, *J* = 19.6, 2.3 Hz, 1H), 3.85 (s, 3H), 3.69 (d, *J* = 15.3 Hz, 1H), 3.53 (dd, *J* = 14.3, 1.7 Hz, 1H), 3.02 (d, *J* = 6.1 Hz, 1H), 2.94 (dd, *J* = 15.0, 6.6 Hz, 1H), 1.58–1.56 (m, 1H), 1.00–0.97 (m, 1H), 0.91–0.86 (m, 1H), 0.69–0.64 (m, 2H). **^{13}C NMR** (151 MHz, $CDCl_3$) δ 160.3, 148.3, 139.9, 135.3, 134.4, 133.6, 132.8, 129.8, 128.9, 127.9, 127.6, 113.7, 55.4, 50.2, 45.8, 39.2, 38.3, 32.0, 5.8, 5.4. **HR–MS** (ESI) *m/z* calc. for $C_{22}H_{24}NO_5S_2$ $[M+H]^+$: 446.1091, found: 446.1098.

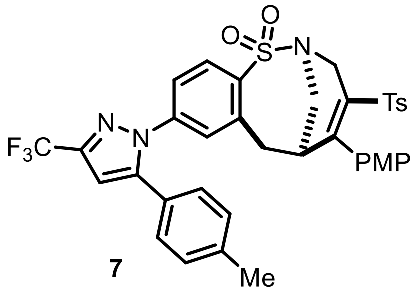

7

5-(4-Methoxyphenyl)-9-(5-(*p*-tolyl)-3-(trifluoromethyl)-1*H*-pyrazol-1-yl)-4-tosyl-6,7-dihydro-3*H*-2,6-methanobenzo[*h*][1,2]thiazonine 1,1-dioxide (7)

The general procedure (B) was followed using **1a** (107 mg, 0.60 mmol), **2q'** (169.5 mg, 0.3 mmol). Purification by column chromatography on silica gel (petroleum ether/ EtOAc = 3:1) yielded **7** (103.5 mg, 48%) as a white solid. **M.p.**: 150–152 °C. **^{1}H NMR** (400 MHz, $CDCl_3$) δ 7.81 (d, *J* = 8.4 Hz, 1H), 7.37 (d, *J* = 1.6 Hz, 1H), 7.23 (d, *J* = 7.9 Hz, 2H), 7.17–7.14 (m, 4H), 6.98 (d, *J* = 8.1 Hz, 2H), 6.87 (d, *J* = 8.6 Hz, 2H), 6.79 (s, 1H), 6.77 (dd, *J* = 8.4, 1.8 Hz, 1H), 6.71 (d, *J* = 8.8 Hz, 2H), 4.46 (d, *J* = 14.4 Hz, 1H), 4.36 (q, *J* = 19.6 Hz, 2H), 3.88 (s, 3H), 3.66 (d, *J* = 13.2 Hz, 1H), 3.50 (d, *J* = 14.3 Hz, 1H), 2.94–2.86 (m, 2H), 2.41 (s, 3H), 2.27 (s, 3H). **^{13}C NMR** (101 MHz, $CDCl_3$) δ 160.2, 149.5, 145.2, 144.0, 141.9, 140.1, 138.8, 137.7, 137.4, 134.7, 130.0, 130.0, 129.9, 129.6, 129.3, 128.8, 128.7, 128.4, 128.3, 127.8, 127.5, 125.7, 122.8, 106.3, 55.3, 50.1, 45.8, 39.1, 38.3, 21.5, 21.4. **^{19}F NMR** (565 MHz, DMSO) δ –60.89. **HR–MS** (ESI) *m/z* calc. for $C_{37}H_{33}F_3N_3O_5S_2$ $[M+H]^+$: 720.1808, found: 720.1825.

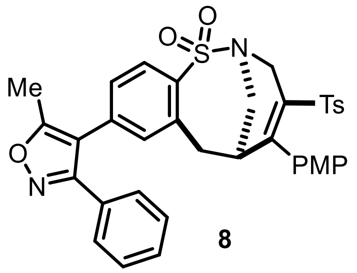

8

5-(4-Methoxyphenyl)-9-(5-methyl-3-phenylisoxazol-4-yl)-4-tosyl-6,7-dihydro-3*H*-2,6-methanobenzo[*h*][1,2]thiazonine 1,1-dioxide (8)

The general procedure (B) was followed using **1a** (107 mg, 0.60 mmol), **2r'** (149.4 mg, 0.3 mmol). Purification by column chromatography on silica gel (petroleum ether/ EtOAc = 3:1) yielded **8** (56.7 mg, 29%) as a yellow solid. **M.p.**: 80–90 °C. **^{1}H NMR** (600 MHz, $CDCl_3$) δ 8.09 (d, J = 8.0 Hz, 1H), 7.51 (dd, J = 7.8, 1.4 Hz, 2H), 7.48–7.43 (m, 3H), 7.27 (dd, J = 8.0, 0.9 Hz, 1H), 7.10 (d, J = 8.2 Hz, 2H), 7.02 (d, J = 8.1 Hz, 2H), 6.81 (s, 1H), 6.55 (d, J = 8.8 Hz, 2H), 6.47 (d, J = 8.2 Hz, 2H), 4.55 (d, J = 19.6 Hz, 1H), 4.46 (d, J = 14.3 Hz, 1H), 4.22 (dd, J = 19.6, 2.0 Hz, 1H), 3.75 (s, 3H), 3.61 (d, J = 15.2 Hz, 1H), 3.44 (dd, J = 14.2, 1.7 Hz, 1H)., 2.84 (s, 1H), 2.79 (dd, J = 15.2, 6.7 Hz, 1H), 2.45 (s, 3H), 2.33 (s, 3H). **^{13}C NMR** (151 MHz, $CDCl_3$) δ 167.5, 160.9, 160.1, 148.9, 144.1, 139.1, 137.4, 135.9, 135.7, 135.3, 134.5, 130.3, 130.0, 129.2, 129.2, 128.5, 128.3, 128.1, 127.4, 114.3, 113.1, 55.5, 49.9, 45.7, 39.3, 38.3, 21.6, 11.7. **HR–MS** (ESI) *m/z* calc. for $C_{36}H_{33}N_2O_6S_2$ $[M+H]^+$: 653.1775, found: 653.1784.

6. Derivatization of the products 4

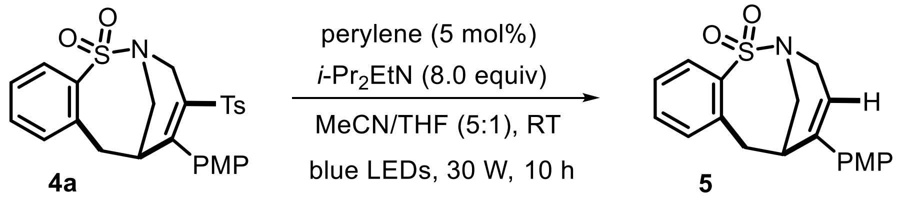

5-(4-Methoxyphenyl)-6,7-dihydro-3*H*-2,6-methanobenzo[*h*][1,2]thiazonine 1,1-dioxide (5)

Compound **4a** (0.3 mmol, 148.5 mg) was placed into a Schlenk tube, perylene (12.8 mg, 0.05 mmol) was added and the flask was evaculated and back filled with N_2. Then, *i*-Pr_2EtN (8.0 eq.) and the solvent mixture (3 mL) were added. The solution was kept stirring under irradiation of 30 W blue LEDs at room temperature for 10 h. Afterwards, the solution was diluted with CH_2Cl_2 and transferred to a round bottom flask. Silica was added to the flask and volatiles were evaporated under vacuum. The purification was performed by flash column chromatography on silica gel (petroleum ether/ EtOAc

= 2:1) to obtain product **5** (35.8 mg, 35% **yield**) as a yellow oil. **^{1}H NMR** (400 MHz, $CDCl_3$) δ 8.00 (d, *J* = 7.5 Hz, 1H), 7.32 (d, *J* = 7.2 Hz, 1H), 7.26 (d, *J* = 7.2 Hz, 1H), 6.95 (d, *J* = 8.6 Hz, 2H), 6.84 (d, *J* = 8.6 Hz, 2H), 6.67 (d, *J* = 7.2 Hz, 1H), 5.42 (s, 1H), 4.56 (d, *J* = 14.1 Hz, 1H), 4.01–3.89 (m, 2H), 3.84 (s, 3H), 3.74 (d, *J* = 14.9 Hz, 1H), 3.54 (d, *J* = 14.1 Hz, 1H), 3.14 (d, *J* = 6.6 Hz, 1H), 3.00 (dd, *J* = 14.8, 7.1 Hz, 1H). **^{13}C NMR** (151 MHz, $CDCl_3$) δ 159.5, 140.0, 138.6, 136.8, 132.8, 132.2, 132.0, 129.4, 127.2, 126.6, 119.0, 114.0, 55.4, 51.6, 46.3, 39.7, 31.9. **HR–MS** (ESI) *m/z* calc. for $C_{19}H_{20}NO_3S$ $[M+H]^+$: 342.1159, found: 342.1156.

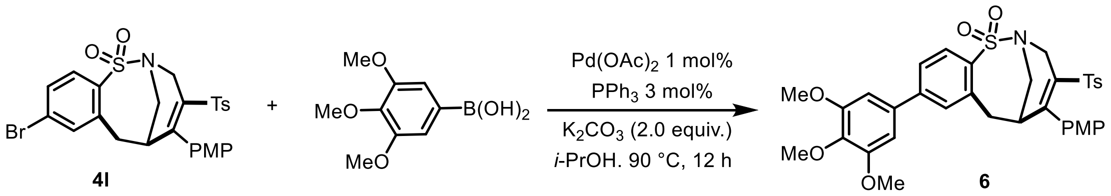

(4-Methoxyphenyl)-4-tosyl-9-(3,4,5-trimethoxyphenyl)-6,7-dihydro-3*H*-2,6-methanobenzo[*h*][1,2]thiazonine 1,1-dioxide (6)

In a 100 mL flask with a stir-bar was charged with **4l** (0.3 mmol) and (3,4,5-trimethoxyphenyl)boronic acid (0.4 mmol). *i*-PrOH (3 mL), $Pd(OAc)_2$ (0.003 mmol) and K_2CO_3 (0.6 mmol) were added and the solution was stirred at RT for 12 hours. The purification was performed by flash column chromatography on silica gel (petroleum ether/ EtOAc = 2:1) to obtain product **6** (128.9mg, 65% **yield**) as a yellow soild. **M.p.**: 207–209 ^oC. **^{1}H NMR** (600 MHz, $CDCl_3$) δ 8.09 (d, *J* = 8.1 Hz, 1H), 7.59 (dd, *J* = 8.1, 1.5 Hz, 1H), 7.09 (d, *J* = 5 Hz, 3H), 7.02 (d, *J* = 8.1 Hz, 2H), 6.75 (s, 2H), 6.72 (d, *J* = 8.6 Hz, 2H), 6.65 (d, *J* = 7.8 Hz, 2H), 4.53 (d, *J* = 19.7 Hz, 1H), 4.48 (d, *J* = 14.2 Hz, 1H), 4.26 (dd, *J* = 19.7, 2.0 Hz, 1H), 3.99 (s, 6H), 3.95 (s, 3H), 3.81 (s, 3H), 3.67 (d, *J* = 14.7 Hz, 1H), 3.47 (d, *J* = 12.9 Hz, 1H), 2.96–2.91 (m, 2H), 2.29 (s, 3H). **^{13}C NMR** (151 MHz, $CDCl_3$) δ 160.1, 153.8, 149.5, 145.3, 144.1, 138.9, 138.3, 137.9, 135.6, 135.2, 134.7, 131.8, 130.4, 129.3, 128.6, 127.3, 125.6, 113.2, 104.6, 61.2, 56.5, 56.5, 55.4, 50.2, 46.0, 39.4, 38.6, 21.6. **HR–MS** (ESI) *m/z* calc. for $C_{35}H_{36}NO_8S_2$ $[M+H]^+$: 662.1877, found: 662.1888.

5. Mechanistic Studies

Radical Trapping Experiment

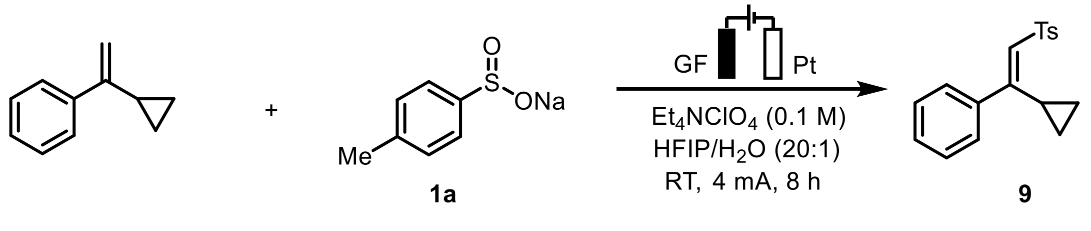

To a 25 mL Schlenk tube containing (1-cyclopropylvinyl)benzene (0.3 mmol, 1.0 equiv.), **1a** (0.3 mmol, 1.0 equiv.), Et_4NClO_4 (92 mg, 0.1 M) were added and subsequently dissolved in a mixture of HFIP/H_2O (3:1, 4 mL). Electrocatalysis was performed at room temperature with a constant current of 4.0 mA maintained for 8 h. The GF anode was washed with ethyl acetate (3 × 3 mL) in an ultrasonic bath and transfered to the round bottom flask with the crude reaction solution. Silica was added to the flask and all volatiles were evaporated under vacuum. Purification by column chromatography on silica gel (petroleum ether/ EtOAc = 3:1) delivered product **9** (53.8 mg, 60%) as a yellow oil. **^{1}H NMR** (600 MHz, $CDCl_3$) δ 7.37 (d, J = 8.2 Hz, 2H), 7.33–7.30 (m, 1H), 7.26 (d, J = 7.7 Hz, 2H), 7.14 (d, J = 8.0 Hz, 2H), 6.92 (d, J = 7.0 Hz, 2H), 6.54 (s, 1H), 2.40 (s, 3H), 1.70–1.67 (m, 1H), 0.84–0.81 (m, 2H), 0.52–0.50 (m, 2H). **^{13}C NMR** (151 MHz, $CDCl_3$) δ 160.3, 143.6, 139.2, 133.8, 129.3, 128.4, 128.2, 127.7, 127.6, 127.0, 21.7, 20.0, 7.1. **HR–MS** (ESI) m/z calc. for $C_{18}H_{19}O_2S$ $[M+H]^+$: 299.1101, found: 299.1102.

6. Density Functional Theory (DFT) Computations

Table S-4 | Calculated activation barriers for radical addition to alkynes and alkenes.

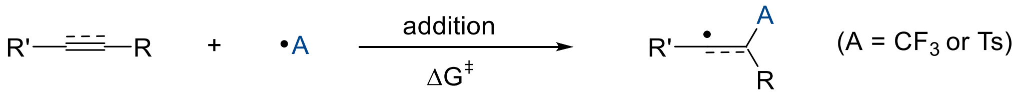

Ehtry	Alkyne/Alkene	ΔG‡/(kcal/mol)^a	ΔG‡/(kcal/mol)^b	Ehtry	Alkyne	ΔG‡/(kcal/mol)^a	ΔG‡/(kcal/mol)^b
1	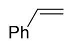	6.6	8.7	5	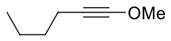	8.3	12.9
2	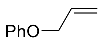	7.5	12.2	6	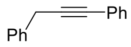	10.0	13.2
3	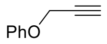	10.0	16.2	7		11.0	14.8
4		8.5	11.4	8	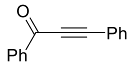	11.0	14.8

The calculated activation barriers for radical addition to various alkenes and alkynes at the M06-2X/def2svp-SMD(DMSO)//M06-2X/def2tzvp-SMD(DMSO) level of theory. ^{a} Additon of $\cdot CF_3$. ^{b} Addition of ·Ts (Ts = *p*-toluene sulfonyl).

Computational details: All density functional theory (DFT) calculations were performed using Gaussian 16.[4] Geometry optimizations and frequencies were calculated at the M06-2X/def-TZVP-SMD(DMSO) level of theory.[5,6] Frequency calculations confirmed that optimized structures are minima (no imaginary frequency) or transition structures (one imaginary frequency). To obtain more accurate electronic energies, single-point energy calculations were performed at the M06-2X-D3/def2-QZVP-SMD(DMSO) level of theory with the optimized structures. Structures were generated using CYLview.[7] Grimme's quasi-RRHO correction[8] for the frequencies that are below 100 cm^{-1} and concentration correction for all species (from 1 atm to 1 mol/L) are implemented by the GoodVibes program.[9]

The calculated Cartesian coordinates and energies of structures

1

C	-2.95242	0.3394	0.00016
C	-1.95027	-0.53576	-0.00014
H	-3.98027	-0.00218	0.00007
H	-2.79416	1.41204	0.00048

H -2.18399 -1.59719 -0.00043
C -0.50944 -0.22396 -0.00008
C 0.40651 -1.27921 0.00001
C -0.01612 1.08579 -0.00011
C 1.77516 -1.03924 0.00009
H 0.03673 -2.29867 0.00001
C 1.34917 1.32614 -0.00003
H -0.70222 1.92414 -0.0002
C 2.25152 0.26522 0.00007
H 2.46796 -1.87202 0.00016
H 1.71389 2.34621 -0.00006
H 3.31741 0.45742 0.00013
M06-2X/def2QZVP-SMD(DMSO): E = -309.651970074 hartree
Corrected Gibbs Free Energy = -309.546512 hartree

2
C 4.18459 0.10512 -0.44251
C 3.15456 -0.21468 0.32683
H 5.15431 -0.36064 -0.31191
H 4.09298 0.84988 -1.22698
H 3.25605 -0.96356 1.10745
C 1.81347 0.4324 0.21633
H 1.59427 1.00504 1.12485
H 1.78036 1.11081 -0.64142
O 0.84404 -0.60482 0.06864
C -0.46486 -0.24571 0.02575
C -1.38303 -1.29584 -0.05718
C -0.91746 1.07178 0.05524
C -2.73954 -1.02772 -0.1098
H -1.0107 -2.31274 -0.07886
C -2.28719 1.32378 0.00283
H -0.2265 1.90117 0.11525
C -3.20315 0.28653 -0.07927
H -3.44167 -1.85036 -0.17353
H -2.63103 2.35092 0.02627
H -4.26481 0.49412 -0.1195
M06-2X/def2QZVP-SMD(DMSO): E = -424.185088496 hartree
Corrected Gibbs Free Energy = -424.049773 hartree

3
C -1.87335 0.6229 -0.00001
H -1.71801 1.24489 -0.88727
H -1.71779 1.24468 0.88736
O -0.97928 -0.4827 -0.00024
C 0.35386 -0.19962 -0.00011
C 1.204 -1.30703 -0.00003
C 0.88282 1.08825 -0.00009
C 2.57555 -1.12415 0.00008
H 0.76952 -2.29905 -0.00004
C 2.26686 1.25374 0.00002
H 0.24522 1.96168 -0.00017
C 3.11795 0.15987 0.00011
H 3.22692 -1.98975 0.00015
H 2.67274 2.25814 0.00004
H 4.19122 0.30079 0.0002
C -3.23833 0.10539 0.0001
C -4.36661 -0.29936 0.00017
H -5.37206 -0.65975 0.00024
M06-2X/def2QZVP-SMD(DMSO): E = -422.937891322 hartree
Corrected Gibbs Free Energy = -422.825899 hartree

4
C -1.50572 1.20422 0.00003
C -0.1187 1.20919 -0.00001
C 0.58383 0. -0.00003
C -0.1187 -1.20919 0.
C -1.50572 -1.20422 0.00004
C -2.2012 0. 0.00005
H -2.04512 2.14318 0.00004
H 0.42805 2.14376 -0.00003
H 0.42805 -2.14376 -0.00002
H -2.04512 -2.14318 0.00005
H -3.28421 0. 0.00009
C 2.01798 0. -0.00008
C 3.21996 0.00001 -0.00009
H 4.28791 -0.00004 0.00041
M06-2X/def2QZVP-SMD(DMSO): E = -308.407090476 hartree
Corrected Gibbs Free Energy = -308.324556 hartree

5
C 1.95999 -0.27618 -0.31048
C 0.83151 -0.6075 -0.06322
C -0.55856 -0.97915 0.21642
H -0.81341 -1.86996 -0.36473
H -0.64766 -1.25986 1.2697
C -1.55685 0.13886 -0.09924
H -1.46719 0.41307 -1.15443
H -1.2976 1.02693 0.48455
C -2.99214 -0.2738 0.20414
H -3.06952 -0.55542 1.25877
H -3.23993 -1.16692 -0.37763
C -3.98905 0.83567 -0.10646
H -3.94493 1.11407 -1.16217
H -5.01236 0.52782 0.11614
H -3.77235 1.72964 0.48332
O 3.18198 0.06469 -0.60236
C 3.89909 0.6728 0.49487
H 4.89216 0.89818 0.11688
H 3.39328 1.58786 0.80372
H 3.95974 -0.02708 1.32852
M06-2X/def2QZVP-SMD(DMSO): E = -349.115622141 hartree
Corrected Gibbs Free Energy = -348.973331 hartree

6

C -1.45709 -1.67302 0.07481
H -1.59019 -2.35488 -0.77066
H -1.60877 -2.2746 0.97517
C -0.08528 -1.17582 0.06303
C 1.04652 -0.76523 0.05163
C 2.38973 -0.26321 0.03732
C 2.63898 1.08303 0.32599
C 3.46045 -1.1107 -0.26683
C 3.93793 1.56934 0.30885
H 1.80996 1.73887 0.56202
C 4.75655 -0.61625 -0.28049
H 3.26833 -2.15269 -0.49049
C 4.99895 0.72269 0.00638
H 4.12229 2.61277 0.53296
H 5.58007 -1.27887 -0.51653
H 6.012 1.10533 -0.00552
C -2.52719 -0.59789 0.01392
C -2.24278 0.73476 -0.26287
C -3.85392 -0.97512 0.2263
C -3.26689 1.67595 -0.32817
H -1.2175 1.0448 -0.42784
C -4.87545 -0.03869 0.16069
H -4.08326 -2.01254 0.44546
C -4.58432 1.2938 -0.11757
H -3.02946 2.71077 -0.54394
H -5.90018 -0.34741 0.32902
H -5.3803 2.02666 -0.16756
M06-2X/def2QZVP-SMD(DMSO): E = -578.789497897 hartree
Corrected Gibbs Free Energy = -578.606781 hartree

7
C 4.12524 1.20409 0.00012
C 2.73855 1.20931 0.00012
C 2.03343 -0.00002 0.
C 2.7386 -1.20932 -0.00012
C 4.12529 -1.20404 -0.00011
C 4.82144 0.00004 0.
H 4.66454 2.14314 0.00021
H 2.19222 2.14413 0.0002
H 2.19231 -2.14417 -0.00021
H 4.66463 -2.14308 -0.0002
H 5.90441 0.00006 0.
C 0.60317 -0.00005 0.
C -0.60317 -0.00007 -0.00001
C -2.03343 -0.00003 0.
C -2.73854 1.20931 -0.00012
C -2.73861 -1.20933 0.00012
C -4.12523 1.20409 -0.00011
H -2.19221 2.14412 -0.00021
C -4.1253 -1.20404 0.00012
H -2.19233 -2.14417 0.0002
C -4.82144 0.00004 0.
H -4.66453 2.14315 -0.0002
H -4.66464 -2.14307 0.00021
H -5.90441 0.00007 0.00001
M06-2X/def2QZVP-SMD(DMSO): E = -539.480595529 hartree
Corrected Gibbs Free Energy = -539.324016 hartree

8
C 0.01229 0.92028 -0.0001
C -1.13969 0.56468 -0.00008
C -2.50187 0.1372 -0.00006
C -2.80527 -1.22915 -0.00063
C -3.53349 1.08286 0.00055
C -4.12878 -1.64003 -0.00059
H -2.00111 -1.95445 -0.0011
C -4.85349 0.66069 0.00059
H -3.29064 2.13795 0.00099
C -5.15228 -0.69791 0.00002
H -4.36304 -2.69709 -0.00104
H -5.65153 1.39244 0.00107
H -6.18521 -1.02331 0.00005
C 1.3872 1.38703 -0.00008
O 1.62876 2.57784 -0.00013
C 2.45938 0.35374 -0.00001
C 2.15983 -1.00795 0.00021
C 3.79021 0.77516 -0.00014
C 3.18656 -1.94234 0.0003
H 1.12795 -1.33795 0.00032
C 4.81178 -0.16009 -0.00005
H 4.01015 1.83526 -0.0003
C 4.5102 -1.51966 0.00016
H 2.95335 -2.99959 0.00047
H 5.84406 0.16659 -0.00016
H 5.31047 -2.24971 0.00023
M06-2X/def2QZVP-SMD(DMSO): E = -652.820211686 hartree
Corrected Gibbs Free Energy = -652.657576 hartree

CF3
C -0.00001 0.00004 0.32621
F 0.85031 0.91737 -0.0725
F 0.36939 -1.19502 -0.07252
F -1.2197 0.27762 -0.07245
M06-2X/def2QZVP-SMD(DMSO): E = -337.619368642 hartree
Corrected Gibbs Free Energy = hartree

Ts
S 2.14228 -0.0025 -0.24187
O 2.66037 1.28335 0.28921
O 2.65473 -1.28796 0.2958
C 0.35264 0.00151 -0.09773
C -0.3113 1.21798 -0.08691
C -1.69663 1.20719 -0.00805
C -2.40693 0.00756 0.02595
C -1.69965 -1.19789 -0.01166
C -0.31792 -1.21491 -0.09046
H 0.23573 2.15157 -0.11327

H -2.23354 2.14769 0.02038
H -2.24237 -2.13572 0.01414
H 0.22619 -2.15015 -0.1188
C -3.90526 -0.0032 0.10441
H -4.23718 -0.52987 1.0015
H -4.30603 1.00921 0.12814
H -4.32975 -0.5253 -0.75558
M06-2X/def2QZVP-SMD(DMSO): E = -819.590768494 hartree
Corrected Gibbs Free Energy = -337.630750 hartree

TS_1+CF3
C 1.05699 1.02421 1.37503
C 0.02813 1.39951 0.59926
H 1.88295 1.7027 1.55248
H 1.04794 0.10613 1.95221
H 0.0727 2.36799 0.10912
C -1.17328 0.60971 0.30904
C -2.19589 1.19124 -0.44815
C -1.33698 -0.71026 0.74926
C -3.35293 0.48455 -0.7477
H -2.07686 2.21017 -0.79981
C -2.49188 -1.41533 0.449
H -0.55639 -1.19182 1.32597
C -3.50512 -0.82185 -0.29981
H -4.13435 0.95359 -1.33314
H -2.60273 -2.43557 0.79594
H -4.40513 -1.37726 -0.53349
C 2.49598 -0.21487 -0.21854
F 2.77248 0.5871 -1.23027
F 3.61439 -0.62769 0.35211
F 1.79332 -1.25022 -0.64113
M06-2X/def2QZVP-SMD(DMSO): E = -647.275700911 hartree
Corrected Gibbs Free Energy = -647.166766 hartree

TS_1+Ts
C 1.8213 -1.95354 -0.90487
C 0.80327 -2.37133 -0.09842
H 2.80072 -2.40565 -0.79833
H 1.63083 -1.43617 -1.83934
H 1.04828 -3.00171 0.75098
C -0.59097 -1.97855 -0.21023
C -1.47947 -2.35223 0.80918
C -1.07937 -1.20122 -1.27283
C -2.80551 -1.94832 0.78073
H -1.11101 -2.95353 1.63297
C -2.40393 -0.80056 -1.2989
H -0.41943 -0.90415 -2.07879
C -3.27179 -1.16627 -0.27097
H -3.47576 -2.23998 1.57996
H -2.76495 -0.19343 -2.12061
H -4.30635 -0.84644 -0.29491
S 2.60381 0.1105 0.10673
O 3.52433 0.84615 -0.8025
O 3.05313 -0.18554 1.49211
C 1.06413 1.01802 0.20427
C 0.68168 1.81649 -0.8643
C -0.56971 2.41405 -0.83285
C -1.44395 2.19716 0.23414
C -1.02826 1.38606 1.292
C 0.22042 0.78292 1.28112
H 1.3533 1.97735 -1.69846
H -0.87807 3.05034 -1.65439
H -1.69562 1.21659 2.12942
H 0.53669 0.14625 2.09818
C -2.82481 2.78459 0.22285
H -2.85559 3.71192 -0.34916
H -3.52305 2.08241 -0.24272
H -3.1789 2.97974 1.2352
M06-2X/def2QZVP-SMD(DMSO): E = -1129.24929794 hartree
Corrected Gibbs Free Energy = -1129.030585 hartree

TS_2+CF3
C -2.10162 0.95283 -1.30393
C -1.45357 1.81518 -0.50954
H -3.10237 1.17584 -1.65502
H -1.58957 0.10913 -1.75113
H -1.95886 2.69655 -0.12755
C -0.05903 1.64037 -0.01619
H 0.52644 2.5429 -0.22918
H -0.07287 1.51101 1.07404
O 0.52828 0.51146 -0.63697
C 1.78993 0.16693 -0.26607
C 2.31454 -0.97178 -0.88152
C 2.55329 0.87174 0.66178
C 3.59301 -1.39985 -0.5698
H 1.70366 -1.50441 -1.60005
C 3.83873 0.42633 0.96419
H 2.16872 1.7567 1.14941
C 4.36596 -0.70309 0.35754
H 3.98998 -2.2844 -1.05313
H 4.42698 0.97908 1.68684
H 5.36573 -1.0397 0.60033
C -3.03723 -0.59026 0.25259
F -3.9402 0.05249 0.97614
F -3.60662 -1.61599 -0.3624
F -2.06519 -1.0237 1.03813
M06-2X/def2QZVP-SMD(DMSO): E = -761.807214471 hartree
Corrected Gibbs Free Energy = -761.668552 hartree

TS_2+Ts
C 2.47758 -2.0123 -0.81141
C 1.28463 -2.27044 -0.19204
H 3.35656 -2.58212 -0.5307
H 2.47768 -1.58317 -1.81034
H 1.25432 -2.85494 0.72084
C -0.01371 -1.76761 -0.72056

H 0.10922 -0.77126 -1.16108
H -0.37884 -2.4367 -1.51229
O -0.95059 -1.73088 0.34612
C -2.16581 -1.17714 0.09514
C -3.02196 -1.06433 1.19319
C -2.58201 -0.73621 -1.1597
C -4.287 -0.52531 1.03278
H -2.6764 -1.40729 2.16084
C -3.85841 -0.19465 -1.30291
H -1.93676 -0.81141 -2.02421
C -4.71579 -0.08632 -0.21893
H -4.94303 -0.44513 1.89122
H -4.17568 0.144 -2.282
H -5.7051 0.3355 -0.34217
S 3.32896 -0.05396 0.11152
O 4.28048 0.5988 -0.82496
O 3.77264 -0.33983 1.49885
C 1.83428 0.91909 0.18681
C 1.45481 1.66043 -0.92333
C 0.19529 2.24335 -0.93403
C -0.68601 2.0685 0.13481
C -0.26721 1.32451 1.2392
C 0.98582 0.73416 1.26974
H 2.12751 1.77598 -1.76414
H -0.11756 2.82231 -1.79521
H -0.94584 1.18028 2.07226
H 1.29655 0.13438 2.11613
C -2.0636 2.66383 0.10914
H -2.08335 3.60702 0.66167
H -2.38705 2.86509 -0.91207
H -2.78135 1.98893 0.5796
M06-2X/def2QZVP-SMD(DMSO): E = -1243.77764748 hartree
Corrected Gibbs Free Energy = -1243.528414 hartree

TS_3+CF3
C 0.01509 0.01808 -0.74038
H 0.1628 0.86862 -0.06469
H -0.25505 0.40655 -1.72766
O -0.99551 -0.84906 -0.24419
C -2.2402 -0.31721 -0.07972
C -3.21285 -1.19748 0.39701
C -2.56866 1.00678 -0.35552
C -4.50743 -0.75151 0.59688
H -2.93361 -2.2231 0.60428
C -3.87756 1.43881 -0.14857
H -1.83188 1.70594 -0.72596
C -4.84982 0.57191 0.32465
H -5.25568 -1.44162 0.96744
H -4.12753 2.47043 -0.36486
H -5.86333 0.91836 0.48123
C 1.25555 -0.7398 -0.83726
C 2.35705 -1.24611 -0.84024
H 3.1645 -1.92586 -1.02515
C 3.74706 0.17767 0.24851
F 4.20548 -0.40788 1.34291
F 4.75862 0.51173 -0.53698
F 3.05751 1.25686 0.5737
M06-2X/def2QZVP-SMD(DMSO): E = -760.555008809 hartree
Corrected Gibbs Free Energy = -760.440596 hartree

TS_3+Ts
C -0.03647 -2.10165 -0.47017
H -0.10843 -1.6309 -1.45681
H -0.50802 -3.09047 -0.52923
O -0.66281 -1.31109 0.52768
C -1.9798 -1.00741 0.34556
C -2.58911 -0.32242 1.3983
C -2.70832 -1.33246 -0.79431
C -3.9223 0.03649 1.30678
H -1.9998 -0.08215 2.27458
C -4.0507 -0.96127 -0.87042
H -2.25904 -1.87002 -1.61803
C -4.66358 -0.27921 0.16874
H -4.38748 0.56739 2.12859
H -4.61405 -1.21754 -1.75957
H -5.70649 0.00247 0.09922
C 1.37171 -2.23985 -0.1245
C 2.57304 -2.12135 0.13728
H 3.48946 -2.63294 0.38412
S 3.29037 -0.03838 0.15118
O 4.14874 0.13249 -1.0453
O 3.84629 0.30421 1.48189
C 1.7677 0.85692 -0.08993
C 1.18222 0.82404 -1.34945
C -0.07363 1.38388 -1.51035
C -0.74188 1.97273 -0.43298
C -0.11738 2.0035 0.8122
C 1.13252 1.42928 0.99973
H 1.69507 0.36293 -2.18539
H -0.55029 1.35838 -2.48402
H -0.62515 2.46213 1.65269
H 1.6053 1.4293 1.97371
C -2.12492 2.52246 -0.62259
H -2.50202 2.97142 0.29574
H -2.13993 3.27601 -1.41281
H -2.80753 1.72104 -0.91929
M06-2X/def2QZVP-SMD(DMSO): E = -1242.52393764 hartree
Corrected Gibbs Free Energy = -1242.298147 hartree

TS_4+CF3
C 3.03159 -1.24819 -0.22672
C 1.78935 -1.17452 0.38328
C 1.19003 0.07354 0.59742
C 1.85145 1.24045 0.19319
C 3.09355 1.15408 -0.41545
C 3.68558 -0.08711 -0.62636
H 3.4915 -2.21467 -0.39109
H 1.27451 -2.07402 0.69674

H 1.38465 2.20302 0.36047
H 3.60196 2.05815 -0.72656
H 4.65623 -0.14959 -1.10246
C -0.08958 0.15398 1.2191
C -1.22273 0.2092 1.64768
H -2.08658 0.28043 2.27541
C -2.59795 -0.01917 -0.24815
F -3.12994 -1.23044 -0.23629
F -3.55715 0.89227 -0.25241
F -1.83625 0.1252 -1.31757
M06-2X/def2QZVP-SMD(DMSO): E = -646.026822096 hartree
Corrected Gibbs Free Energy = -645.941808 hartree

TS_4+Ts
C 2.8183 -0.76805 -1.13199
C 1.49397 -1.09105 -1.3735
C 0.76516 -1.81093 -0.41194
C 1.39217 -2.21763 0.77861
C 2.71722 -1.89051 1.00373
C 3.431 -1.1612 0.055
H 3.37529 -0.20327 -1.86941
H 1.00594 -0.78132 -2.28924
H 0.82464 -2.77118 1.51617
H 3.19718 -2.19857 1.92418
H 4.46614 -0.90192 0.2399
C -0.6081 -2.06757 -0.615
C -1.83601 -2.0655 -0.69974
H -2.76214 -2.53454 -0.9777
S -2.67881 -0.08394 0.11292
O -3.17587 -0.29094 1.49638
O -3.59152 0.51772 -0.89202
C -1.18455 0.89484 0.18682
C -0.35354 0.75878 1.28997
C 0.86769 1.41556 1.28748
C 1.26737 2.18632 0.19366
C 0.40679 2.30174 -0.8996
C -0.81579 1.64625 -0.91941
H -0.65542 0.15326 2.13592
H 1.5258 1.32162 2.14383
H 0.70331 2.90244 -1.75163
H -1.47541 1.72586 -1.77447
C 2.61815 2.83993 0.17954
H 3.37175 2.12775 -0.16974
H 2.63495 3.69942 -0.49043
H 2.90925 3.16342 1.17918
M06-2X/def2QZVP-SMD(DMSO): E = -1127.99800408 hartree
Corrected Gibbs Free Energy = -1127.804329 hartree

TS_5+CF3
C 0.79043 1.3409 -0.36735
C 0.08722 0.37367 -0.1469
C 1.65849 -1.33978 0.0081
F 1.60791 -2.1136 -1.073
F 2.85368 -0.76094 0.0649
F 1.4977 -2.09954 1.08937
C -1.12612 -0.43053 0.07837
H -1.12896 -1.27424 -0.61813
H -1.08194 -0.85836 1.08406
C -2.41203 0.38165 -0.08409
H -2.44547 0.80129 -1.09367
H -2.39376 1.22477 0.6125
C -3.65529 -0.46437 0.16377
H -3.60969 -0.88259 1.17386
H -3.65678 -1.31279 -0.52722
C -4.93955 0.33796 -0.0035
H -5.01721 0.74152 -1.0159
H -5.82189 -0.27776 0.18014
H -4.96667 1.17888 0.69366
O 1.6728 2.24735 -0.61921
C 2.25084 2.86591 0.55958
H 2.97111 3.59013 0.19184
H 1.46666 3.36054 1.13135
H 2.74458 2.10404 1.16184
M06-2X/def2QZVP-SMD(DMSO): E = -686.735952166 hartree
Corrected Gibbs Free Energy = -686.590821 hartree

TS_5+Ts
C -1.34519 0.14036 -0.06604
C -0.68968 1.18712 -0.20637
S -0.03965 -1.63821 0.23117
O -0.21507 -2.61957 -0.87597
O -0.1654 -2.13455 1.6298
C 1.58132 -0.89686 0.05736
C 2.1463 -0.79685 -1.20716
C 3.34958 -0.12403 -1.34818
C 3.97977 0.46399 -0.24811
C 3.37972 0.35638 1.00618
C 2.17663 -0.31831 1.16768
H 1.65666 -1.2451 -2.06285
H 3.80624 -0.04531 -2.32827
H 3.85671 0.81023 1.86722
H 1.71307 -0.39971 2.14335
C 5.29098 1.17585 -0.41808
H 5.51134 1.8071 0.44228
H 6.10389 0.45287 -0.5232
H 5.28579 1.79481 -1.31625
C -2.71727 -0.42326 -0.02928
H -2.80722 -1.16888 -0.82506
H -2.85099 -0.95489 0.91792
C -3.79576 0.64648 -0.18784
H -3.68451 1.38736 0.60934
H -3.64195 1.17088 -1.13546
C -5.19853 0.05187 -0.1484
H -5.29544 -0.69432 -0.94262
H -5.33851 -0.47743 0.79883
C -6.27656 1.11652 -0.30768
H -7.27631 0.67958 -0.27839
H -6.21159 1.85883 0.49144

H -6.16768 1.64109 -1.25996
O 0.25585 2.03745 -0.3625
C 0.602 2.81118 0.82108
H 1.49475 3.36841 0.5555
H -0.22097 3.48418 1.05631
H 0.79837 2.13233 1.64949
M06-2X/def2QZVP-SMD(DMSO): E = -1168.70399190 hartree
Corrected Gibbs Free Energy = -1168.450742 hartree

TS_6+CF3
C 1.61939 1.20305 -0.1598
H 1.7317 1.76819 -1.08743
H 1.74195 1.90556 0.66803
C 0.263 0.6292 -0.10999
C -0.66566 -0.15749 -0.08056
C -1.84553 -0.95321 -0.04422
C -2.33683 -1.43434 1.17738
C -2.52826 -1.25612 -1.23066
C -3.48945 -2.20334 1.20589
H -1.80811 -1.19958 2.09277
C -3.68039 -2.02505 -1.19
H -2.14762 -0.8831 -2.1733
C -4.16303 -2.50063 0.02535
H -3.86426 -2.57228 2.15252
H -4.20421 -2.25504 -2.10952
H -5.06329 -3.10198 0.05222
C 2.67 0.11598 -0.07899
C 3.20232 -0.43867 -1.23951
C 3.09583 -0.35662 1.16021
C 4.14948 -1.4535 -1.1632
H 2.87402 -0.07219 -2.20597
C 4.04238 -1.37059 1.23824
H 2.68351 0.07453 2.06608
C 4.57118 -1.9216 0.07584
H 4.56014 -1.87639 -2.0721
H 4.36998 -1.72867 2.20676
H 5.31065 -2.71078 0.13618
C -0.88634 2.6482 0.04457
F -2.13062 2.42859 -0.34677
F -0.8817 3.01495 1.31822
F -0.35357 3.61202 -0.69472
M06-2X/def2QZVP-SMD(DMSO): E = -916.408479963 hartree
Corrected Gibbs Free Energy = -916.221672 hartree

TS_6+Ts
C 2.54906 -1.29317 -0.05181
H 2.51742 -1.99316 0.78576
H 2.68188 -1.88087 -0.96476
C 1.25086 -0.58157 -0.13157
C 0.58279 0.45813 -0.12407
C -0.39018 1.47861 -0.15459
C -0.71227 2.1129 -1.36865
C -1.08589 1.82811 1.01605
C -1.72115 3.05812 -1.40655
H -0.17342 1.84122 -2.26788
C -2.09865 2.77044 0.96105
H -0.83496 1.33888 1.94899
C -2.4208 3.38489 -0.24641
H -1.97025 3.54019 -2.34367
H -2.64385 3.02514 1.8617
H -3.21598 4.11926 -0.28375
C 3.69569 -0.32237 0.11669
C 4.31351 -0.16537 1.3531
C 4.13434 0.43719 -0.96683
C 5.35913 0.7395 1.50685
H 3.97621 -0.75547 2.19791
C 5.1773 1.34048 -0.81486
H 3.6548 0.316 -1.93237
C 5.79206 1.49437 0.42429
H 5.835 0.85264 2.47344
H 5.51328 1.92378 -1.66364
H 6.60649 2.19851 0.54297
S -0.18559 -2.34053 -0.43221
O 0.08328 -3.37041 0.60611
O -0.16468 -2.75222 -1.86017
C -1.76848 -1.58453 -0.08538
C -2.22941 -1.55978 1.22242
C -3.38743 -0.84967 1.50582
C -4.06459 -0.15013 0.50608
C -3.57192 -0.19481 -0.80019
C -2.42017 -0.90348 -1.10452
H -1.69451 -2.08489 2.00399
H -3.75972 -0.82071 2.52325
H -4.08765 0.34801 -1.58441
H -2.03082 -0.92162 -2.11507
C -5.29869 0.64373 0.82191
H -5.30803 1.58166 0.2643
H -6.19533 0.08505 0.54117
H -5.36071 0.86641 1.88687
M06-2X/def2QZVP-SMD(DMSO): E = -1398.37970800 hartree
Corrected Gibbs Free Energy = -1398.083795 hartree

TS_7+CF3
C 4.31105 -0.64851 -1.20256
C 2.92592 -0.63839 -1.21013
C 2.21996 -0.6198 0.00211
C 2.92512 -0.61342 1.21493
C 4.31026 -0.62366 1.2085
C 5.00579 -0.64155 0.00326
H 4.85211 -0.66156 -2.14045
H 2.37868 -0.64346 -2.14437
H 2.37726 -0.59924 2.14872
H 4.85069 -0.61725 2.14681
H 6.08873 -0.64943 0.00369
C 0.80135 -0.60155 0.00154
C -0.40886 -0.43978 0.00008
C -1.82616 -0.71364 0.00219
C -2.5155 -0.84376 -1.20646

C -2.51508 -0.8289 1.21256
C -3.87793 -1.10483 -1.19939
H -1.97839 -0.74177 -2.14141
C -3.87759 -1.08992 1.20907
H -1.97769 -0.71578 2.14607
C -4.56077 -1.22845 0.00577
H -4.40792 -1.21041 -2.13785
H -4.40732 -1.18402 2.1489
H -5.62464 -1.43099 0.00719
C -0.57411 1.82971 -0.00873
F -1.18441 2.23634 1.09548
F 0.64522 2.34169 -0.05355
F -1.26216 2.23226 -1.0679
M06-2X/def2QZVP-SMD(DMSO): E = -877.097442155 hartree
Corrected Gibbs Free Energy = -876.937191 hartree

TS_7+Ts
C 5.06737 0.82614 0.81008
C 3.68499 0.86852 0.92471
C 2.88975 0.63205 -0.19845
C 3.48263 0.33234 -1.42632
C 4.8655 0.29423 -1.53125
C 5.65848 0.54021 -0.41549
H 5.68356 1.01758 1.67975
H 3.21624 1.09005 1.87558
H 2.85726 0.13579 -2.28832
H 5.32505 0.06951 -2.48573
H 6.73761 0.50817 -0.50132
C 1.44124 0.65798 -0.07737
C 0.37898 1.29804 -0.14425
C -0.94417 1.77055 -0.13684
C -1.76011 1.609 -1.27232
C -1.47894 2.35922 1.02576
C -3.08523 2.00457 -1.22893
H -1.34462 1.15859 -2.16508
C -2.80361 2.75364 1.0503
H -0.84724 2.48187 1.89659
C -3.61173 2.57254 -0.07092
H -3.71444 1.8662 -2.09953
H -3.21422 3.19915 1.94775
H -4.6507 2.87667 -0.04278
S 0.97991 -1.45091 0.52715
O 1.26425 -1.59825 1.97728
O 1.63504 -2.38717 -0.42043
C -0.78909 -1.54026 0.28136
C -1.62826 -1.07588 1.28496
C -2.99145 -1.02068 1.03972
C -3.51657 -1.40947 -0.195
C -2.64539 -1.87584 -1.18054
C -1.27695 -1.93139 -0.9567
H -1.22297 -0.76244 2.2394
H -3.65901 -0.66096 1.81465
H -3.04207 -2.18803 -2.1396
H -0.60168 -2.27966 -1.72827
C -4.98734 -1.28275 -0.46443
H -5.292 -1.90988 -1.30183
H -5.57278 -1.55554 0.41426
H -5.23154 -0.24563 -0.71326
M06-2X/def2QZVP-SMD(DMSO): E = -1359.06753860 hartree
Corrected Gibbs Free Energy = -1358.798446 hartree

TS_8+CF3
C -0.128 0.42918 -0.2532
C 0.91138 -0.20834 -0.25461
C 2.19129 -0.81927 -0.21089
C 2.42574 -1.89986 0.65055
C 3.22391 -0.33342 -1.02537
C 3.68156 -2.48218 0.6935
H 1.62226 -2.26844 1.27589
C 4.47386 -0.92753 -0.9748
H 3.03351 0.50402 -1.68501
C 4.70407 -1.99959 -0.11786
H 3.86447 -3.31561 1.35987
H 5.27209 -0.55351 -1.60339
H 5.68381 -2.45958 -0.08143
C -1.53323 0.73135 -0.53938
O -1.84995 1.80583 -1.00402
C -2.53213 -0.33552 -0.24721
C -2.17473 -1.55198 0.33324
C -3.86788 -0.08438 -0.56964
C -3.14726 -2.50939 0.58769
H -1.14184 -1.75284 0.5878
C -4.83425 -1.04334 -0.31651
H -4.13527 0.86341 -1.01894
C -4.47443 -2.25711 0.263
H -2.86813 -3.45256 1.04011
H -5.8688 -0.84759 -0.56901
H -5.23113 -3.00635 0.46111
C 0.50311 2.5313 0.49028
F -0.3947 3.12075 1.25997
F 1.59802 2.28698 1.19105
F 0.80008 3.3037 -0.53852
M06-2X/def2QZVP-SMD(DMSO): E = -990.438481511 hartree
Corrected Gibbs Free Energy = -990.270817 hartree

TS_8+Ts
C 1.10851 -0.33221 0.55418
C 0.29784 0.59371 0.67473
C -0.80357 1.46309 0.77247
C -1.42666 1.68221 2.01438
C -1.32162 2.06062 -0.3897
C -2.55776 2.47418 2.08046
H -1.02097 1.21529 2.90295
C -2.45991 2.84317 -0.30613
H -0.83479 1.88694 -1.34132
C -3.08036 3.04919 0.92327
H -3.04174 2.64011 3.03463
H -2.86907 3.2923 -1.20257

H	-3.97237	3.66065	0.98055
C	2.53237	-0.7315	0.58997
O	2.85304	-1.78759	1.08496
C	3.52698	0.21619	0.01227
C	3.15297	1.32643	-0.74403
C	4.88016	-0.05045	0.23448
C	4.12692	2.16344	-1.27151
H	2.10765	1.53529	-0.93203
C	5.84772	0.79212	-0.28681
H	5.16054	-0.91634	0.82052
C	5.47148	1.89987	-1.04131
H	3.8348	3.02187	-1.863
H	6.89562	0.58763	-0.10674
H	6.22877	2.5573	-1.45067
S	0.01278	-2.33089	0.25134
O	-0.22206	-3.04791	1.52713
O	0.62833	-3.05488	-0.8875
C	-1.55196	-1.65072	-0.29437
C	-2.51277	-1.33176	0.65591
C	-3.6652	-0.68233	0.24279
C	-3.856	-0.3366	-1.09747
C	-2.87113	-0.6735	-2.02599
C	-1.70739	-1.31986	-1.63139
H	-2.36123	-1.58446	1.69803
H	-4.42382	-0.4248	0.97361
H	-3.00807	-0.41296	-3.06896
H	-0.93759	-1.56529	-2.35247
C	-5.09495	0.40187	-1.51276
H	-5.1795	1.3411	-0.96061
H	-5.0831	0.62656	-2.57864
H	-5.98788	-0.18683	-1.29202

M06-2X/def2QZVP-SMD(DMSO): E = -1472.40849847 hartree

Corrected Gibbs Free Energy = -1472.131933 hartree

7. References

[1] J. A. Johnson, B. M. Petersen, A. Kormos, E. Echeverria, Y.-S. Chen, J. Zhang, *J. Am. Chem. Soc*. **2016**, *138*, 10293–10298.

[2] R. Kim, K. Kwon, H.-Y. Lee, *Chem. Asian J.* **2021**, *16*, 3909–3913.

[3] P. Chen, Q. Zhou, Z. Chen, Y.-K. Liu, Y. Liang, K.-W. Tang, *Org. Biomol. Chem.* **2020**, *18*, 7345–7354.

[4] M. J. Frisch, G. W. Trucks, H. B. Schlegel, G. E. Scuseria, M. A. Robb, J. R. Cheeseman, G. Scalmani, V. Barone, G. A. Petersson, H. Nakatsuji, X. Li, M. Caricato, A. V. Marenich, J. Bloino, B. G. Janesko, R. Gomperts, B. Mennucci, H. P. Hratchian, J. V. Ortiz, A. F. Izmaylov, J. L. Sonnenberg, D. Williams-Young, F. Ding, F. Lipparini, F. Egidi, J. Goings, B. Peng, A. Petrone, T. Henderson, D. Ranasinghe, V. G. Zakrzewski, J. Gao, N. Rega, G. Zheng, W. Liang, M. Hada, M. Ehara, K. Toyota, R. Fukuda, J. Hasegawa, M. Ishida, T. Nakajima, Y. Honda, O. Kitao, H. Nakai, T. Vreven, K. Throssell, Jr. J. A. Montgomery, J. E. Peralta, F. Ogliaro, M. J. Bearpark, J. J. Heyd, E. N. Brothers, K. N. Kudin, V. N. Staroverov, T. A. Keith, R. Kobayashi, J. Normand, K. Raghavachari, A. P. Rendell, J. C. Burant, S. S. Iyengar, J. Tomasi, M. Cossi, J. M. Millam, M. Klene, C. Adamo, R. Cammi, J. W. Ochterski, R. L. Martin, K. Morokuma, O. Farkas, J. B. Foresman, and D. J. Fox. *Gaussian 16, Revision A.03*, Gaussian, Inc., Wallingford CT, **2016**.

[5] (a) C. Lee, W. Yang, R. G. Parr. Development of the Colle-Salvetti Correlation-Energy Formula into a Functional of the Electron Density. *Phys. Rev. B: Condens.*

Matter Mater. Phys. **1988**, *37*, 785. (b) A. D. Becke. Density‐functional thermochemistry. III. The Role of Exact Exchange. *J. Chem. Phys*. **1993**, *98*, 5648.

[6] A. V. Marenich, C. J. Cramer, D. G. Truhlar. Universal Solvation Model Based on Solute Electron Density and on a Continuum Model of the Solvent Defined by the Bulk Dielectric Constant and Atomic Surface Tensions. *J. Phys. Chem. B* **2009**, *113*, 6378.

[7] C. Y. Legault. CYLview, 1.0b; Université de Sherbrooke, Sherbrooke, Canada, **2009**, http://www.cylview.org.

[8] S. Grimme. Supramolecular Binding Thermodynamics by Dispersion-Corrected Density Functional Theory. *Chem. Eur. J*. **2012**, *18*, 9955.

[9] G. Luchini, J. V. Alegre-Requena, Y. Guan, I. Funes-Ardoiz, R. S. Paton. **2019**, GoodVibes: GoodVibes 3.0.1. http://doi.org/10.5281/zenodo.595246.

7. NMR Spectra

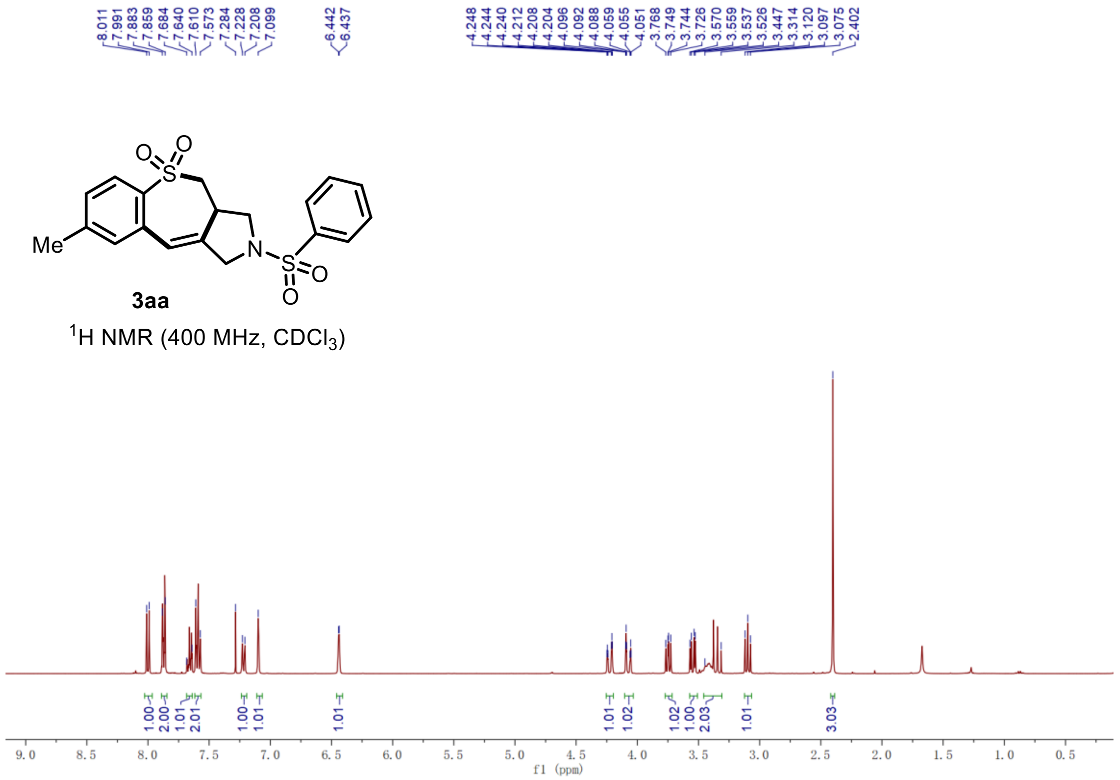

3aa

^{1}H NMR (400 MHz, $CDCl_3$)

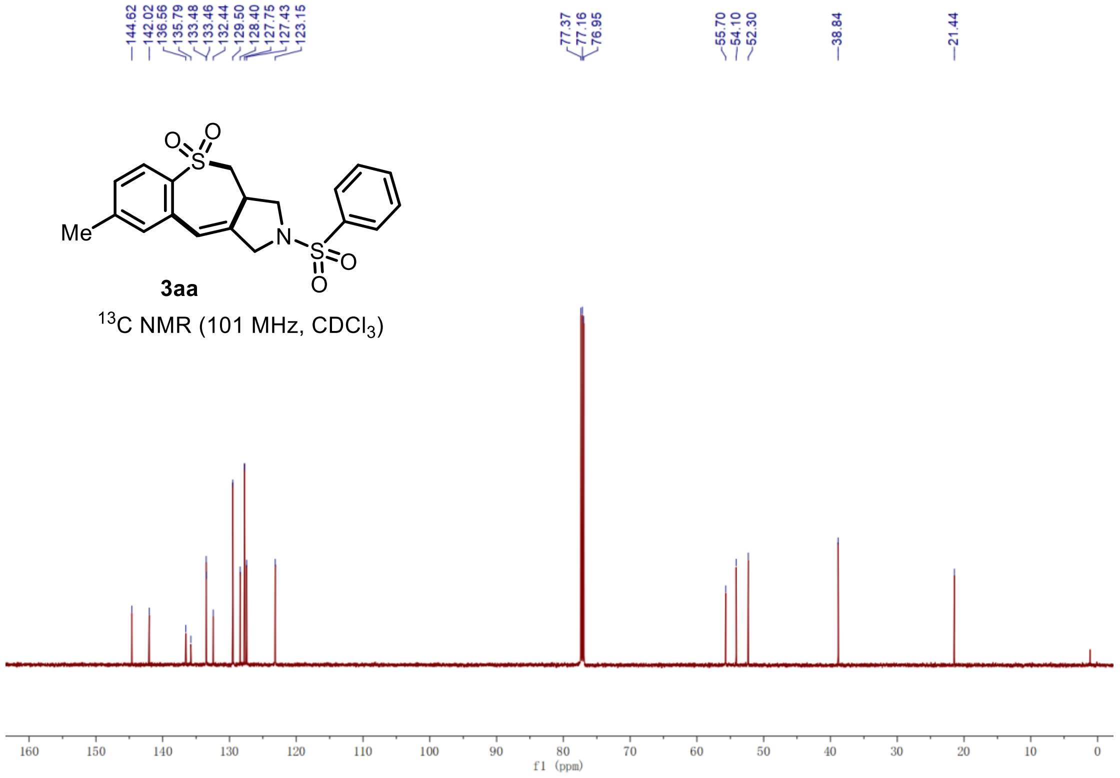

3aa

^{13}C NMR (101 MHz, $CDCl_3$)

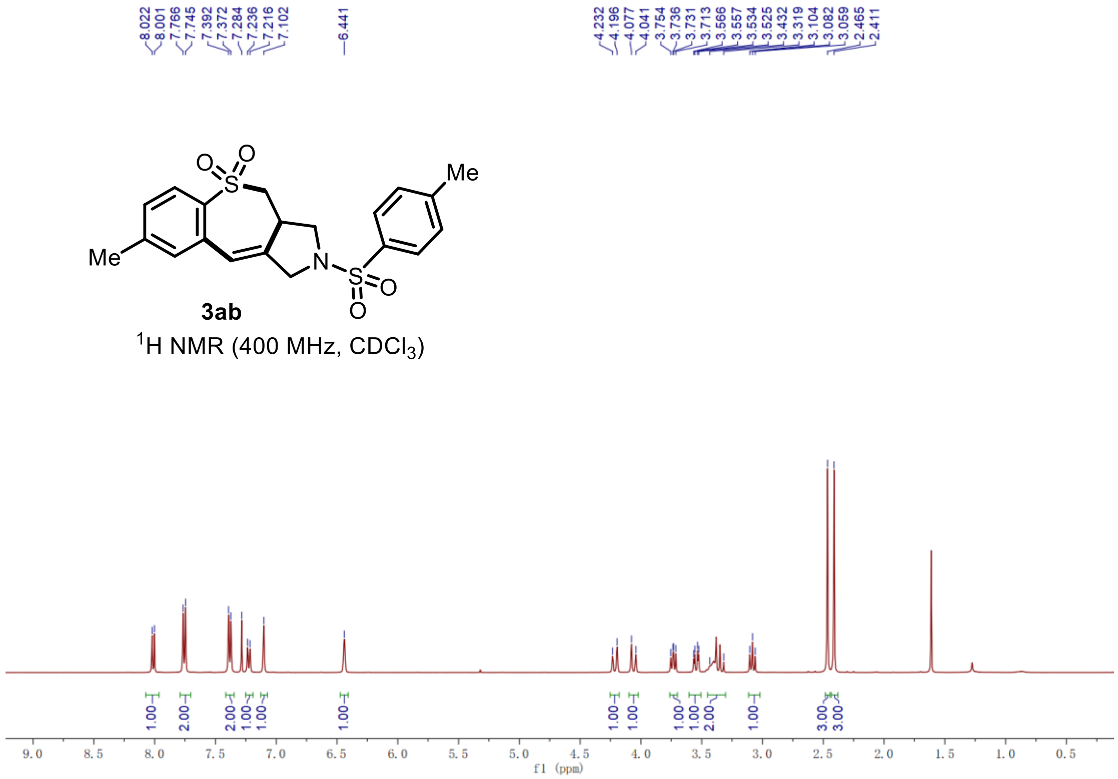
8.022
8.001
7.766
7.745
7.392
7.372
7.284
7.236
7.216
7.102
6.441
4.232
4.196
4.077
4.041
3.754
3.736
3.731
3.713
3.566
3.557
3.534
3.525
3.432
3.319
3.104
3.082
3.059
2.465
2.411
Me
O
S
O
N
S
O
O
Me
3ab
^{1}H NMR (400 MHz, CDCl$_3$)
1.00
2.00
2.00
1.00
1.00
1.00
1.00
1.00
1.00
1.00
2.00
1.00
3.00
3.00
f1 (ppm)

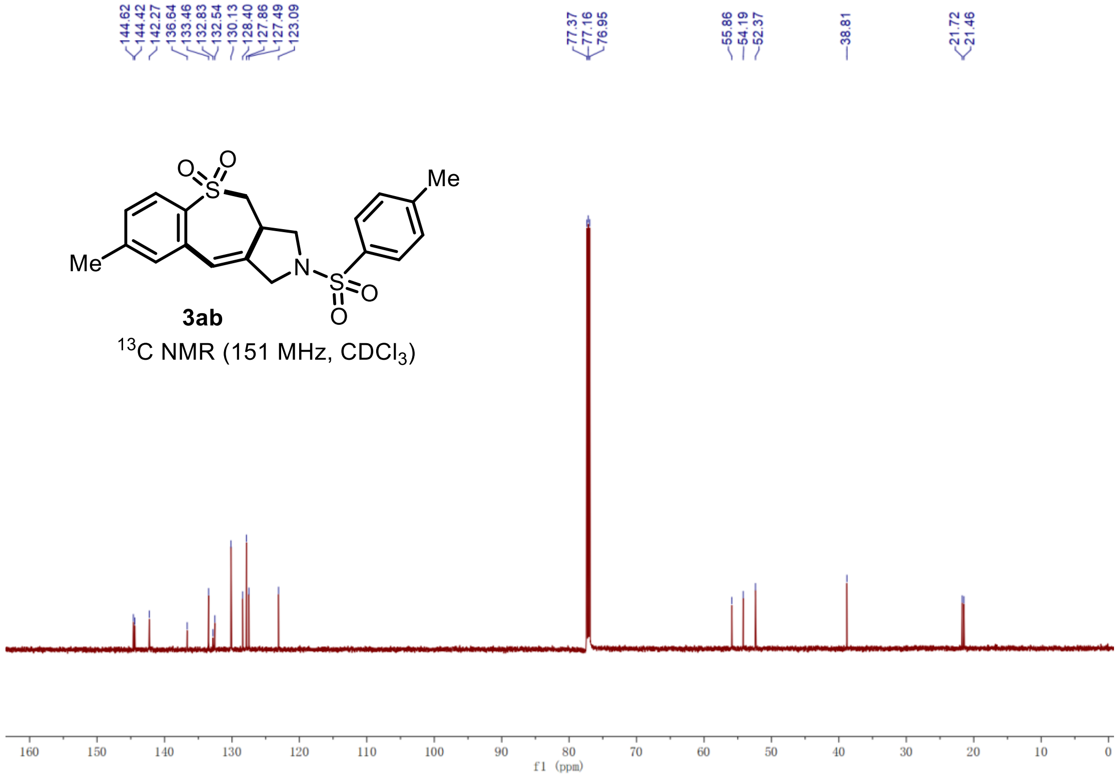
144.62
144.42
142.27
136.64
133.46
132.83
132.54
130.13
128.40
127.86
127.49
123.09
77.37
77.16
76.95
55.86
54.19
52.37
38.81
21.72
21.46
Me
O
S
O
N
S
O
O
Me
3ab
^{13}C NMR (151 MHz, CDCl$_3$)
f1 (ppm)

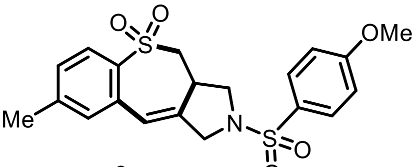

3ac

^{1}H NMR (400 MHz, $CDCl_3$)

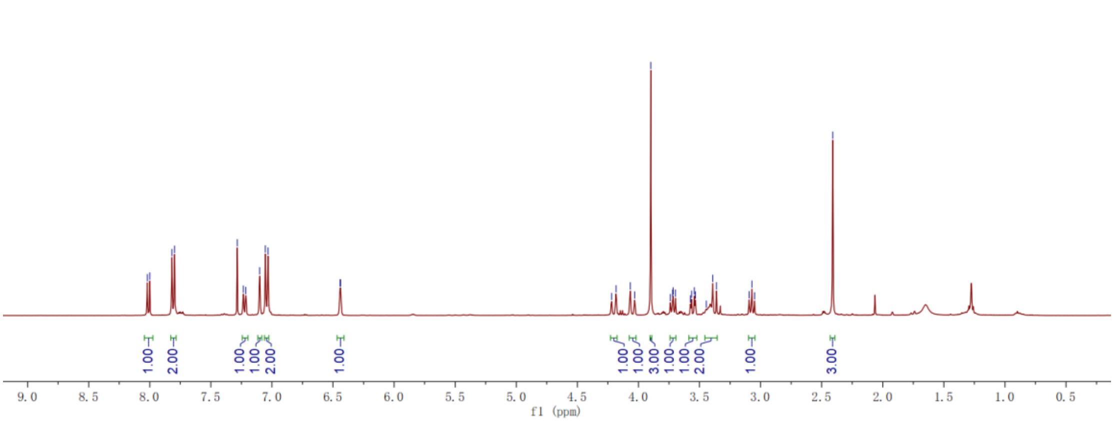

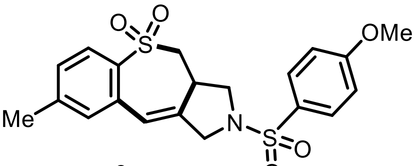

3ac

^{13}C NMR (151 MHz, $CDCl_3$)

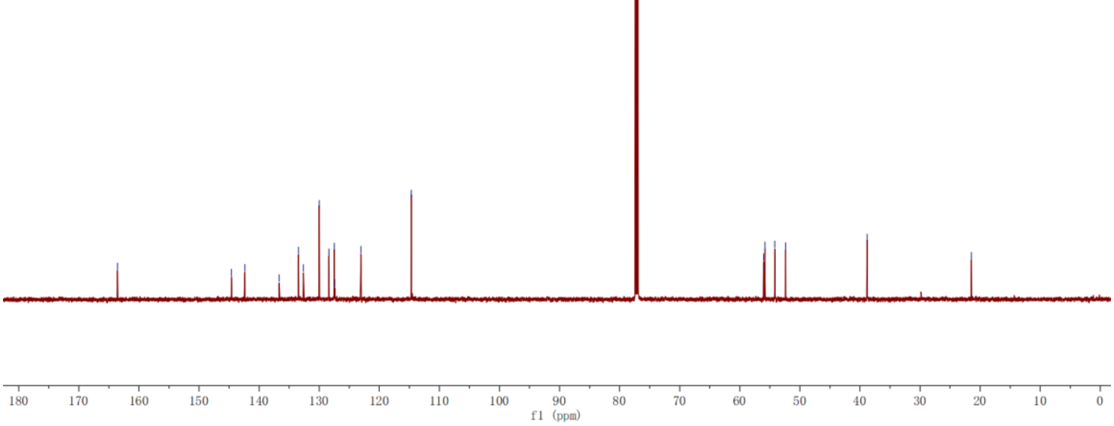

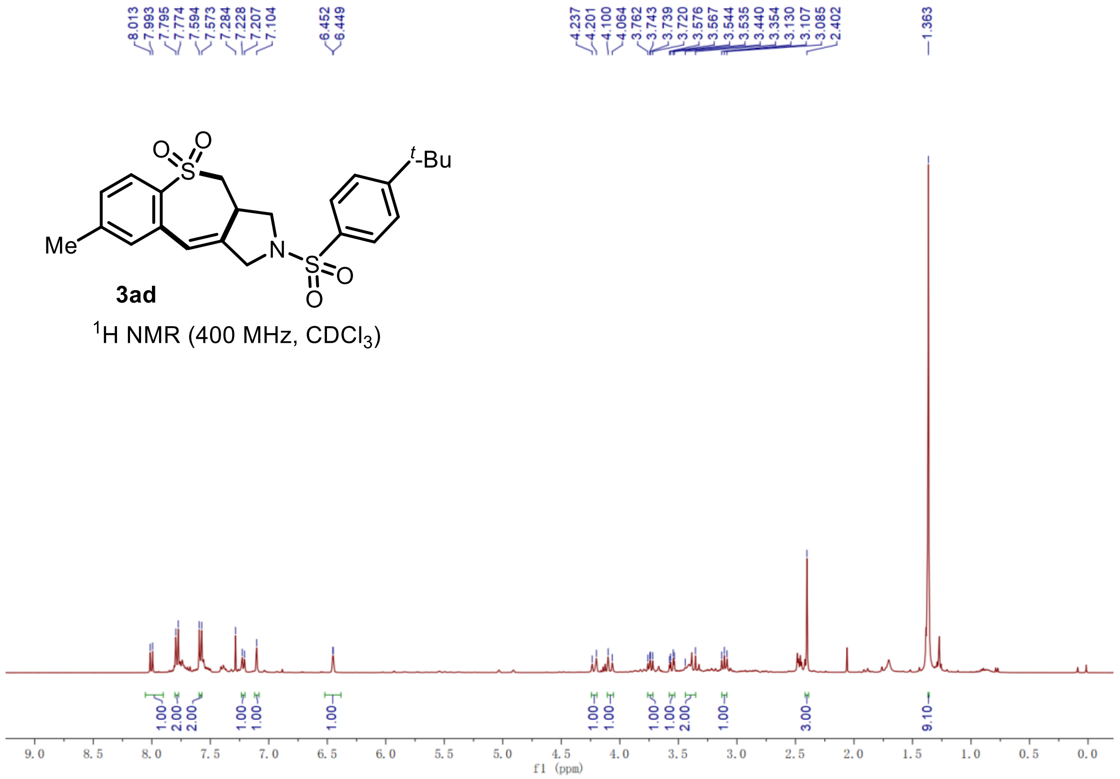
8.013
7.993
7.795
7.774
7.594
7.573
7.284
7.228
7.207
7.104
6.452
6.449
4.237
4.201
4.100
4.064
3.762
3.743
3.739
3.720
3.576
3.567
3.544
3.535
3.440
3.354
3.130
3.107
3.085
2.402
1.363
Me
t-Bu
3ad
^{1}H NMR (400 MHz, CDCl$_3$)
1.00
2.00
2.00
1.00
1.00
1.00
1.00
1.00
1.00
1.00
2.00
1.00
3.00
9.10
f1 (ppm)

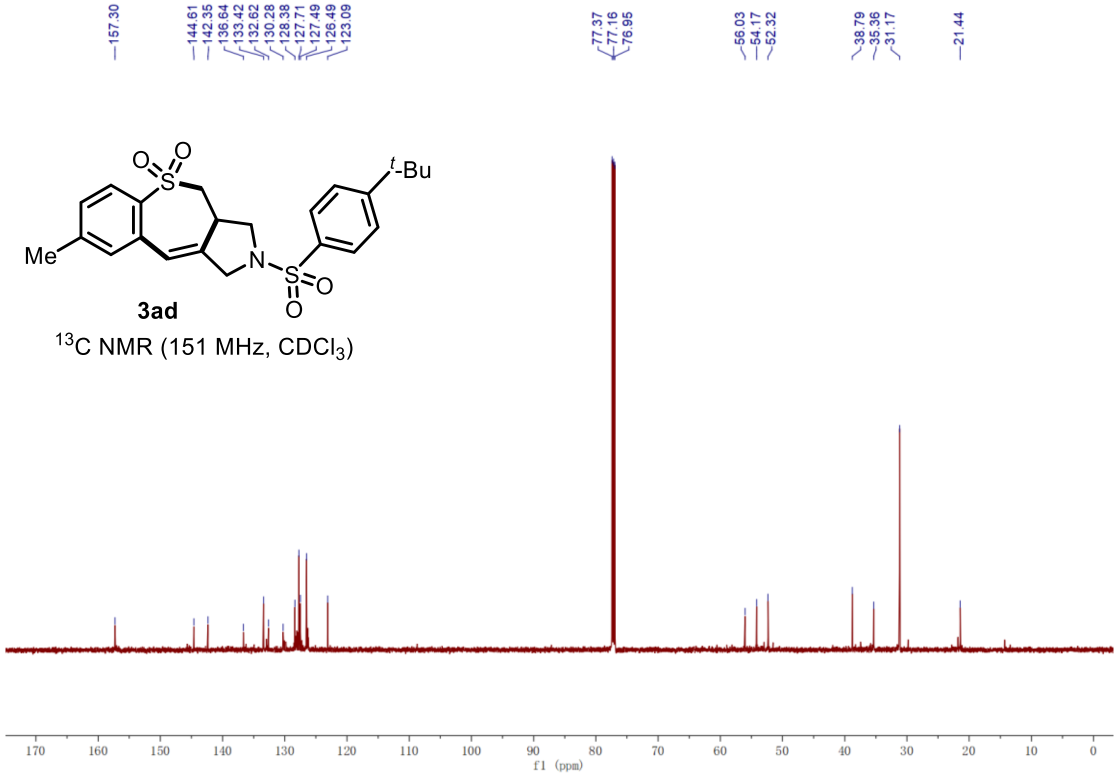
157.30
144.61
142.35
136.64
133.42
132.62
130.28
128.38
127.71
127.49
126.49
123.09
77.37
77.16
76.95
56.03
54.17
52.32
38.79
35.36
31.17
21.44
Me
t-Bu
3ad
^{13}C NMR (151 MHz, CDCl$_3$)
f1 (ppm)

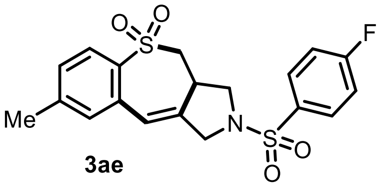

3ae

^{1}H NMR (400 MHz, $CDCl_3$)

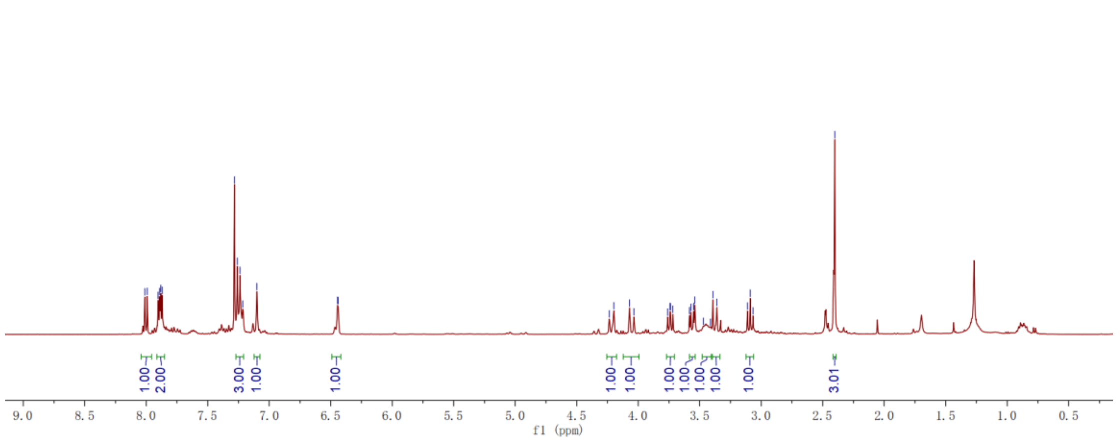

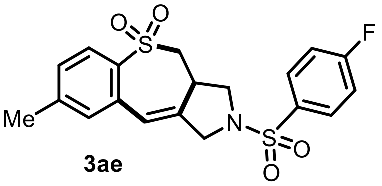

3ae

^{13}C NMR (101 MHz, $CDCl_3$)

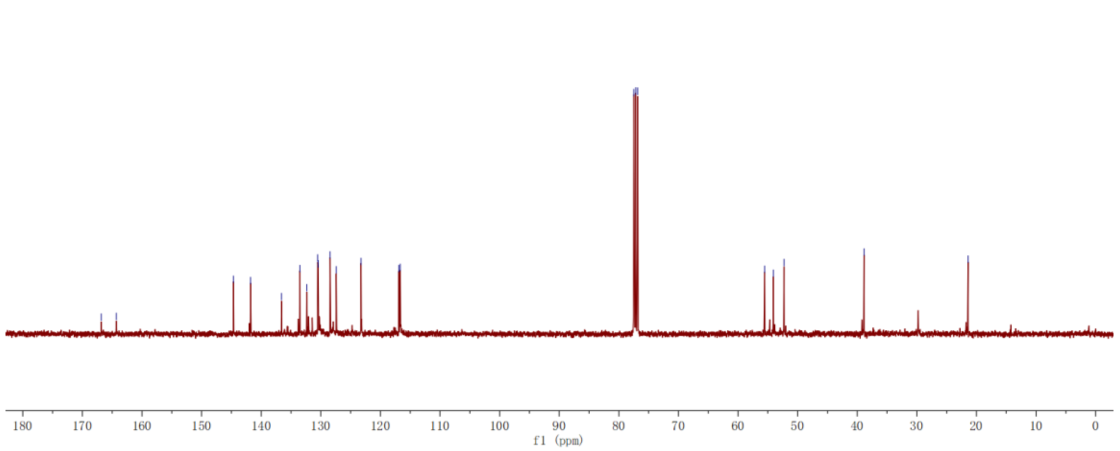

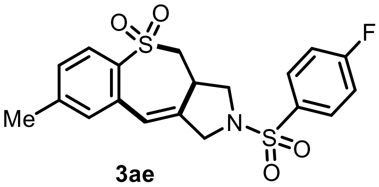

3ae

^{19}F NMR (377 MHz, $CDCl_3$)

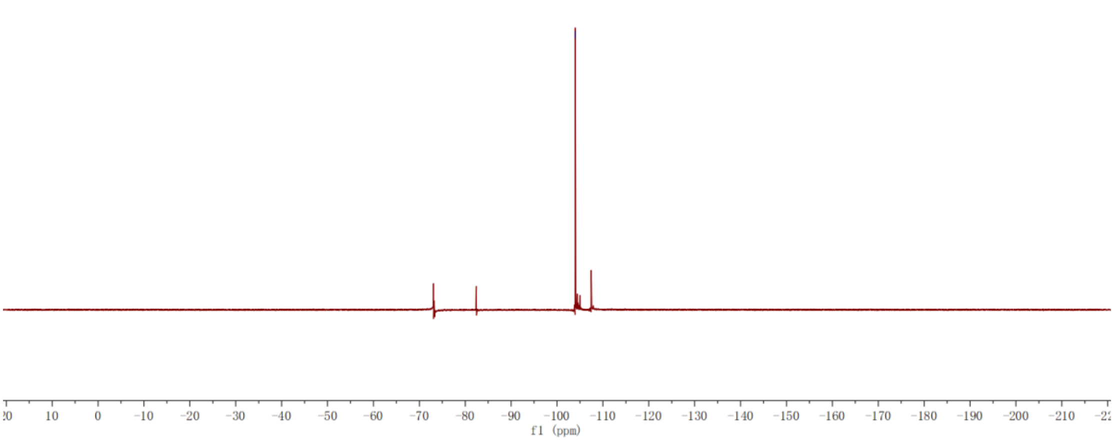

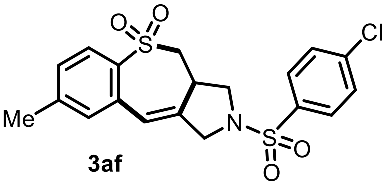

3af

^{1}H NMR (400 MHz, $CDCl_3$)

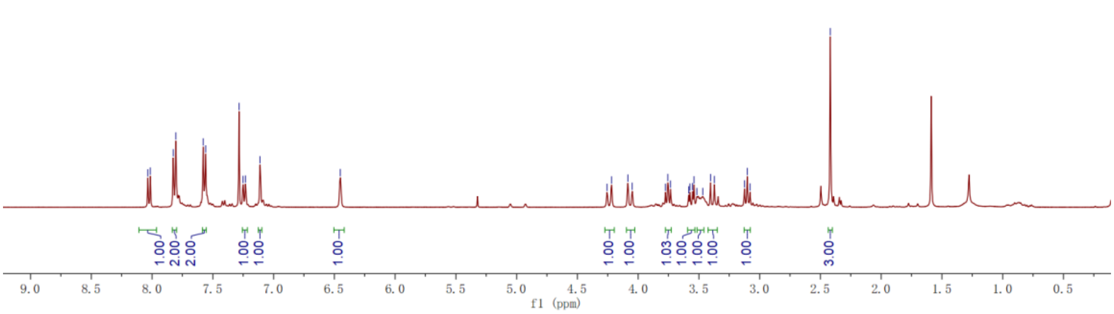

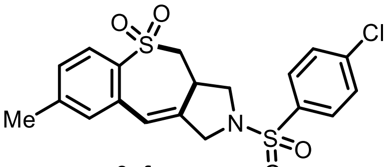

3af

^{13}C NMR (101 MHz, $CDCl_3$)

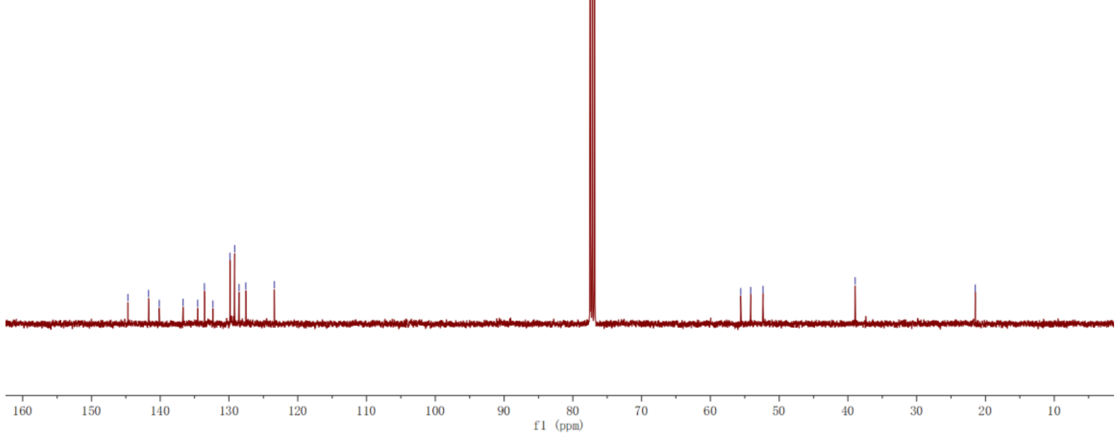

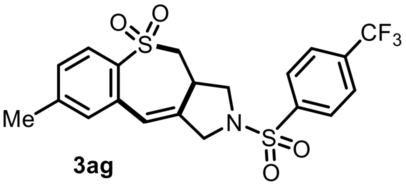

3ag

^{1}H NMR (400 MHz, DMSO)

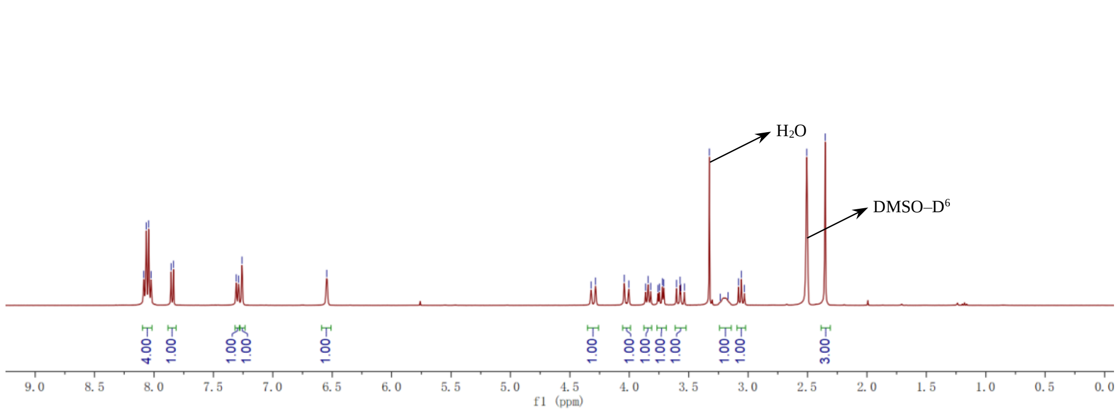

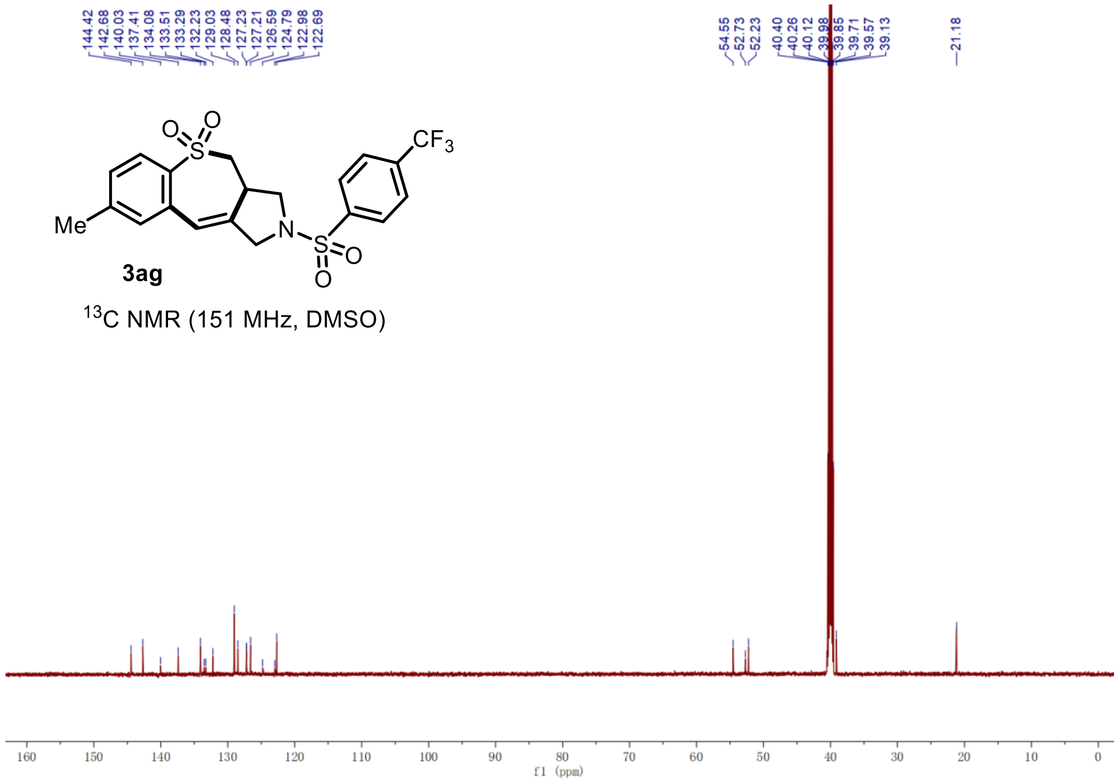
144.42
142.68
140.03
137.41
134.08
133.51
133.29
132.23
129.03
128.48
127.23
127.21
126.59
124.79
122.98
122.69
54.55
52.73
52.23
40.40
40.26
40.12
39.98
39.85
39.71
39.57
39.13
21.18
CF3
Me
3ag
13C NMR (151 MHz, DMSO)
f1 (ppm)

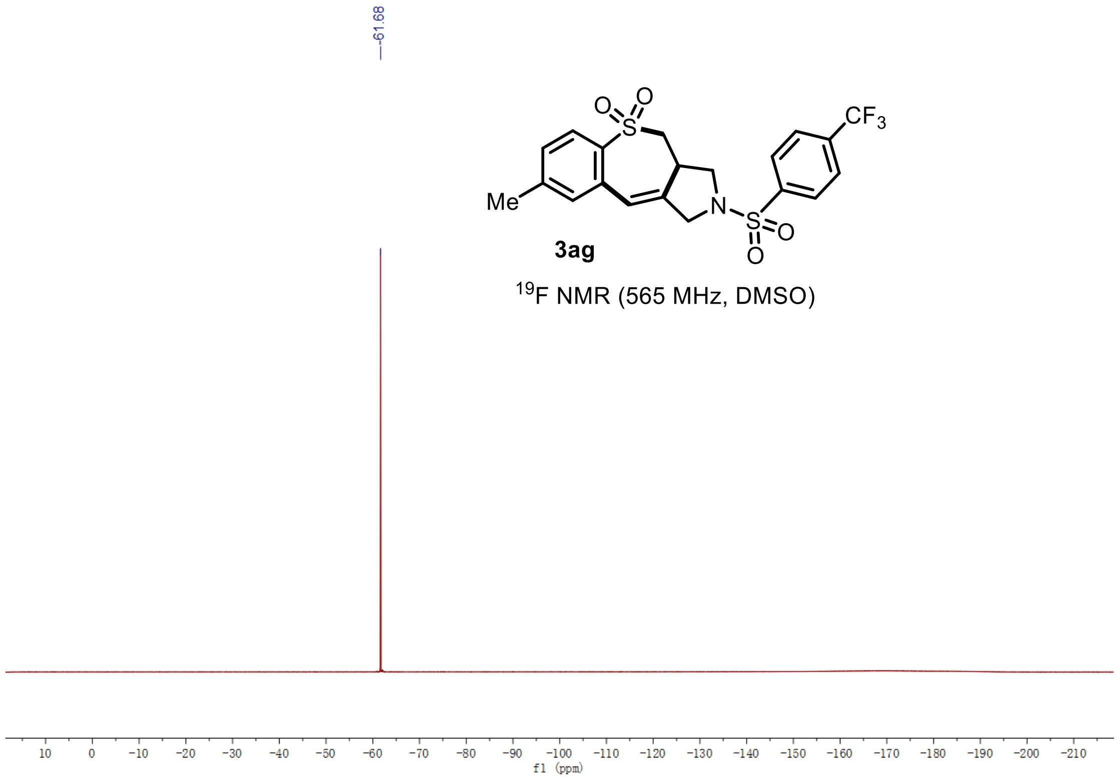
-61.68
CF3
Me
3ag
19F NMR (565 MHz, DMSO)
f1 (ppm)

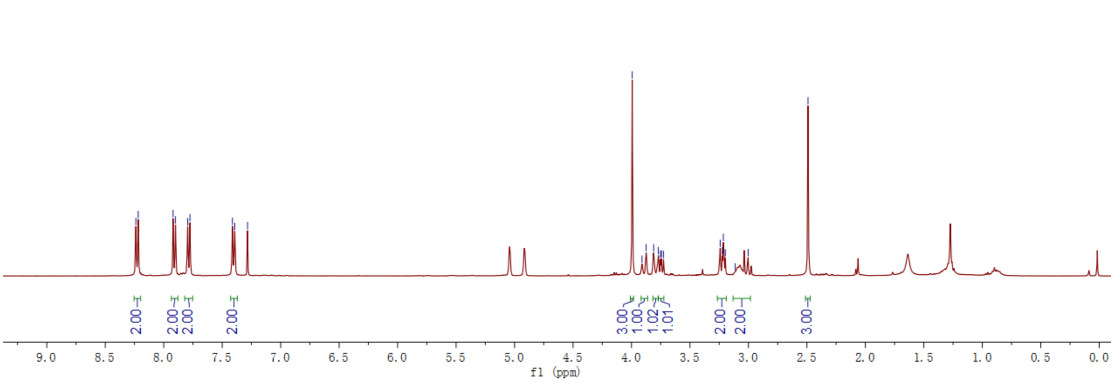

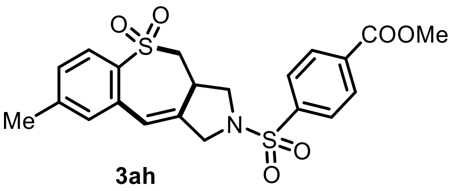

3ah

^{1}H NMR (400 MHz, CDCl3)

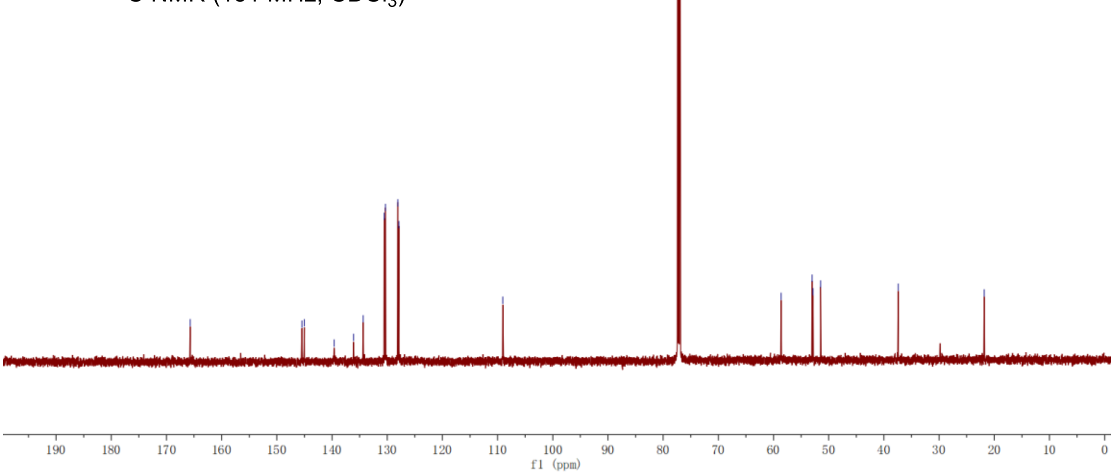

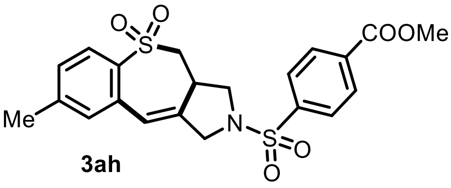

3ah

^{13}C NMR (151 MHz, $CDCl_3$)

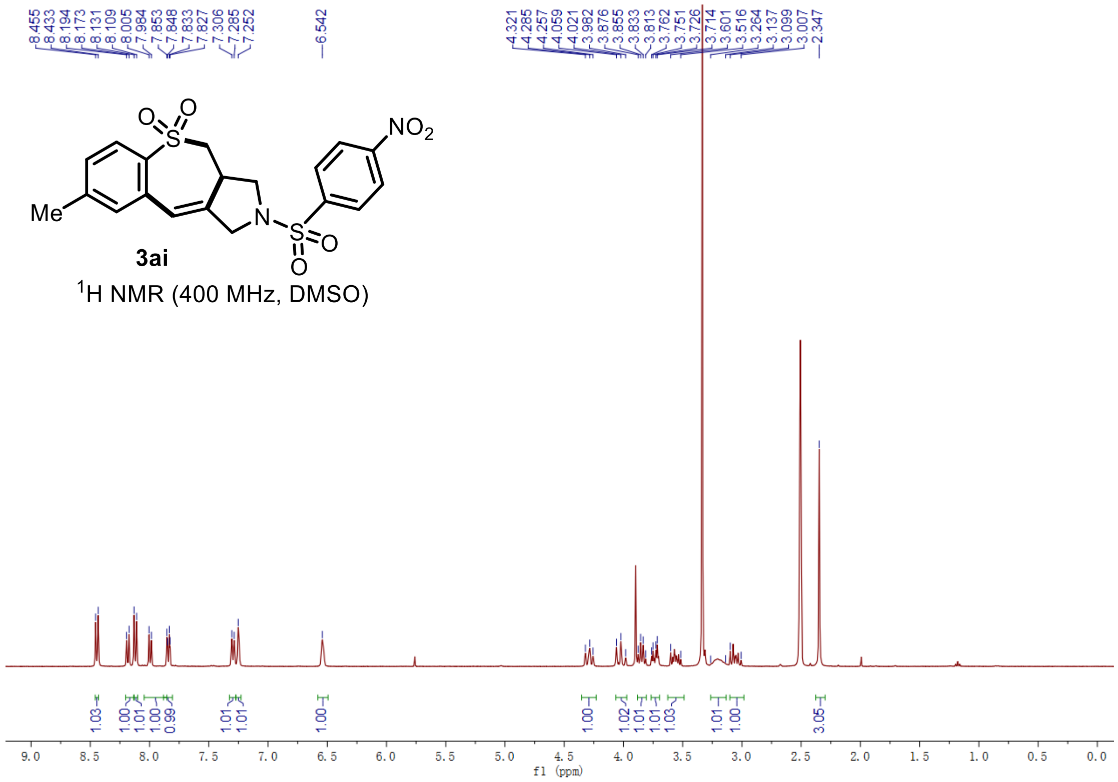

NO2
Me
3ai
¹H NMR (400 MHz, DMSO)
f1 (ppm)

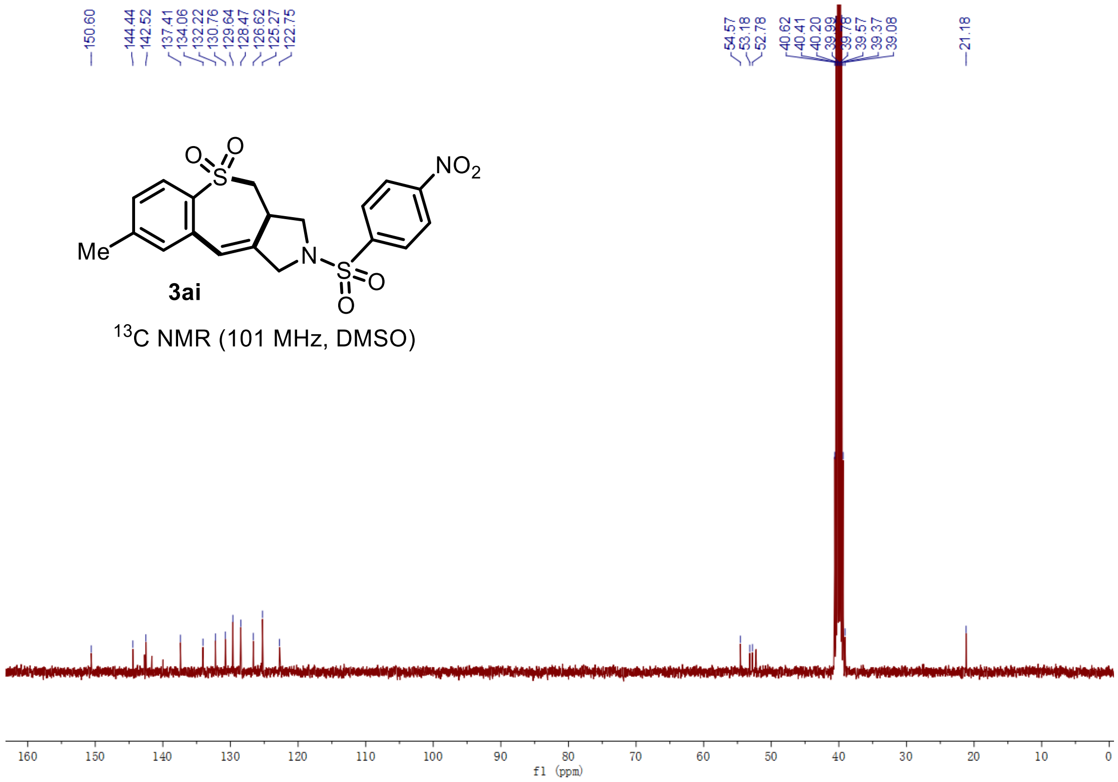

NO2
Me
3ai
¹³C NMR (101 MHz, DMSO)
f1 (ppm)

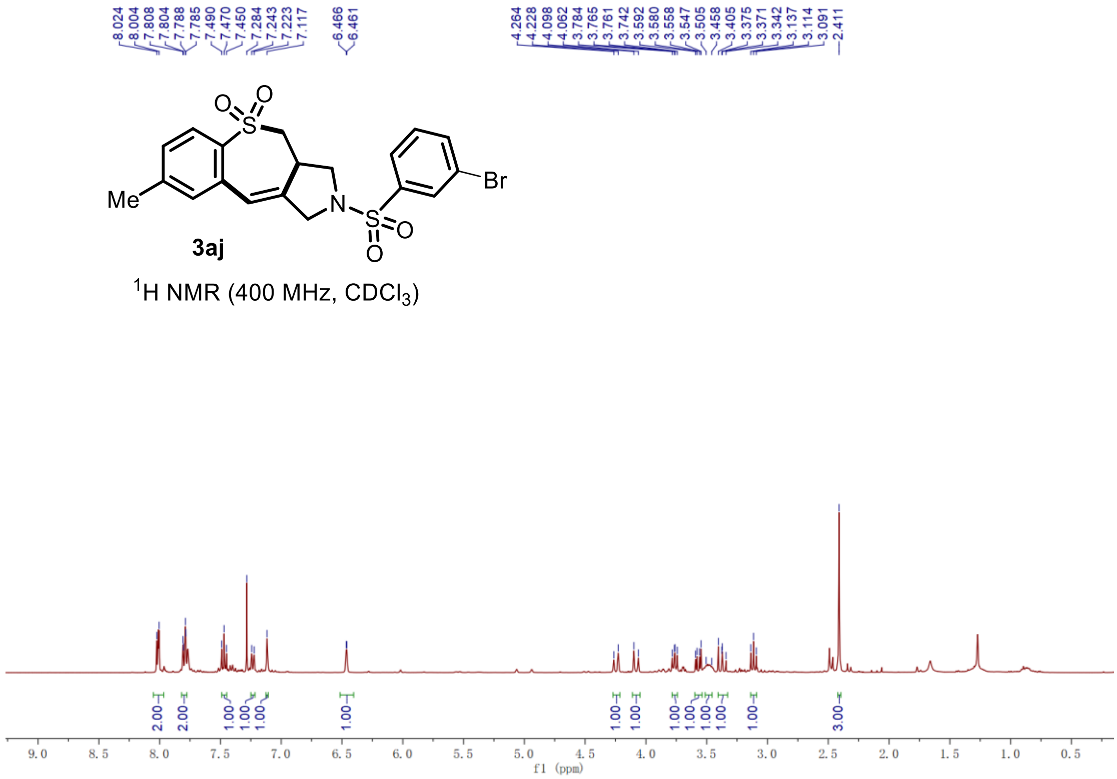

3aj

^{1}H NMR (400 MHz, $CDCl_3$)

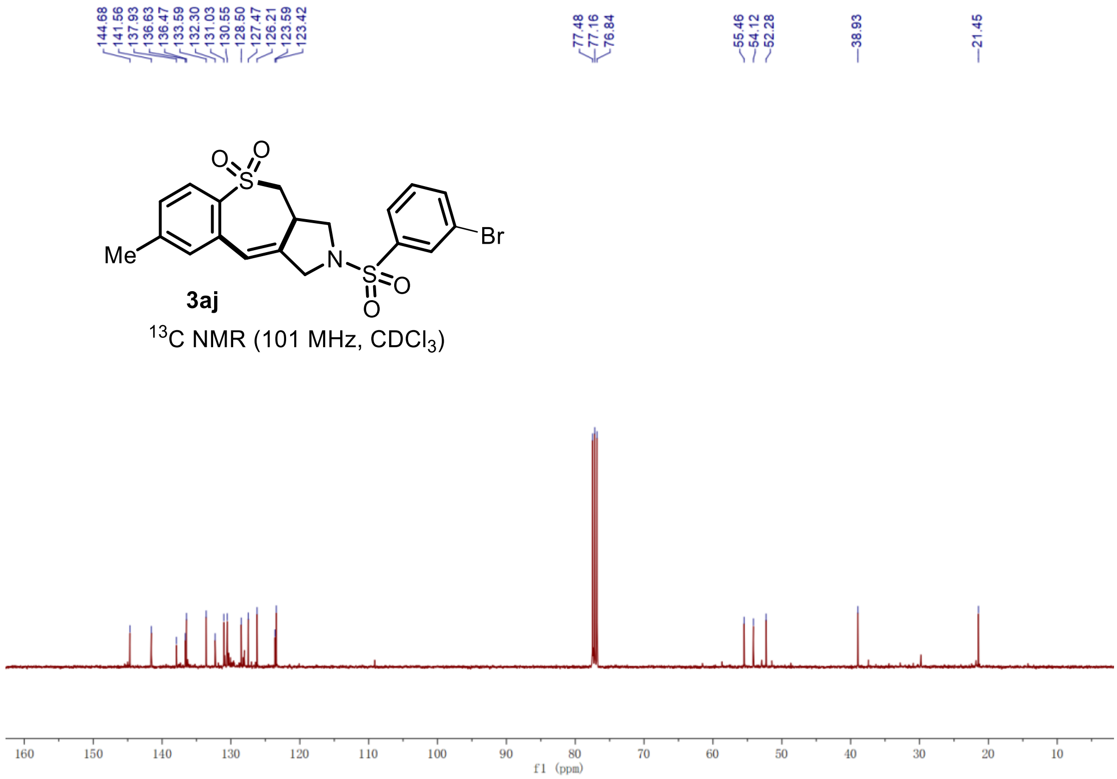

3aj

^{13}C NMR (101 MHz, $CDCl_3$)

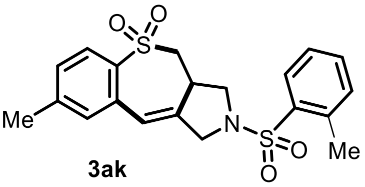

3ak

^{1}H NMR (400 MHz, $CDCl_3$)

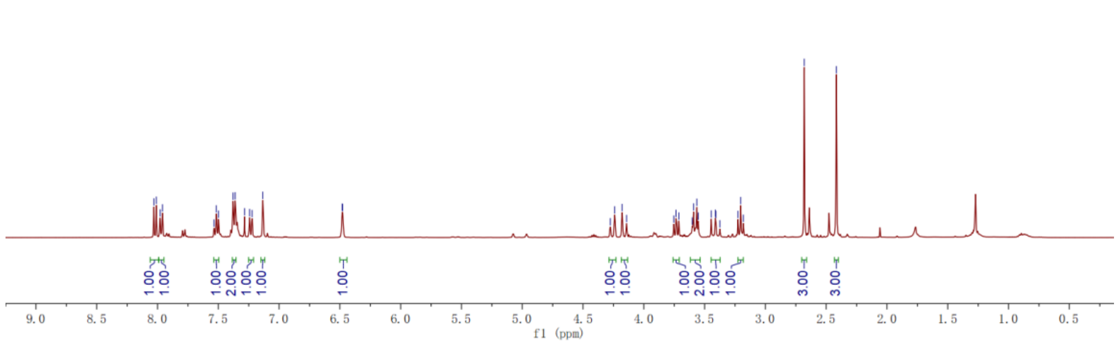

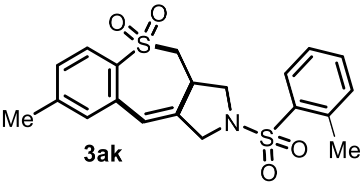

3ak

^{13}C NMR (151 MHz, $CDCl_3$)

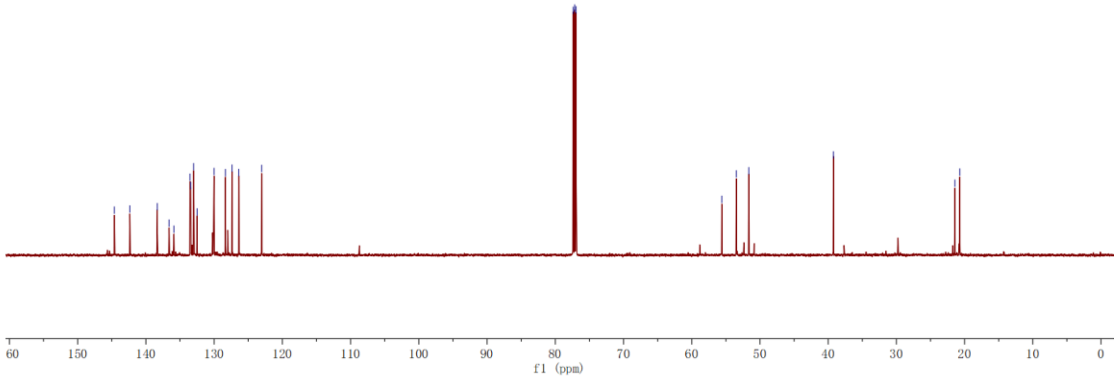

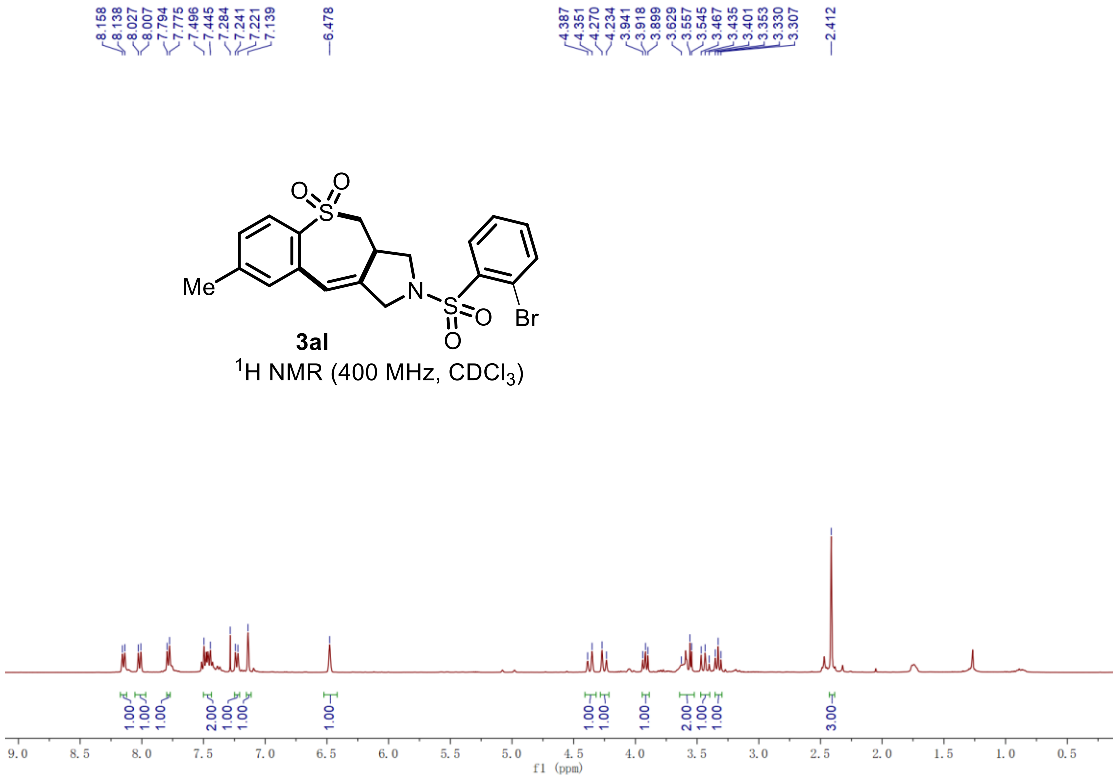
8.158
8.138
8.027
8.007
7.794
7.775
7.496
7.445
7.284
7.241
7.221
7.139
6.478
4.387
4.351
4.270
4.234
3.941
3.918
3.899
3.629
3.557
3.545
3.467
3.435
3.401
3.353
3.330
3.307
2.412
Me
Br
3al
^{1}H NMR (400 MHz, $CDCl_3$)
f1 (ppm)

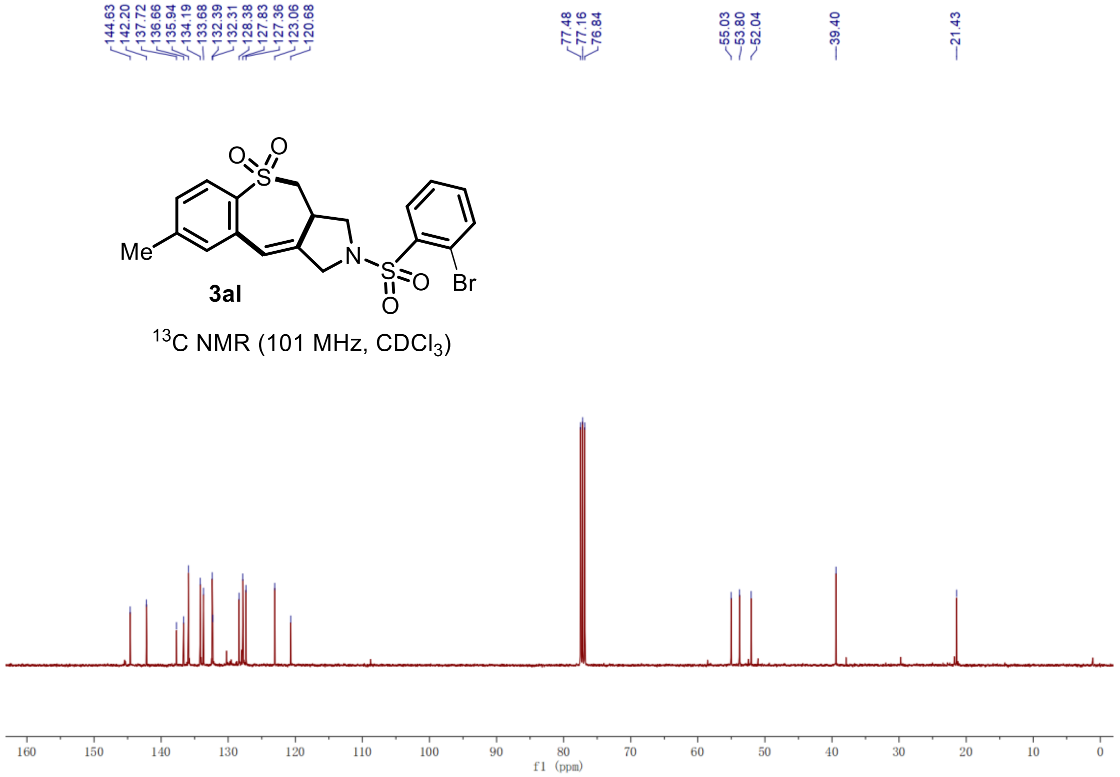
144.63
142.20
137.72
136.66
135.94
134.19
133.68
132.39
132.31
128.38
127.83
127.36
123.06
120.68
77.48
77.16
76.84
55.03
53.80
52.04
39.40
21.43
Me
Br
3al
^{13}C NMR (101 MHz, $CDCl_3$)
f1 (ppm)

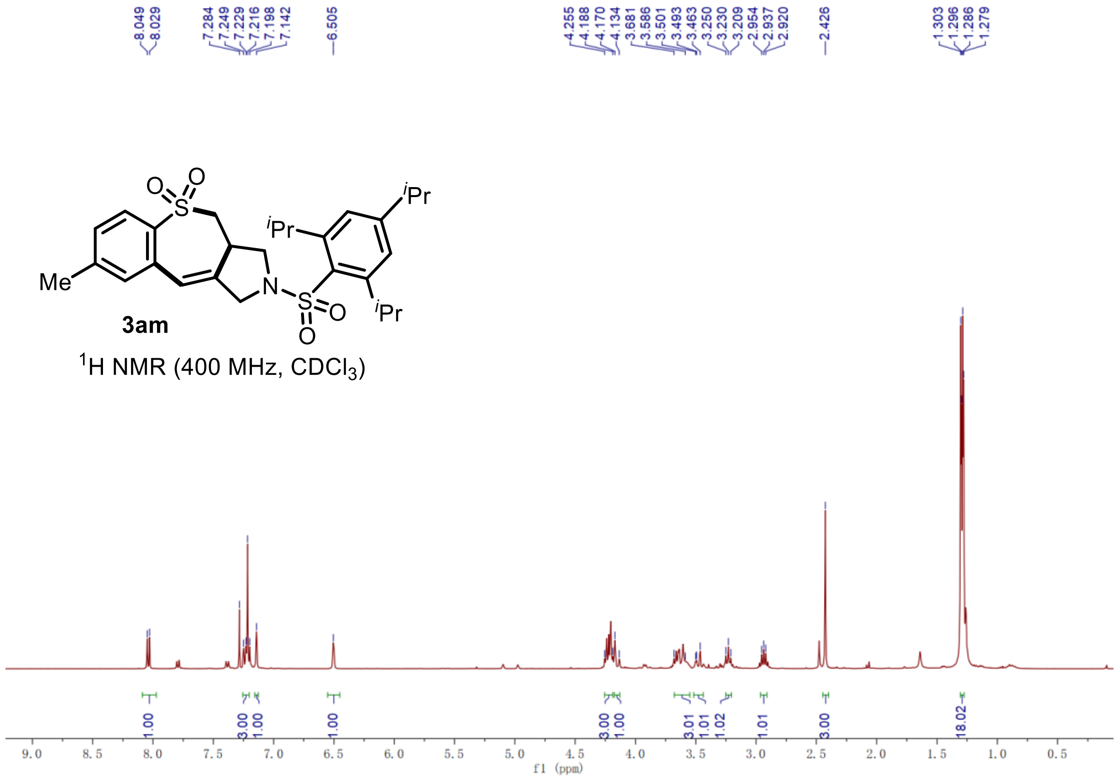

8.049
8.029
7.284
7.249
7.229
7.216
7.198
7.142
6.505
4.255
4.188
4.170
4.134
3.681
3.586
3.501
3.493
3.463
3.250
3.230
3.209
2.954
2.937
2.920
2.426
1.303
1.296
1.286
1.279
Me
iPr
iPr
iPr
3am
1H NMR (400 MHz, CDCl3)
1.00
3.00
1.00
1.00
3.00
1.00
3.01
1.01
1.02
1.01
3.00
18.02
f1 (ppm)

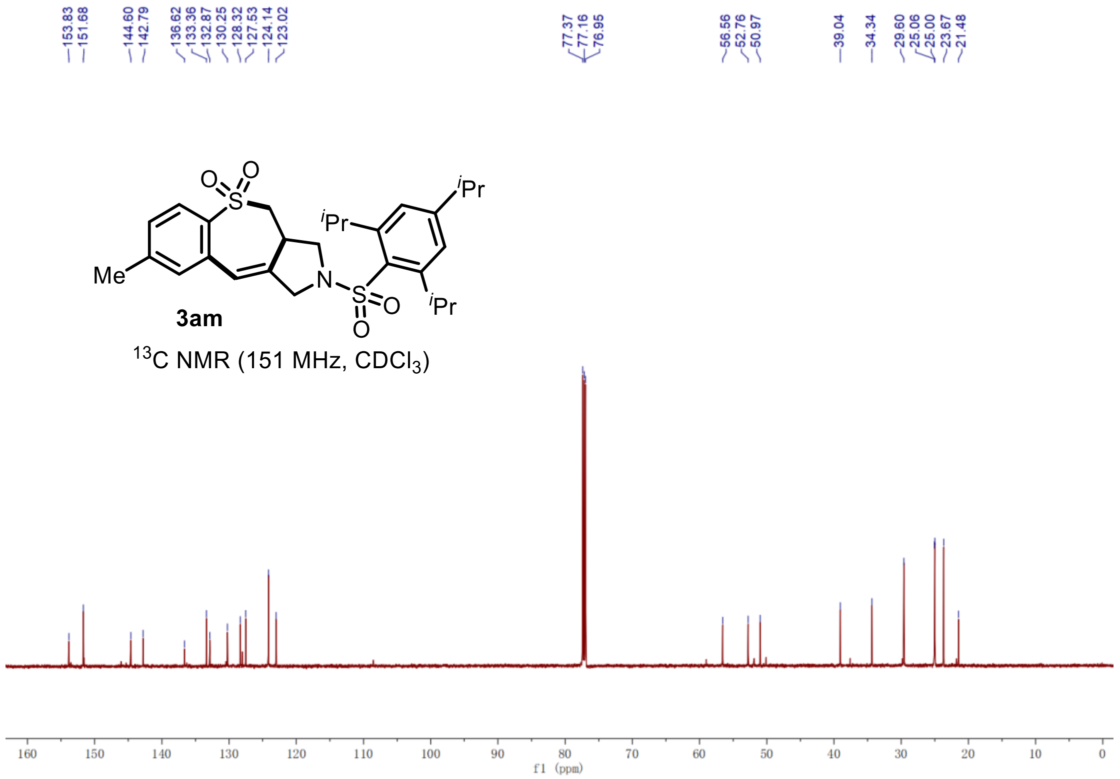

153.83
151.68
144.60
142.79
136.62
133.36
132.87
130.25
128.32
127.53
124.14
123.02
77.37
77.16
76.95
56.56
52.76
50.97
39.04
34.34
29.60
25.06
25.00
23.67
21.48
Me
iPr
iPr
iPr
3am
13C NMR (151 MHz, CDCl3)
f1 (ppm)

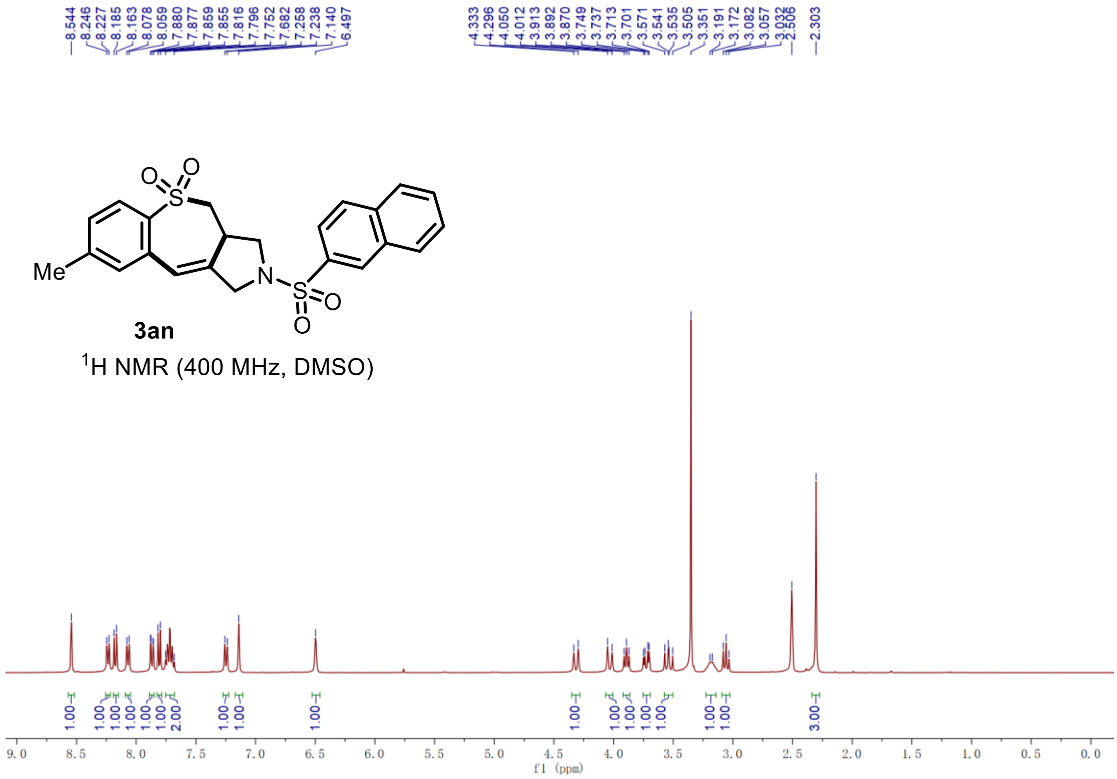
8.544
8.246
8.227
8.185
8.163
8.078
8.059
7.880
7.877
7.859
7.855
7.816
7.796
7.752
7.682
7.258
7.238
7.140
6.497
4.333
4.296
4.050
4.012
3.913
3.892
3.870
3.749
3.737
3.713
3.701
3.571
3.541
3.535
3.505
3.351
3.191
3.172
3.082
3.057
3.032
2.506
2.303
Me
N
S
O
O
O
O
S
3an
1H NMR (400 MHz, DMSO)
f1 (ppm)

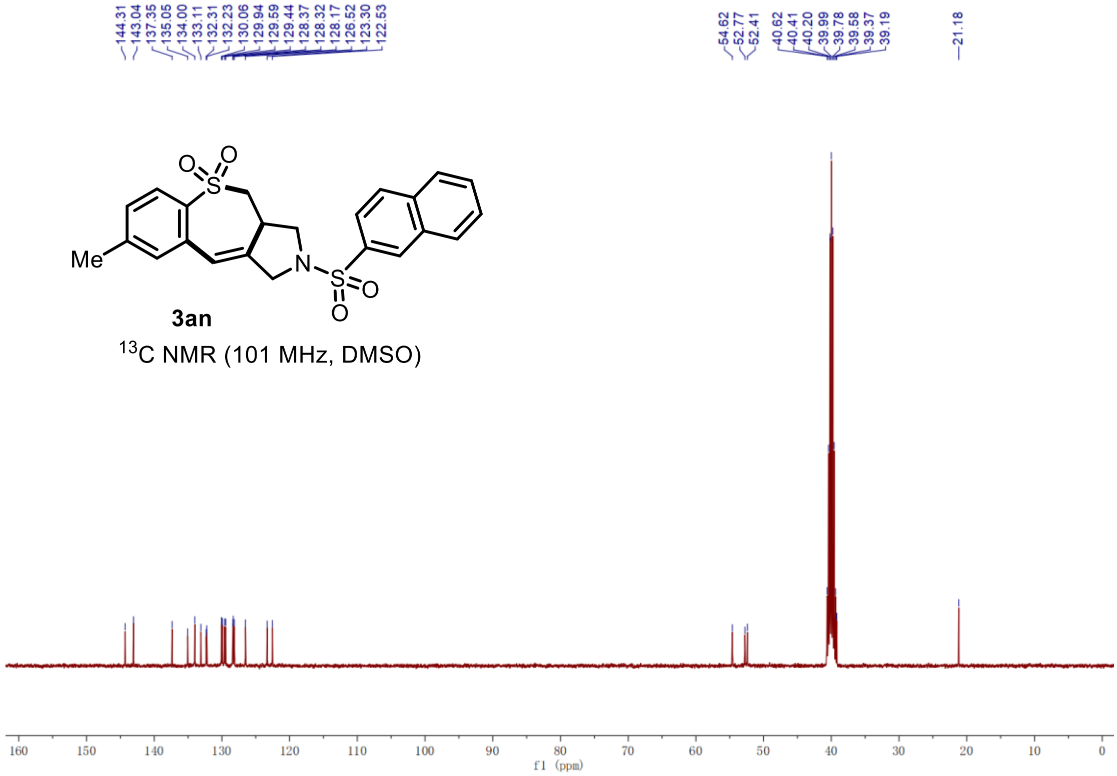
144.31
143.04
137.35
135.05
134.00
133.11
132.31
132.23
130.06
129.94
129.59
129.44
128.37
128.32
128.17
126.52
123.30
122.53
54.62
52.77
52.41
40.62
40.41
40.20
39.99
39.78
39.58
39.37
39.19
21.18
Me
N
S
O
O
O
O
S
3an
13C NMR (101 MHz, DMSO)
f1 (ppm)

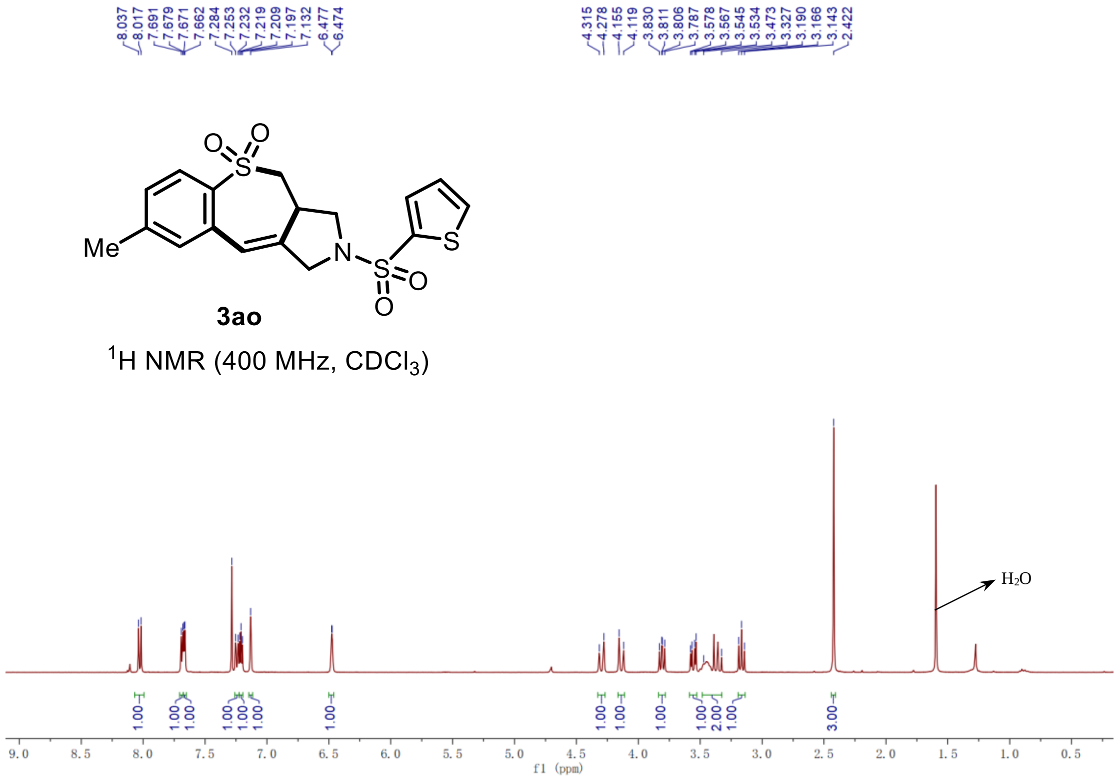

3ao

^{1}H NMR (400 MHz, $CDCl_3$)

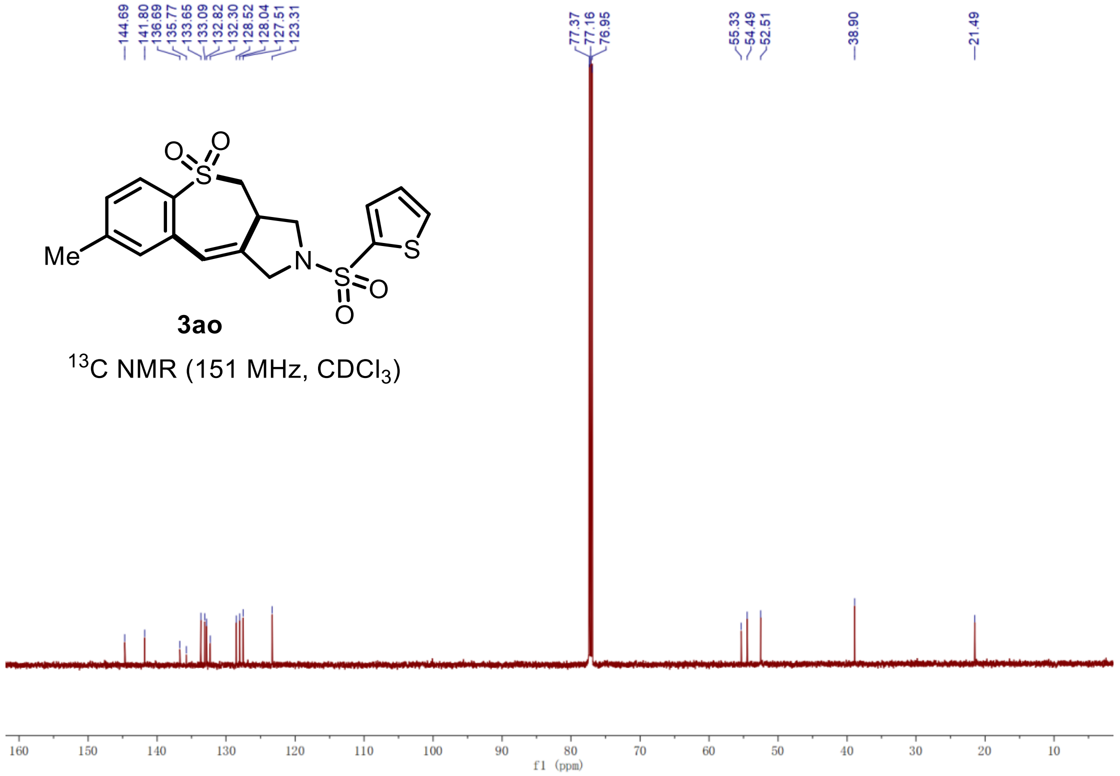

3ao

^{13}C NMR (151 MHz, $CDCl_3$)

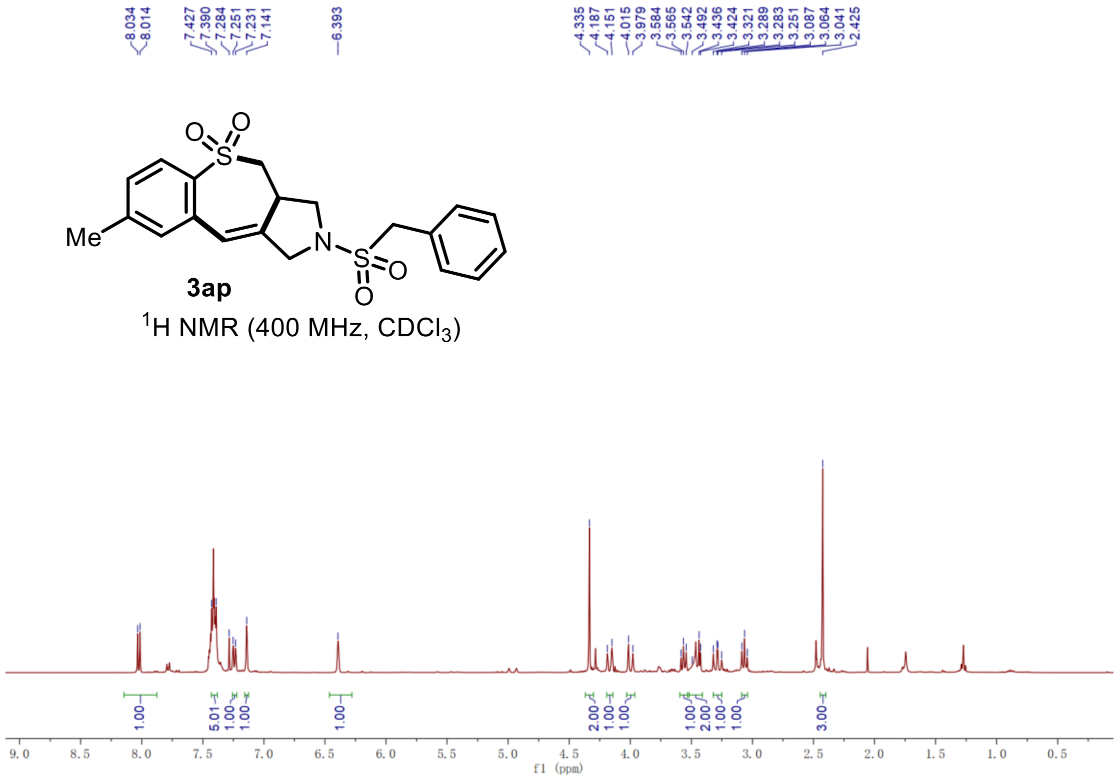

3ap

^{1}H NMR (400 MHz, $CDCl_3$)

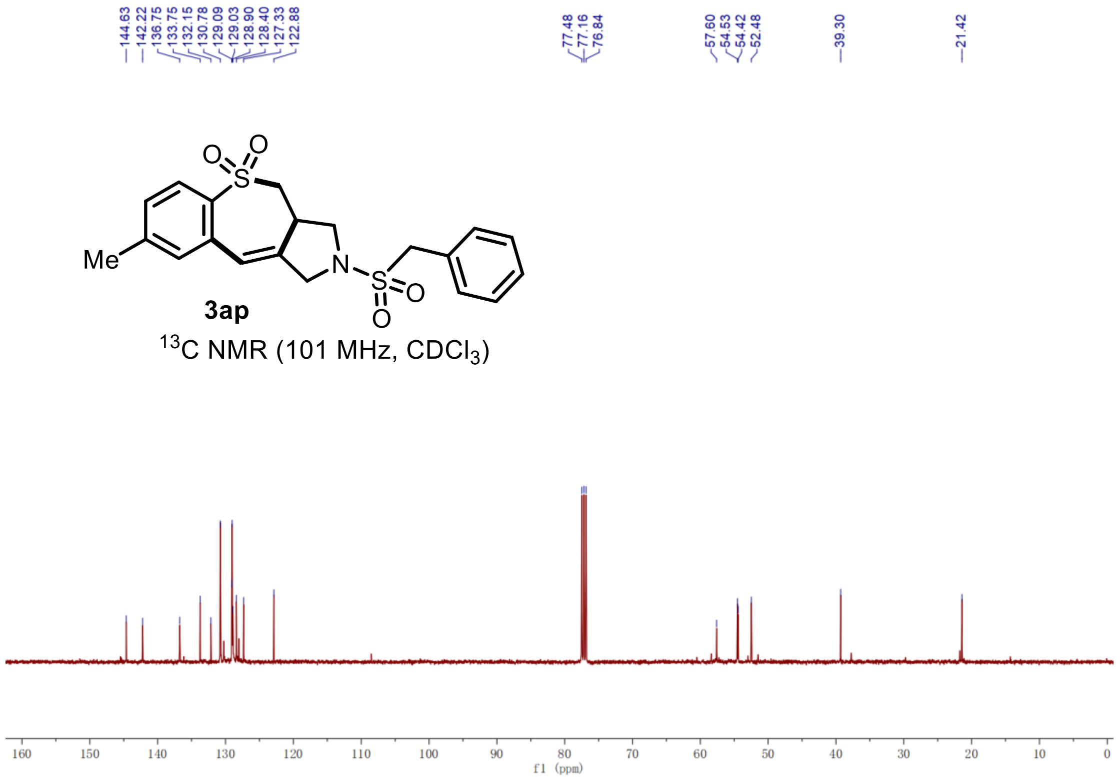

3ap

^{13}C NMR (101 MHz, $CDCl_3$)

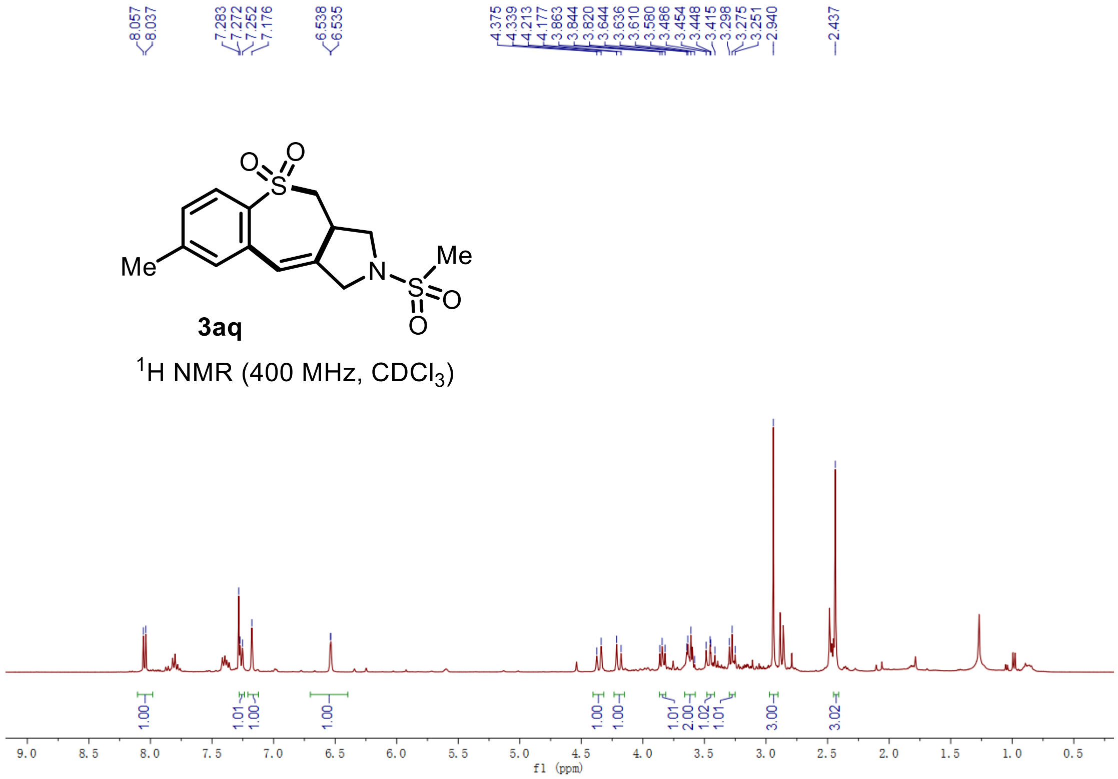
8.057
8.037
7.283
7.272
7.252
7.176
6.538
6.535
4.375
4.339
4.213
4.177
3.863
3.844
3.820
3.644
3.636
3.610
3.580
3.486
3.454
3.448
3.415
3.298
3.275
3.251
2.940
2.437
Me
Me
3aq
^{1}H NMR (400 MHz, $CDCl_3$)
1.00
1.01
1.00
1.00
1.00
1.00
1.01
2.00
1.02
1.01
3.00
3.02
f1 (ppm)

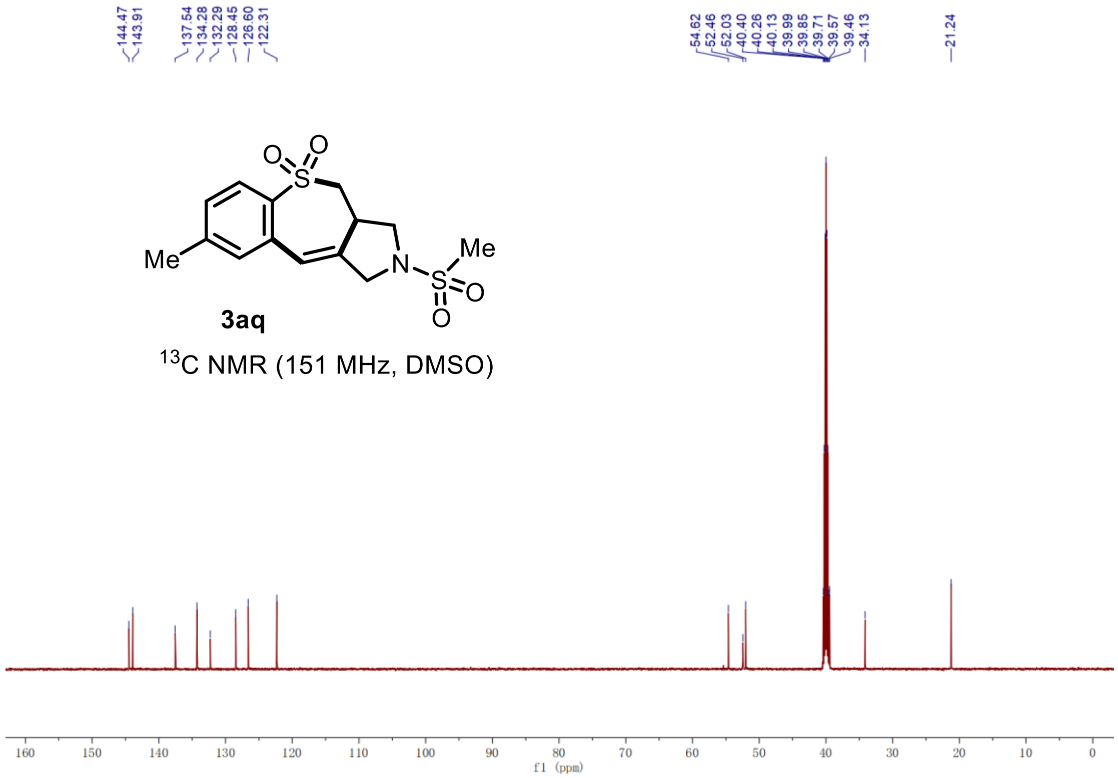
144.47
143.91
137.54
134.28
132.29
128.45
126.60
122.31
54.62
52.46
52.03
40.40
40.26
40.13
39.99
39.85
39.71
39.57
39.46
34.13
21.24
Me
Me
3aq
^{13}C NMR (151 MHz, DMSO)
f1 (ppm)

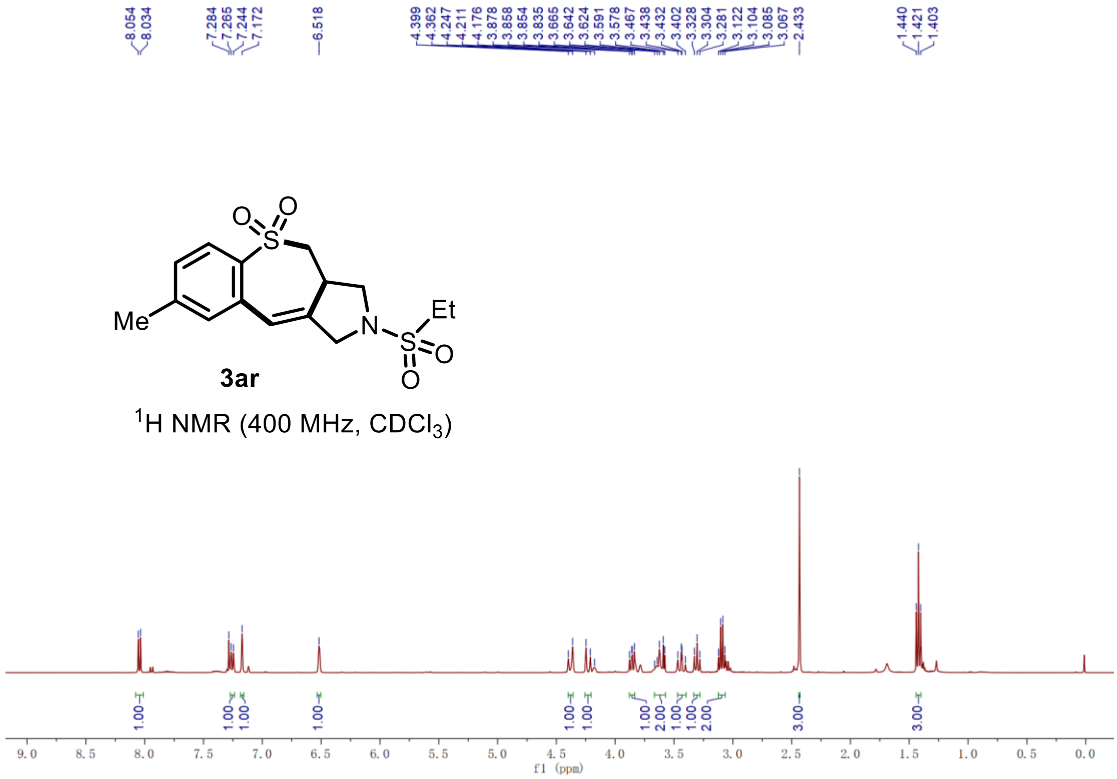

3ar

^{1}H NMR (400 MHz, $CDCl_3$)

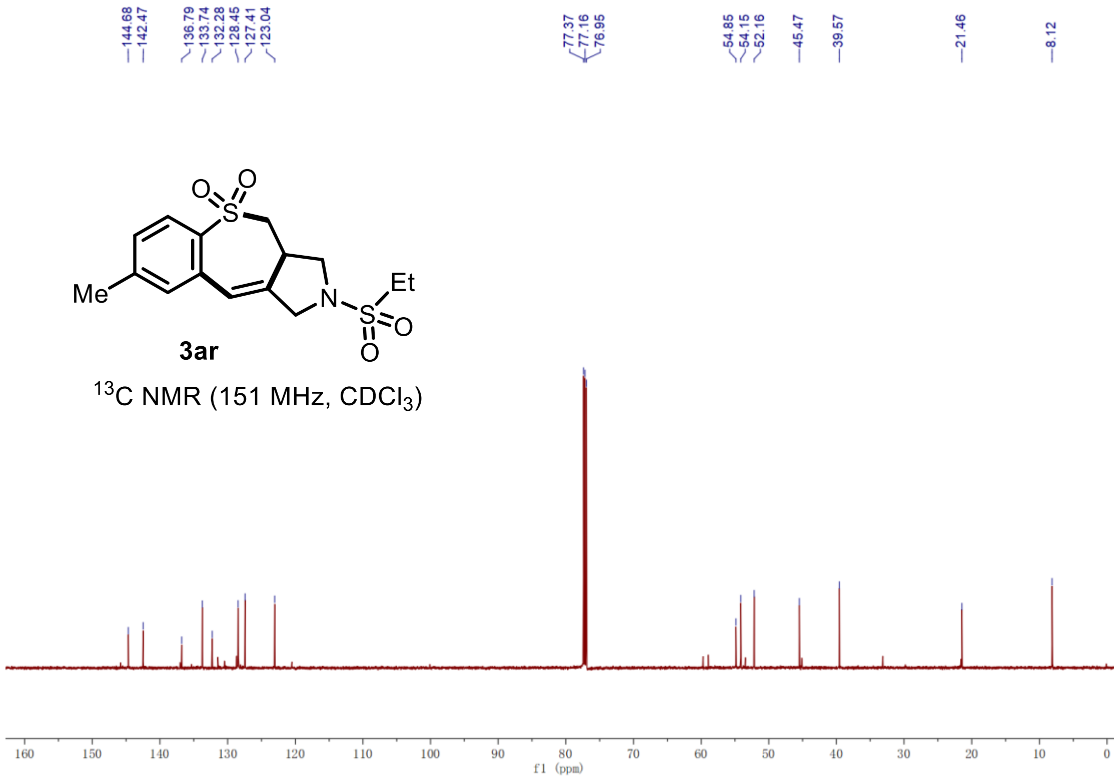

3ar

^{13}C NMR (151 MHz, $CDCl_3$)

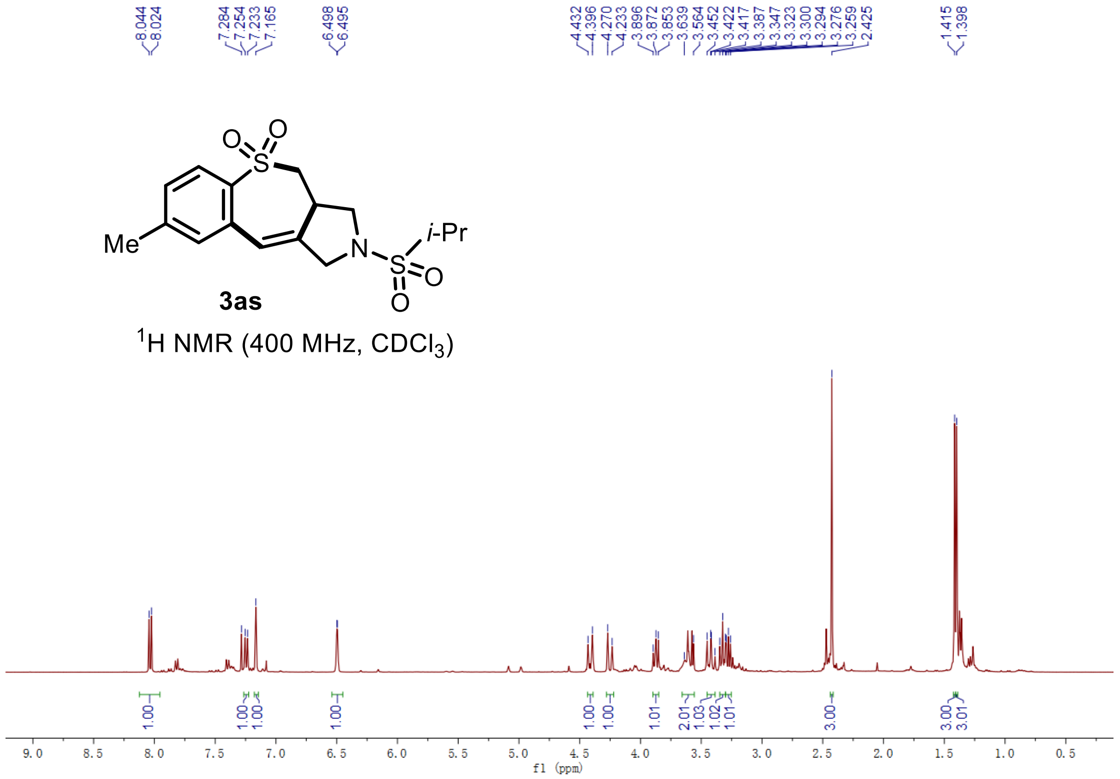
Me
i-Pr
3as
1H NMR (400 MHz, CDCl3)
8.044
8.024
7.284
7.254
7.233
7.165
6.498
6.495
4.432
4.396
4.270
4.233
3.896
3.872
3.853
3.639
3.564
3.452
3.422
3.417
3.387
3.347
3.323
3.300
3.294
3.276
3.259
2.425
1.415
1.398
f1 (ppm)

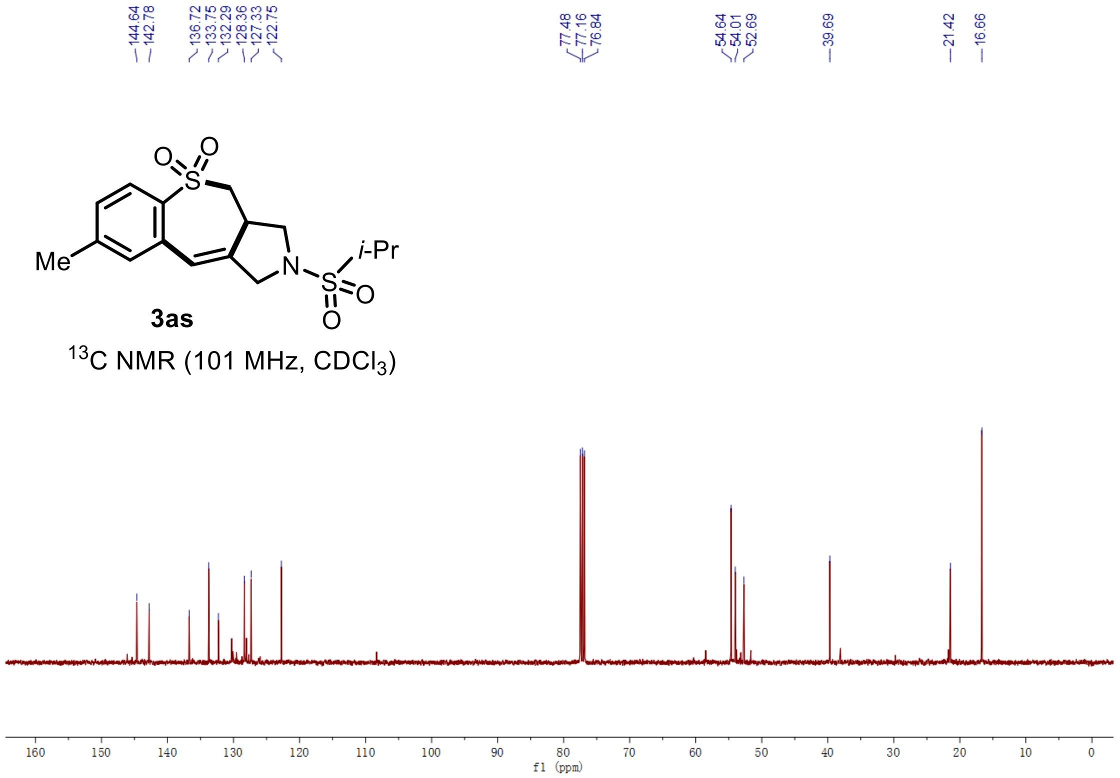
Me
i-Pr
3as
13C NMR (101 MHz, CDCl3)
144.64
142.78
136.72
133.75
132.29
128.36
127.33
122.75
77.48
77.16
76.84
54.64
54.01
52.69
39.69
21.42
16.66
f1 (ppm)

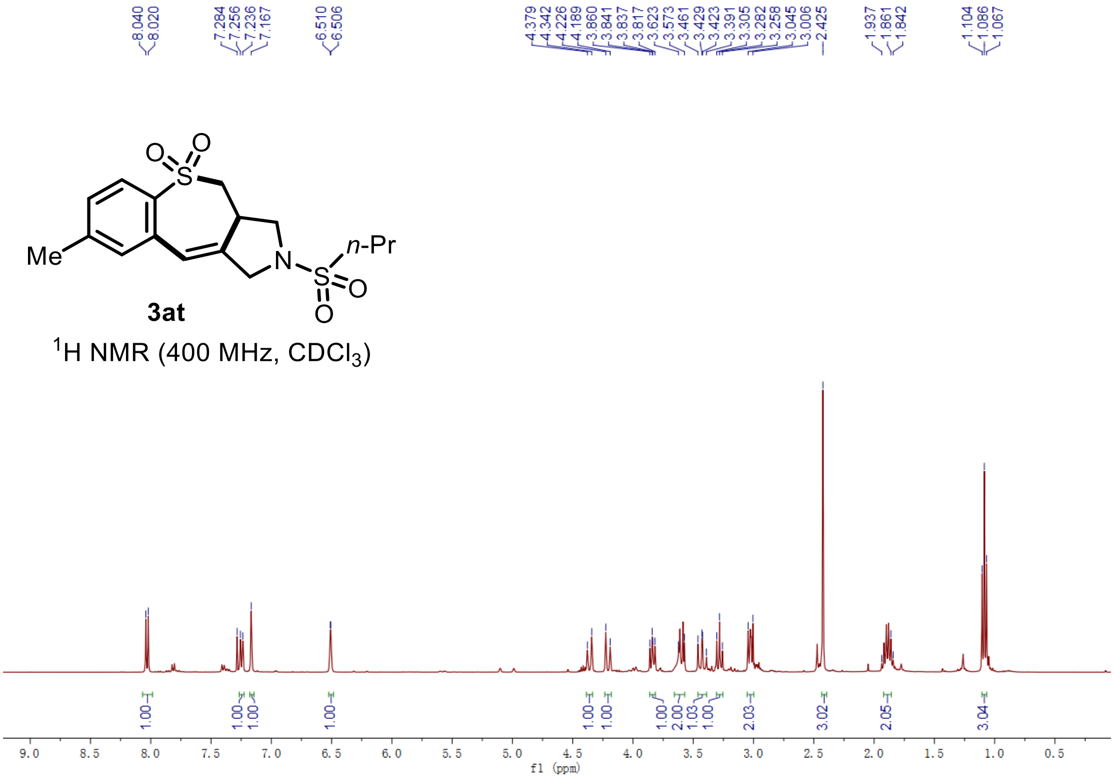
8.040
8.020
7.284
7.256
7.236
7.167
6.510
6.506
4.379
4.342
4.226
4.189
3.860
3.841
3.837
3.817
3.623
3.573
3.461
3.429
3.423
3.391
3.305
3.282
3.258
3.045
3.006
2.425
1.937
1.861
1.842
1.104
1.086
1.067
Me
n-Pr
3at
1H NMR (400 MHz, CDCl3)
1.00
1.00
1.00
1.00
1.00
1.00
1.00
2.00
1.03
1.00
2.03
3.02
2.05
3.04
f1 (ppm)

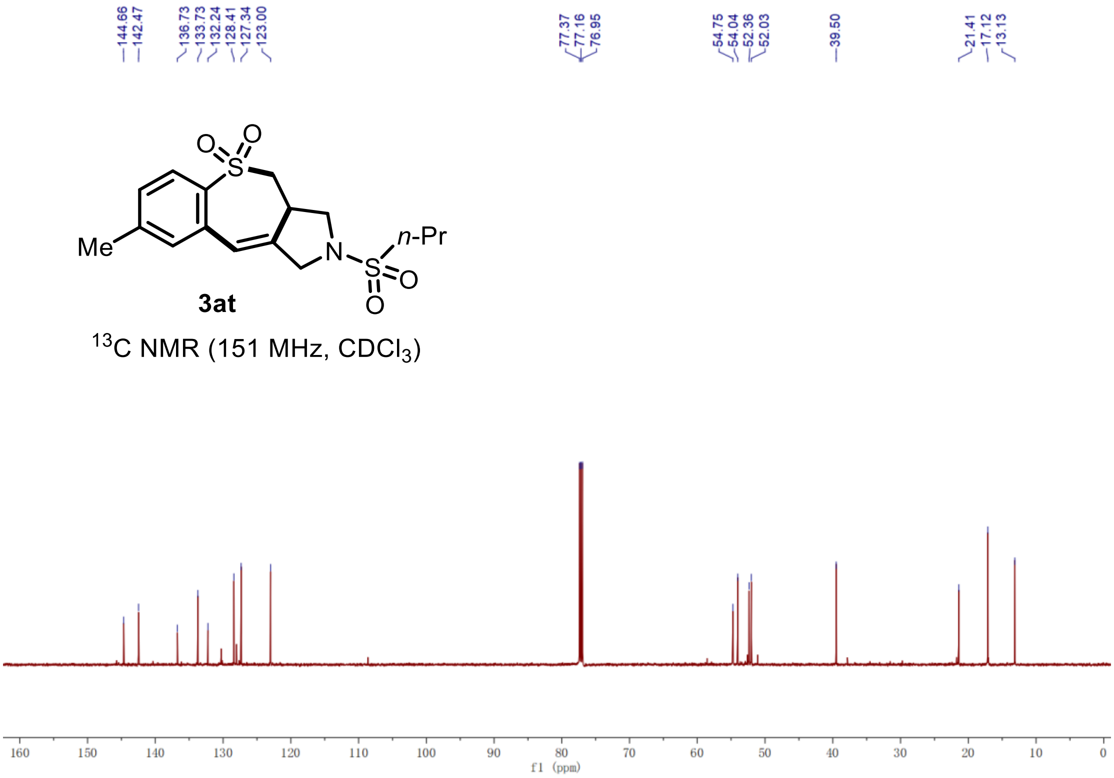
144.66
142.47
136.73
133.73
132.24
128.41
127.34
123.00
77.37
77.16
76.95
54.75
54.04
52.36
52.03
39.50
21.41
17.12
13.13
Me
n-Pr
3at
13C NMR (151 MHz, CDCl3)
f1 (ppm)

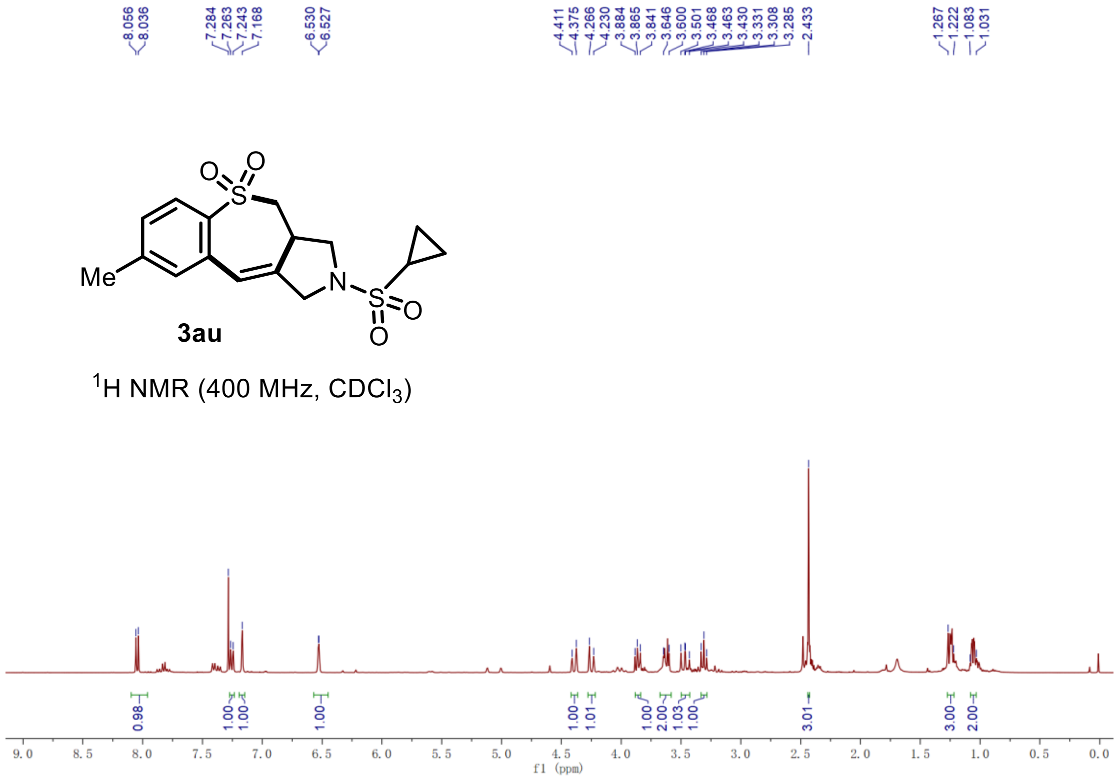
8.056
8.036
7.284
7.263
7.243
7.168
6.530
6.527
4.411
4.375
4.266
4.230
3.884
3.865
3.841
3.646
3.600
3.501
3.468
3.463
3.430
3.331
3.308
3.285
2.433
1.267
1.222
1.083
1.031
Me
3au
^{1}H NMR (400 MHz, CDCl$_3$)
0.98
1.00
1.00
1.00
1.00
1.01
1.00
2.00
1.03
1.00
3.01
3.00
2.00
f1 (ppm)

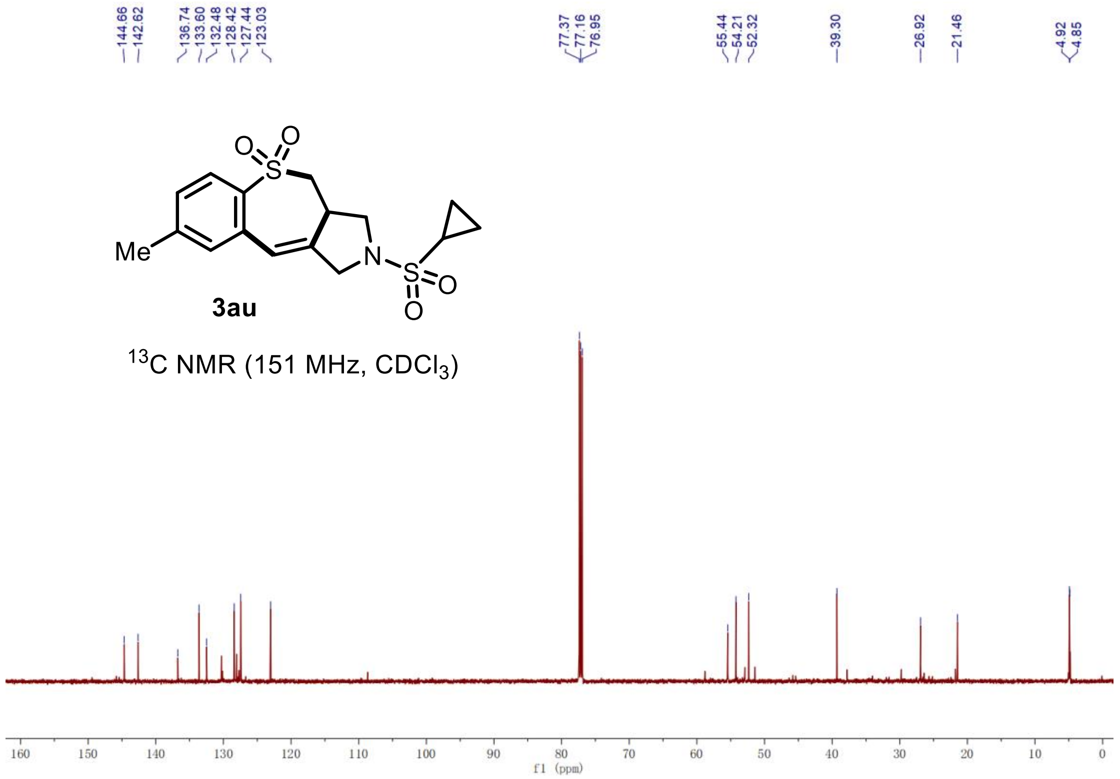
144.66
142.62
136.74
133.60
132.48
128.42
127.44
123.03
77.37
77.16
76.95
55.44
54.21
52.32
39.30
26.92
21.46
4.92
4.85
Me
3au
^{13}C NMR (151 MHz, CDCl$_3$)
f1 (ppm)

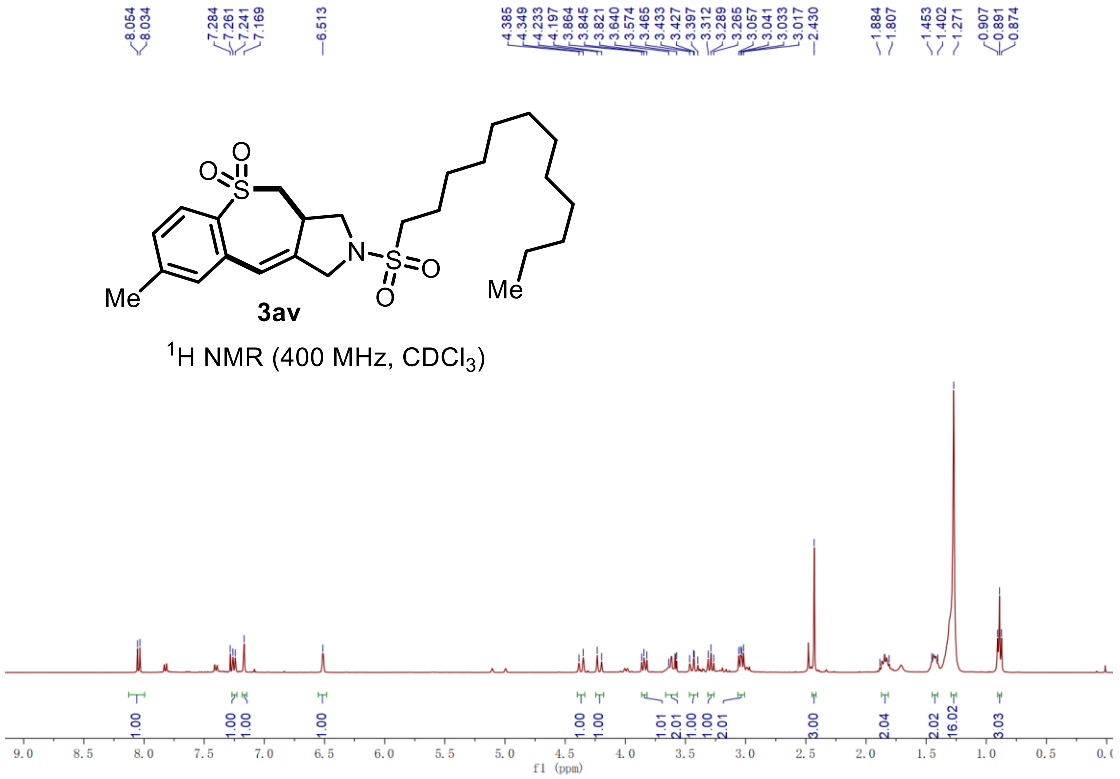

^{1}H NMR (400 MHz, $CDCl_3$)

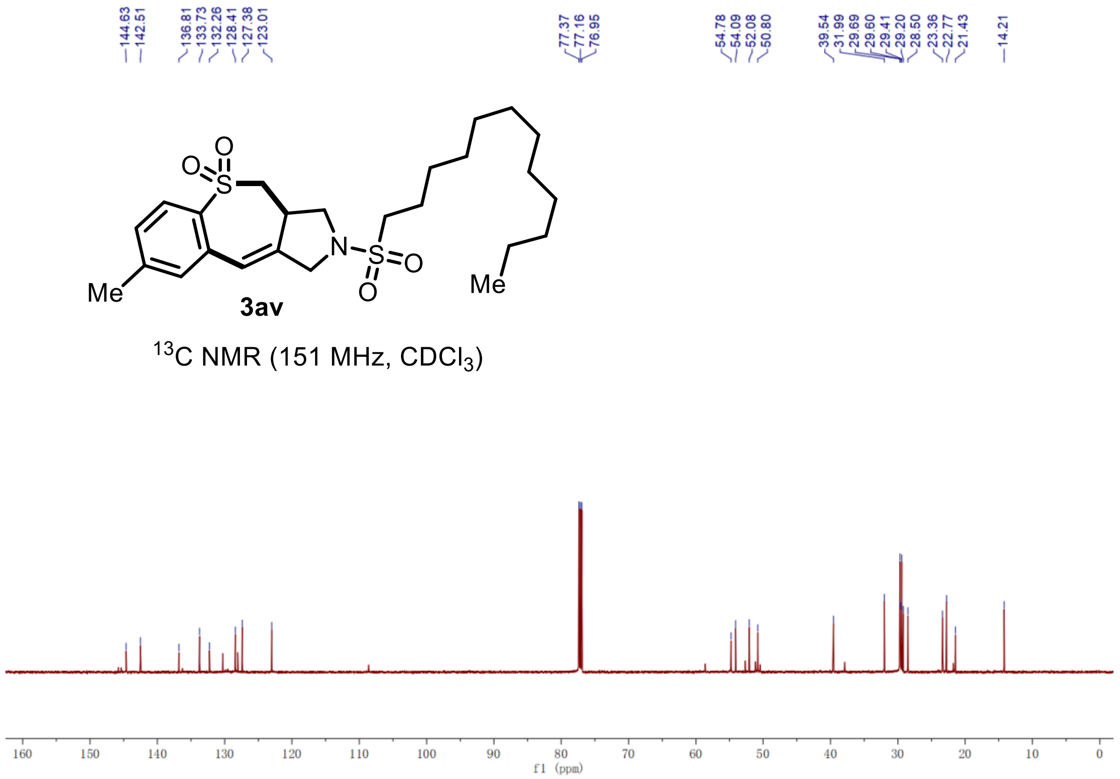

^{13}C NMR (151 MHz, $CDCl_3$)

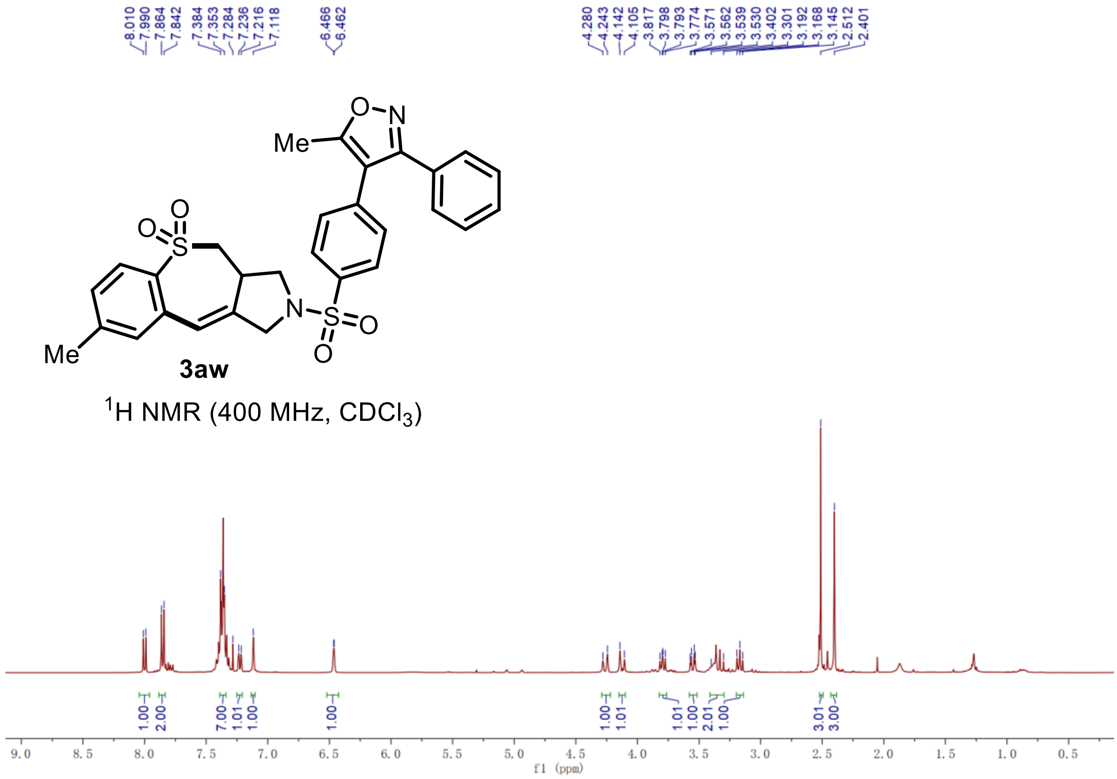
8.010 7.990 7.864 7.842 7.384 7.353 7.284 7.236 7.216 7.118 6.466 6.462 4.280 4.243 4.142 4.105 3.817 3.798 3.793 3.774 3.571 3.562 3.539 3.530 3.402 3.301 3.192 3.168 3.145 2.512 2.401
O N Me O O S N S O O Me
3aw
^{1}H NMR (400 MHz, $CDCl_3$)
1.00 2.00 7.00 1.01 1.00 1.00 1.00 1.01 1.01 1.00 2.01 1.00 3.01 3.00
9.0 8.5 8.0 7.5 7.0 6.5 6.0 5.5 5.0 4.5 4.0 3.5 3.0 2.5 2.0 1.5 1.0 0.5
f1 (ppm)

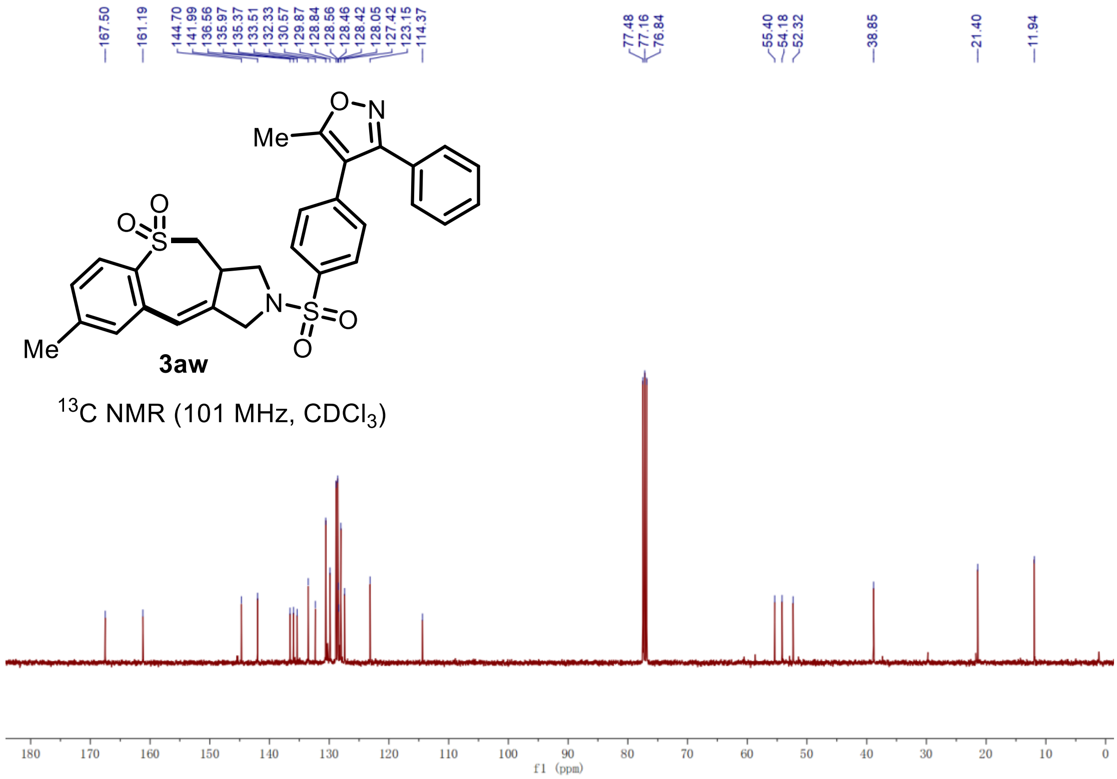
167.50 161.19 144.70 141.99 136.56 135.97 135.37 133.51 132.33 130.57 129.87 128.84 128.56 128.46 128.42 128.05 127.42 123.15 114.37 77.48 77.16 76.84 55.40 54.18 52.32 38.85 21.40 11.94
O N Me O O S N S O O Me
3aw
^{13}C NMR (101 MHz, $CDCl_3$)
180 170 160 150 140 130 120 110 100 90 80 70 60 50 40 30 20 10 0
f1 (ppm)

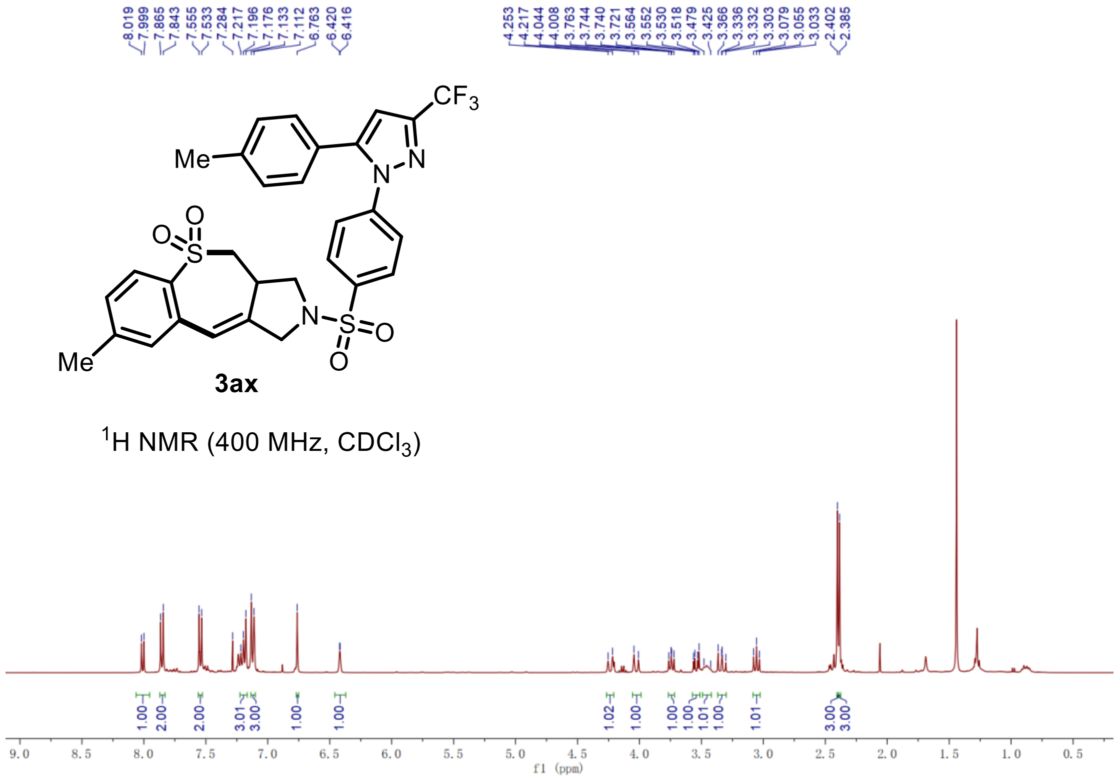
8.019
7.999
7.865
7.843
7.555
7.533
7.284
7.217
7.196
7.176
7.133
7.112
6.763
6.420
6.416
4.253
4.217
4.044
4.008
3.763
3.744
3.740
3.721
3.564
3.552
3.530
3.518
3.479
3.425
3.366
3.336
3.332
3.303
3.079
3.055
3.033
2.402
2.385
CF3
Me
N
N
O
O
S
N
S
O
O
Me
3ax
1H NMR (400 MHz, CDCl3)
1.00
2.00
2.00
3.01
3.00
1.00
1.00
1.02
1.00
1.00
1.00
1.01
1.00
1.01
3.00
3.00
9.0
8.5
8.0
7.5
7.0
6.5
6.0
5.5
5.0
4.5
4.0
3.5
3.0
2.5
2.0
1.5
1.0
0.5
f1 (ppm)

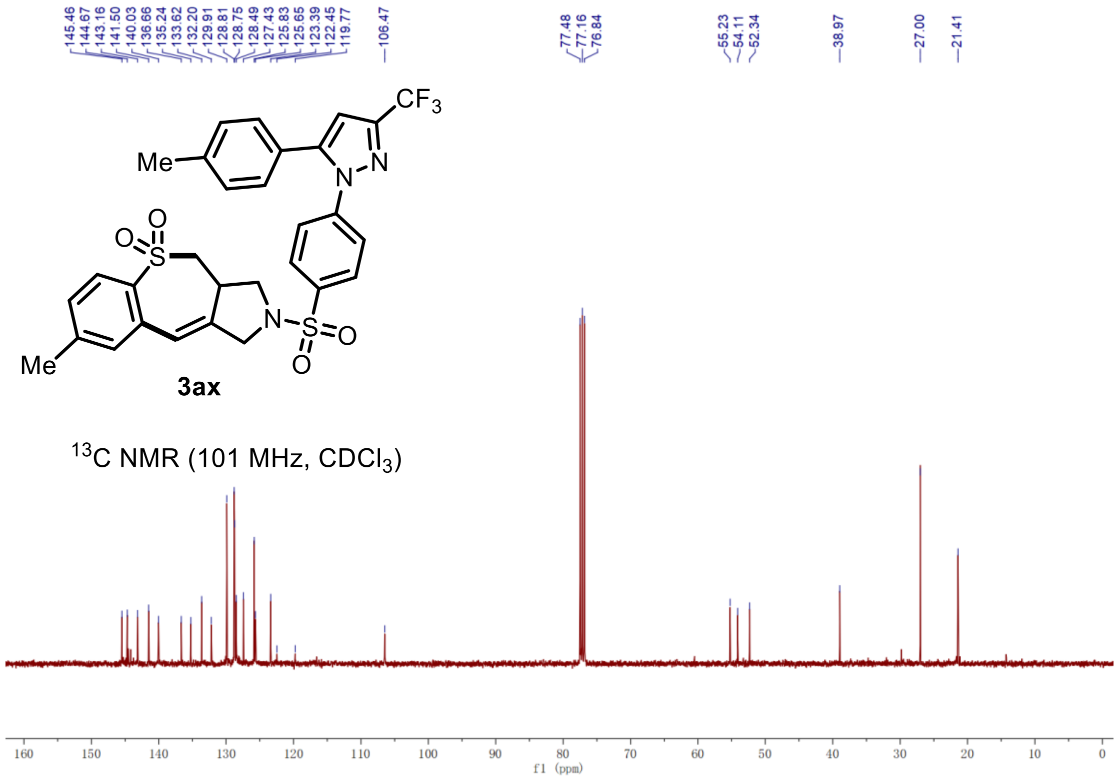
145.46
144.67
143.16
141.50
140.03
136.66
135.24
133.62
132.20
129.91
128.81
128.75
128.49
127.43
125.83
125.65
123.39
122.45
119.77
106.47
77.48
77.16
76.84
55.23
54.11
52.34
38.97
27.00
21.41
CF3
Me
N
N
O
O
S
N
S
O
O
Me
3ax
13C NMR (101 MHz, CDCl3)
160
150
140
130
120
110
100
90
80
70
60
50
40
30
20
10
0
f1 (ppm)

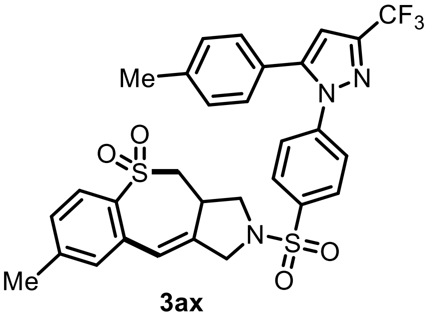

3ax

^{19}F NMR (377 MHz, $CDCl_3$)

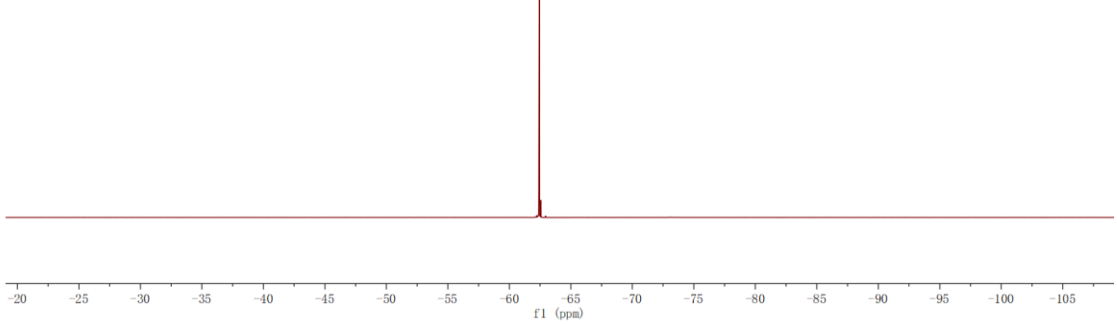

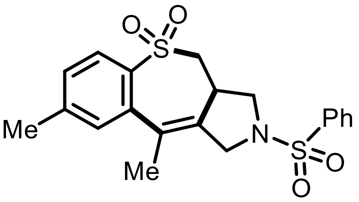

3ay

^{1}H NMR (400 MHz, $CDCl_3$)

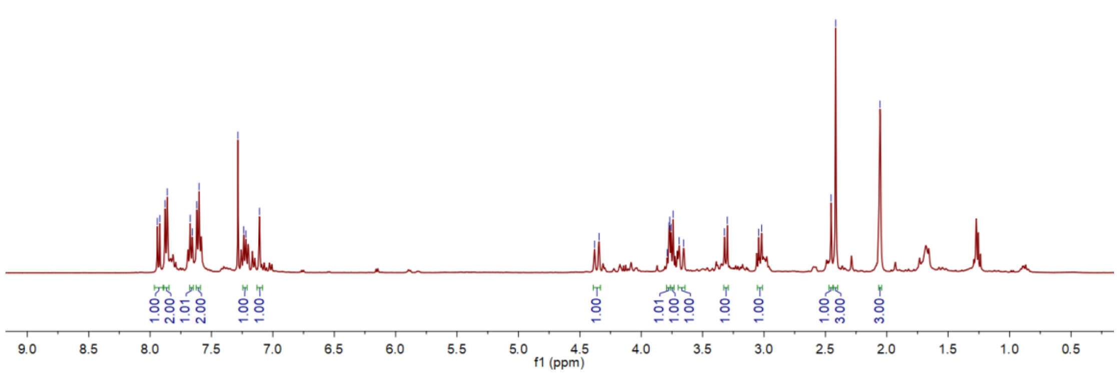

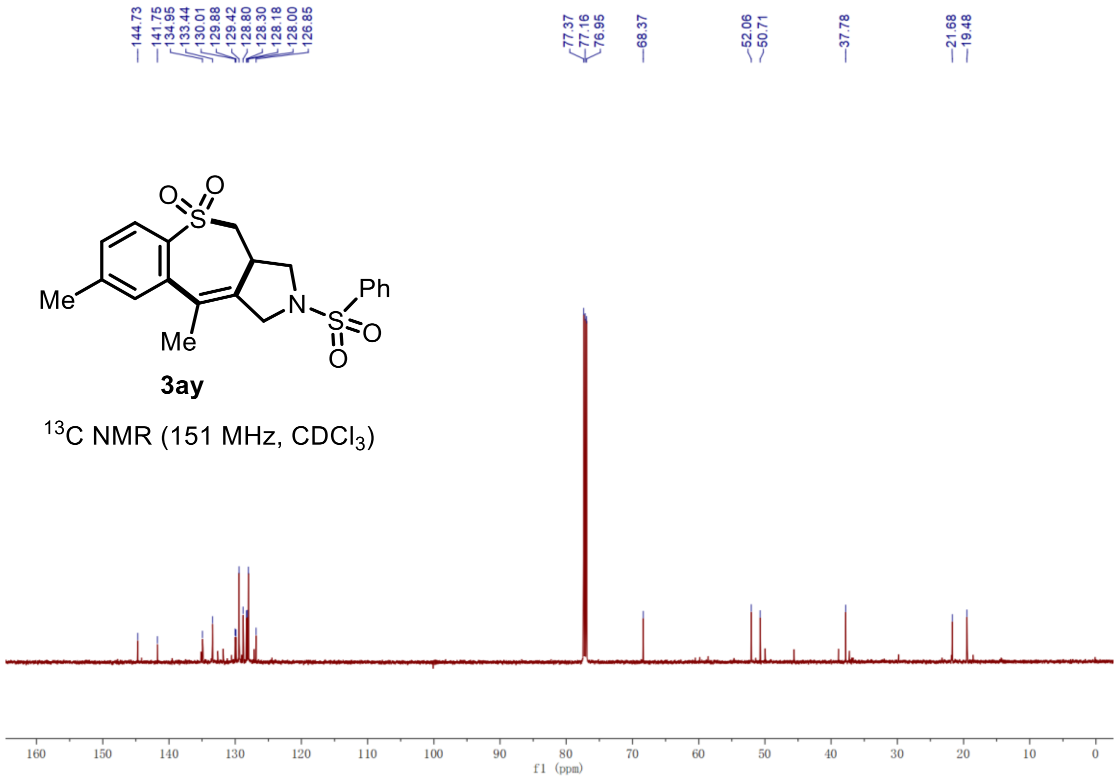

3ay

^{13}C NMR (151 MHz, $CDCl_3$)

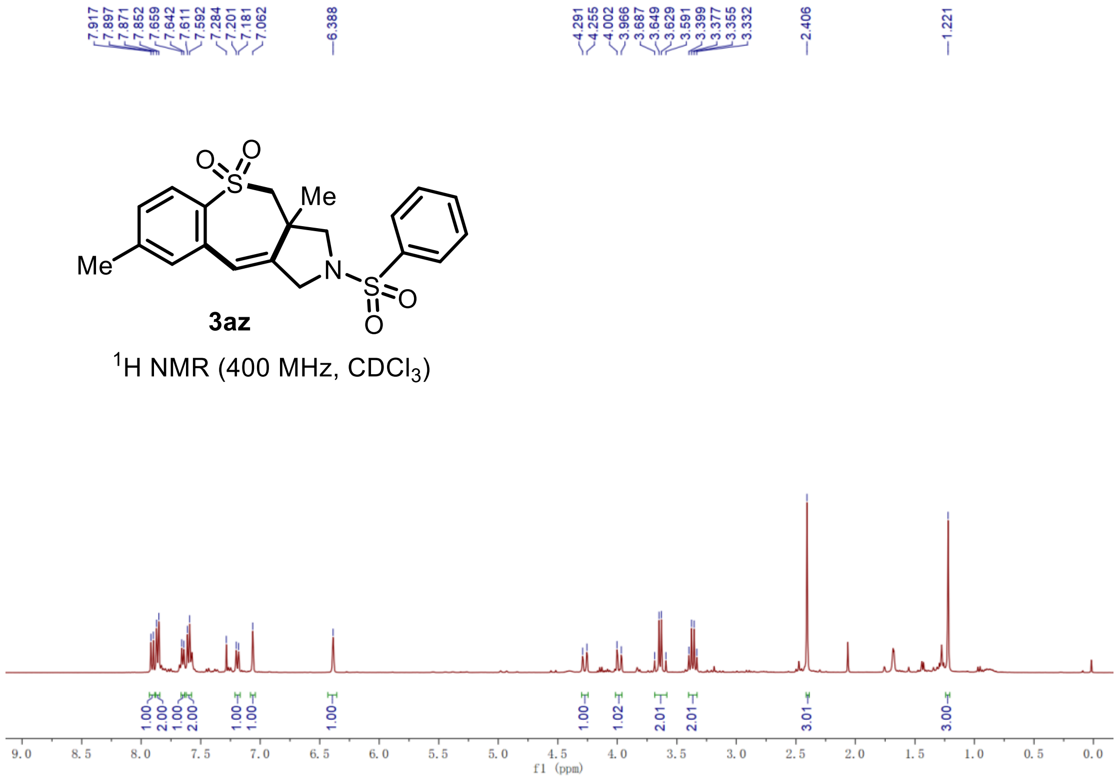

3az

^{1}H NMR (400 MHz, $CDCl_3$)

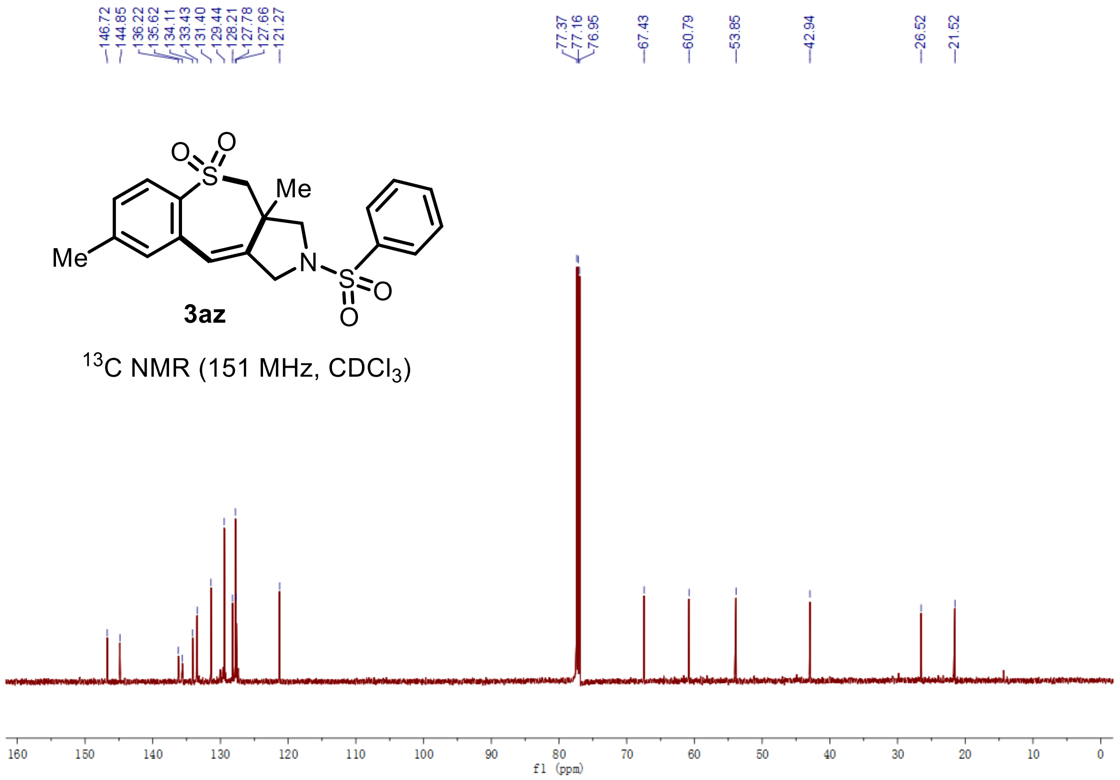
146.72
144.85
136.22
135.62
134.11
133.43
131.40
129.44
128.21
127.78
127.66
121.27
77.37
77.16
76.95
67.43
60.79
53.85
42.94
26.52
21.52
Me
Me
3az
^{13}C NMR (151 MHz, CDCl$_3$)
f1 (ppm)

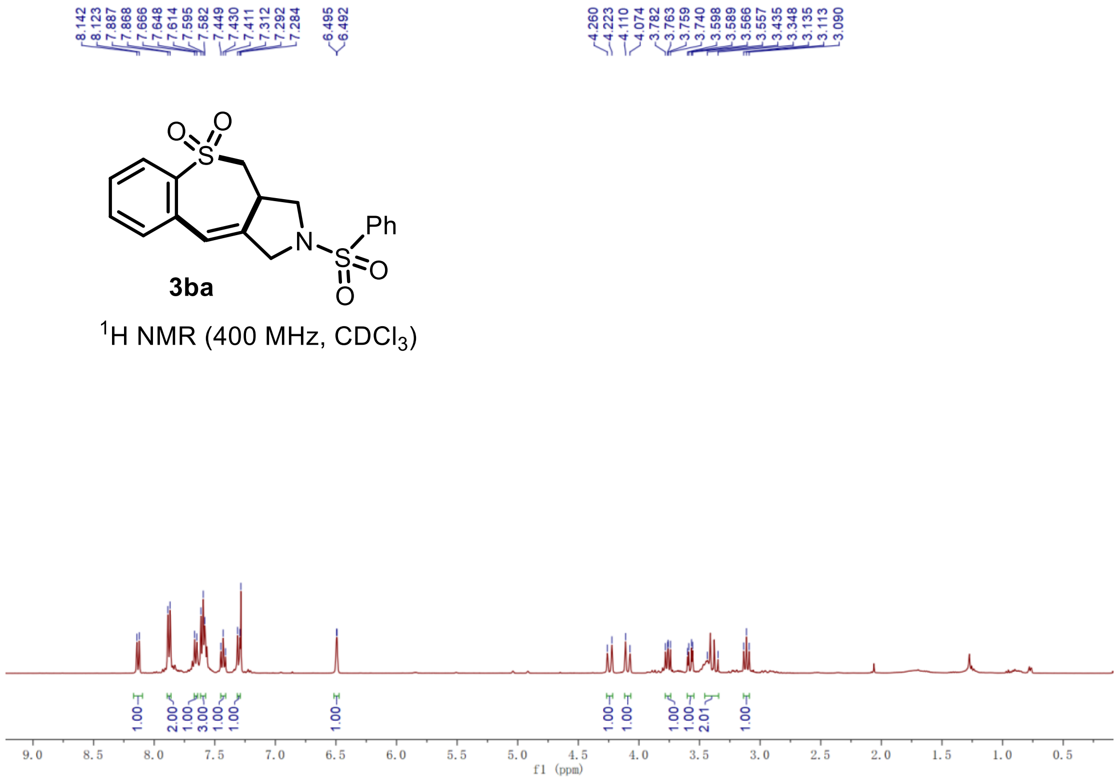
8.142
8.123
7.887
7.868
7.666
7.648
7.614
7.595
7.582
7.449
7.430
7.411
7.312
7.292
7.284
6.495
6.492
4.260
4.223
4.110
4.074
3.782
3.763
3.759
3.740
3.598
3.589
3.566
3.557
3.435
3.348
3.135
3.113
3.090
Ph
3ba
^{1}H NMR (400 MHz, CDCl$_3$)
1.00
2.00
1.00
3.00
1.00
1.00
1.00
1.00
1.00
1.00
1.00
2.01
1.00
f1 (ppm)

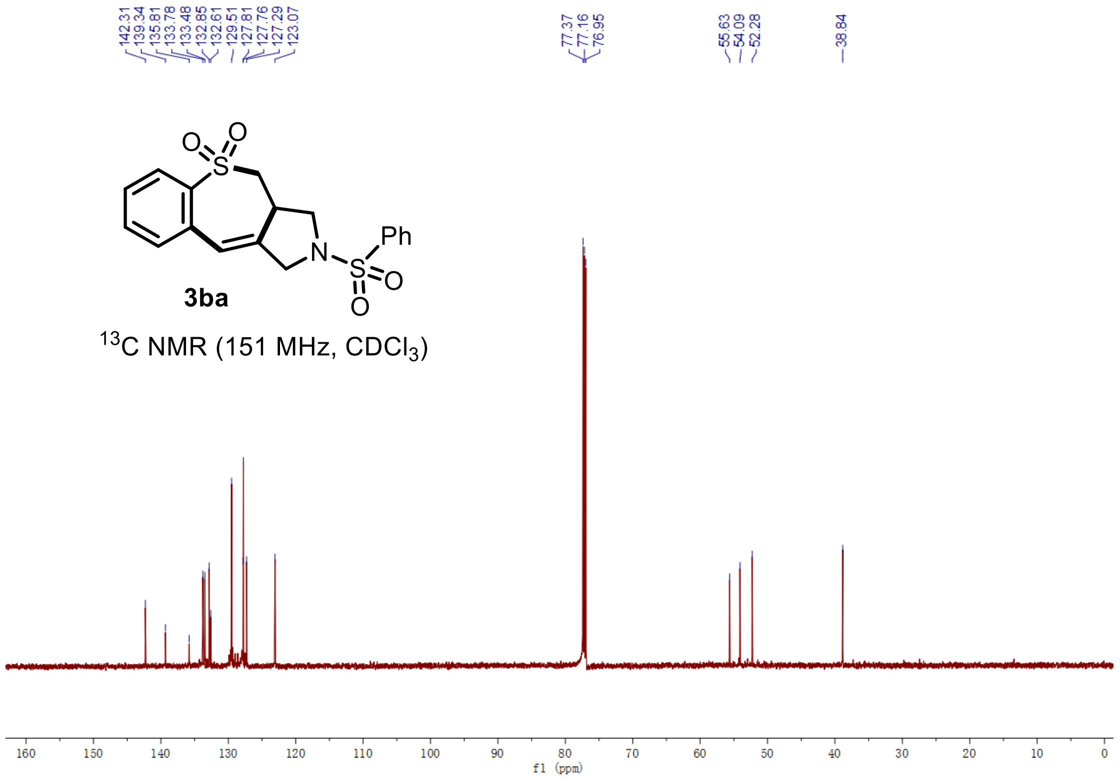
142.31
139.34
135.81
133.78
133.48
132.85
132.61
129.51
127.81
127.76
127.29
123.07
77.37
77.16
76.95
55.63
54.09
52.28
38.84
Ph
3ba
13C NMR (151 MHz, CDCl3)
f1 (ppm)

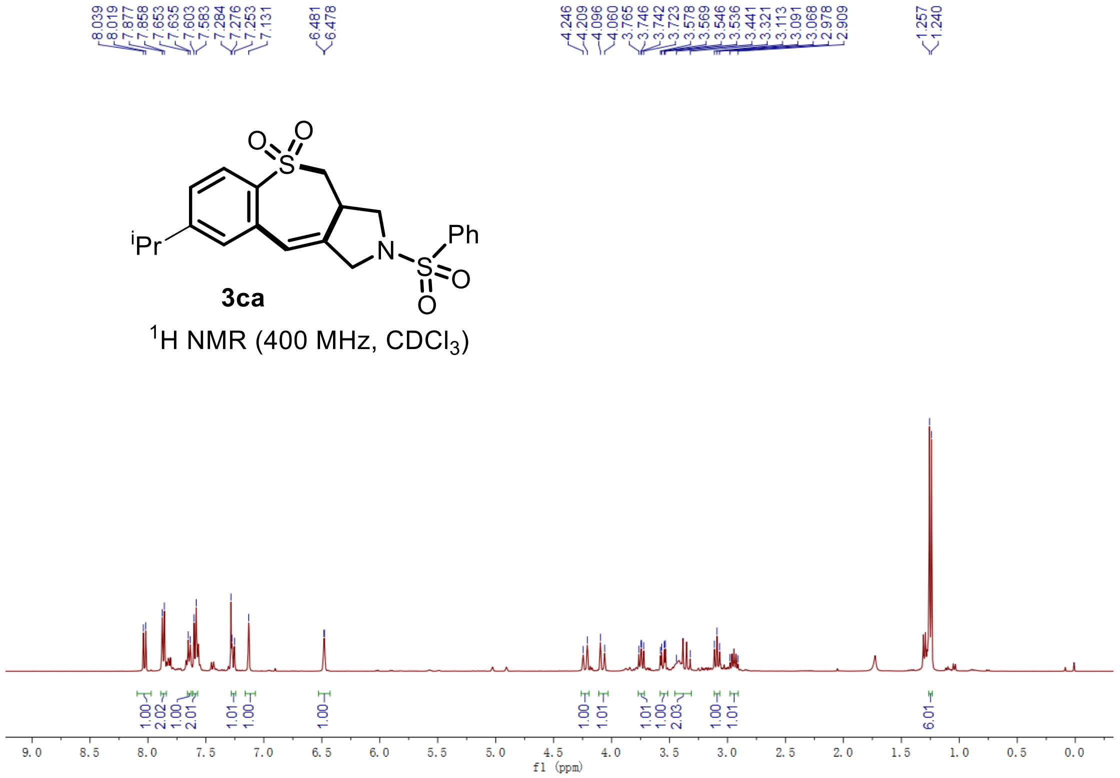
8.039
8.019
7.877
7.858
7.653
7.635
7.603
7.583
7.284
7.276
7.253
7.131
6.481
6.478
4.246
4.209
4.096
4.060
3.765
3.746
3.742
3.723
3.578
3.569
3.546
3.536
3.441
3.321
3.113
3.091
3.068
2.978
2.909
1.257
1.240
iPr
Ph
3ca
1H NMR (400 MHz, CDCl3)
1.00
2.02
1.00
2.01
1.01
1.00
1.00
1.00
1.01
1.01
1.00
2.03
1.00
1.01
6.01
f1 (ppm)

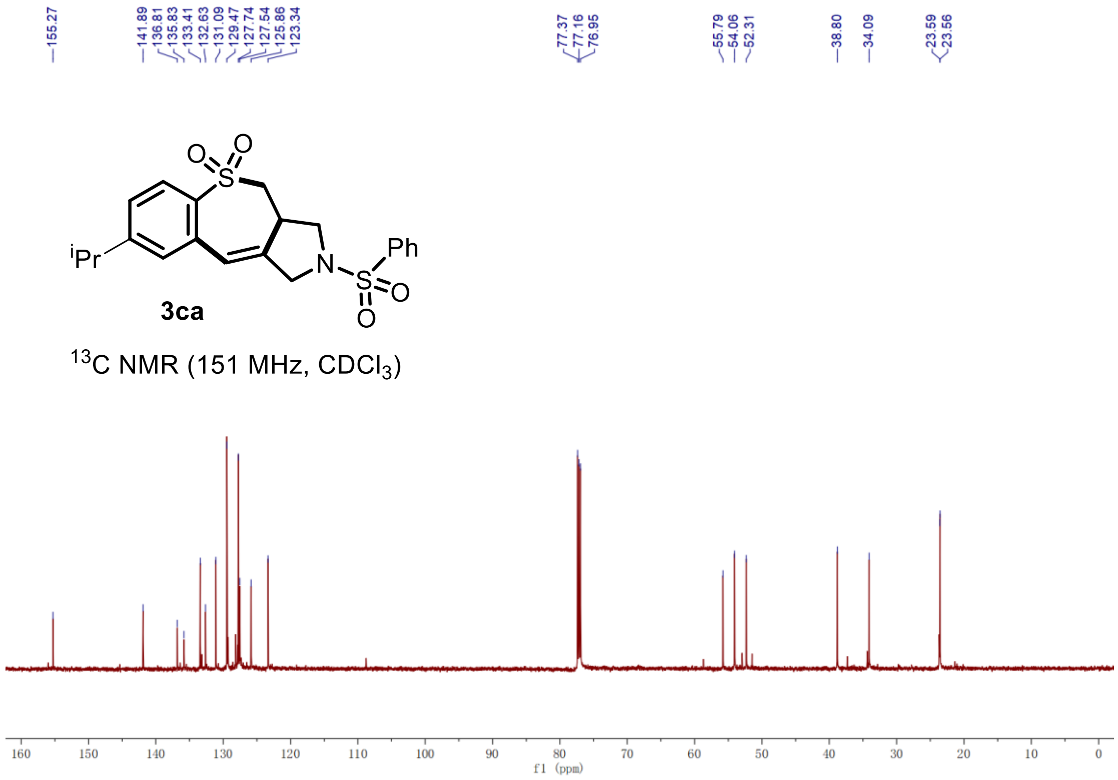
155.27
141.89
136.81
135.83
133.41
132.63
131.09
129.47
127.74
127.54
125.86
123.34
77.37
77.16
76.95
55.79
54.06
52.31
38.80
34.09
23.59
23.56
iPr
Ph
3ca
13C NMR (151 MHz, CDCl3)
f1 (ppm)

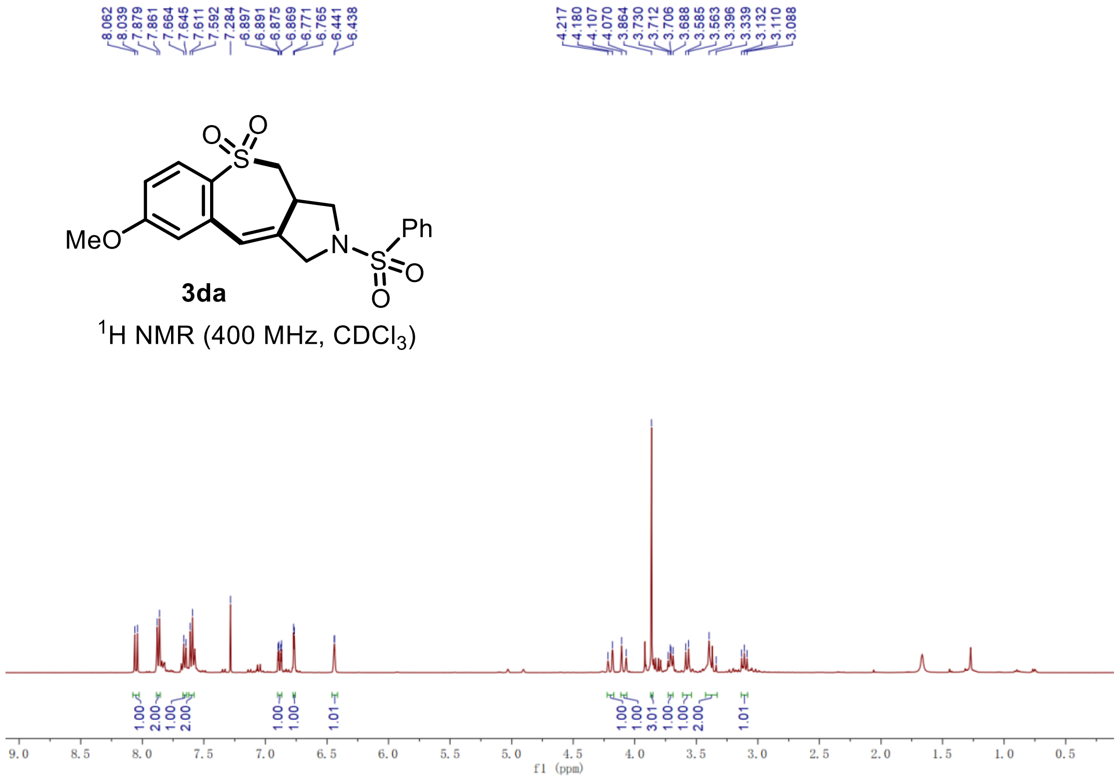
8.062
8.039
7.879
7.861
7.664
7.645
7.611
7.592
7.284
6.897
6.891
6.875
6.869
6.771
6.765
6.441
6.438
4.217
4.180
4.107
4.070
3.864
3.730
3.712
3.706
3.688
3.585
3.563
3.396
3.339
3.132
3.110
3.088
MeO
Ph
3da
1H NMR (400 MHz, CDCl3)
f1 (ppm)

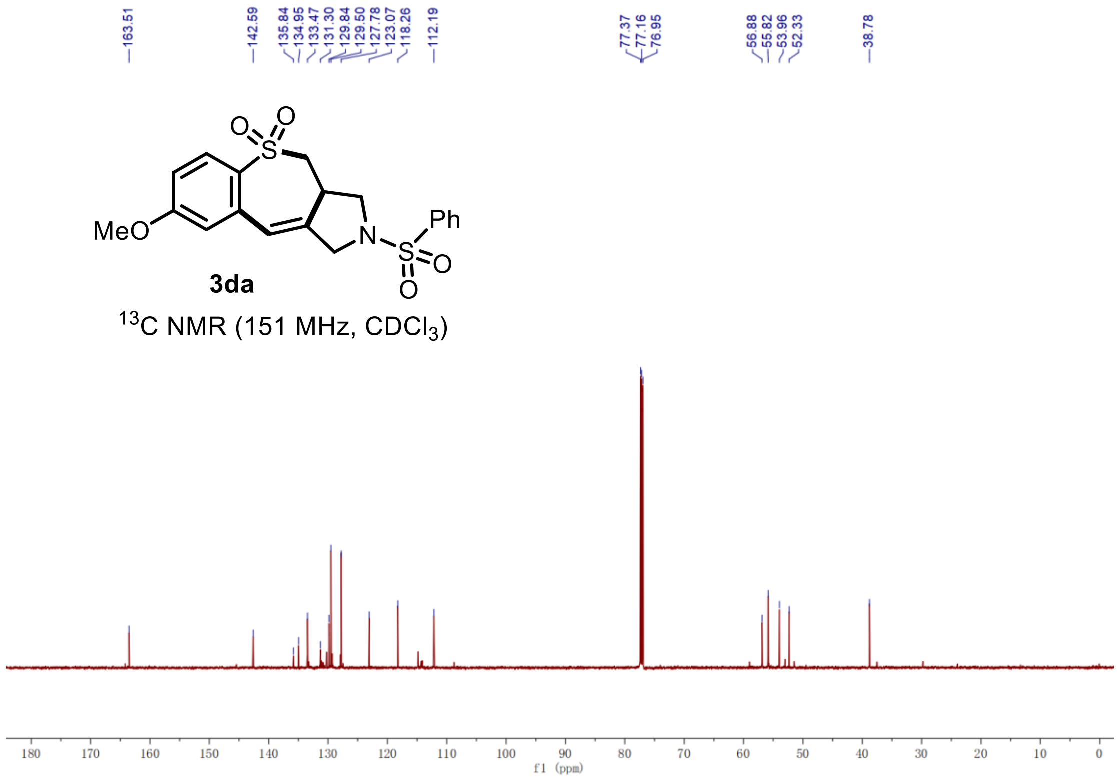

3da

^{13}C NMR (151 MHz, $CDCl_3$)

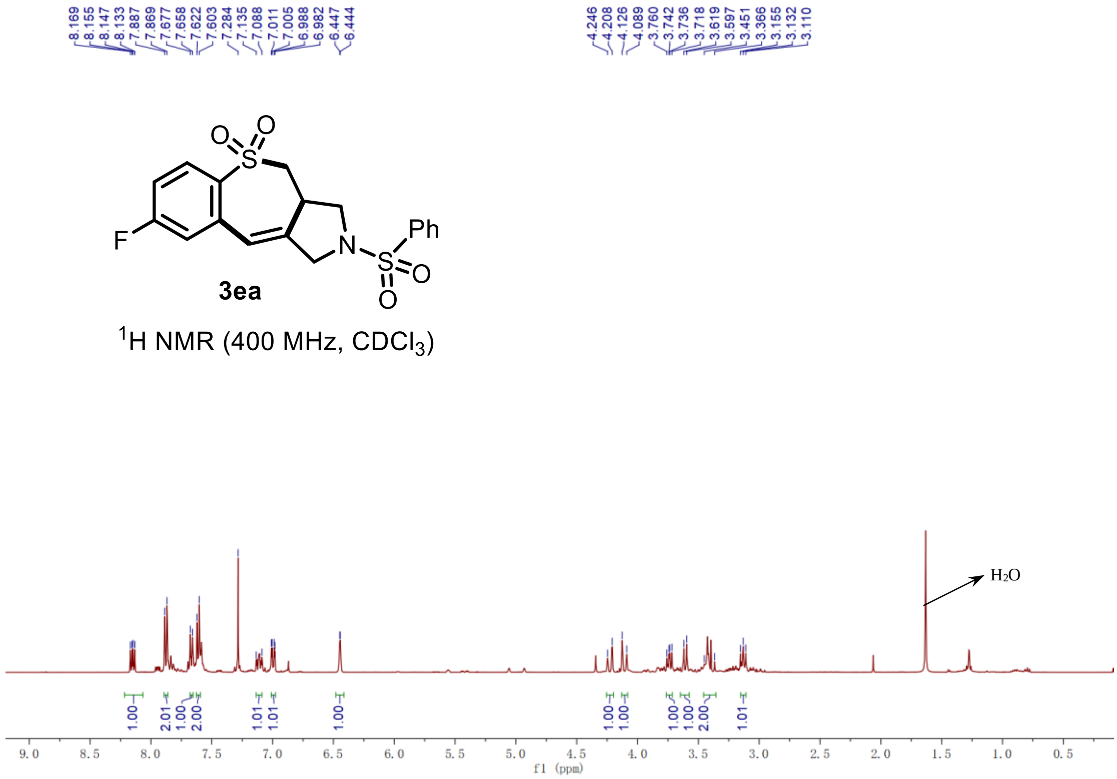

3ea

^{1}H NMR (400 MHz, $CDCl_3$)

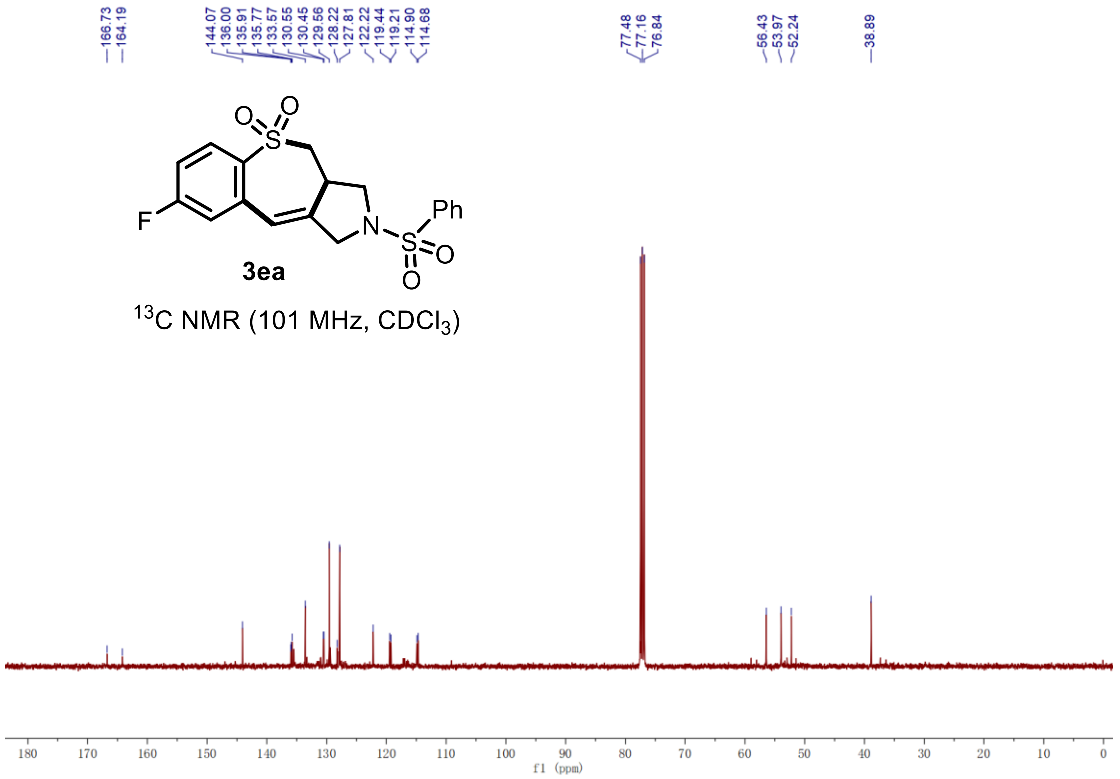
166.73
164.19
144.07
136.00
135.91
135.77
133.57
130.55
130.45
129.56
128.22
127.81
122.22
119.44
119.21
114.90
114.68
77.48
77.16
76.84
56.43
53.97
52.24
38.89
3ea
13C NMR (101 MHz, CDCl3)
f1 (ppm)

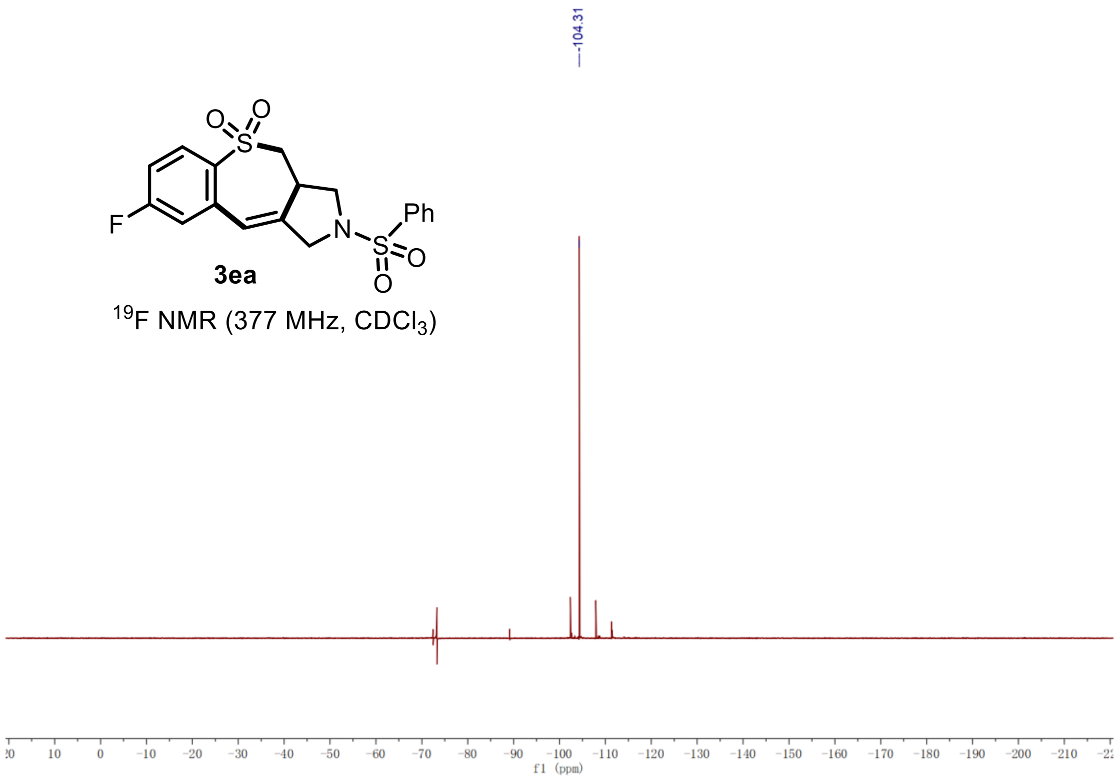
-104.31
3ea
19F NMR (377 MHz, CDCl3)
f1 (ppm)

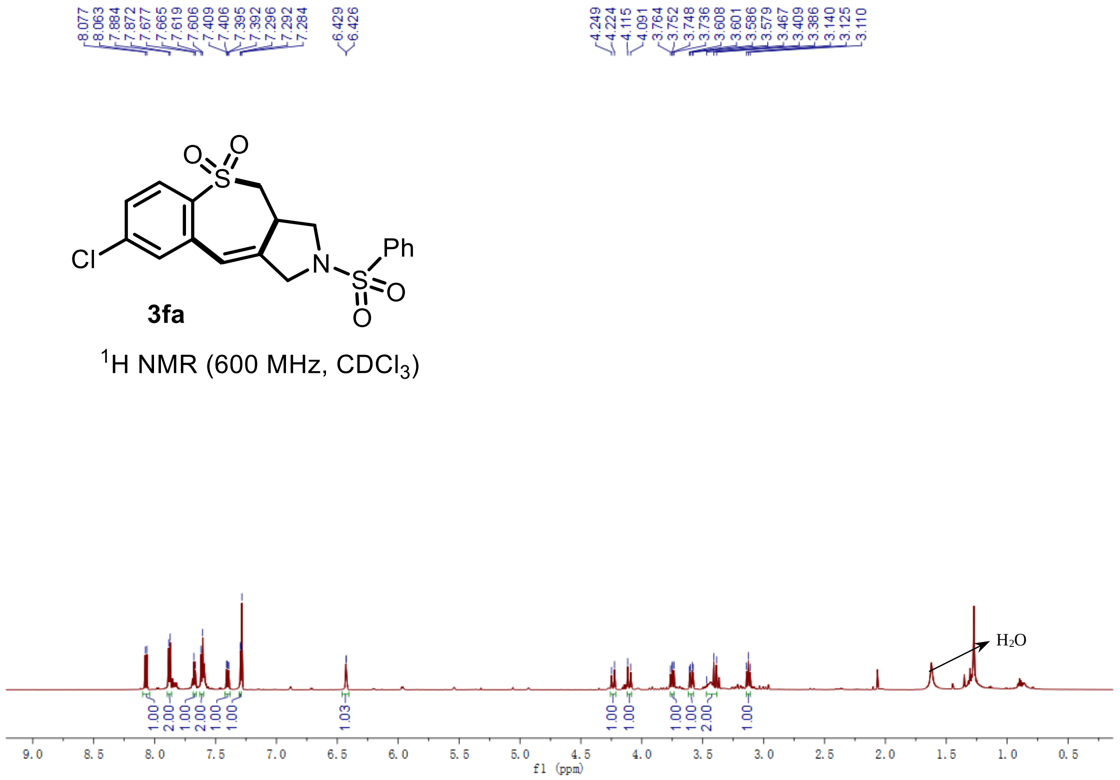

3fa

^{1}H NMR (600 MHz, $CDCl_3$)

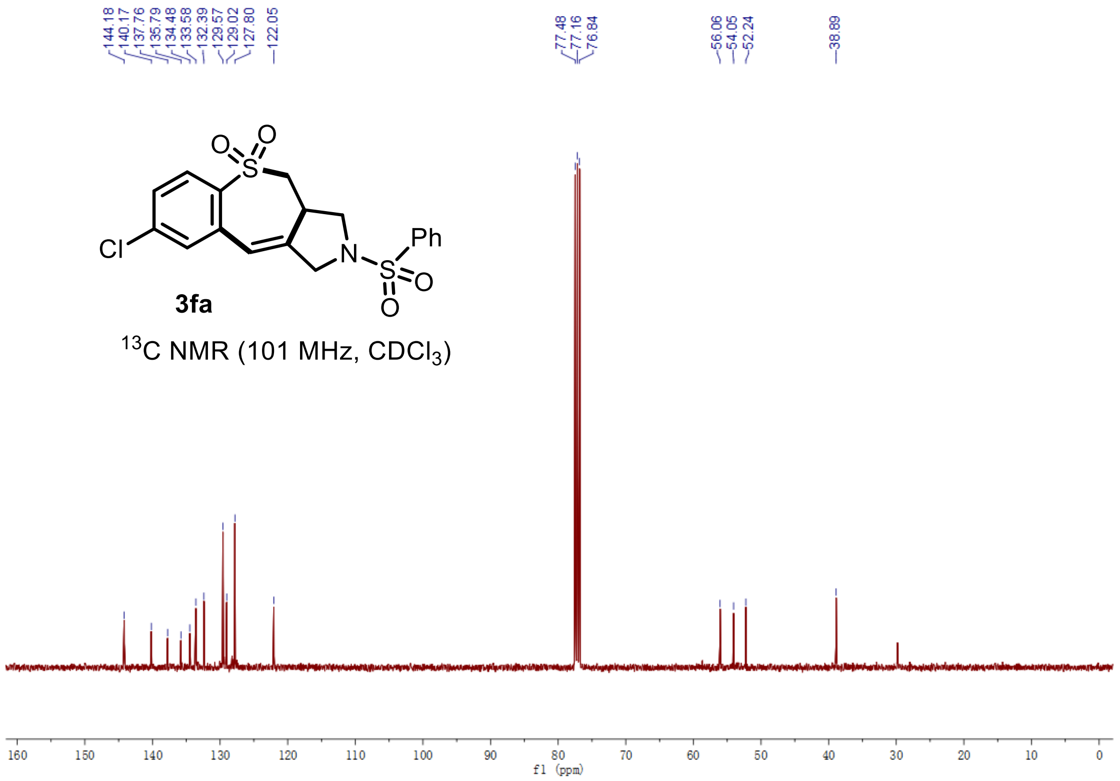

3fa

^{13}C NMR (101 MHz, $CDCl_3$)

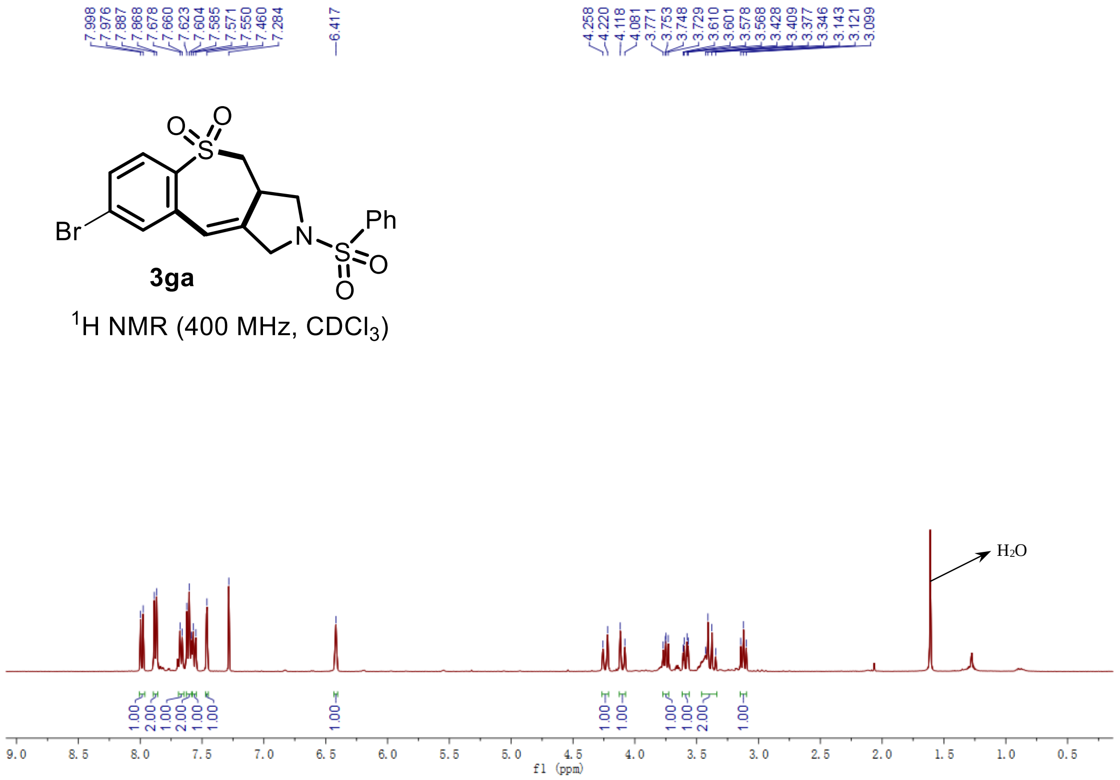
7.998
7.976
7.887
7.868
7.678
7.660
7.623
7.604
7.585
7.571
7.550
7.460
7.284
6.417
4.258
4.220
4.118
4.081
3.771
3.753
3.748
3.729
3.610
3.601
3.578
3.568
3.428
3.409
3.377
3.346
3.143
3.121
3.099
Br
Ph
3ga
^{1}H NMR (400 MHz, CDCl$_3$)
H$_2$O
f1 (ppm)

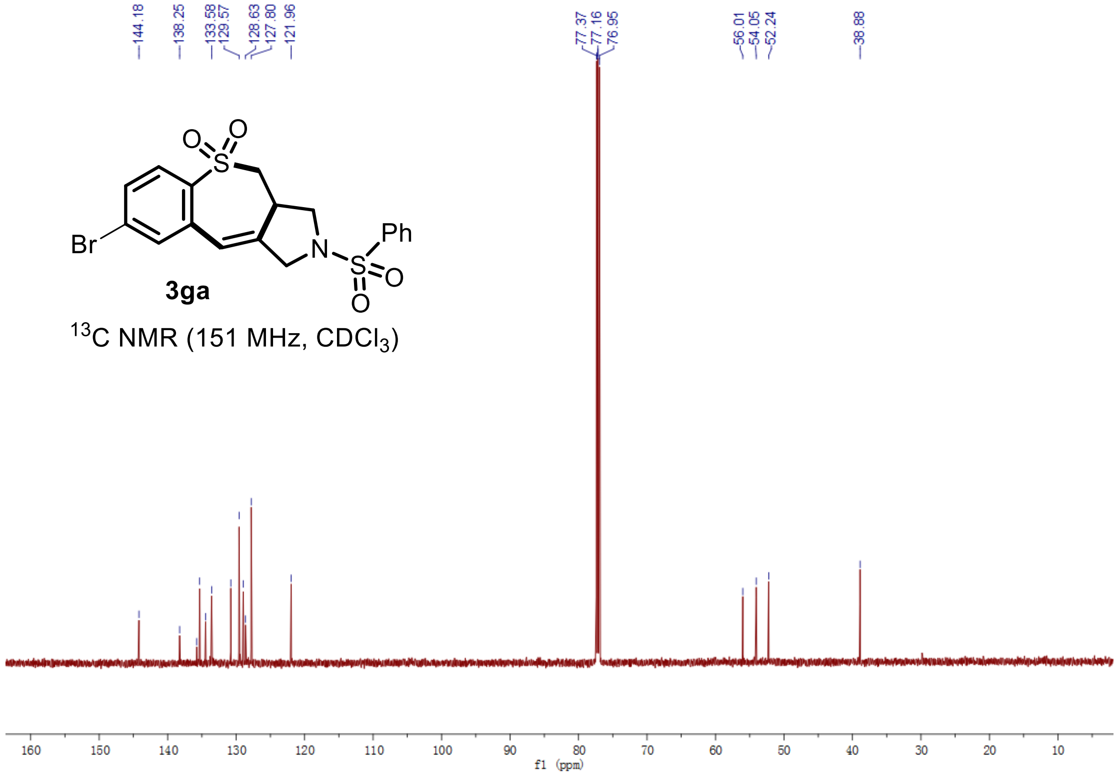
144.18
138.25
133.58
129.57
128.63
127.80
121.96
77.37
77.16
76.95
56.01
54.05
52.24
38.88
Br
Ph
3ga
^{13}C NMR (151 MHz, CDCl$_3$)
f1 (ppm)

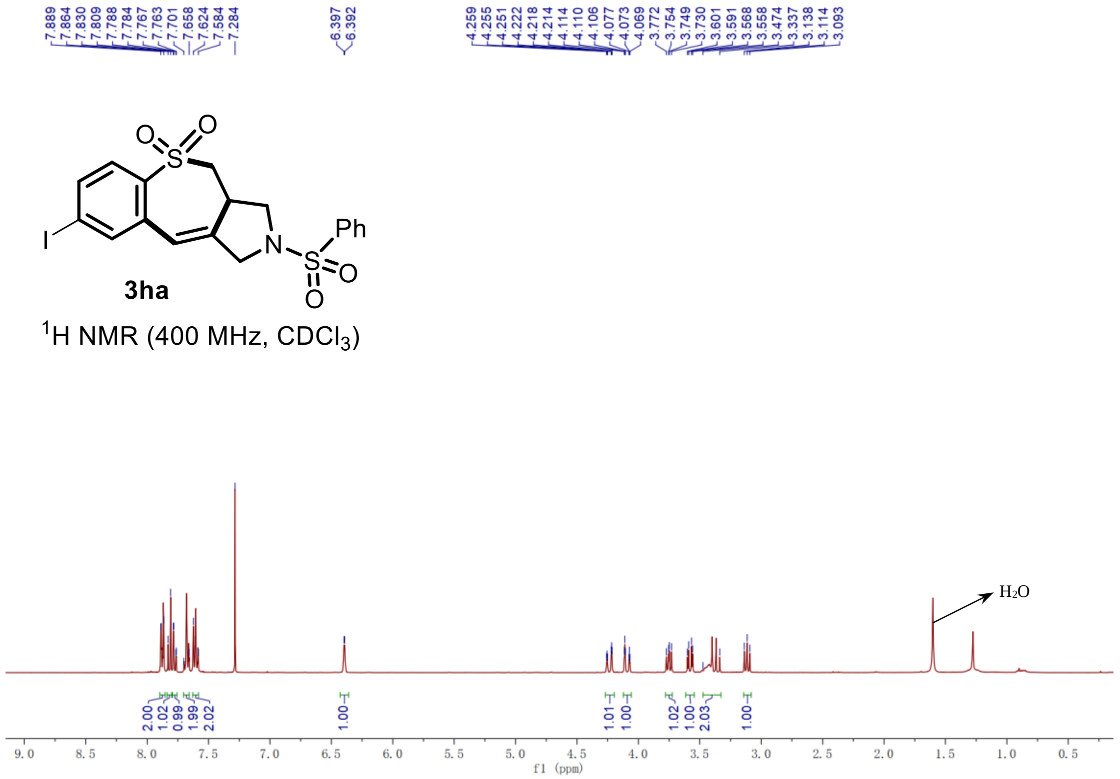
7.889
7.864
7.830
7.809
7.788
7.784
7.767
7.763
7.701
7.658
7.624
7.584
7.284
6.397
6.392
4.259
4.255
4.251
4.222
4.218
4.214
4.114
4.110
4.106
4.077
4.073
4.069
3.772
3.754
3.749
3.730
3.601
3.591
3.568
3.558
3.474
3.337
3.138
3.114
3.093
3ha
1H NMR (400 MHz, CDCl3)
H2O
2.00
1.02
0.99
1.99
2.02
1.00
1.01
1.00
1.02
1.00
2.03
1.00
f1 (ppm)

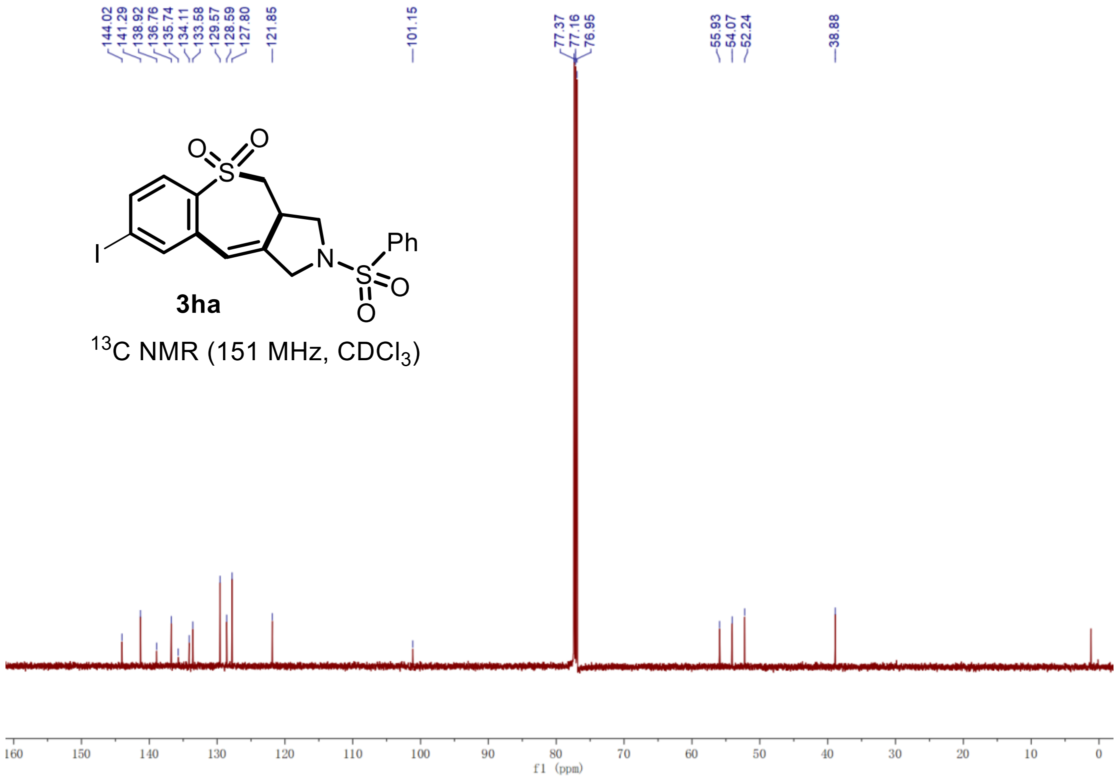
144.02
141.29
138.92
136.76
135.74
134.11
133.58
129.57
128.59
127.80
121.85
101.15
77.37
77.16
76.95
55.93
54.07
52.24
38.88
3ha
13C NMR (151 MHz, CDCl3)
f1 (ppm)

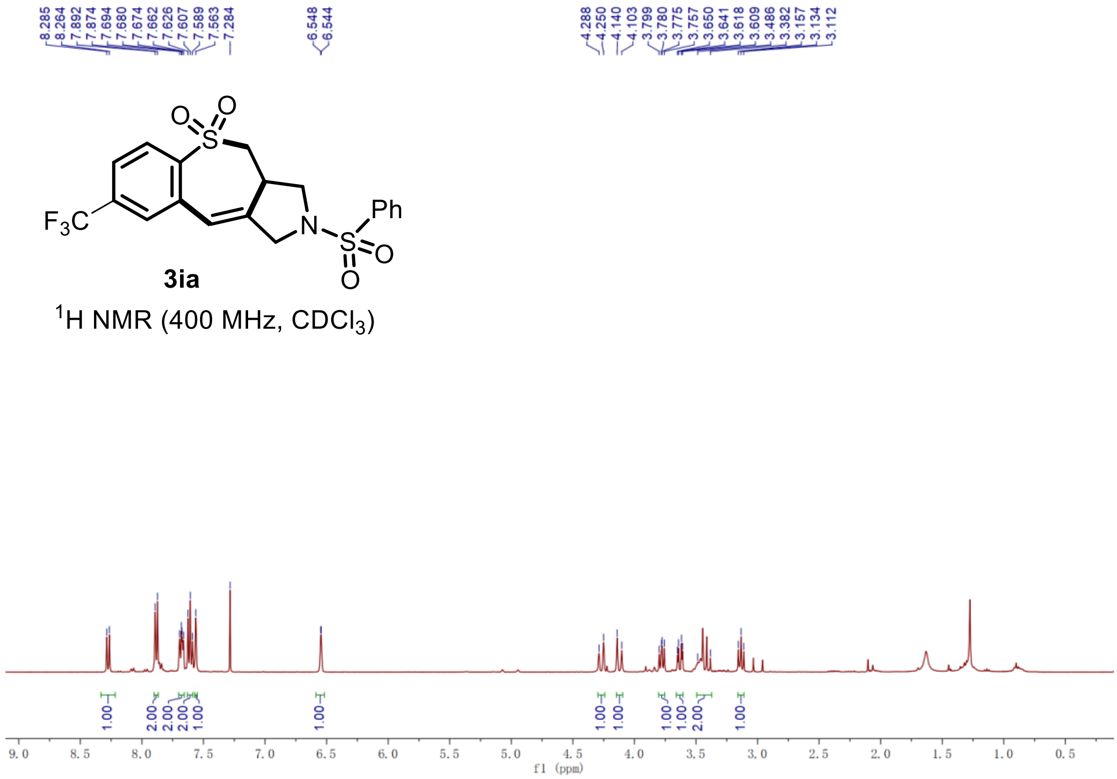
8.285
8.264
7.892
7.874
7.694
7.680
7.674
7.662
7.626
7.607
7.589
7.563
7.284
6.548
6.544
4.288
4.250
4.140
4.103
3.799
3.780
3.775
3.757
3.650
3.641
3.618
3.609
3.486
3.382
3.157
3.134
3.112
O
O
S
F3C
N
Ph
S
O
O
3ia
1H NMR (400 MHz, CDCl3)
1.00
2.00
2.00
2.00
1.00
1.00
1.00
1.00
1.00
1.00
2.00
1.00
9.0
8.5
8.0
7.5
7.0
6.5
6.0
5.5
5.0
4.5
4.0
3.5
3.0
2.5
2.0
1.5
1.0
0.5
f1 (ppm)

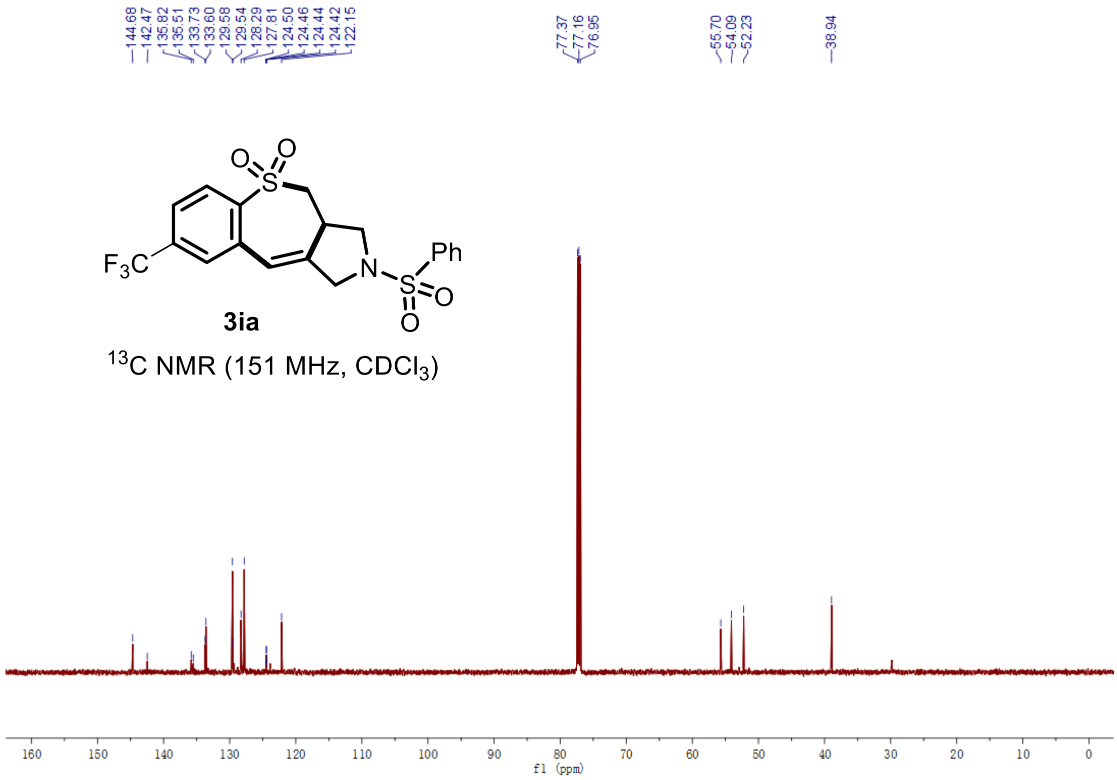
144.68
142.47
135.82
135.51
133.73
133.60
129.58
129.54
128.29
127.81
124.50
124.46
124.44
124.42
122.15
77.37
77.16
76.95
55.70
54.09
52.23
38.94
O
O
S
F3C
N
Ph
S
O
O
3ia
13C NMR (151 MHz, CDCl3)
160
150
140
130
120
110
100
90
80
70
60
50
40
30
20
10
0
f1 (ppm)

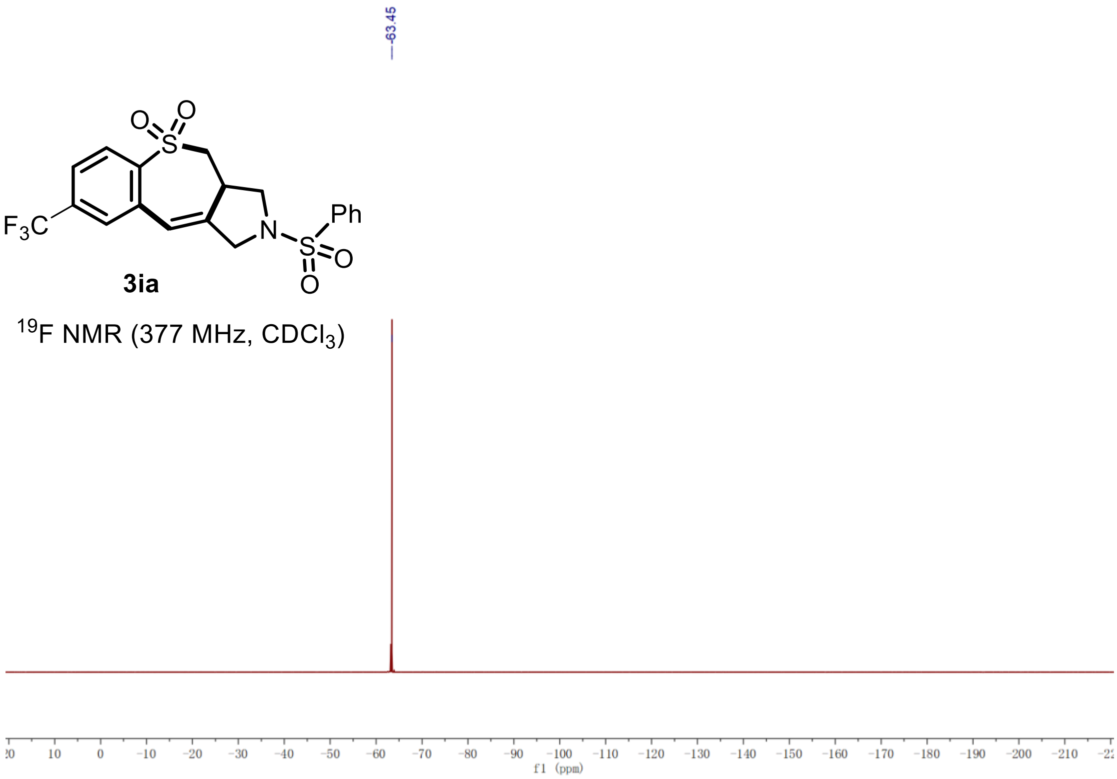
-63.45
F3C
Ph
3ia
19F NMR (377 MHz, CDCl3)
f1 (ppm)

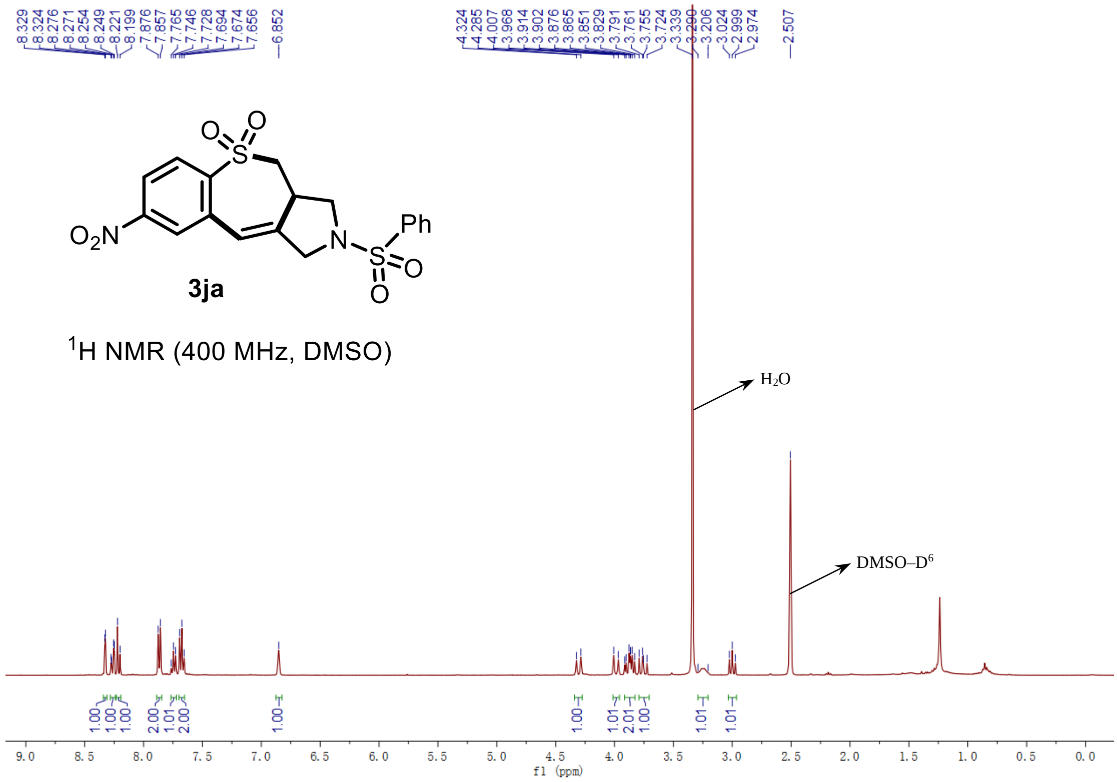
8.329
8.324
8.276
8.271
8.254
8.249
8.221
8.199
7.876
7.857
7.765
7.746
7.728
7.694
7.674
7.656
6.852
4.324
4.285
4.007
3.968
3.914
3.902
3.876
3.865
3.851
3.829
3.791
3.761
3.755
3.724
3.339
3.206
3.024
2.999
2.974
2.507
O2N
Ph
3ja
1H NMR (400 MHz, DMSO)
H2O
DMSO–D6
f1 (ppm)

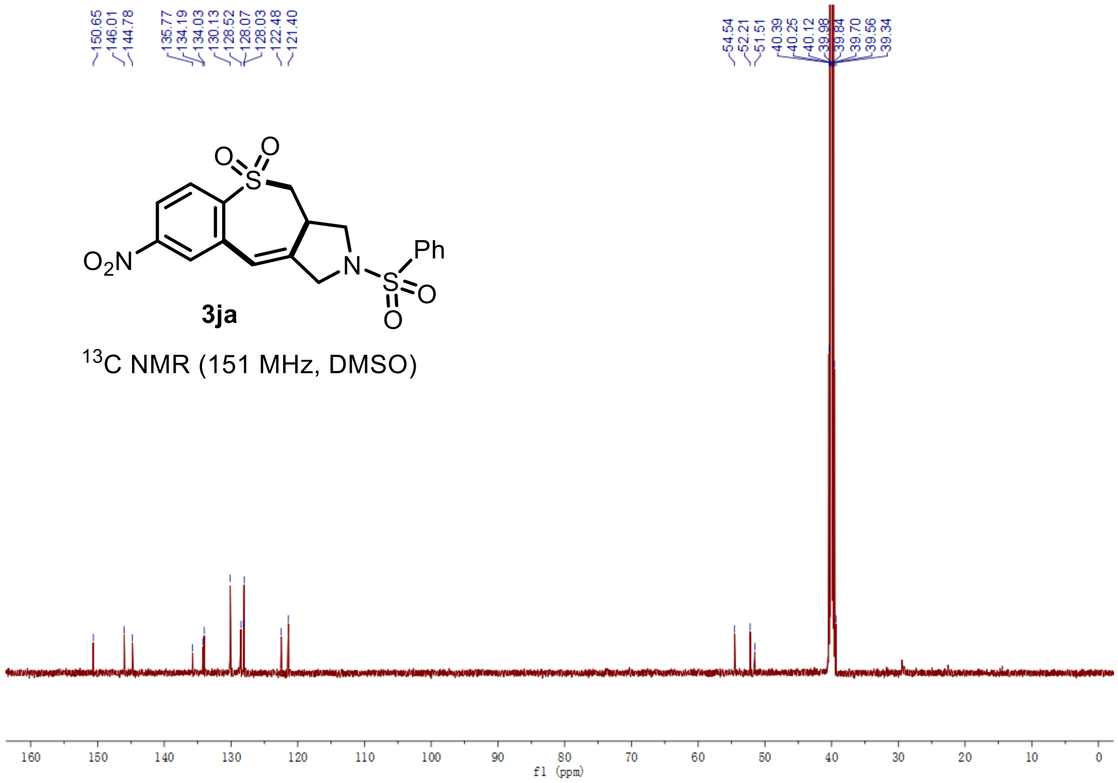
150.65
146.01
144.78
135.77
134.19
134.03
130.13
128.52
128.07
128.03
122.48
121.40
54.54
52.21
51.51
40.39
40.25
40.12
39.98
39.84
39.70
39.56
39.34
O
O
S
O_2N
N
Ph
S
O
O
3ja
^{13}C NMR (151 MHz, DMSO)
f1 (ppm)

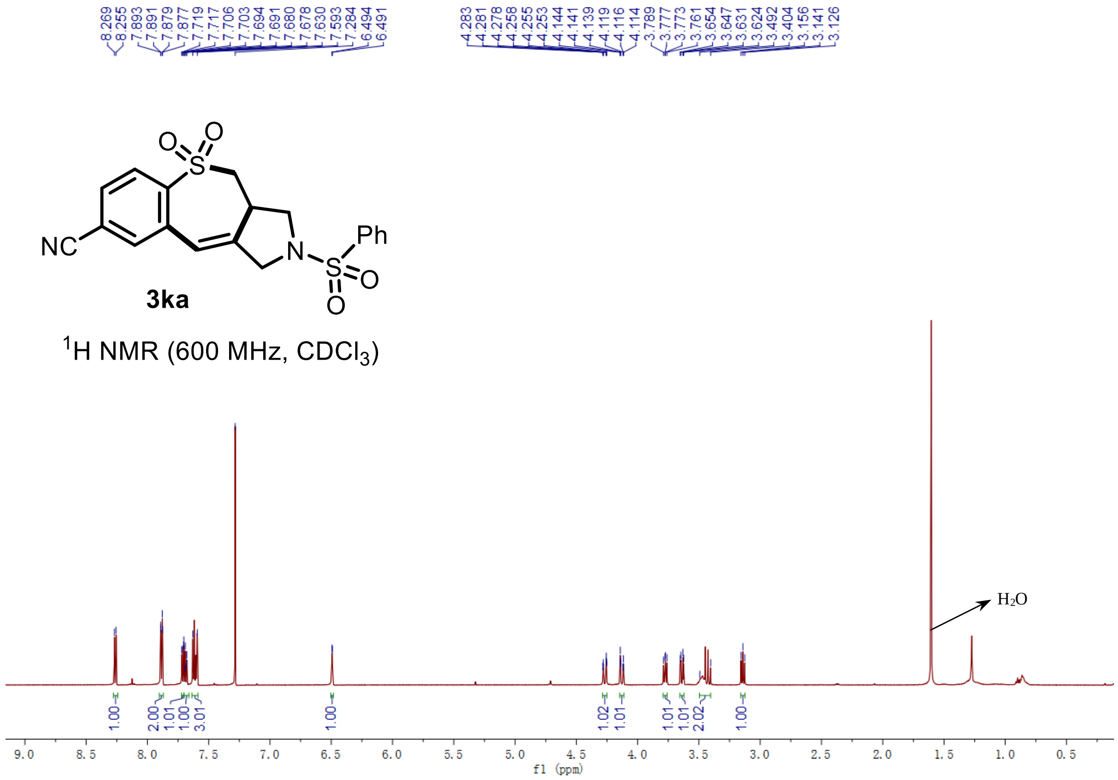
8.269
8.255
7.893
7.891
7.879
7.877
7.719
7.717
7.706
7.703
7.694
7.691
7.680
7.678
7.630
7.593
7.284
6.494
6.491
4.283
4.281
4.278
4.258
4.255
4.253
4.144
4.141
4.139
4.119
4.116
4.114
3.789
3.777
3.773
3.761
3.654
3.647
3.631
3.624
3.492
3.404
3.156
3.141
3.126
O
O
S
NC
N
Ph
S
O
O
3ka
^{1}H NMR (600 MHz, $CDCl_3$)
H_2O
1.00
2.00
1.01
1.00
3.01
1.00
1.02
1.01
1.01
1.01
2.02
1.00
f1 (ppm)

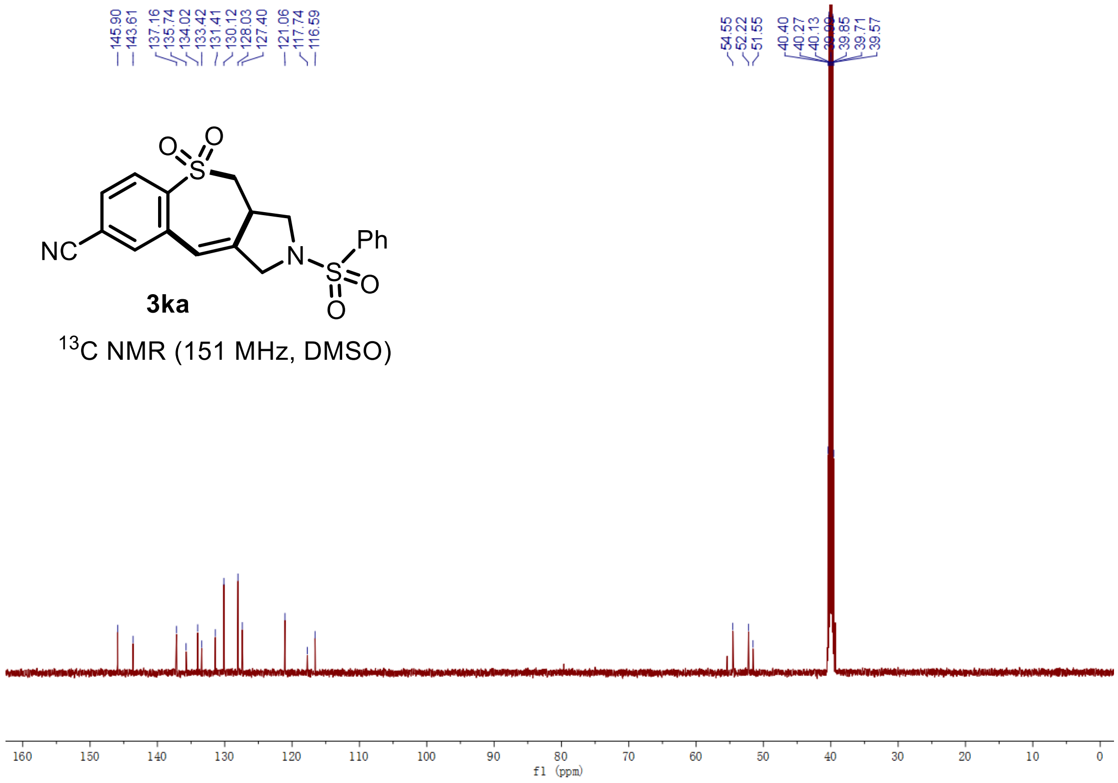
145.90
143.61
137.16
135.74
134.02
133.42
131.41
130.12
128.03
127.40
121.06
117.74
116.59
54.55
52.22
51.55
40.40
40.27
40.13
39.85
39.71
39.57
NC
Ph
3ka
13C NMR (151 MHz, DMSO)
f1 (ppm)

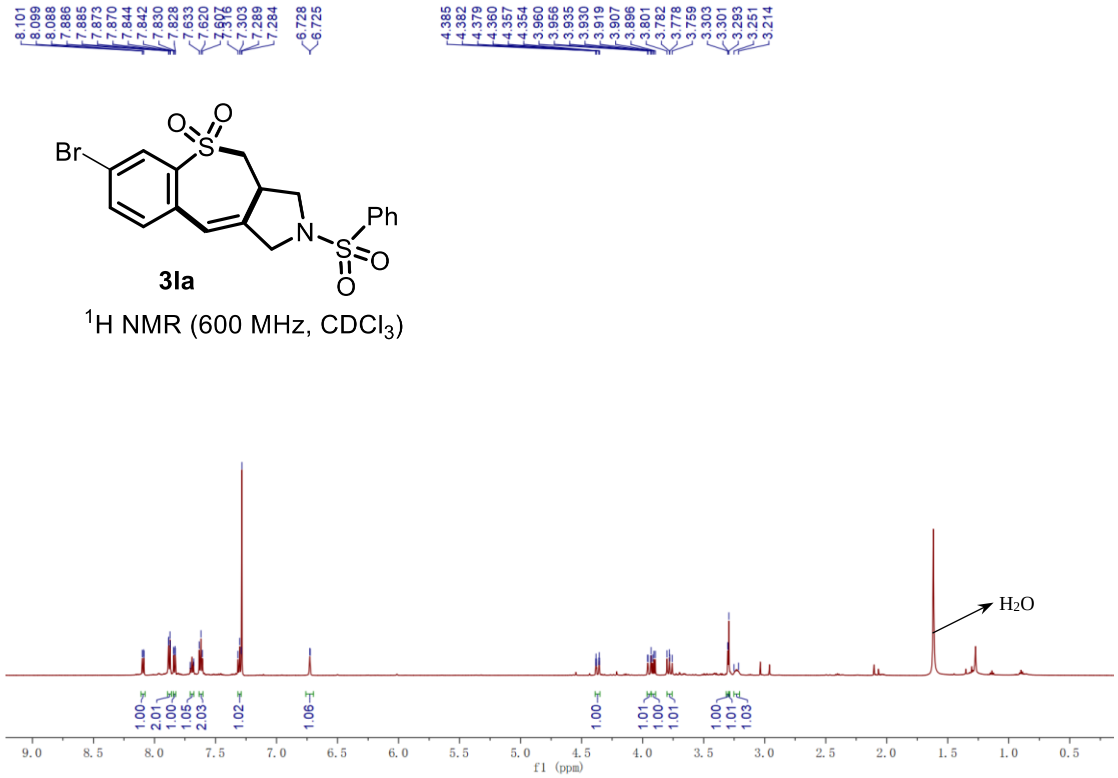
8.101
8.099
8.088
7.886
7.885
7.873
7.870
7.844
7.842
7.830
7.828
7.633
7.620
7.303
7.289
7.284
6.728
6.725
4.385
4.382
4.379
4.360
4.357
4.354
3.960
3.956
3.935
3.930
3.919
3.907
3.896
3.801
3.782
3.778
3.759
3.303
3.301
3.293
3.251
3.214
Br
Ph
3la
1H NMR (600 MHz, CDCl3)
H2O
f1 (ppm)

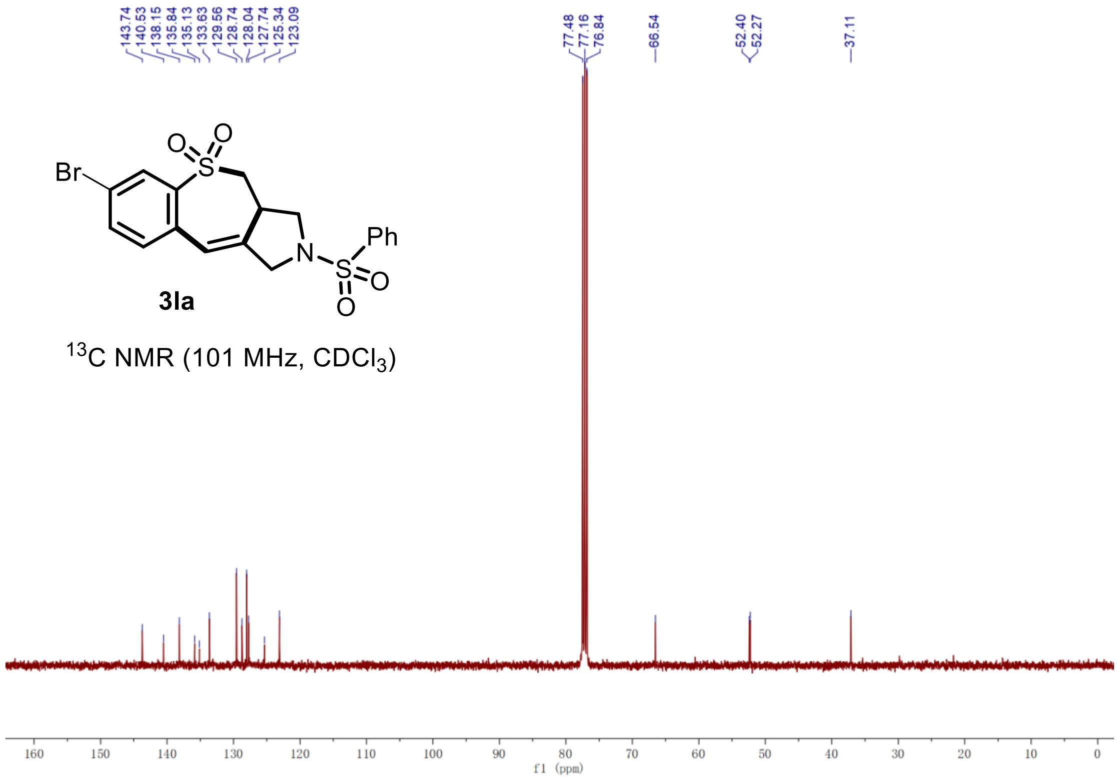
143.74
140.53
138.15
135.84
135.13
133.63
129.56
128.74
128.04
127.74
125.34
123.09
77.48
77.16
76.84
66.54
52.40
52.27
37.11
Br
Ph
3la
^{13}C NMR (101 MHz, $CDCl_3$)
f1 (ppm)

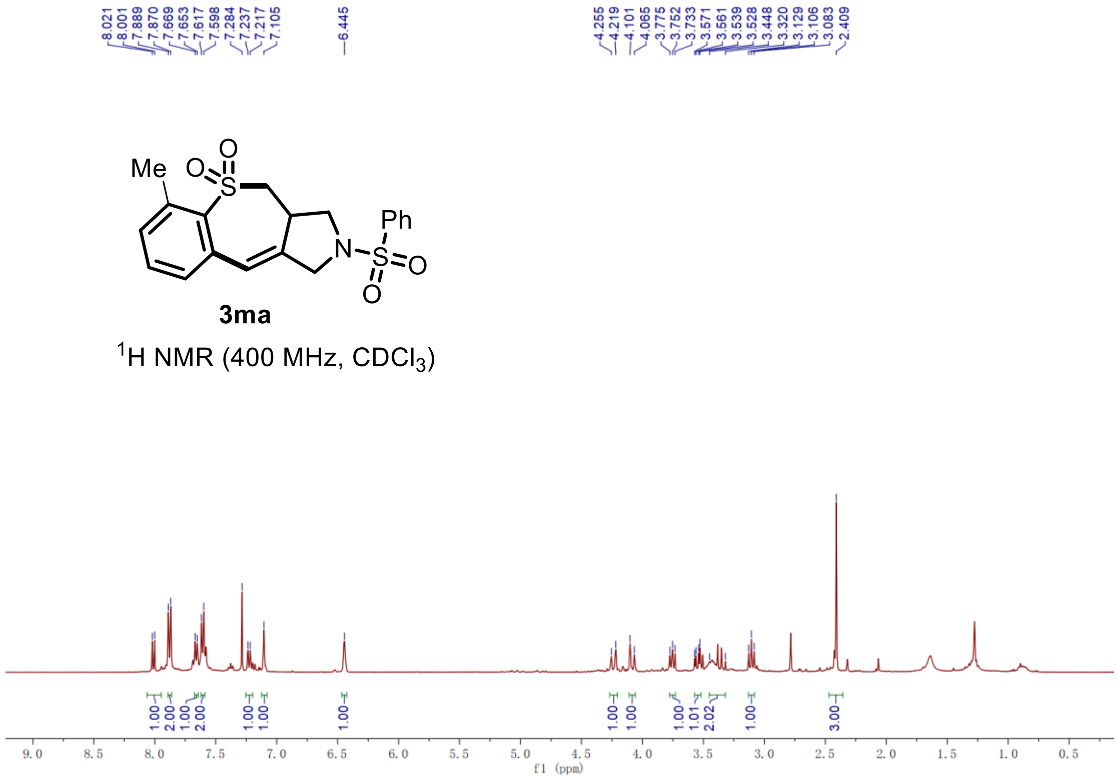
8.021
8.001
7.889
7.870
7.669
7.653
7.617
7.598
7.284
7.237
7.217
7.105
6.445
4.255
4.219
4.101
4.065
3.775
3.752
3.733
3.571
3.561
3.539
3.528
3.448
3.320
3.129
3.106
3.083
2.409
Me
Ph
3ma
^{1}H NMR (400 MHz, $CDCl_3$)
1.00
2.00
1.00
2.00
1.00
1.00
1.00
1.00
1.00
1.00
1.01
2.02
1.00
3.00
f1 (ppm)

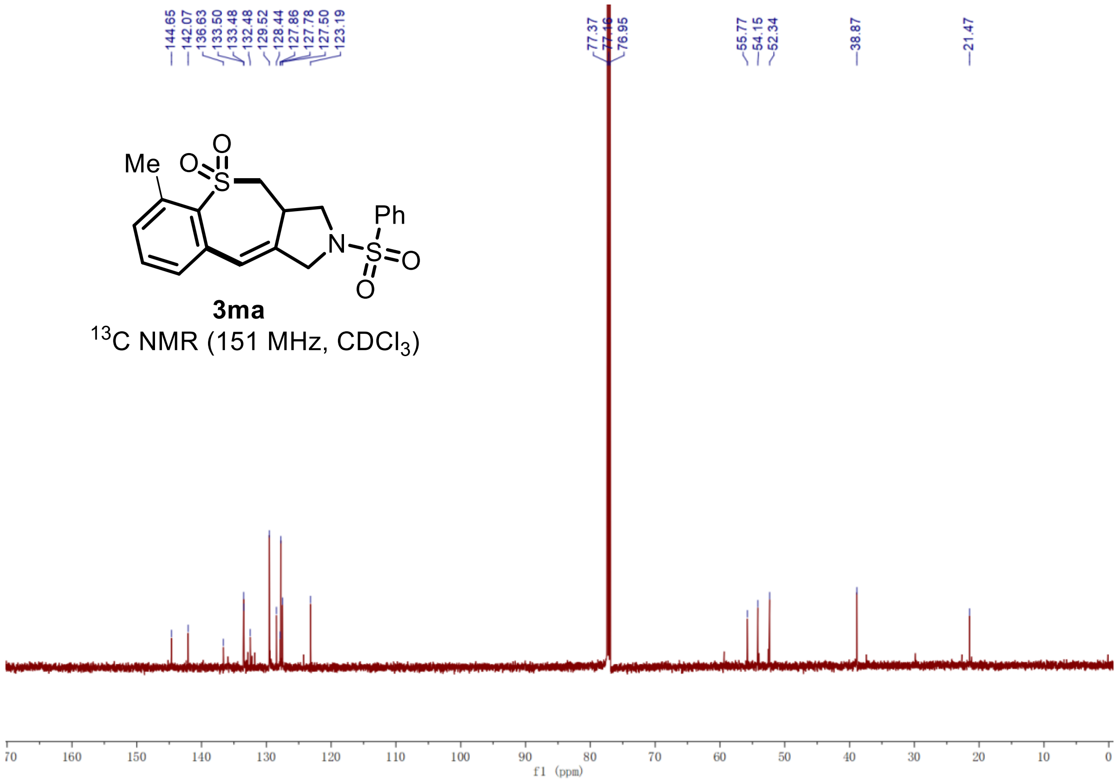

3ma

^{13}C NMR (151 MHz, $CDCl_3$)

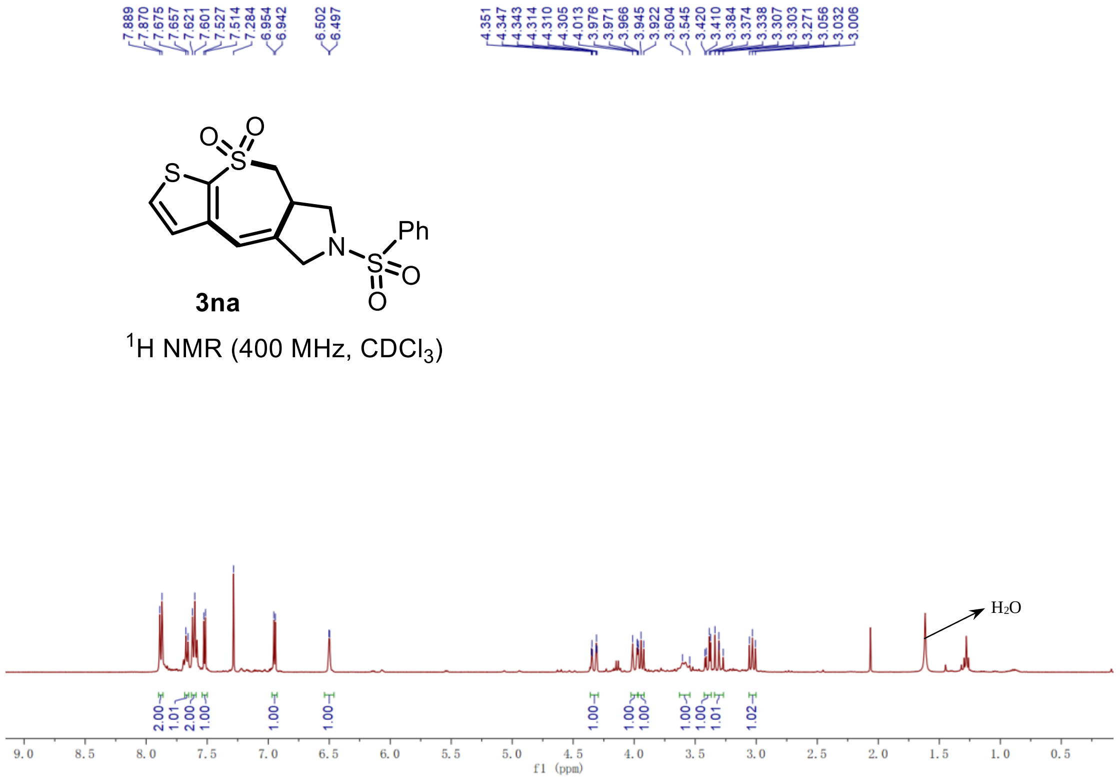

3na

^{1}H NMR (400 MHz, $CDCl_3$)

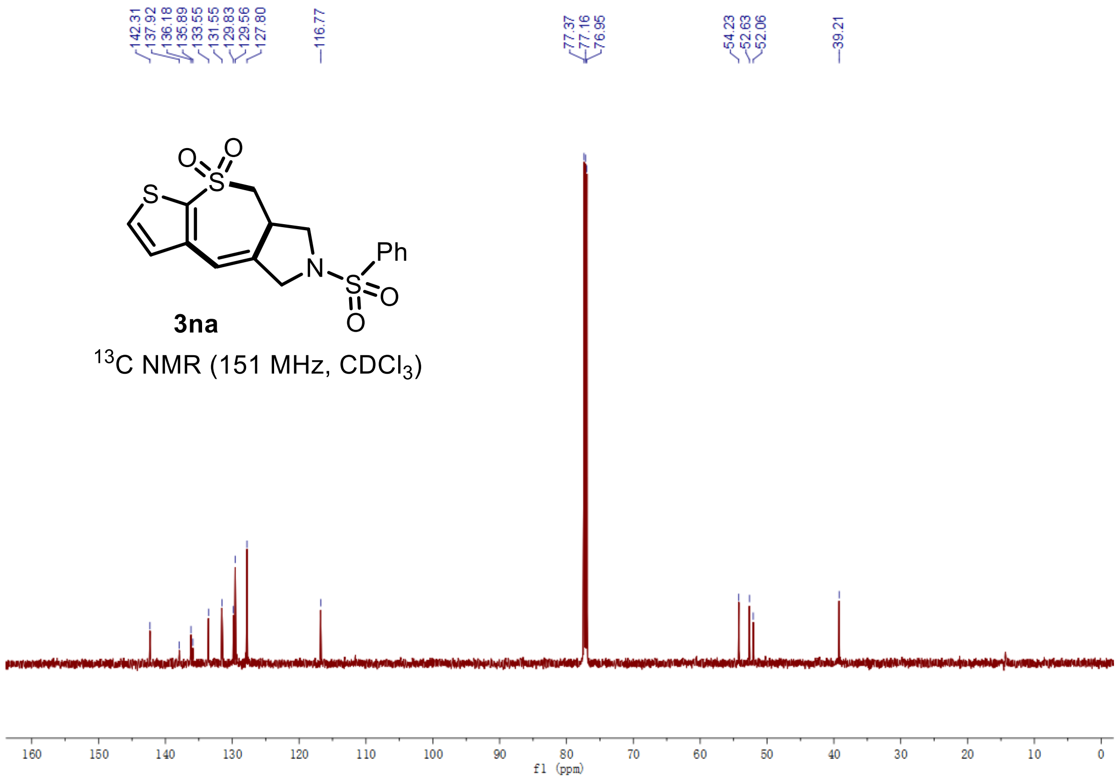
142.31
137.92
136.18
135.89
133.55
131.55
129.83
129.56
127.80
116.77
77.37
77.16
76.95
54.23
52.63
52.06
39.21
3na
^{13}C NMR (151 MHz, $CDCl_3$)
f1 (ppm)

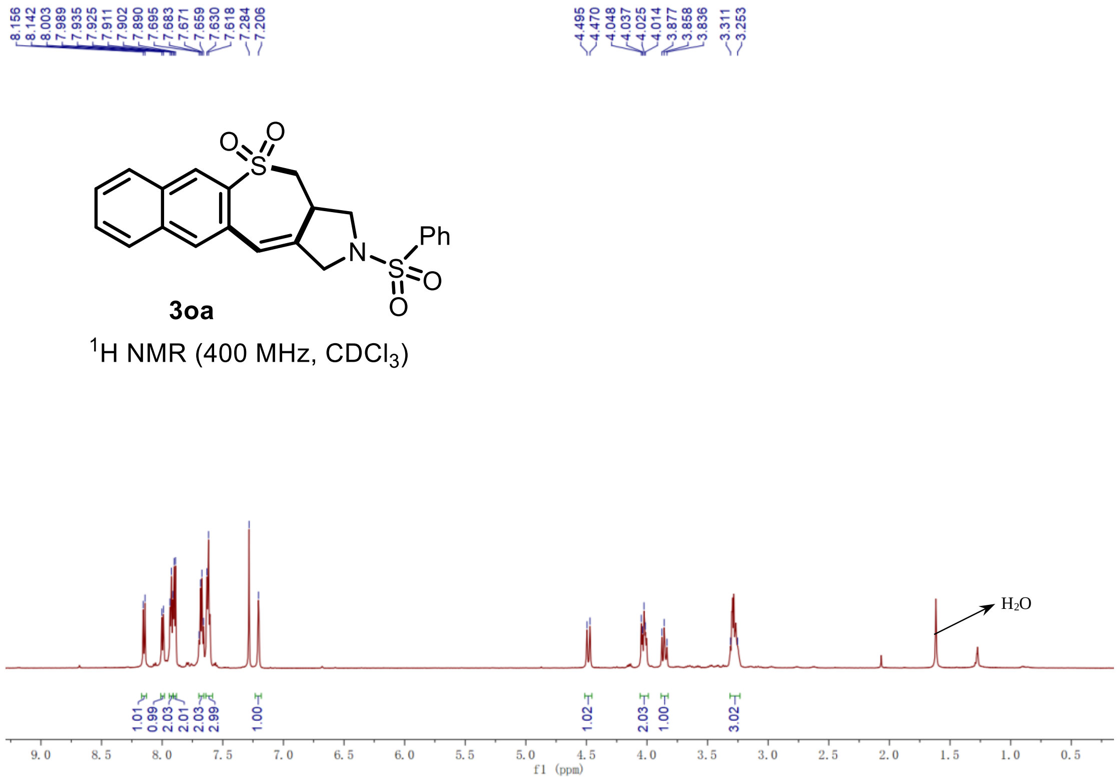
8.156
8.142
8.003
7.989
7.935
7.925
7.911
7.902
7.890
7.695
7.683
7.671
7.659
7.630
7.618
7.284
7.206
4.495
4.470
4.048
4.037
4.025
4.014
3.877
3.858
3.836
3.311
3.253
3oa
^{1}H NMR (400 MHz, $CDCl_3$)
H_2O
1.01
0.99
2.03
2.01
2.03
2.99
1.00
1.02
2.03
1.00
3.02
f1 (ppm)

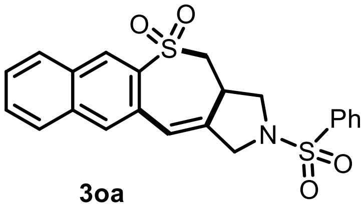

3oa

^{13}C NMR (151 MHz, $CDCl_3$)

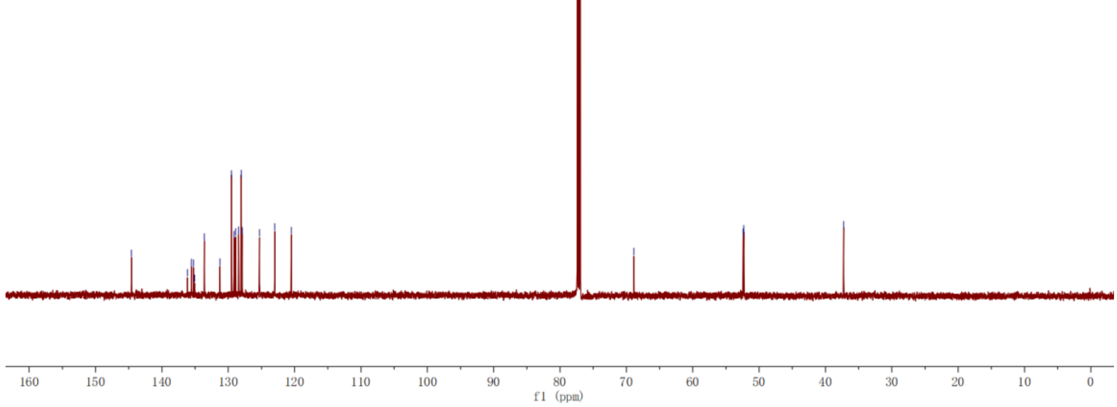

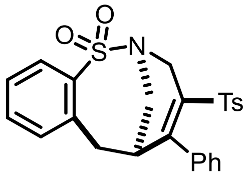

4a

^{1}H NMR (600 MHz, $CDCl_3$)

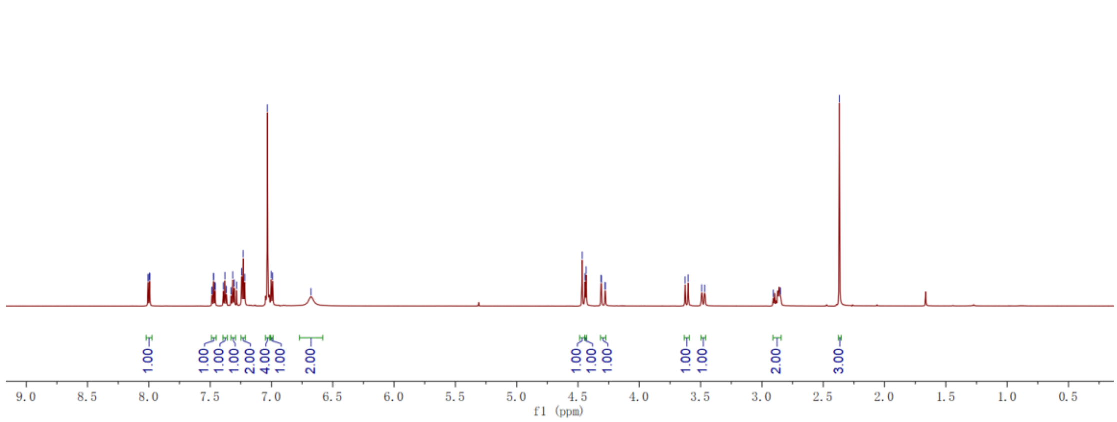

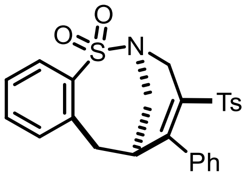

4a

^{13}C NMR (151 MHz, $CDCl_3$)

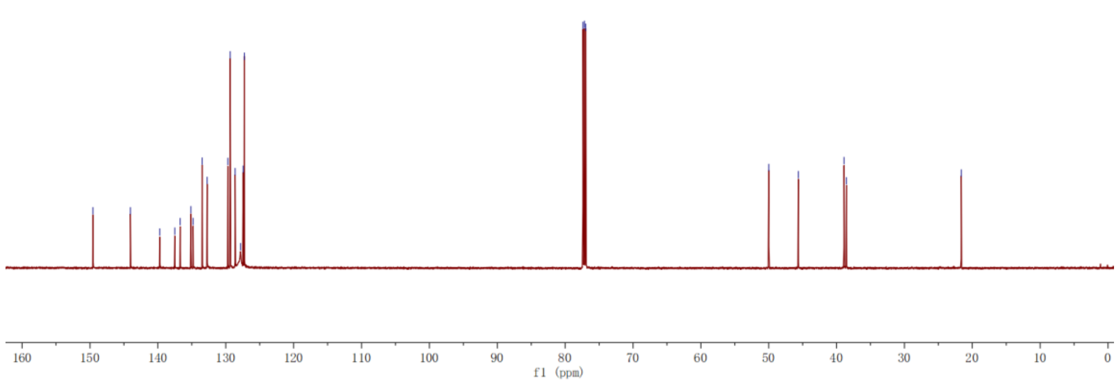

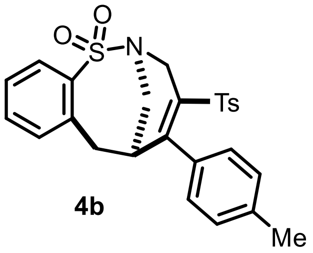

4b

^{1}H NMR (600 MHz, $CDCl_3$)

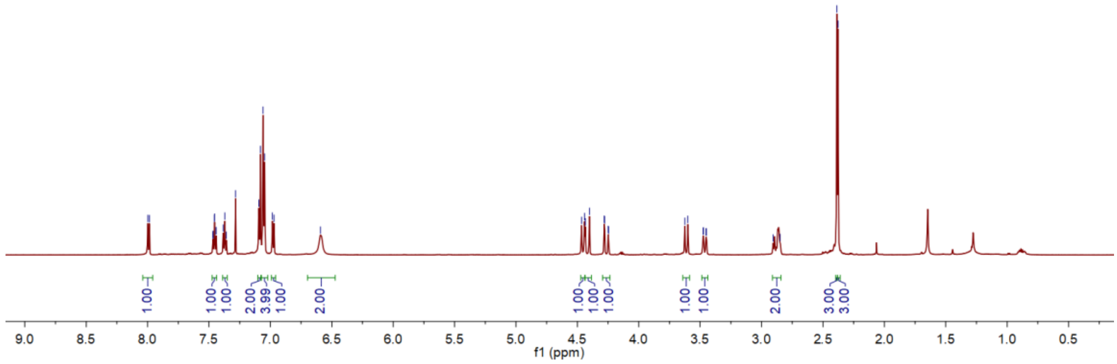

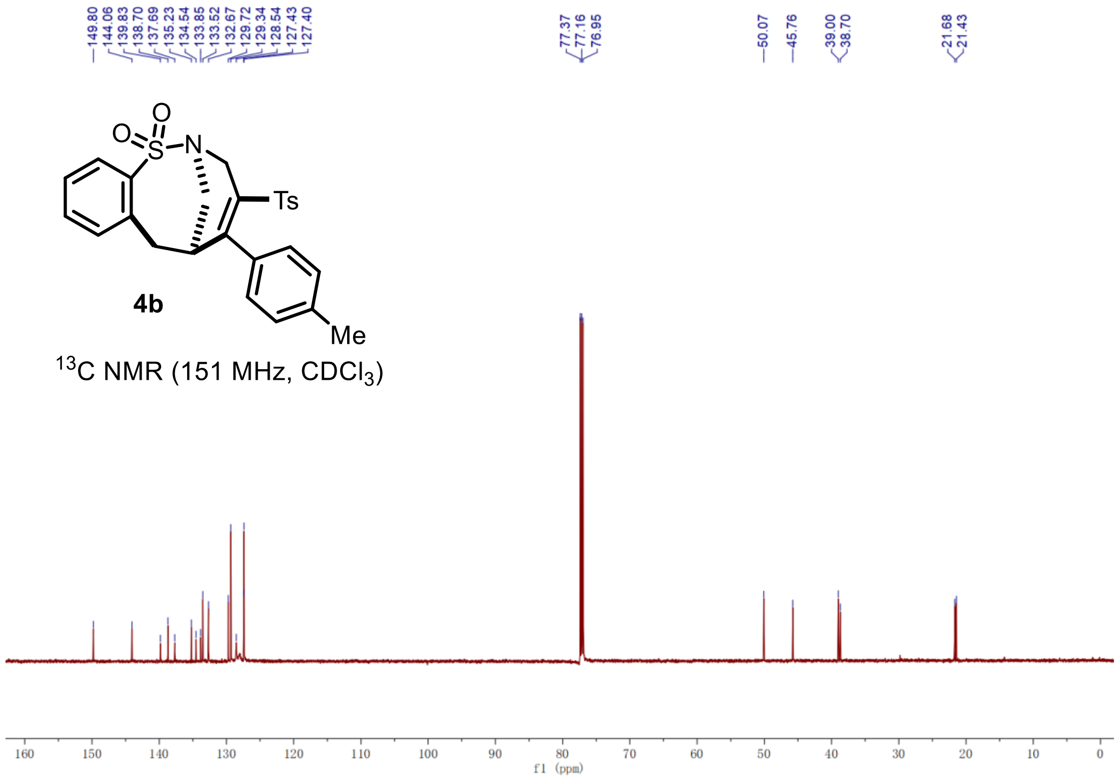
149.80
144.06
139.83
138.70
137.69
135.23
134.54
133.85
133.52
132.67
129.72
129.34
128.54
127.43
127.40
77.37
77.16
76.95
50.07
45.76
39.00
38.70
21.68
21.43
Ts
Me
4b
13C NMR (151 MHz, CDCl3)
f1 (ppm)

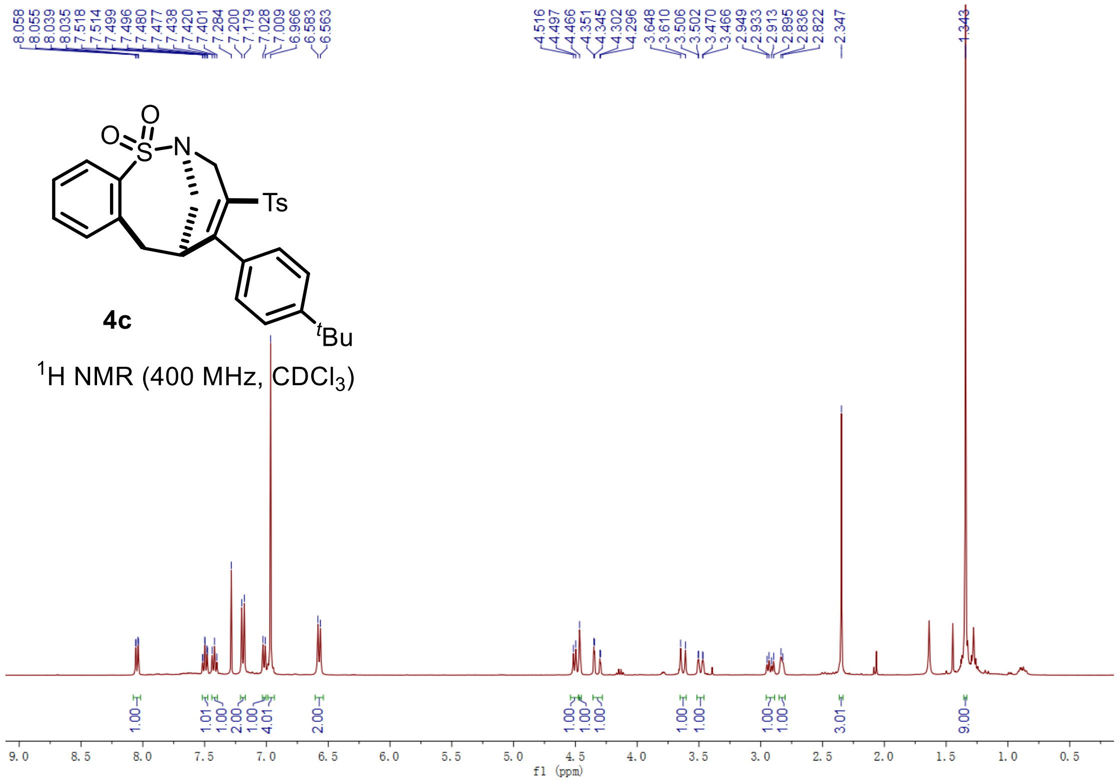
8.058
8.055
8.039
8.035
7.518
7.514
7.499
7.496
7.480
7.477
7.438
7.420
7.401
7.284
7.200
7.179
7.028
7.009
6.966
6.583
6.563
4.516
4.497
4.466
4.351
4.345
4.302
4.296
3.648
3.610
3.506
3.502
3.470
3.466
2.949
2.933
2.913
2.895
2.836
2.822
2.347
1.343
Ts
tBu
4c
1H NMR (400 MHz, CDCl3)
f1 (ppm)

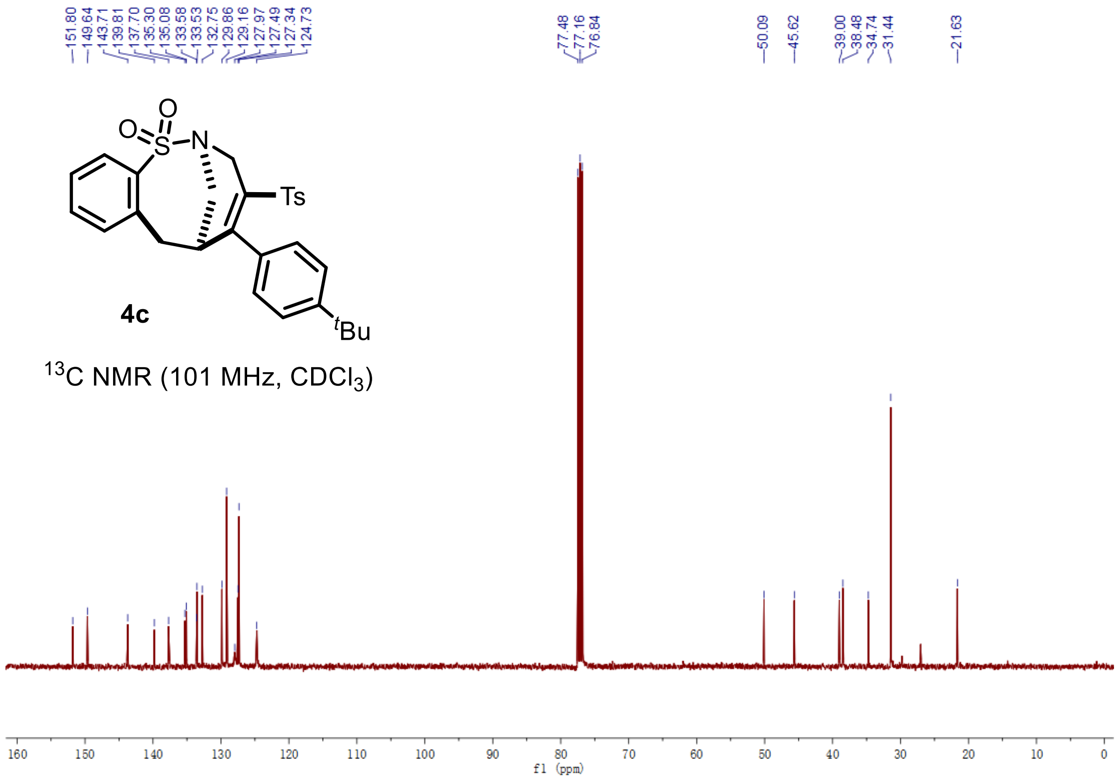

151.80
149.64
143.71
139.81
137.70
135.30
135.08
133.58
133.53
132.75
129.86
129.16
127.97
127.49
127.34
124.73
77.48
77.16
76.84
50.09
45.62
39.00
38.48
34.74
31.44
21.63
Ts
4c
tBu
13C NMR (101 MHz, CDCl3)
f1 (ppm)

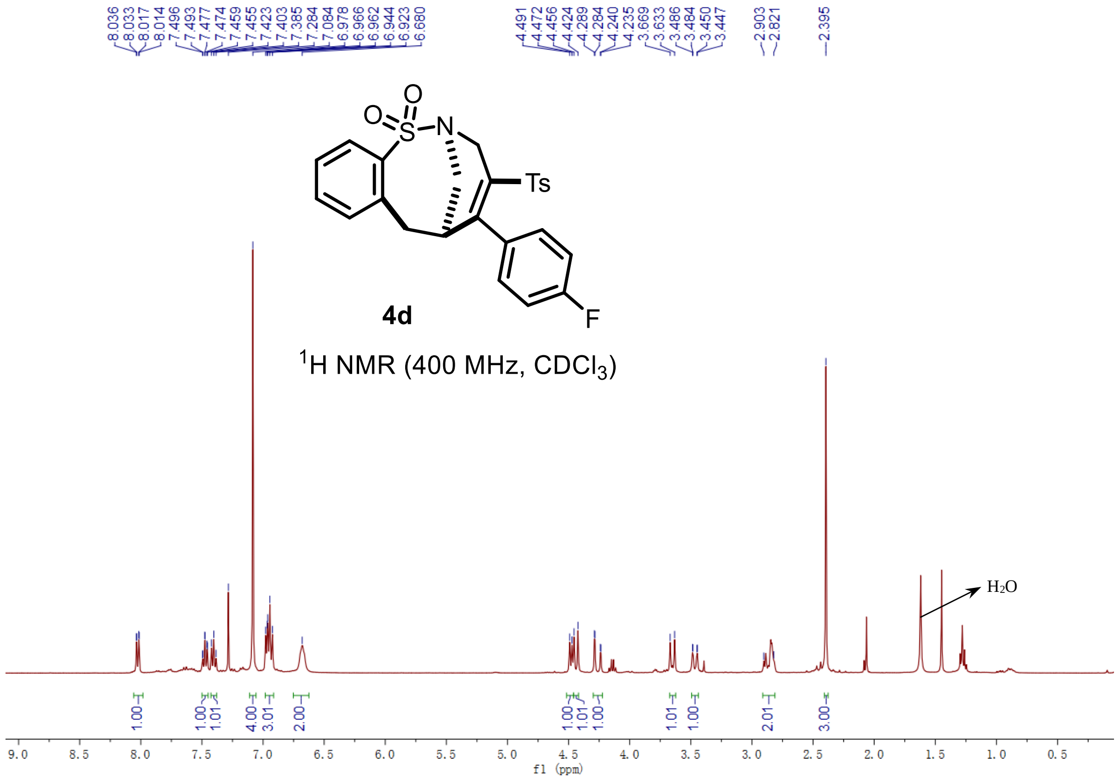

8.036
8.033
8.017
8.014
7.496
7.493
7.477
7.474
7.459
7.455
7.423
7.403
7.385
7.284
7.084
6.978
6.966
6.962
6.944
6.923
6.680
4.491
4.472
4.456
4.424
4.289
4.284
4.240
4.235
3.669
3.633
3.486
3.484
3.450
3.447
2.903
2.821
2.395
Ts
4d
F
1H NMR (400 MHz, CDCl3)
H2O
1.00
1.00
1.01
4.00
3.01
2.00
1.00
1.01
1.00
1.01
1.00
2.01
3.00
f1 (ppm)

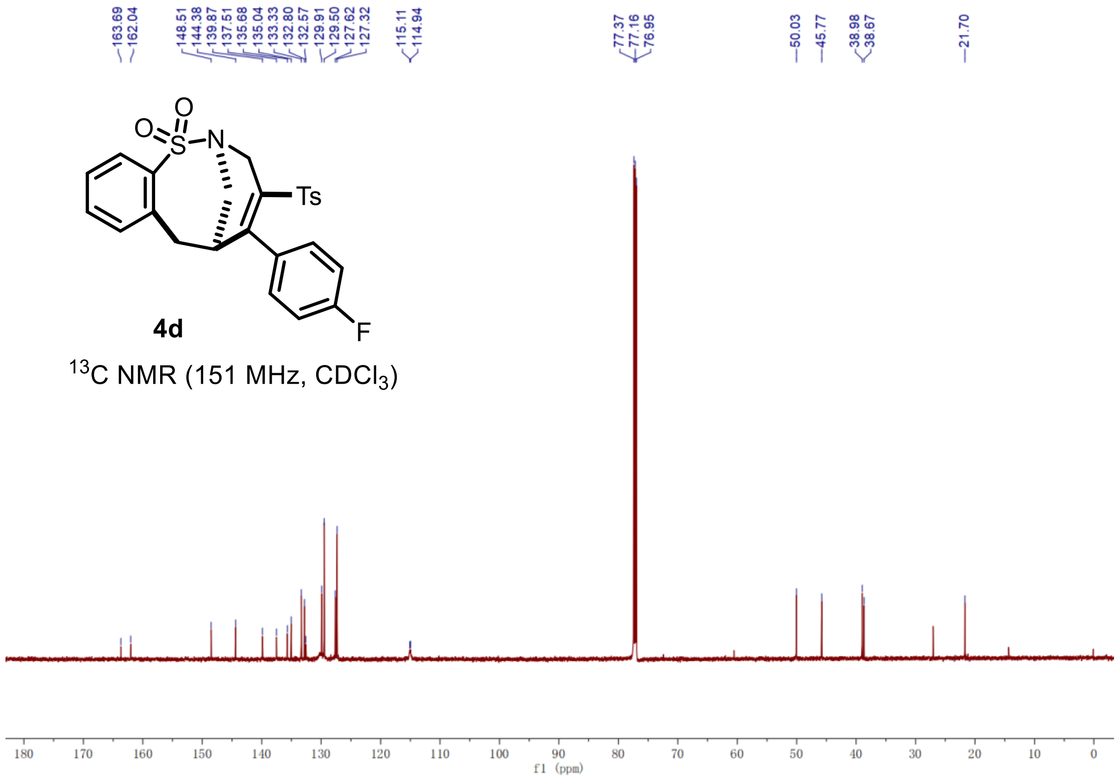
163.69
162.04
148.51
144.38
139.87
137.51
135.68
135.04
133.33
132.80
132.57
129.91
129.50
127.62
127.32
115.11
114.94
77.37
77.16
76.95
50.03
45.77
38.98
38.67
21.70
Ts
F
4d
^{13}C NMR (151 MHz, $CDCl_3$)
f1 (ppm)

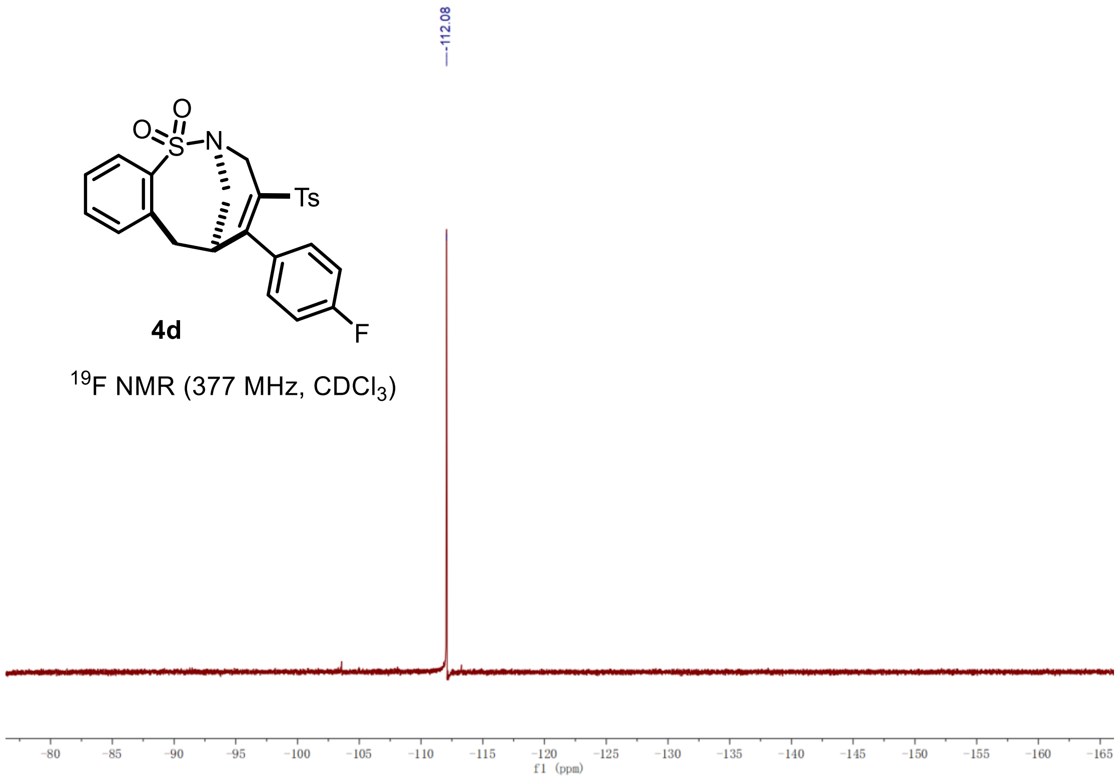
-112.08
Ts
F
4d
^{19}F NMR (377 MHz, $CDCl_3$)
f1 (ppm)

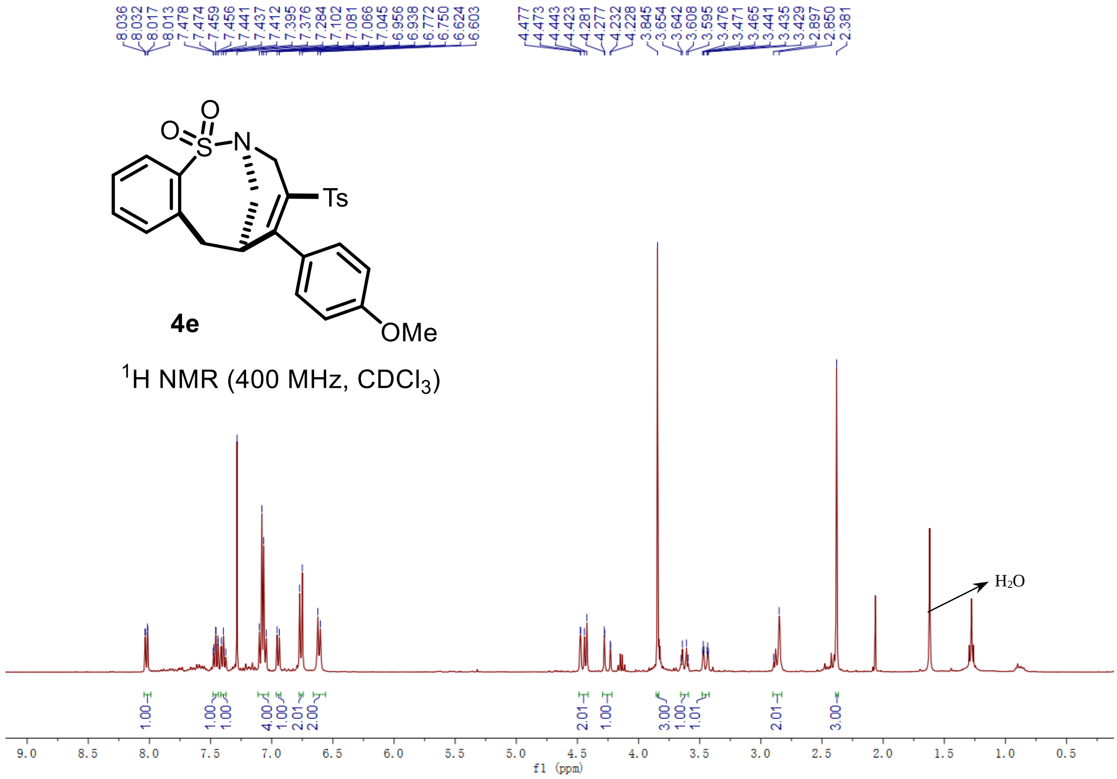
8.036 8.032 8.017 8.013 7.478 7.474 7.459 7.456 7.441 7.437 7.412 7.395 7.376 7.284 7.102 7.081 7.066 7.045 6.956 6.938 6.772 6.750 6.624 6.603
4.477 4.473 4.443 4.423 4.281 4.277 4.232 4.228 3.845 3.654 3.642 3.608 3.595 3.476 3.471 3.465 3.441 3.435 3.429 2.897 2.850 2.381
Ts
OMe
4e
1H NMR (400 MHz, CDCl3)
H2O
1.00 1.00 1.00 4.00 1.00 2.01 2.00 2.01 1.00 3.00 1.00 1.01 2.01 3.00
f1 (ppm)

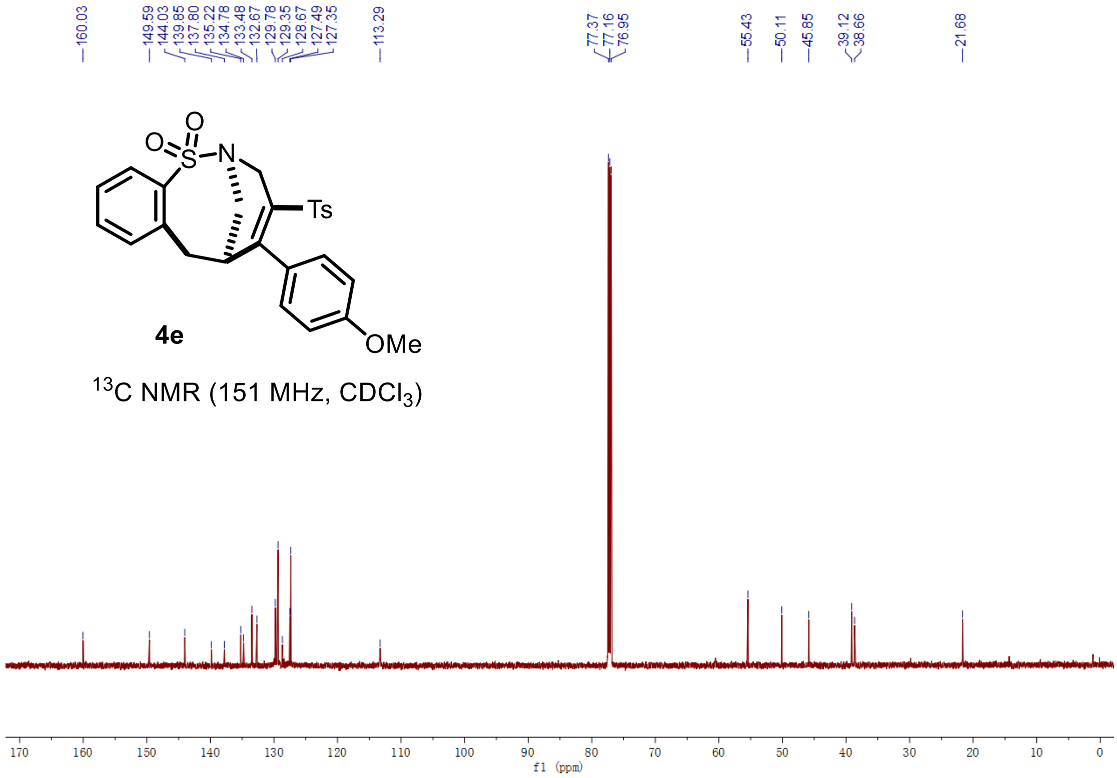
160.03 149.59 144.03 139.85 137.80 135.22 134.78 133.48 132.67 129.78 129.35 128.67 127.49 127.35 113.29
77.37 77.16 76.95 55.43 50.11 45.85 39.12 38.66 21.68
Ts
OMe
4e
13C NMR (151 MHz, CDCl3)
f1 (ppm)

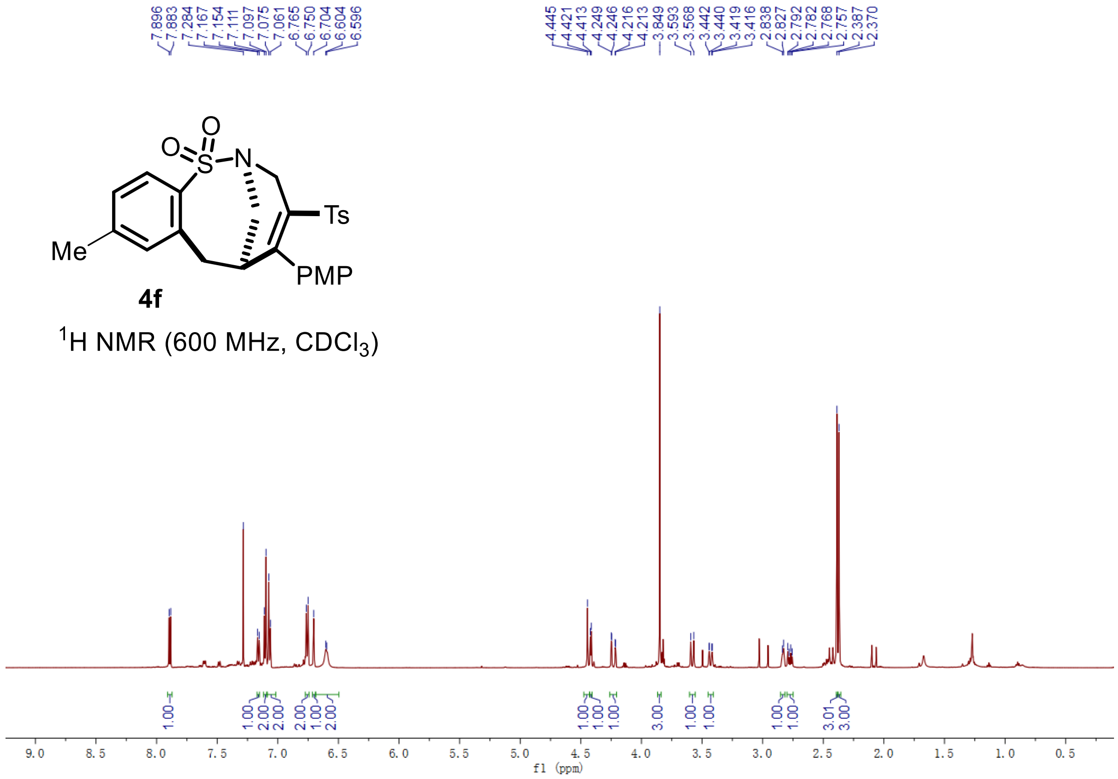
7.896
7.883
7.284
7.167
7.154
7.111
7.097
7.075
7.061
6.765
6.750
6.704
6.604
6.596
4.445
4.421
4.413
4.249
4.246
4.216
4.213
3.849
3.593
3.568
3.442
3.440
3.419
3.416
2.838
2.827
2.792
2.782
2.768
2.757
2.387
2.370
O
O
S
N
Ts
Me
PMP
4f
1H NMR (600 MHz, CDCl3)
1.00
1.00
2.00
2.00
2.00
1.00
2.00
1.00
1.00
1.00
3.00
1.00
1.00
1.00
1.00
3.01
3.00
f1 (ppm)

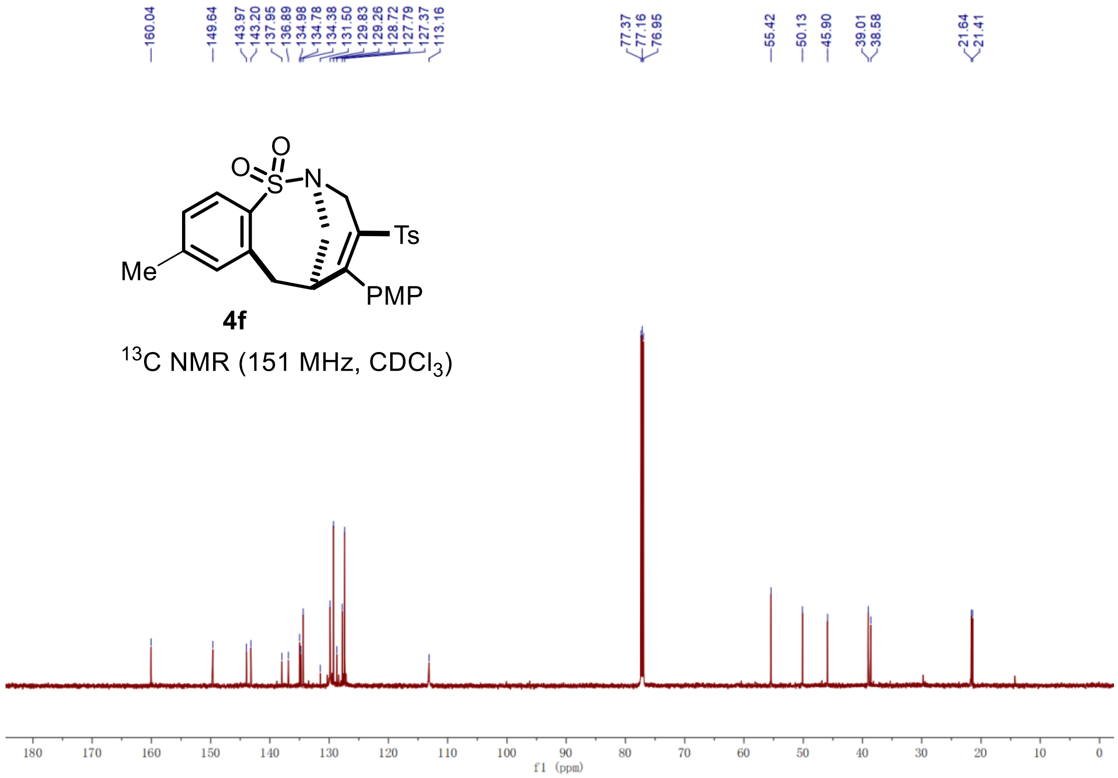
160.04
149.64
143.97
143.20
137.95
136.89
134.98
134.78
134.38
131.50
129.83
129.26
128.72
127.79
127.37
113.16
77.37
77.16
76.95
55.42
50.13
45.90
39.01
38.58
21.64
21.41
O
O
S
N
Ts
Me
PMP
4f
13C NMR (151 MHz, CDCl3)
f1 (ppm)

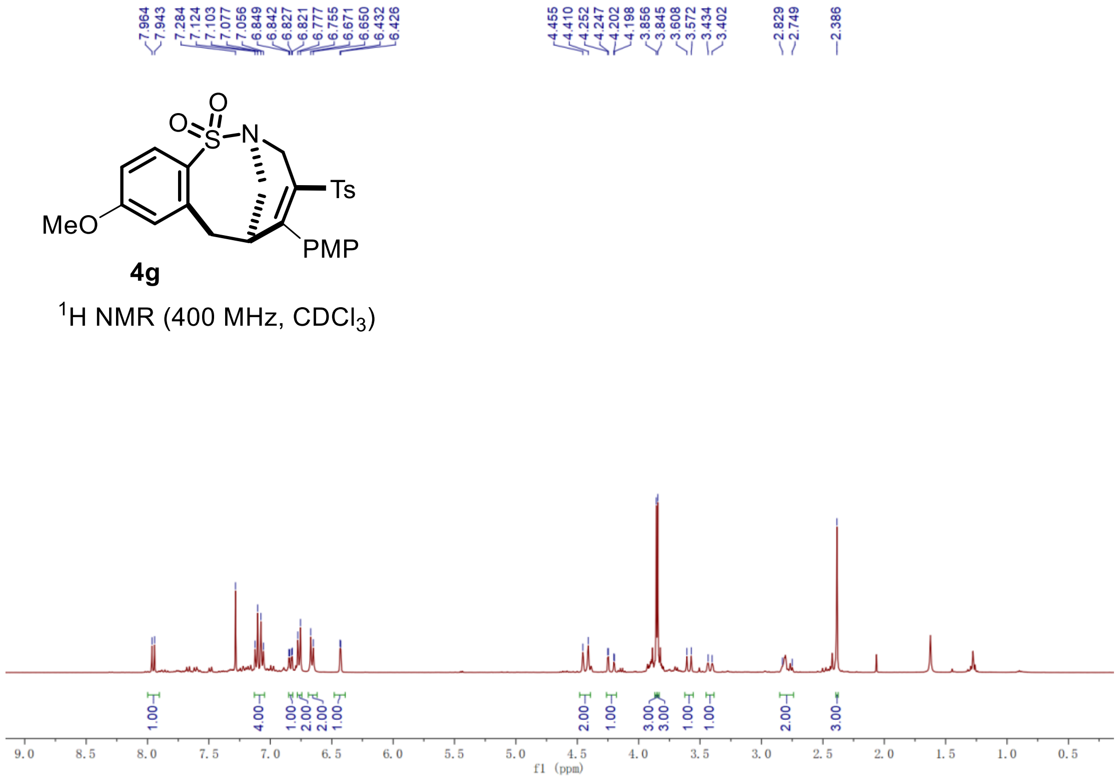
7.964
7.943
7.284
7.124
7.103
7.077
7.056
6.849
6.842
6.827
6.821
6.777
6.755
6.671
6.650
6.432
6.426
4.455
4.410
4.252
4.247
4.202
4.198
3.856
3.845
3.608
3.572
3.434
3.402
2.829
2.749
2.386
MeO
Ts
PMP
4g
^{1}H NMR (400 MHz, $CDCl_3$)
1.00
4.00
1.00
2.00
2.00
1.00
2.00
1.00
3.00
3.00
1.00
1.00
2.00
3.00
f1 (ppm)

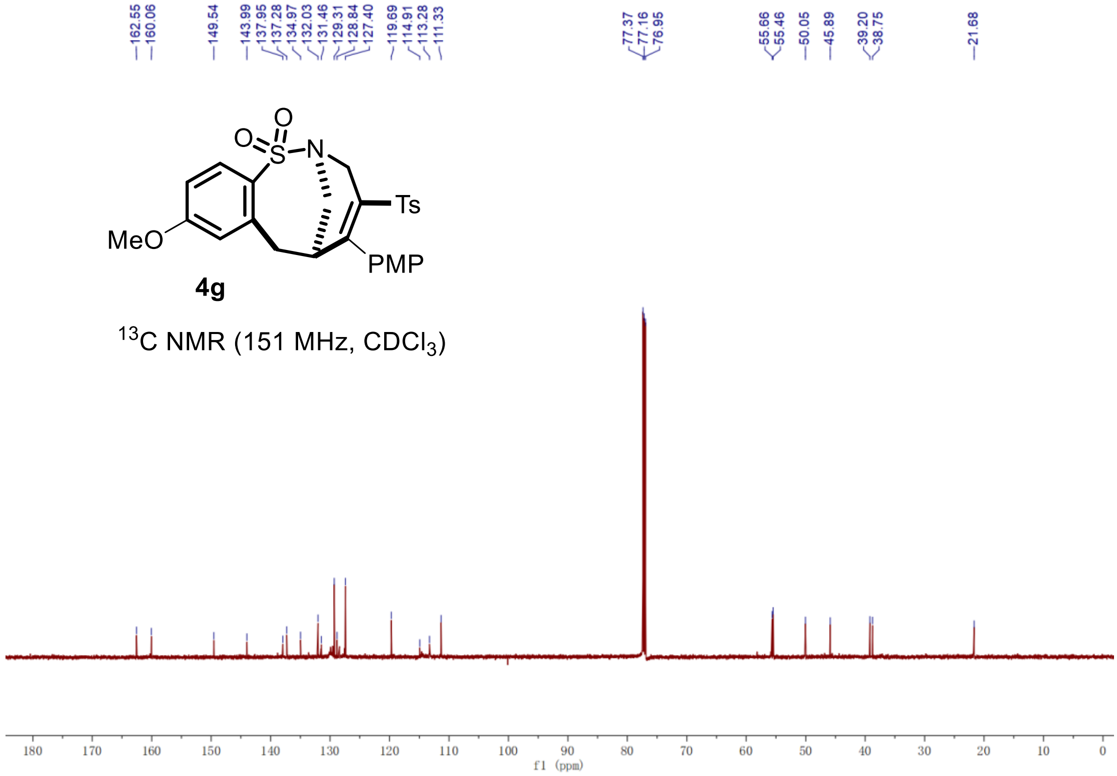
162.55
160.06
149.54
143.99
137.95
137.28
134.97
132.03
131.46
129.31
128.84
127.40
119.69
114.91
113.28
111.33
77.37
77.16
76.95
55.66
55.46
50.05
45.89
39.20
38.75
21.68
MeO
Ts
PMP
4g
^{13}C NMR (151 MHz, $CDCl_3$)
f1 (ppm)

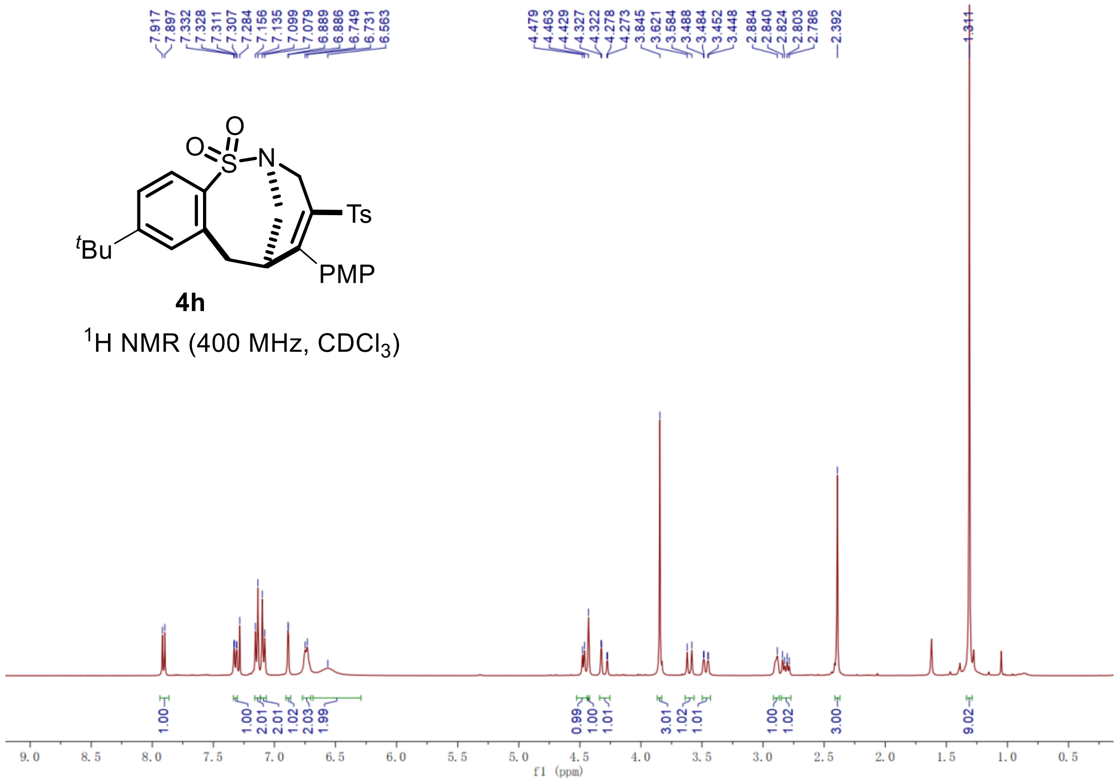
7.917
7.897
7.332
7.328
7.311
7.307
7.284
7.156
7.135
7.099
7.079
6.889
6.886
6.749
6.731
6.563
4.479
4.463
4.429
4.327
4.322
4.278
4.273
3.845
3.621
3.584
3.488
3.484
3.452
3.448
2.884
2.840
2.824
2.803
2.786
2.392
1.311
Ts
^{t}Bu
PMP
4h
^{1}H NMR (400 MHz, $CDCl_3$)
1.00
1.00
2.01
2.01
1.02
2.03
1.99
0.99
1.00
1.01
3.01
1.02
1.01
1.00
1.02
3.00
9.02
f1 (ppm)

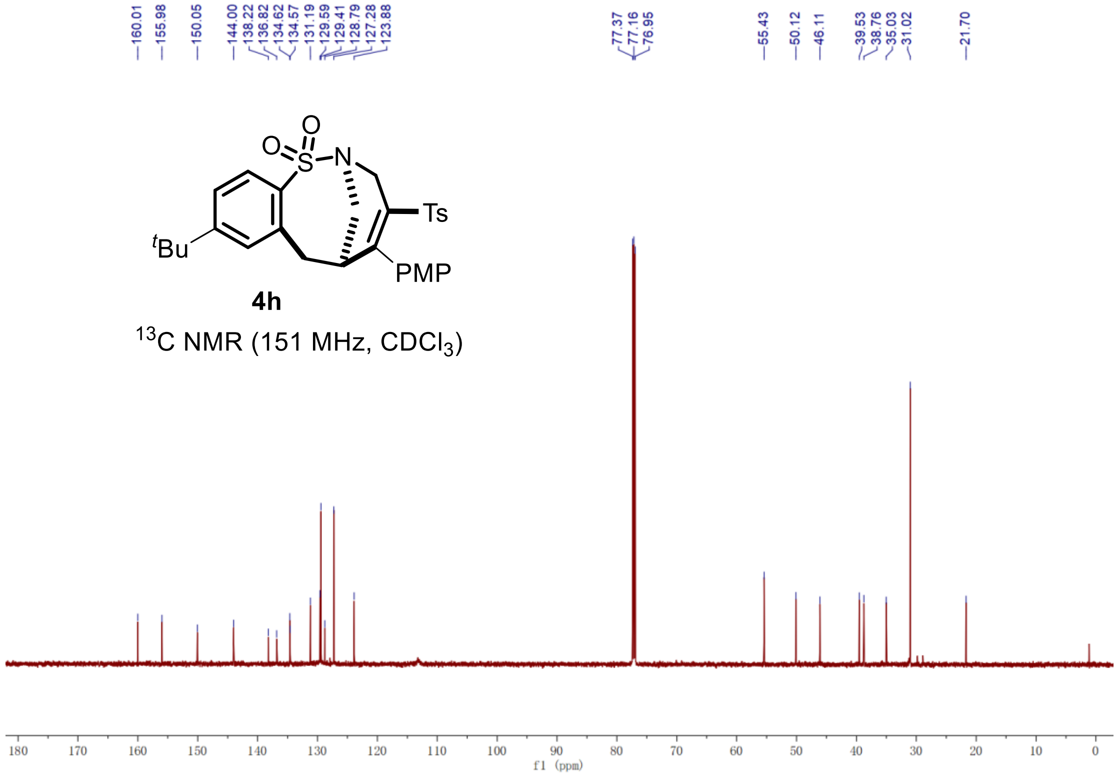
160.01
155.98
150.05
144.00
138.22
136.82
134.62
134.57
131.19
129.59
129.41
128.79
127.28
123.88
77.37
77.16
76.95
55.43
50.12
46.11
39.53
38.76
35.03
31.02
21.70
Ts
^{t}Bu
PMP
4h
^{13}C NMR (151 MHz, $CDCl_3$)
f1 (ppm)

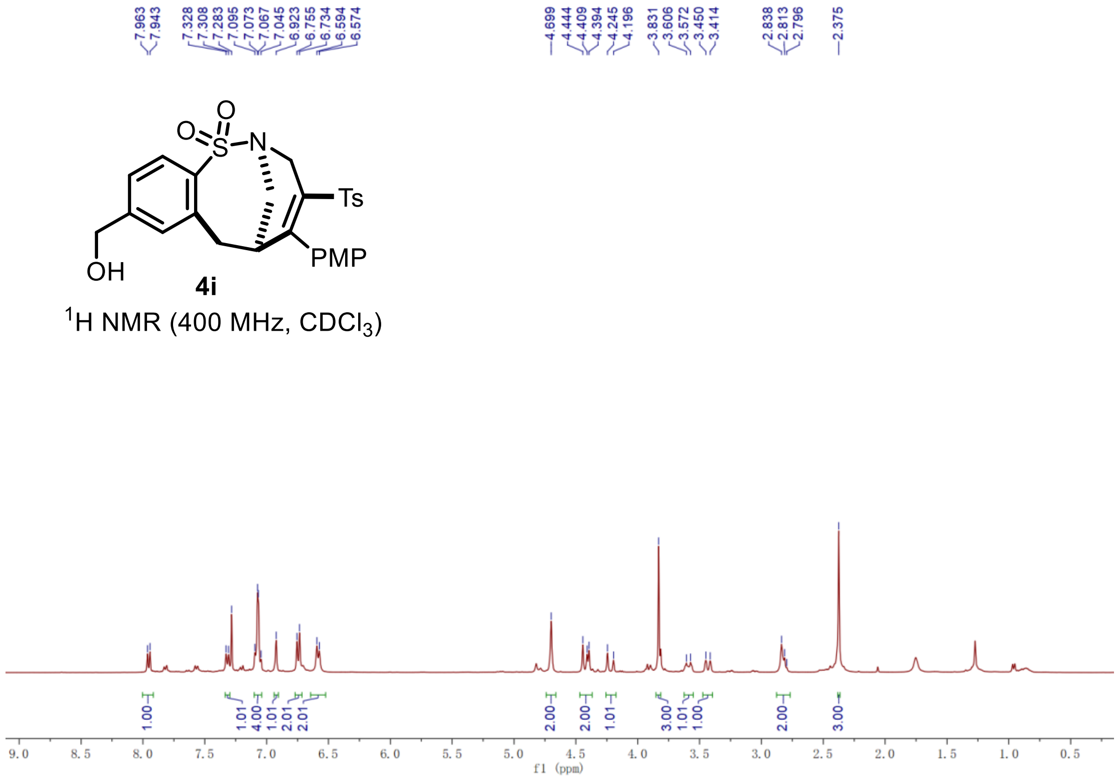
7.963
7.943
7.328
7.308
7.283
7.095
7.073
7.067
7.045
6.923
6.755
6.734
6.594
6.574
4.699
4.444
4.409
4.394
4.245
4.196
3.831
3.606
3.572
3.450
3.414
2.838
2.813
2.796
2.375
O
O
S
N
Ts
PMP
OH
4i
^{1}H NMR (400 MHz, $CDCl_3$)
1.00
1.01
4.00
1.01
2.01
2.01
2.00
2.00
1.01
3.00
1.01
1.00
2.00
3.00
f1 (ppm)

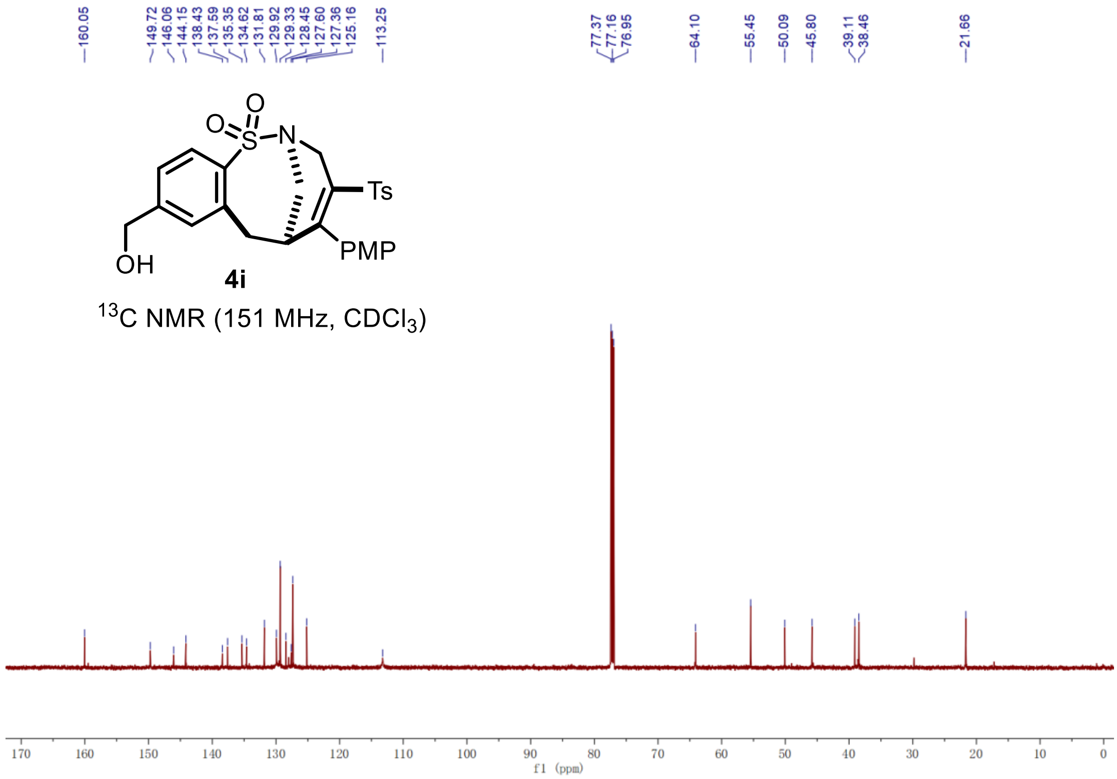
160.05
149.72
146.06
144.15
138.43
137.59
135.35
134.62
131.81
129.92
129.33
128.45
127.60
127.36
125.16
113.25
77.37
77.16
76.95
64.10
55.45
50.09
45.80
39.11
38.46
21.66
O
O
S
N
Ts
PMP
OH
4i
^{13}C NMR (151 MHz, $CDCl_3$)
f1 (ppm)

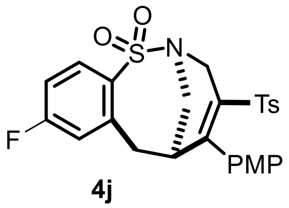

4j

^{1}H NMR (400 MHz, $CDCl_3$)

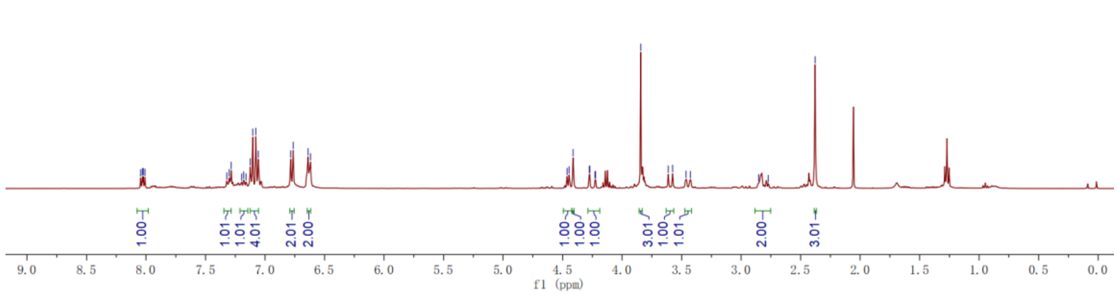

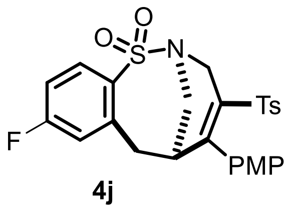

4j

^{13}C NMR (151 MHz, $CDCl_3$)

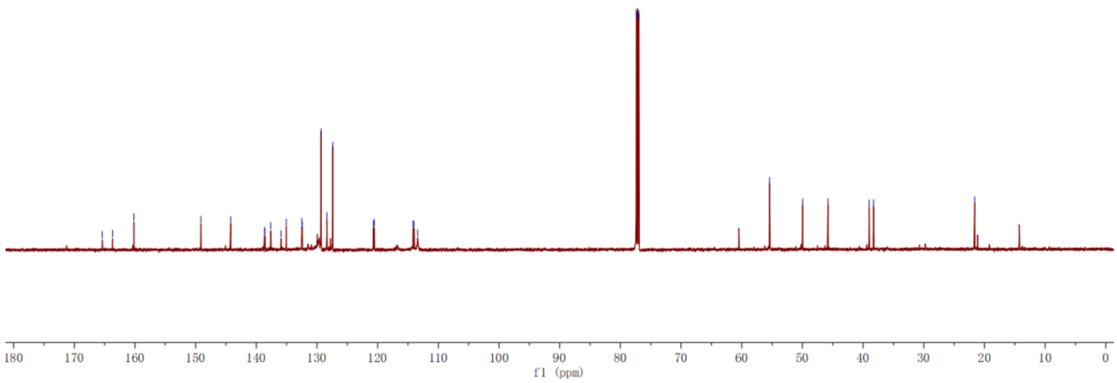

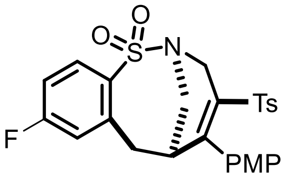

4j

^{19}F NMR (565 MHz, $CDCl_3$)

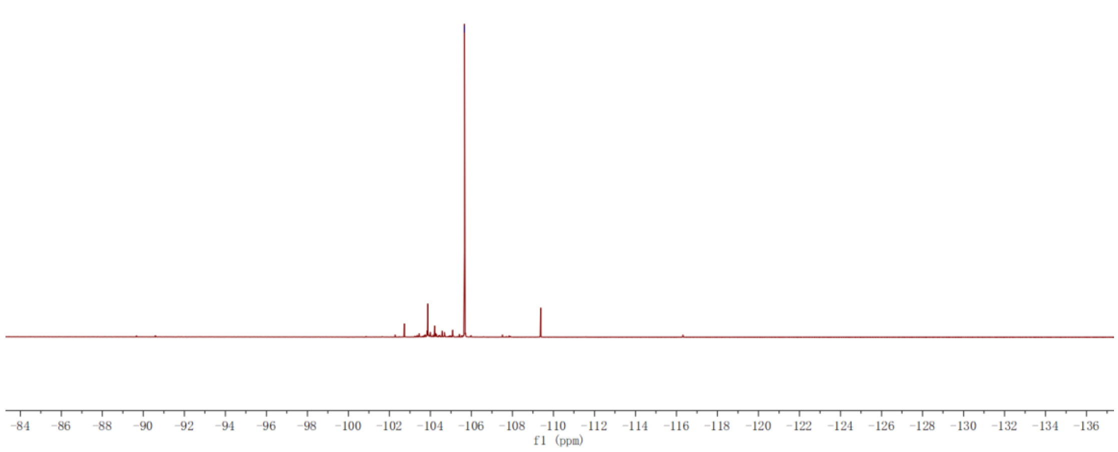

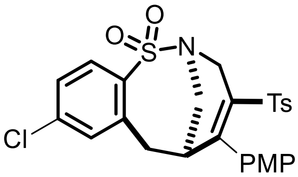

4k

^{1}H NMR (600 MHz, $CDCl_3$)

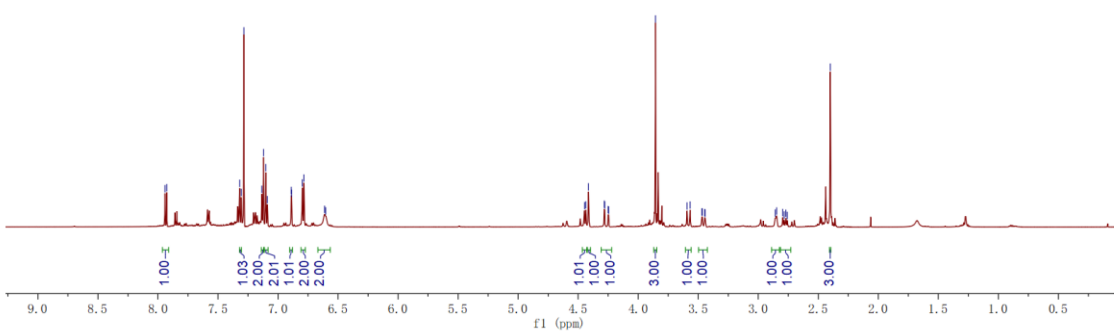

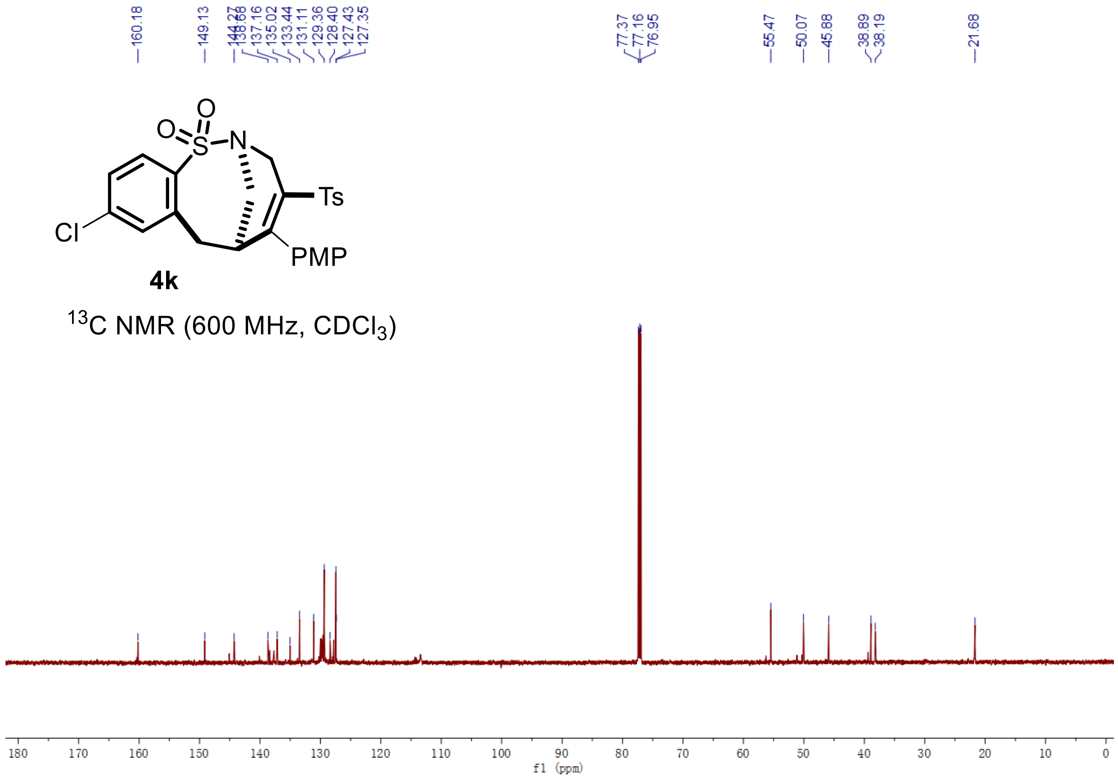
160.18
149.13
144.27
137.16
135.02
133.44
131.11
129.36
128.40
127.43
127.35
77.37
77.16
76.95
55.47
50.07
45.88
38.89
38.19
21.68
O
O
S
N
Ts
Cl
PMP
4k
13C NMR (600 MHz, CDCl3)
f1 (ppm)

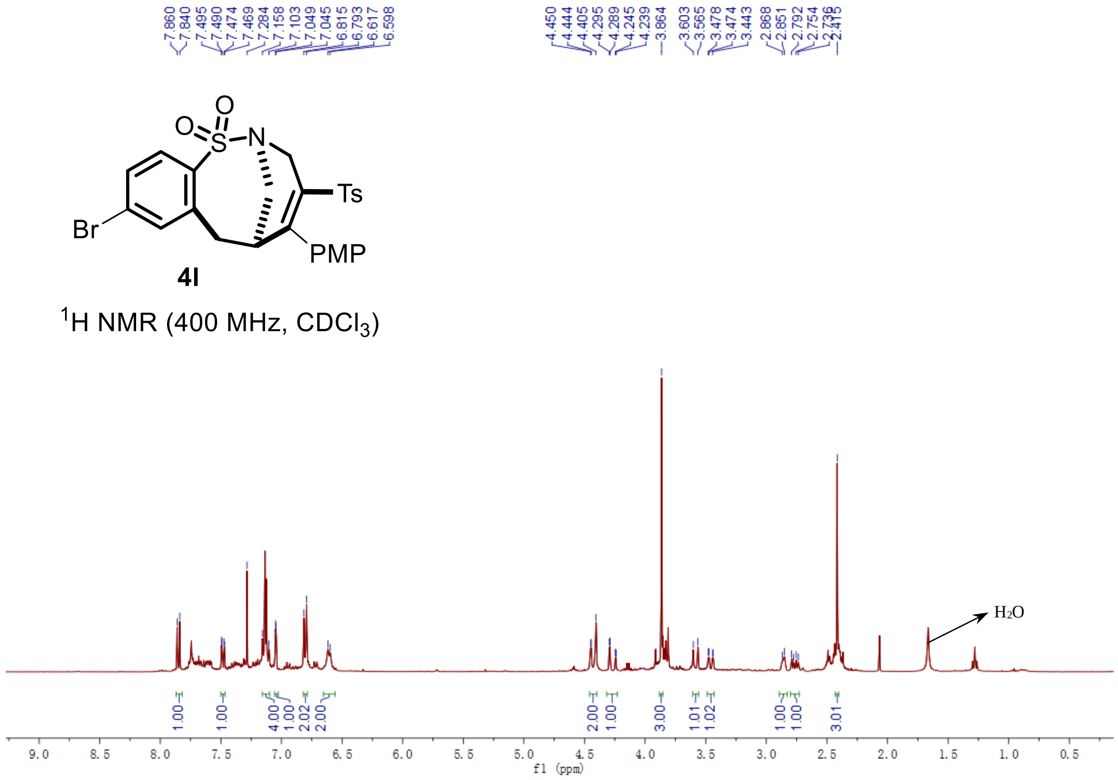
7.860
7.840
7.495
7.490
7.474
7.469
7.284
7.158
7.103
7.049
7.045
6.815
6.793
6.617
6.598
4.450
4.444
4.405
4.295
4.289
4.245
4.239
3.864
3.603
3.565
3.478
3.474
3.443
2.868
2.851
2.792
2.754
2.415
O
O
S
N
Ts
Br
PMP
4l
1H NMR (400 MHz, CDCl3)
H2O
1.00
1.00
4.00
1.00
2.02
2.00
2.00
1.00
3.00
1.01
1.02
1.00
1.00
3.01
f1 (ppm)

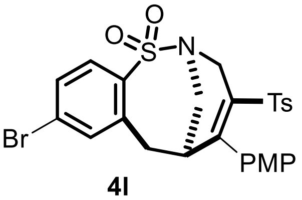

4l

^{13}C NMR (101 MHz, $CDCl_3$)

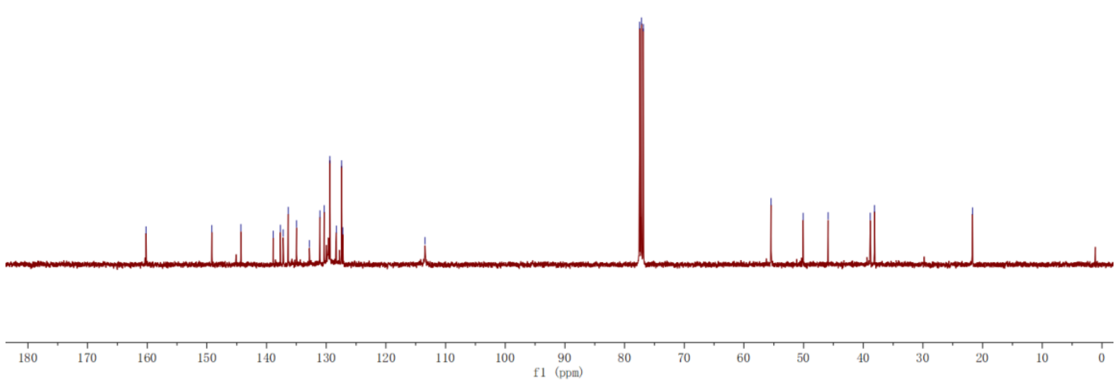

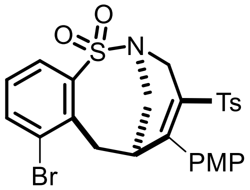

4m

^{1}H NMR (600 MHz, $CDCl_3$)

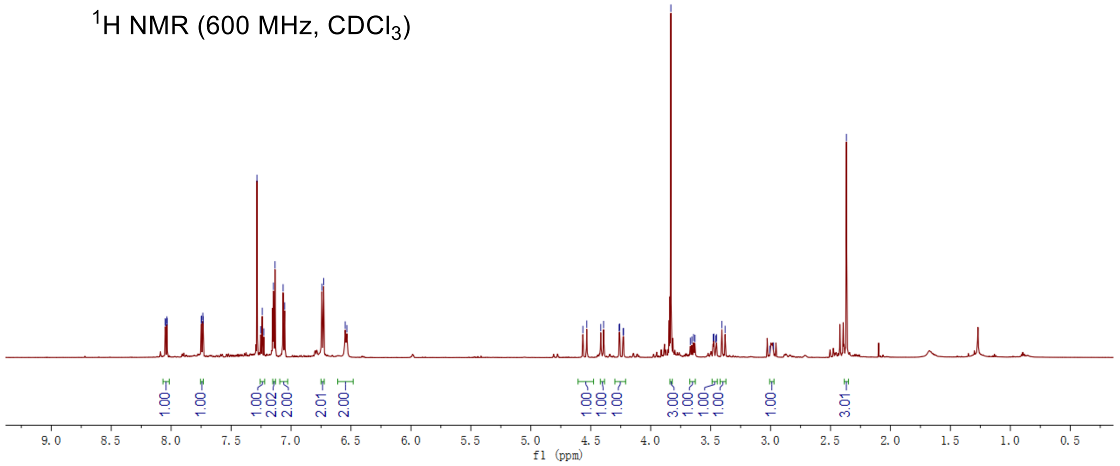

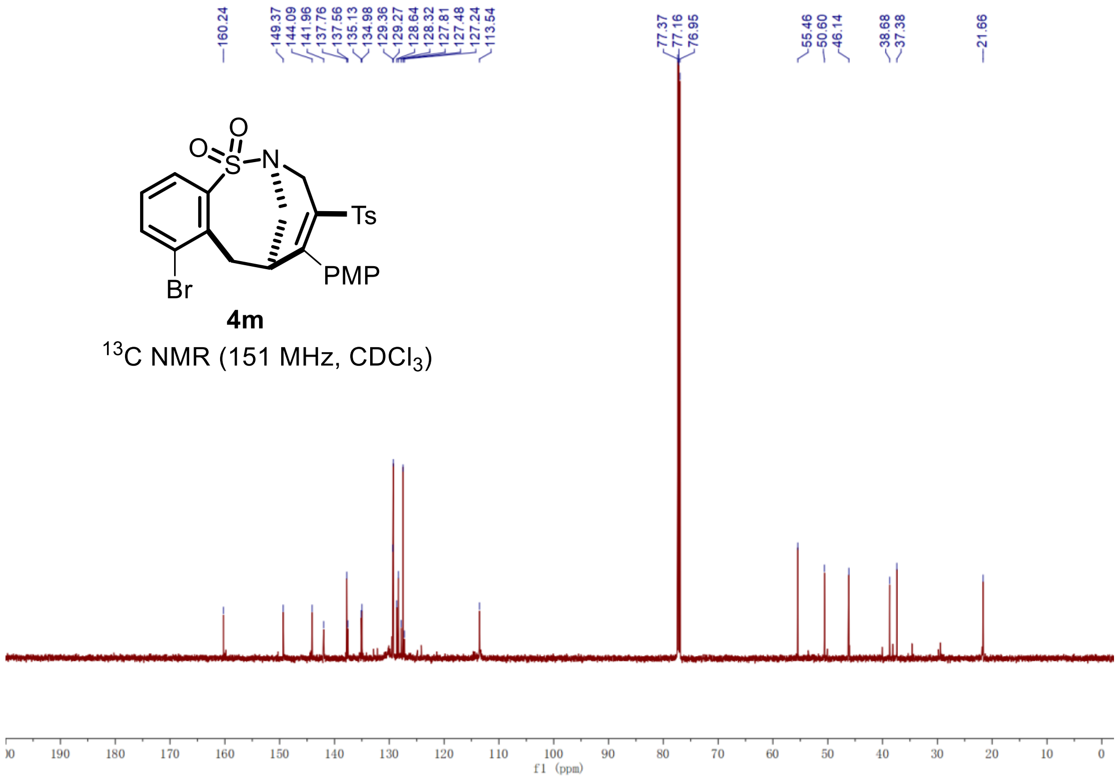
160.24
149.37
144.09
141.96
137.76
137.56
135.13
134.98
129.36
129.27
128.64
128.32
127.81
127.48
127.24
113.54
77.37
77.16
76.95
55.46
50.60
46.14
38.68
37.38
21.66
Ts
PMP
Br
4m
^{13}C NMR (151 MHz, $CDCl_3$)
f1 (ppm)

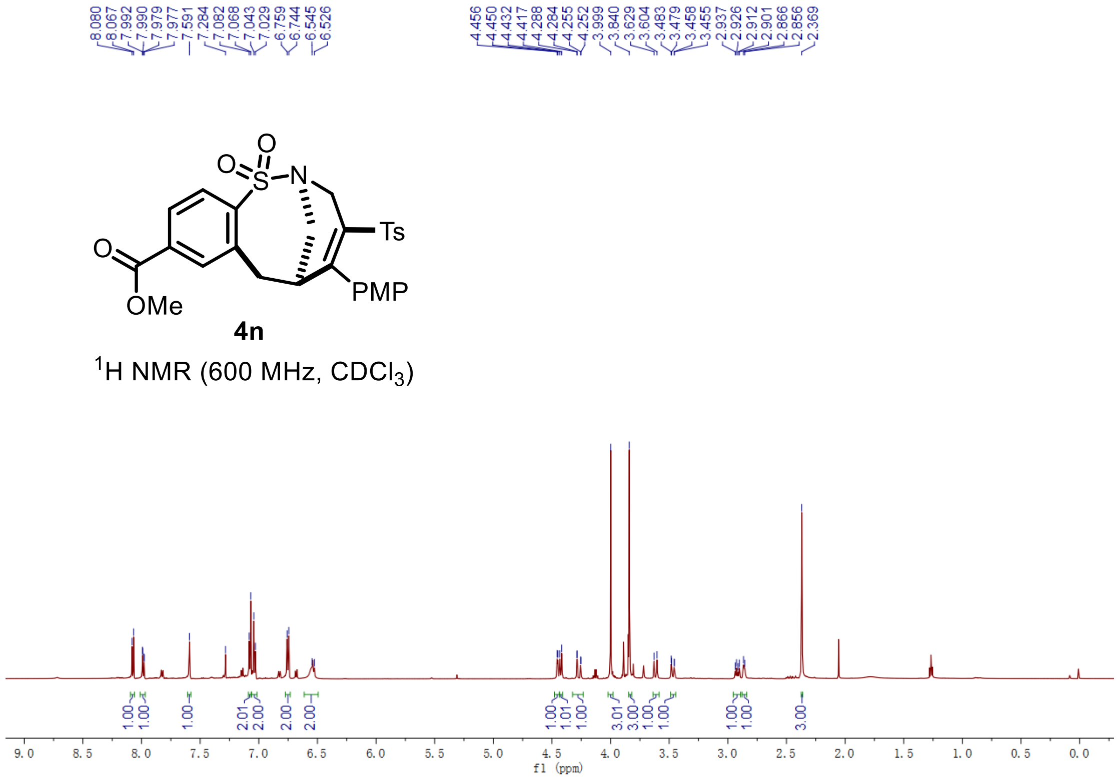
8.080
8.067
7.992
7.990
7.979
7.977
7.591
7.284
7.082
7.068
7.043
7.029
6.759
6.744
6.545
6.526
4.456
4.450
4.432
4.417
4.288
4.284
4.255
4.252
3.999
3.840
3.629
3.604
3.483
3.479
3.458
3.455
2.937
2.926
2.912
2.901
2.866
2.856
2.369
Ts
PMP
OMe
4n
^{1}H NMR (600 MHz, $CDCl_3$)
f1 (ppm)

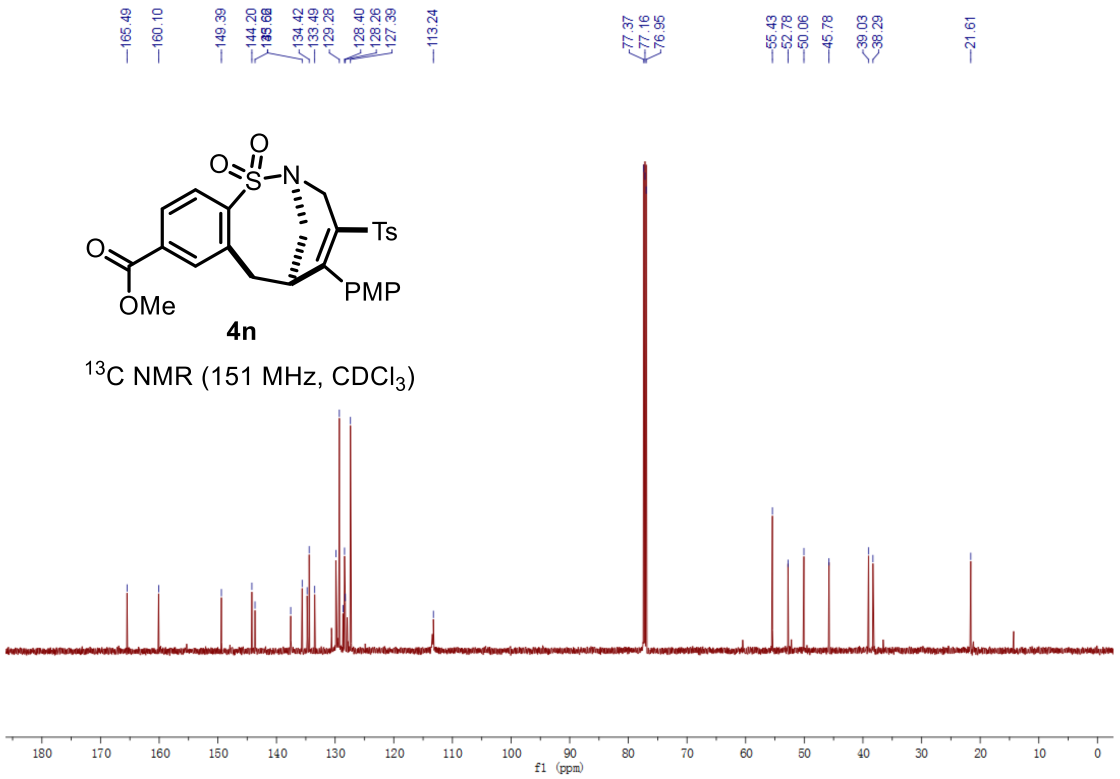
165.49
160.10
149.39
144.20
134.42
133.49
129.28
128.40
128.26
127.39
113.24
77.37
77.16
76.95
55.43
52.78
50.06
45.78
39.03
38.29
21.61
Ts
PMP
OMe
4n
^{13}C NMR (151 MHz, CDCl$_3$)
f1 (ppm)

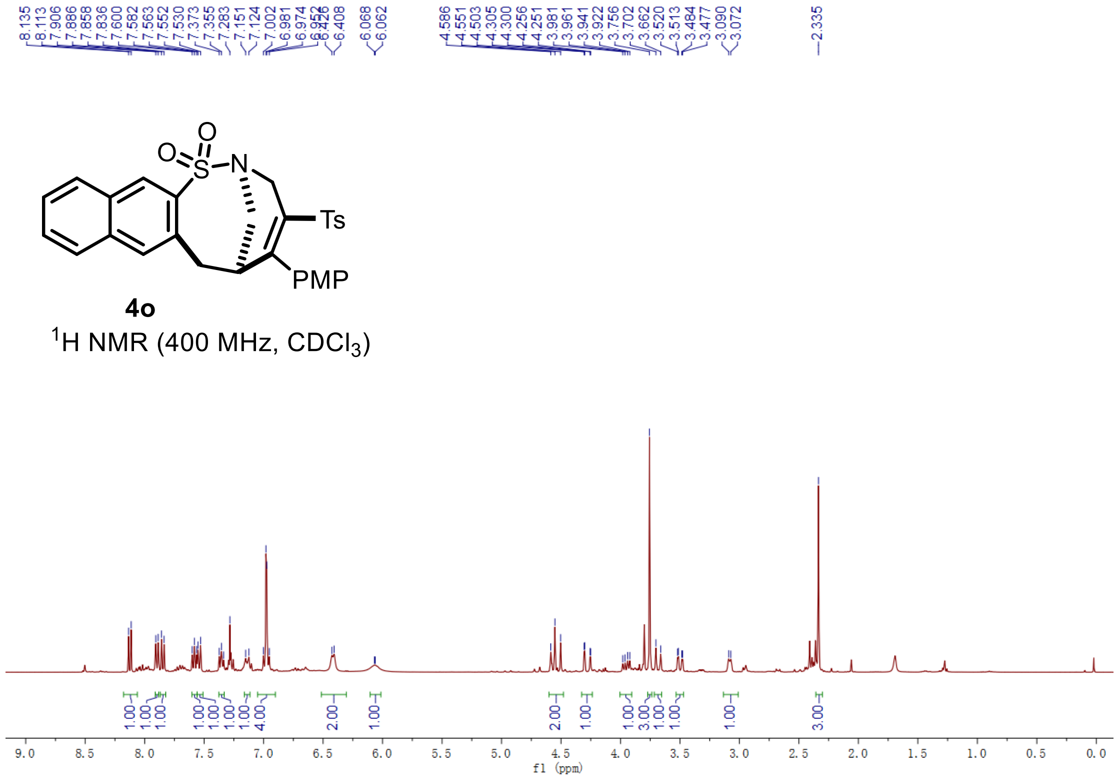
8.135
8.113
7.906
7.886
7.858
7.836
7.600
7.582
7.563
7.552
7.530
7.373
7.355
7.283
7.151
7.124
7.002
6.981
6.974
6.408
6.068
6.062
4.586
4.551
4.503
4.305
4.300
4.256
4.251
3.981
3.961
3.941
3.922
3.756
3.702
3.662
3.520
3.513
3.484
3.477
3.090
3.072
2.335
Ts
PMP
4o
^{1}H NMR (400 MHz, CDCl$_3$)
f1 (ppm)

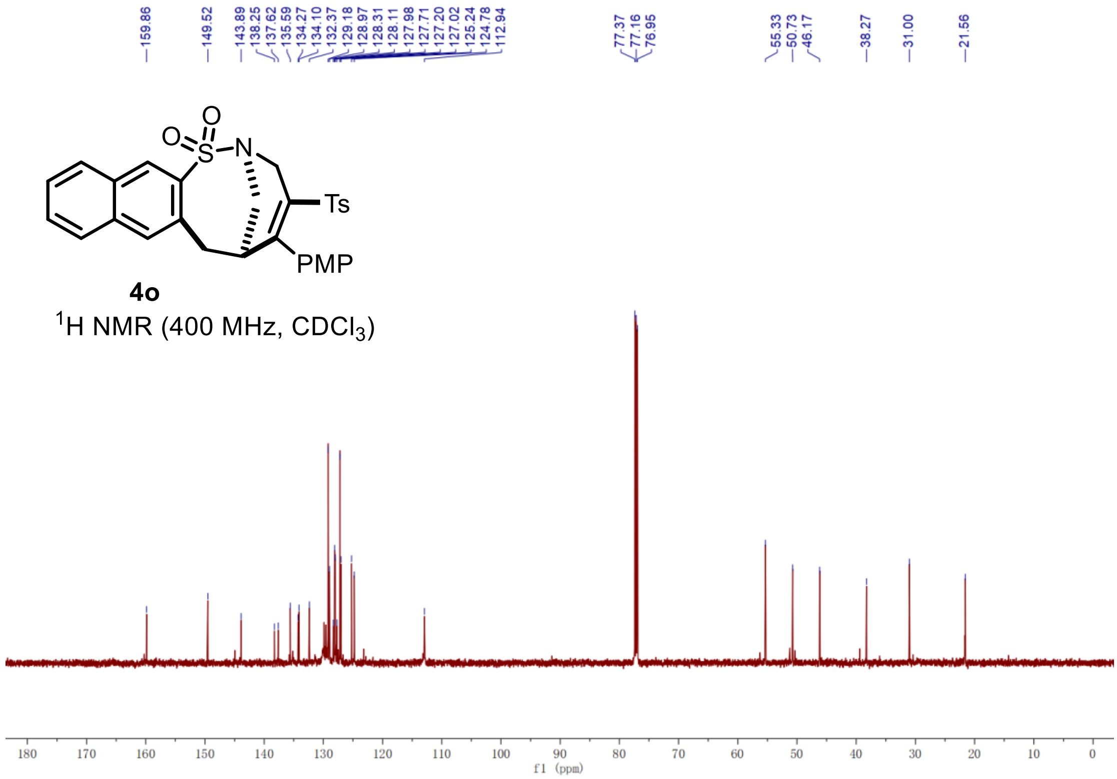

4o

^{1}H NMR (400 MHz, $CDCl_3$)

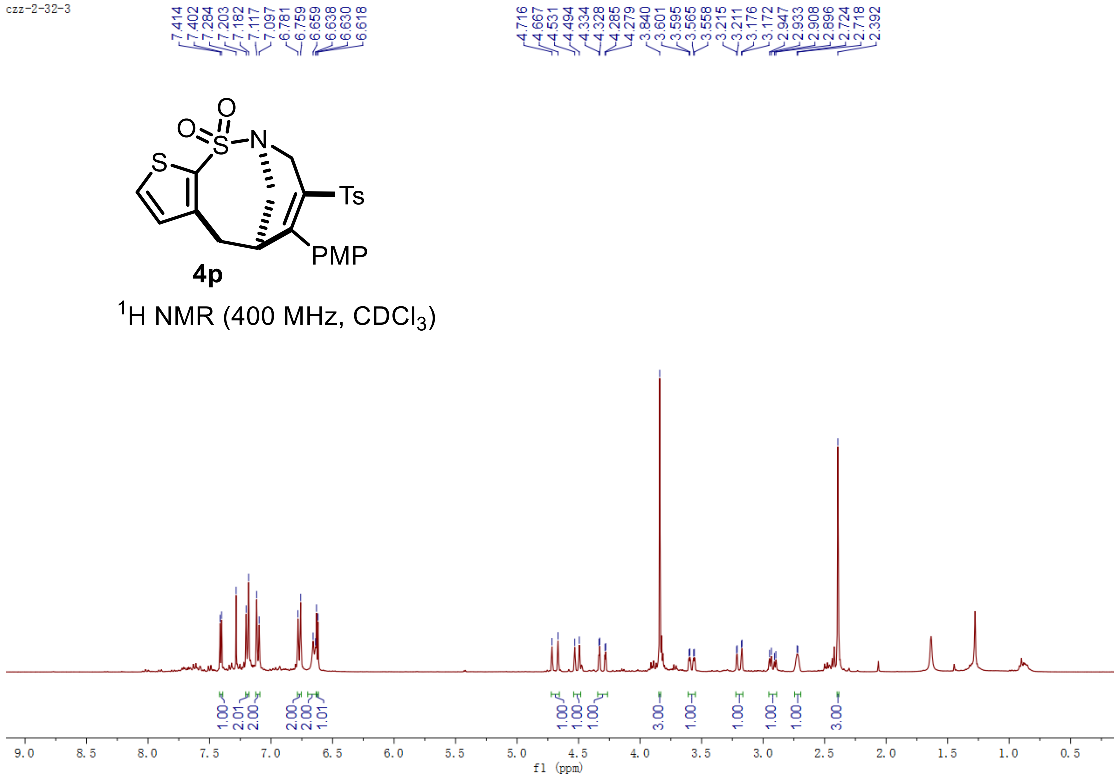

4p

^{1}H NMR (400 MHz, $CDCl_3$)

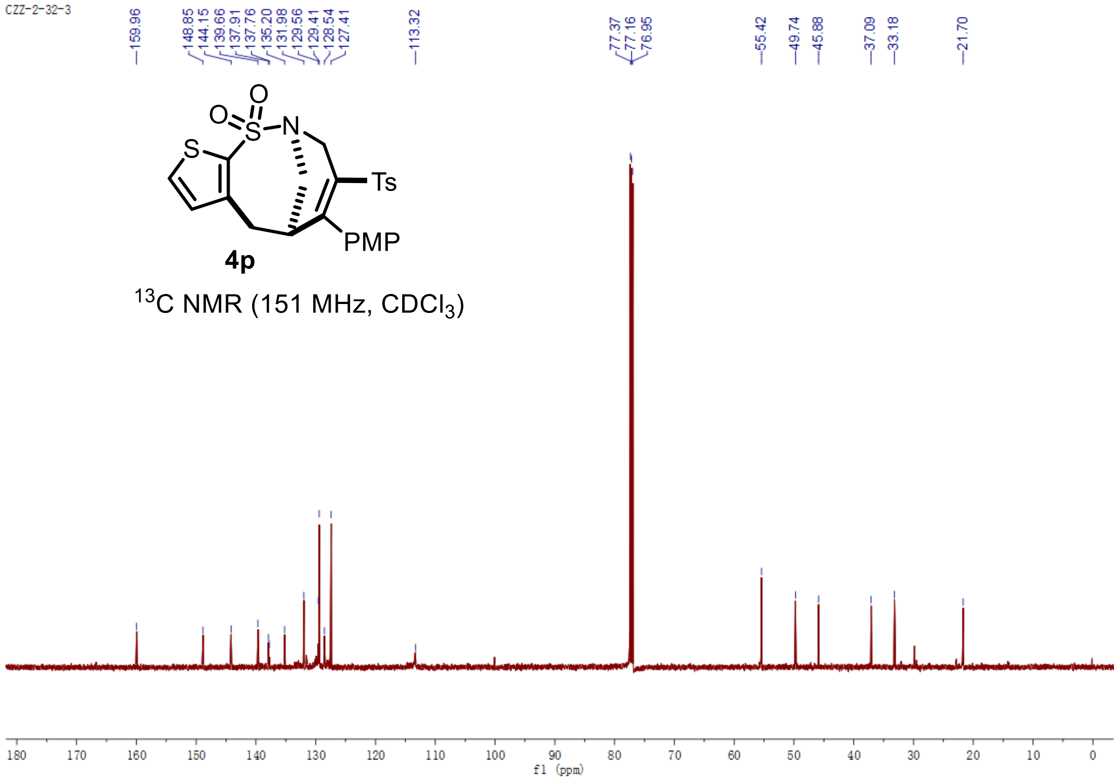

4p

^{13}C NMR (151 MHz, $CDCl_3$)

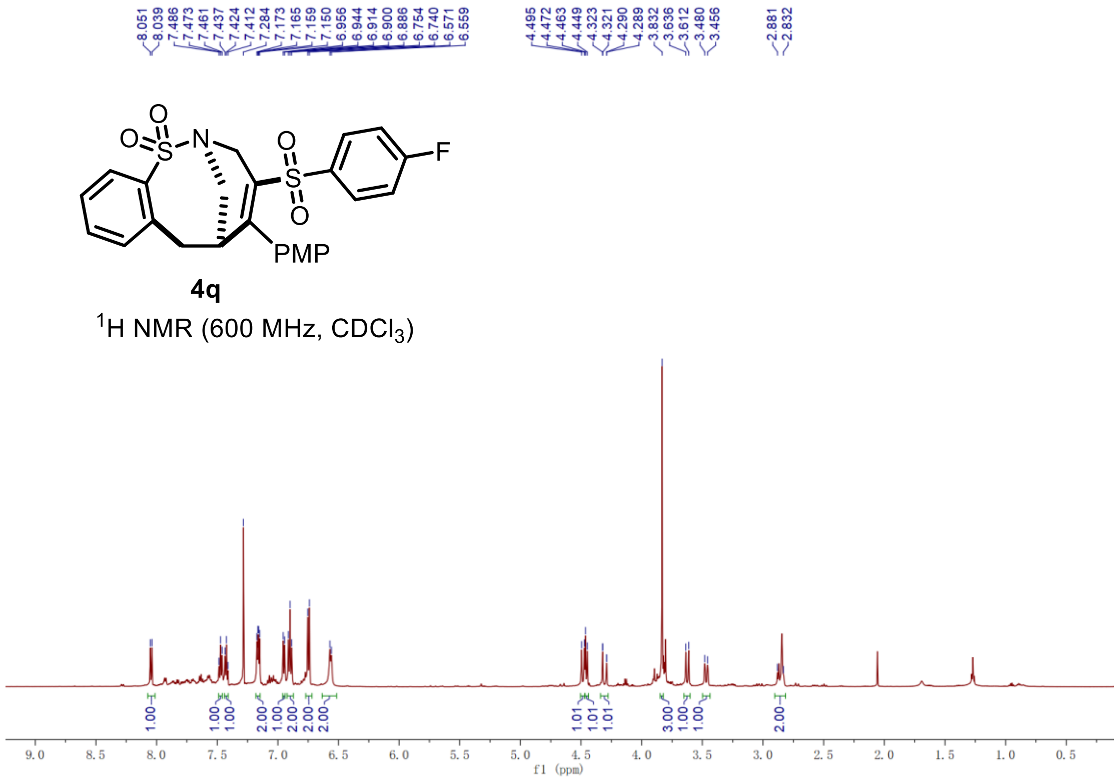

4q

^{1}H NMR (600 MHz, $CDCl_3$)

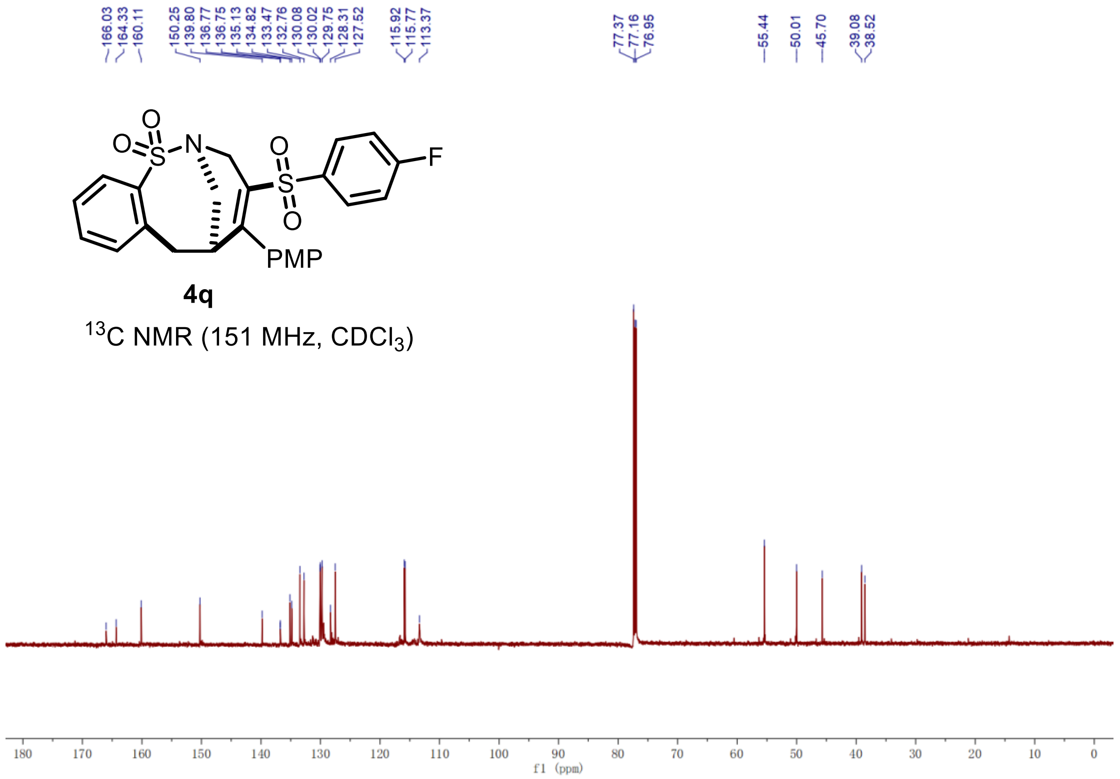
166.03
164.33
160.11
150.25
139.80
136.77
136.75
135.13
134.82
133.47
132.76
130.08
130.02
129.75
128.31
127.52
115.92
115.77
113.37
77.37
77.16
76.95
55.44
50.01
45.70
39.08
38.52
PMP
F
4q
^{13}C NMR (151 MHz, CDCl$_3$)
f1 (ppm)

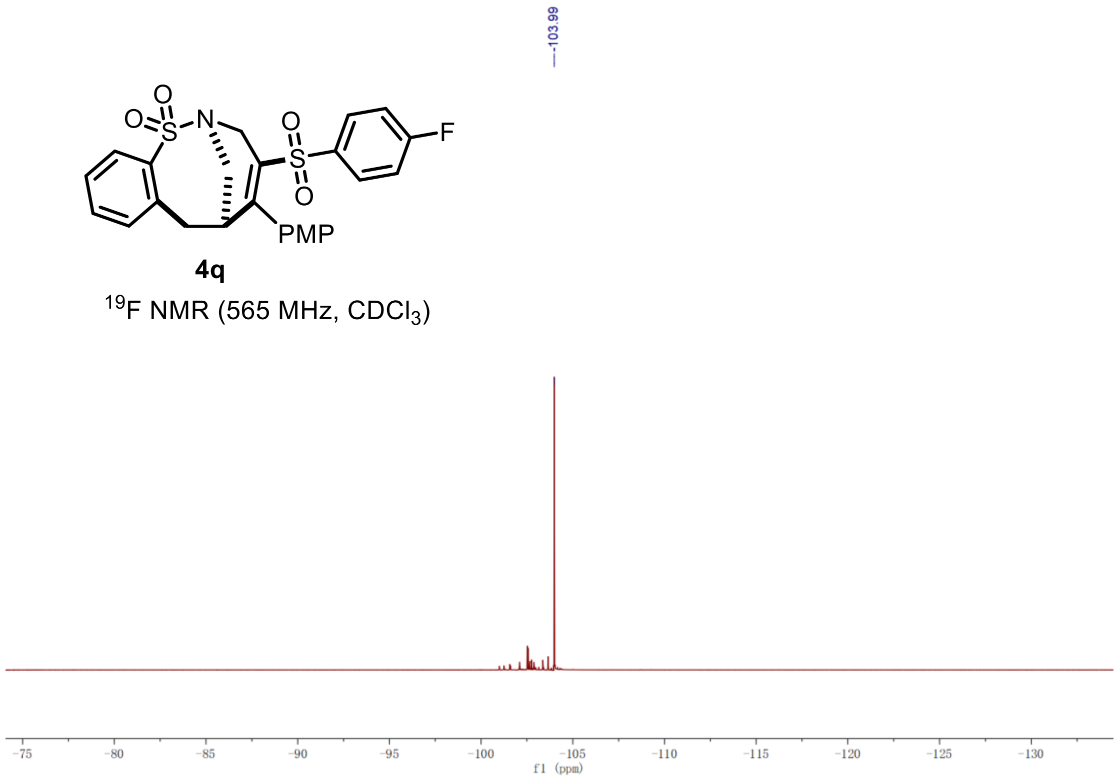
-103.99
PMP
F
4q
^{19}F NMR (565 MHz, CDCl$_3$)
f1 (ppm)

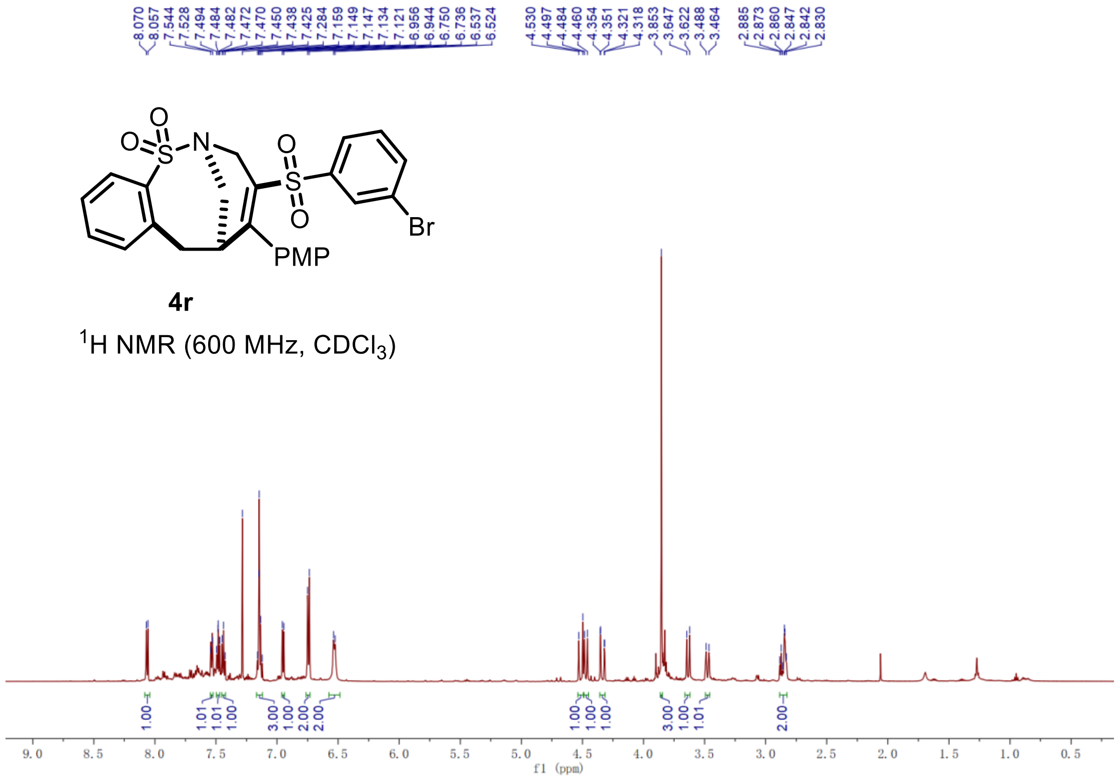
Br
PMP
4r
1H NMR (600 MHz, CDCl3)
f1 (ppm)

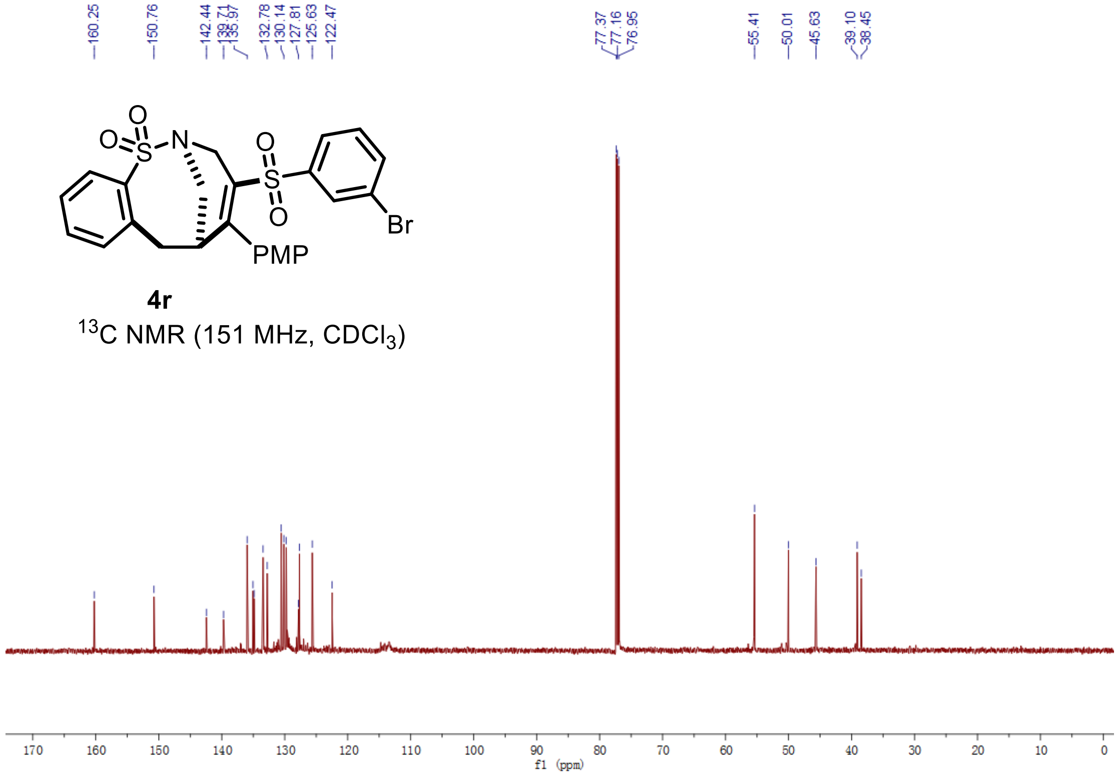
Br
PMP
4r
13C NMR (151 MHz, CDCl3)
f1 (ppm)

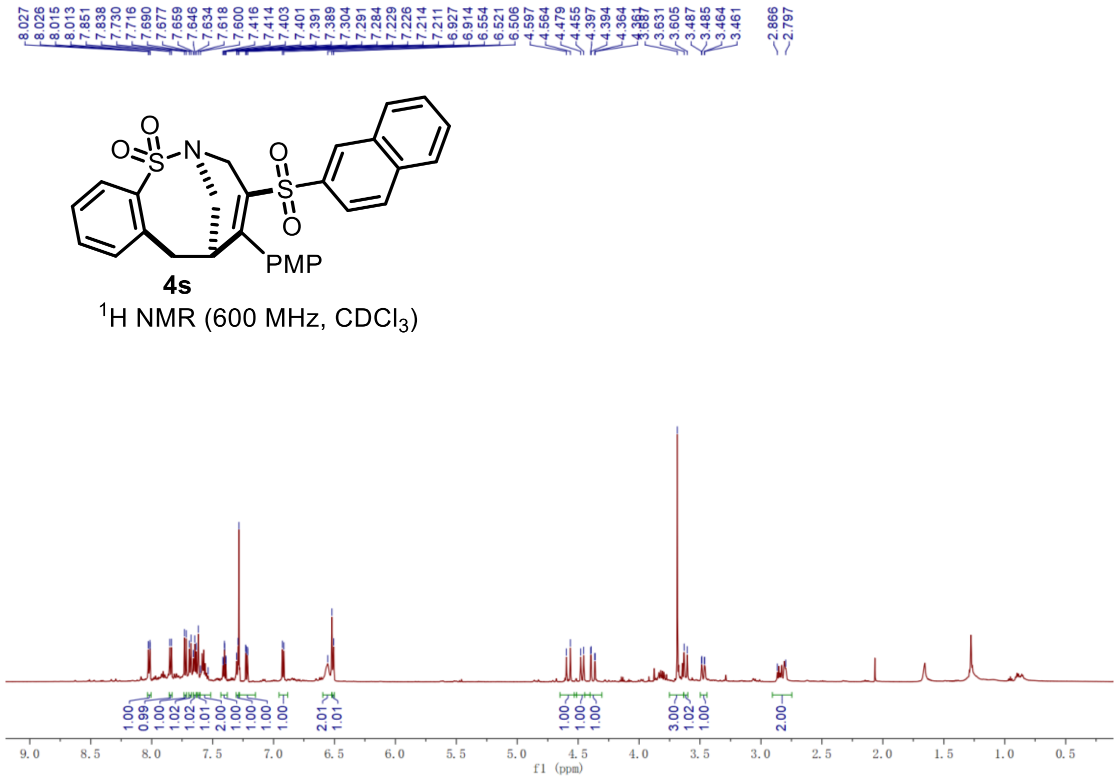
8.027 8.026 8.015 8.013 7.851 7.838 7.730 7.716 7.690 7.677 7.659 7.646 7.634 7.618 7.600 7.416 7.414 7.403 7.401 7.391 7.389 7.304 7.291 7.284 7.229 7.226 7.214 7.211 6.927 6.914 6.554 6.521 6.506 4.597 4.564 4.479 4.455 4.397 4.394 4.364 4.361 3.687 3.631 3.605 3.487 3.485 3.464 3.461 2.866 2.797
O
O
S
N
O
S
O
PMP
4s
^{1}H NMR (600 MHz, CDCl$_3$)
1.00 0.99 1.00 1.02 1.02 1.01 2.00 1.00 1.00 1.00 1.00 2.01 1.01
1.00 1.00 1.00
3.00 1.02 1.00
2.00
f1 (ppm)

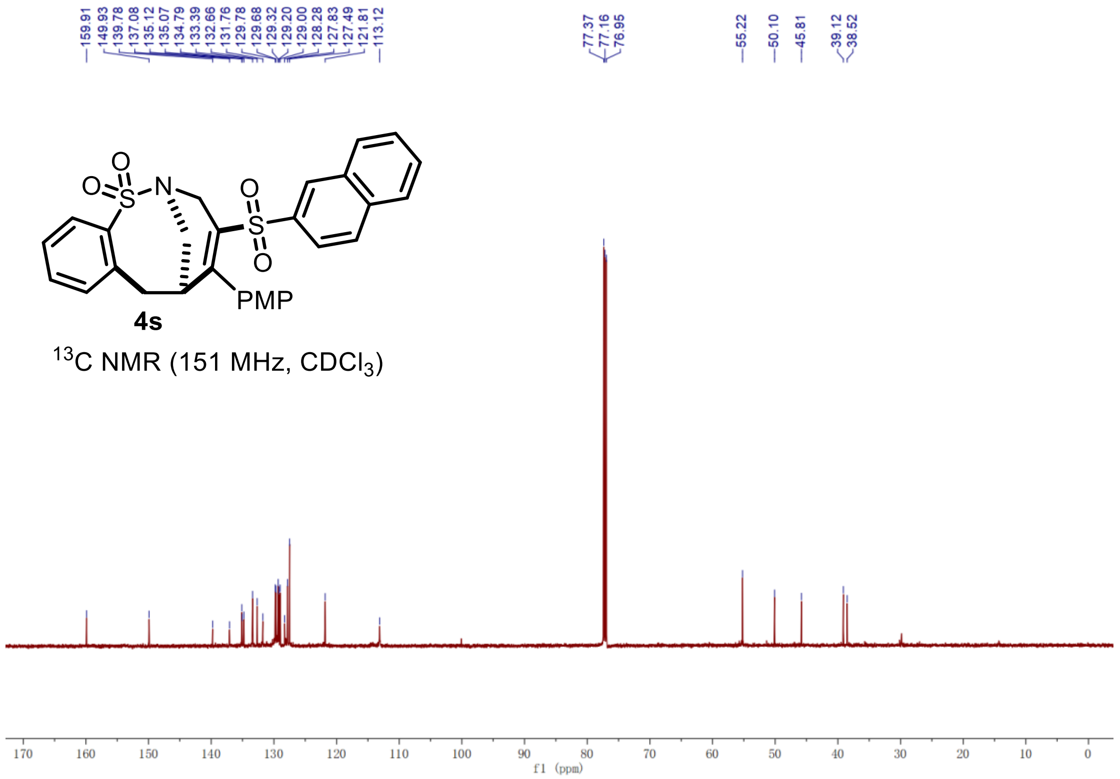
159.91 149.93 139.78 137.08 135.12 135.07 134.79 133.39 132.66 131.76 129.78 129.68 129.32 129.20 129.00 128.28 127.83 127.49 121.81 113.12
77.37 77.16 76.95
55.22
50.10
45.81
39.12 38.52
O
O
S
N
O
S
O
PMP
4s
^{13}C NMR (151 MHz, CDCl$_3$)
f1 (ppm)

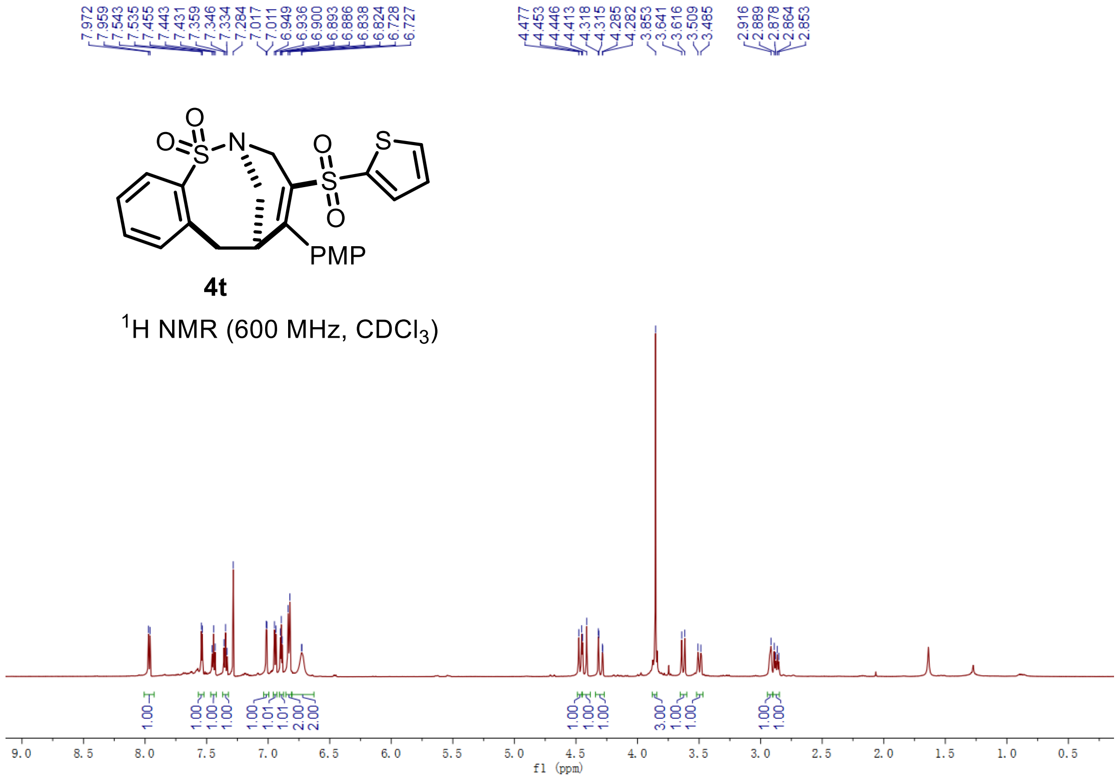
PMP
4t
1H NMR (600 MHz, CDCl3)
f1 (ppm)

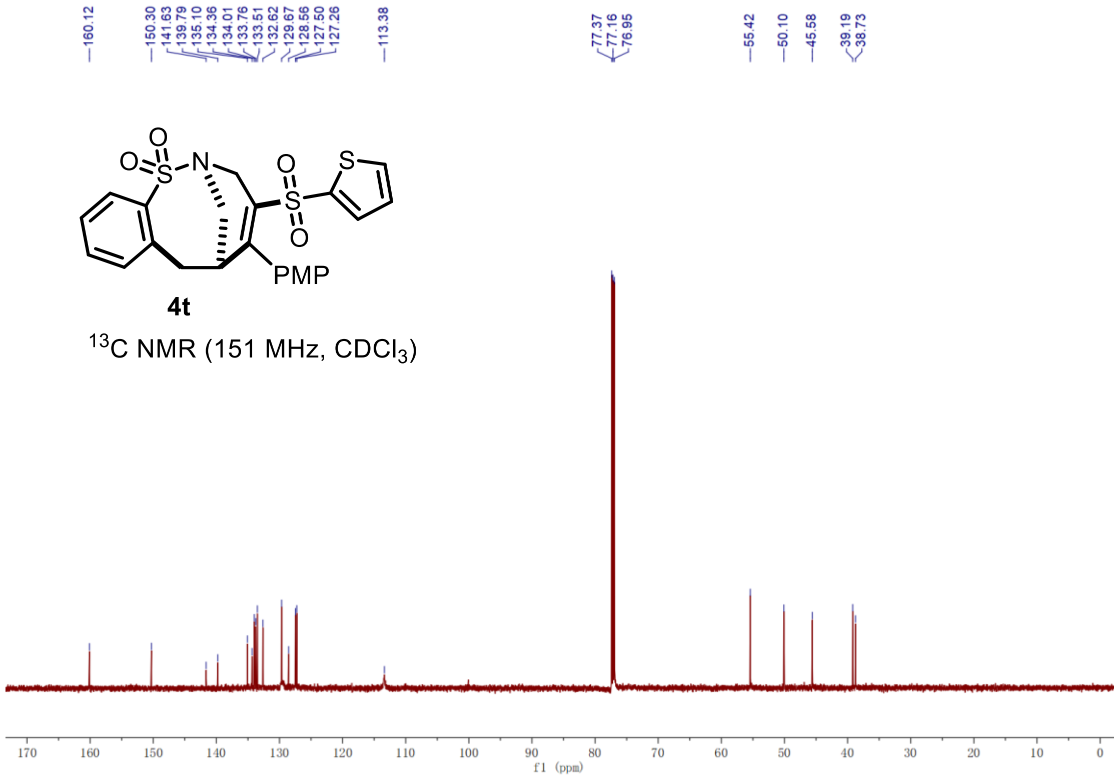
PMP
4t
13C NMR (151 MHz, CDCl3)
f1 (ppm)

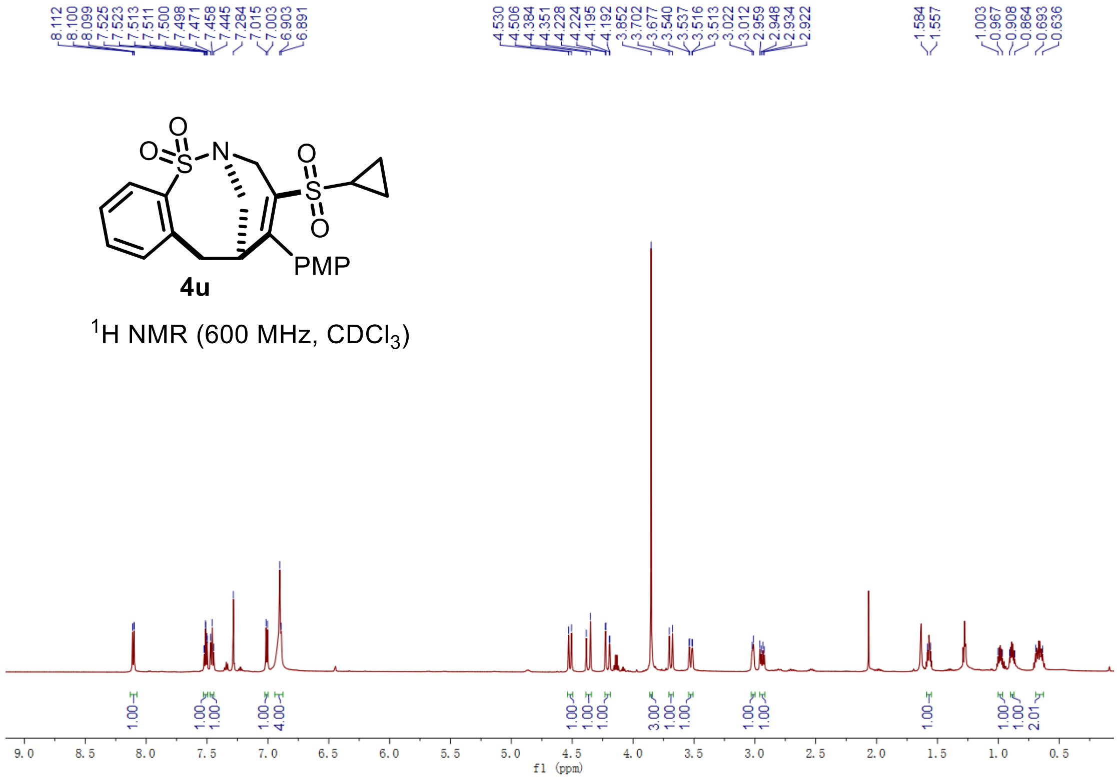

4u
1H NMR (600 MHz, CDCl3)
f1 (ppm)

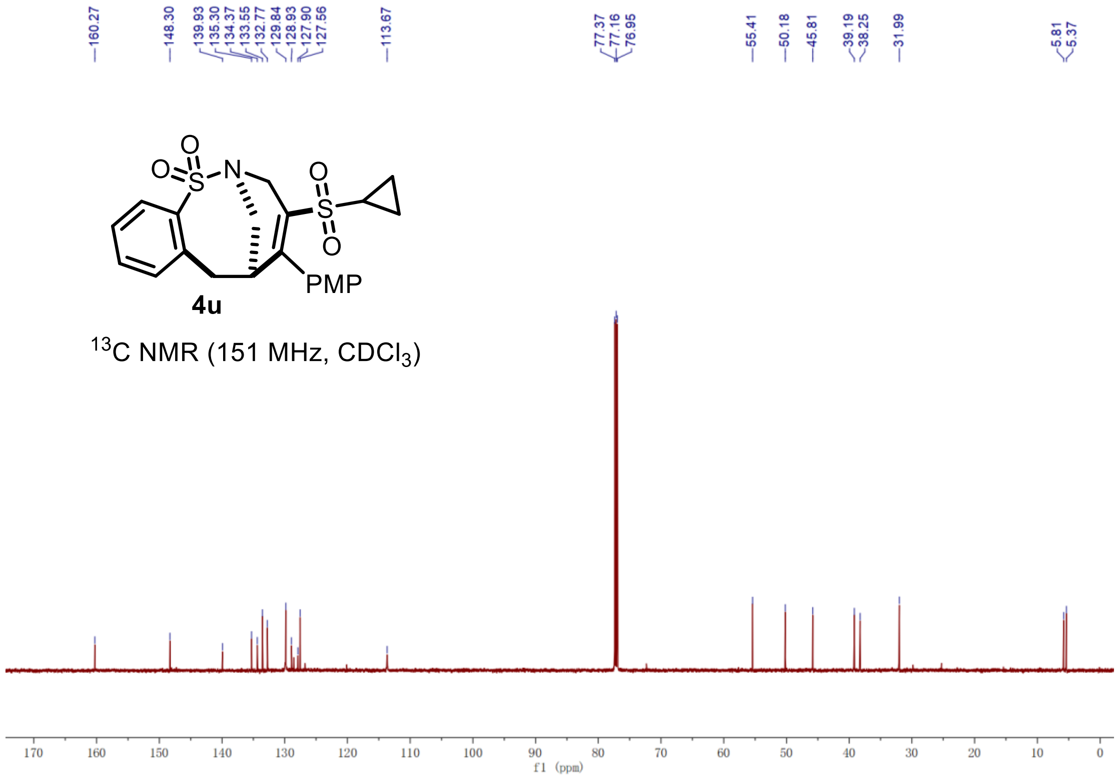

4u
13C NMR (151 MHz, CDCl3)
f1 (ppm)

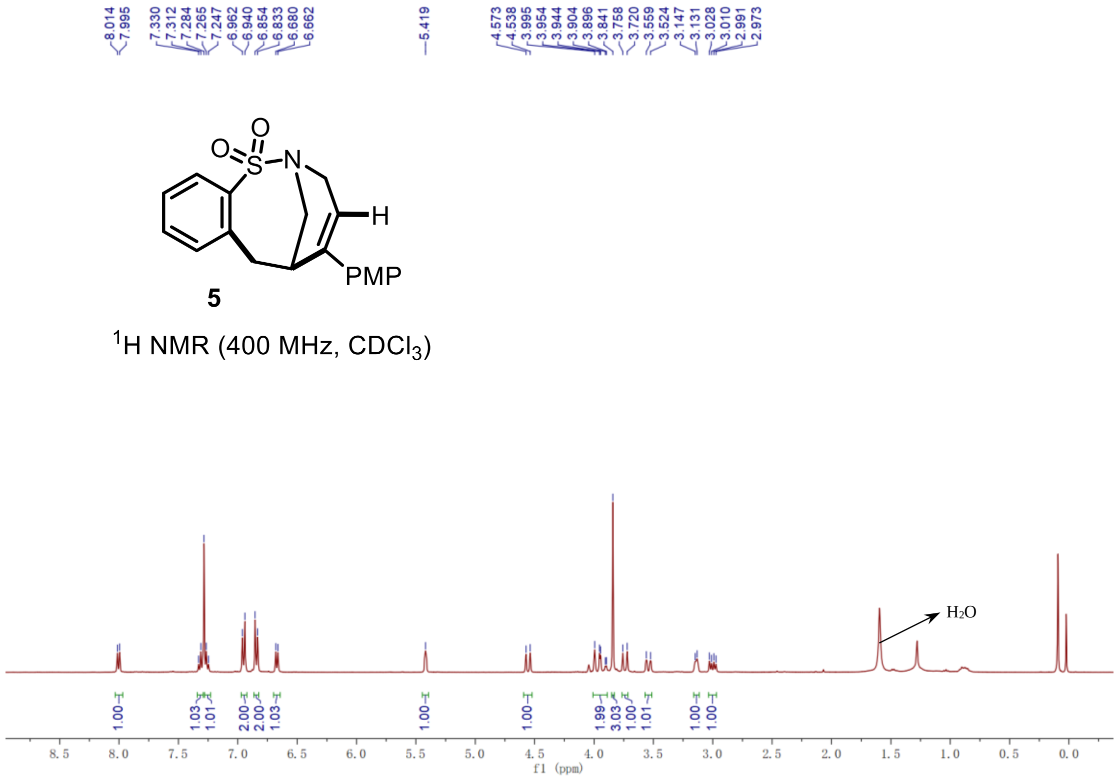

^{1}H NMR (400 MHz, $CDCl_3$)

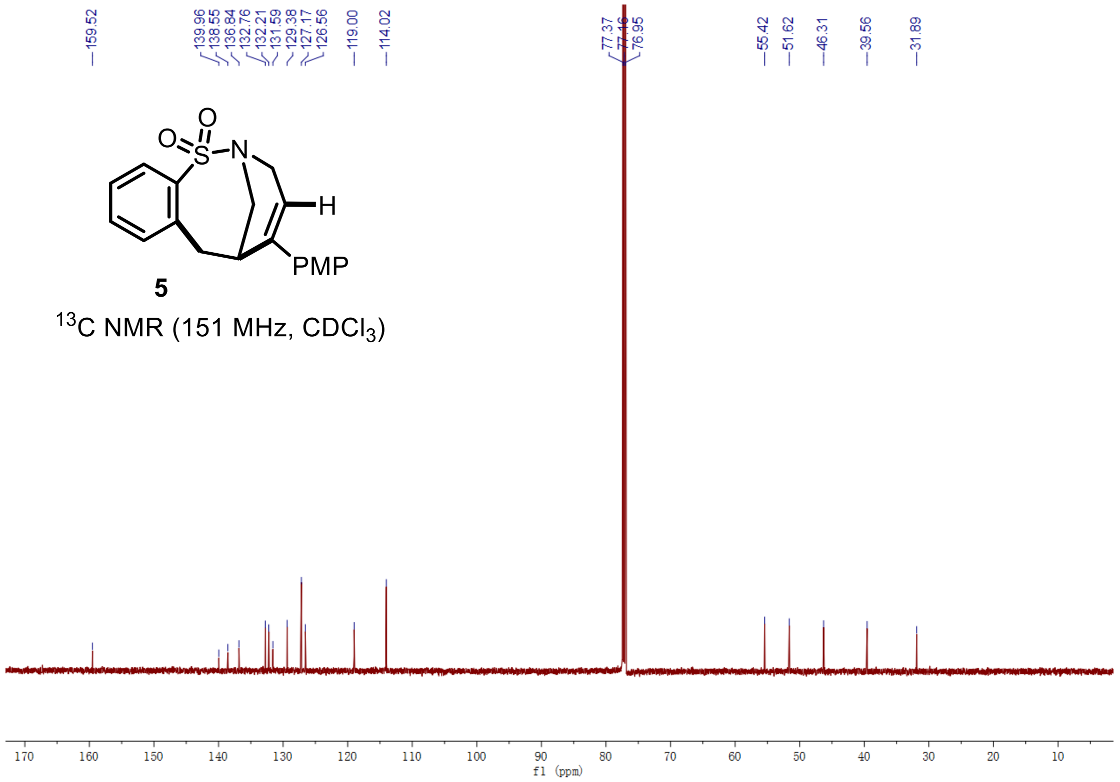

^{13}C NMR (151 MHz, $CDCl_3$)

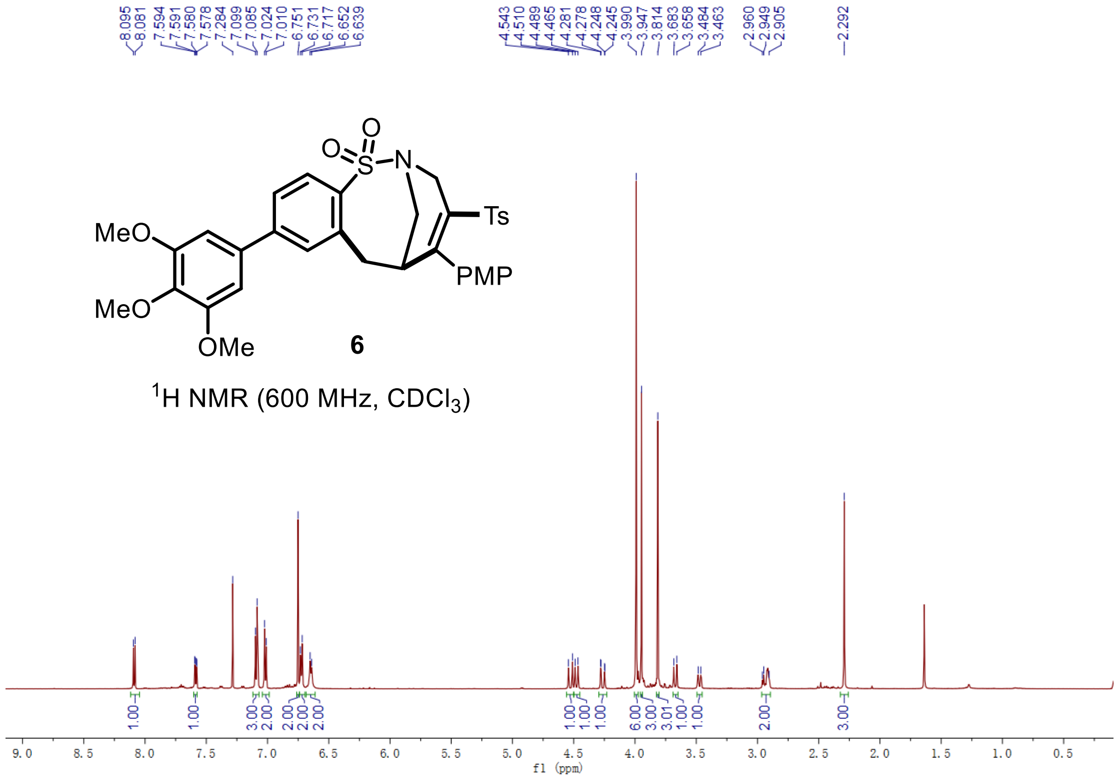
8.095
8.081
7.594
7.591
7.580
7.578
7.284
7.099
7.085
7.024
7.010
6.751
6.731
6.717
6.652
6.639
4.543
4.510
4.489
4.465
4.281
4.278
4.248
4.245
3.990
3.947
3.814
3.683
3.658
3.484
3.463
2.960
2.949
2.905
2.292
Ts
MeO
MeO
OMe
PMP
6
1H NMR (600 MHz, CDCl3)
f1 (ppm)

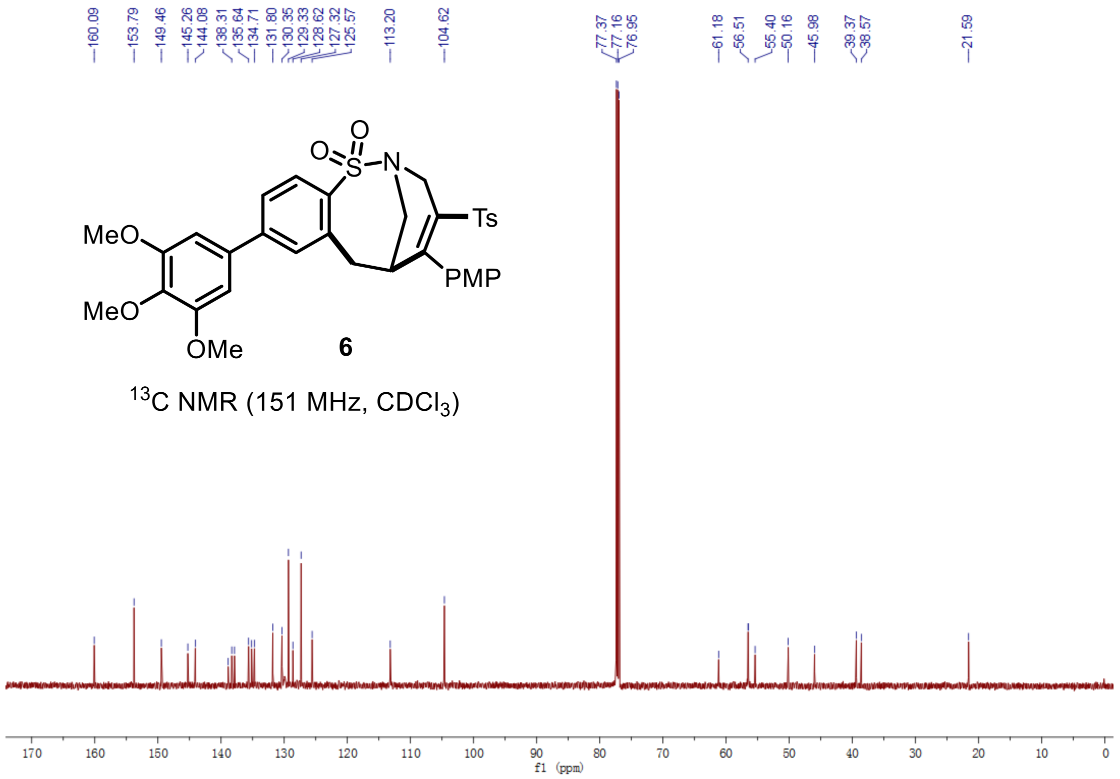
160.09
153.79
149.46
145.26
144.08
138.31
135.64
134.71
131.80
130.35
129.33
128.62
127.32
125.57
113.20
104.62
77.37
77.16
76.95
61.18
56.51
55.40
50.16
45.98
39.37
38.57
21.59
Ts
MeO
MeO
OMe
PMP
6
13C NMR (151 MHz, CDCl3)
f1 (ppm)

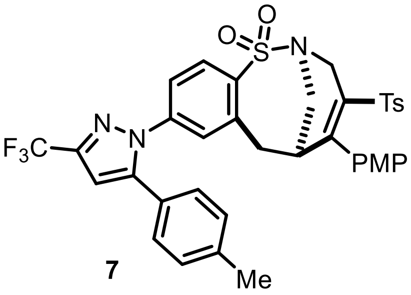

7

^{1}H NMR (400 MHz, $CDCl_3$)

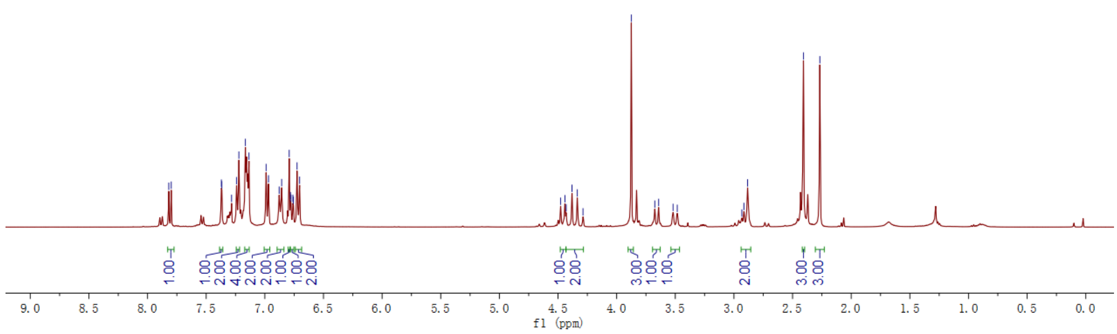

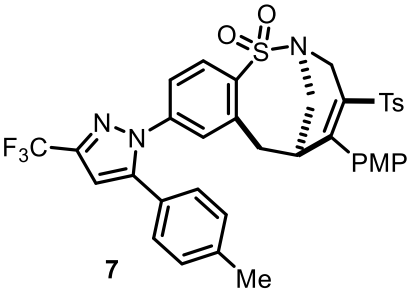

7

^{13}C NMR (101 MHz, $CDCl_3$)

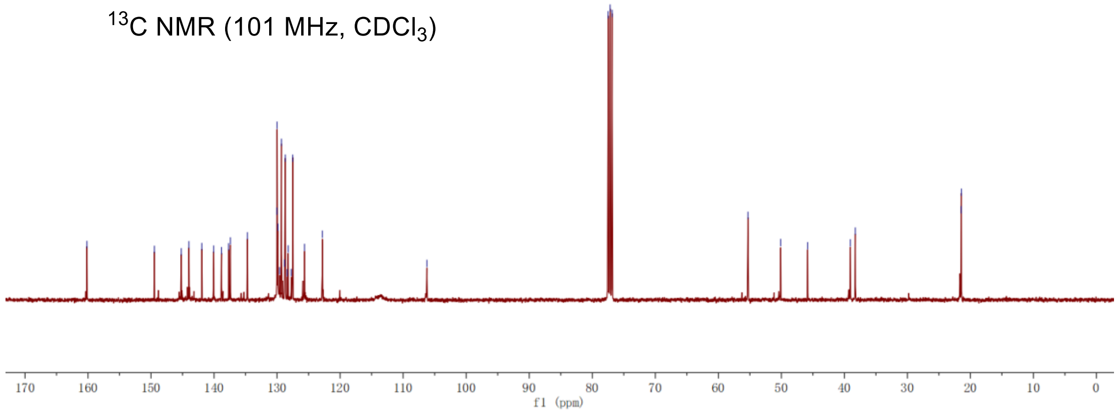

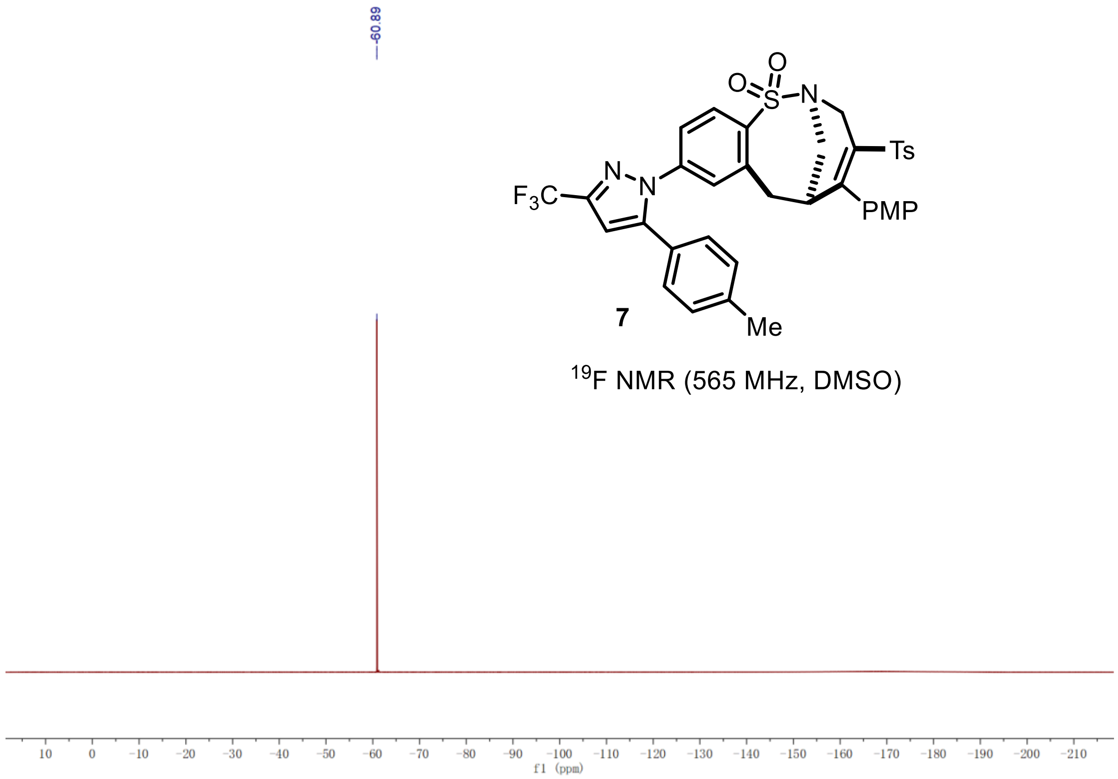
-60.89
F3C
N
N
O
O
S
N
Ts
PMP
Me
7
19F NMR (565 MHz, DMSO)
f1 (ppm)

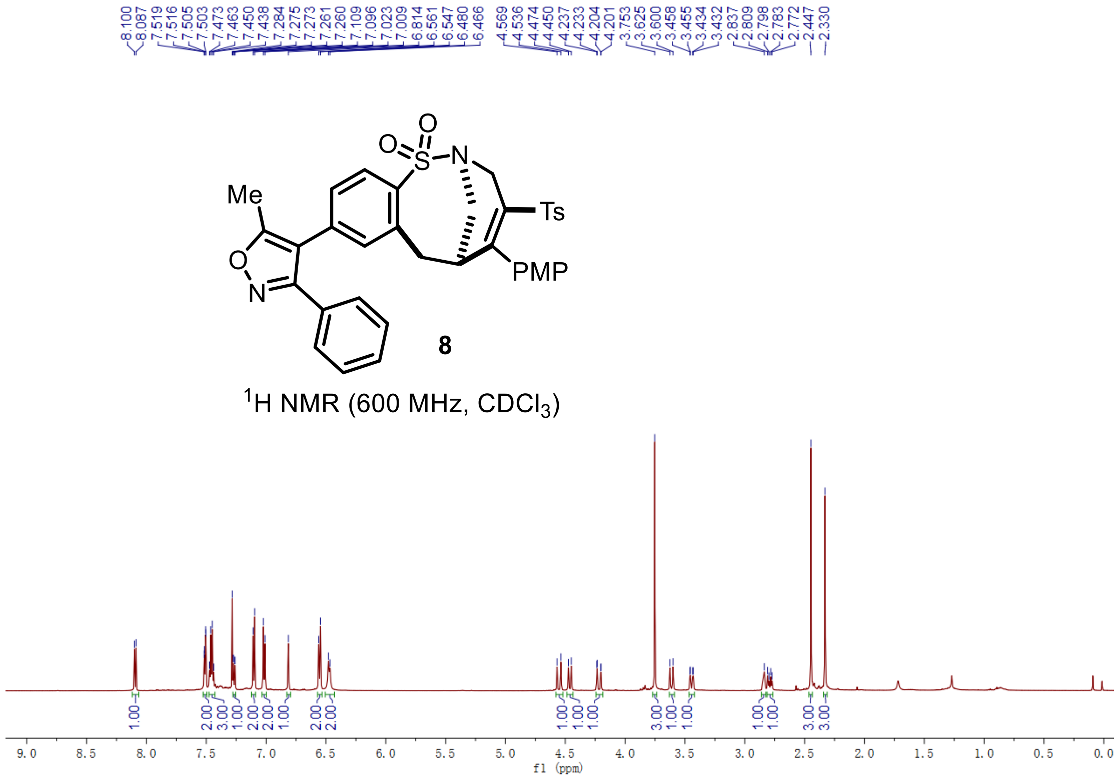
8.100
8.087
7.519
7.516
7.505
7.503
7.473
7.463
7.450
7.438
7.284
7.275
7.273
7.261
7.260
7.109
7.096
7.023
7.009
6.814
6.561
6.547
6.480
6.466
4.569
4.536
4.474
4.450
4.237
4.233
4.204
4.201
3.753
3.625
3.600
3.458
3.455
3.434
3.432
2.837
2.809
2.798
2.783
2.772
2.447
2.330
Me
O
N
O
O
S
N
Ts
PMP
8
1H NMR (600 MHz, CDCl3)
f1 (ppm)

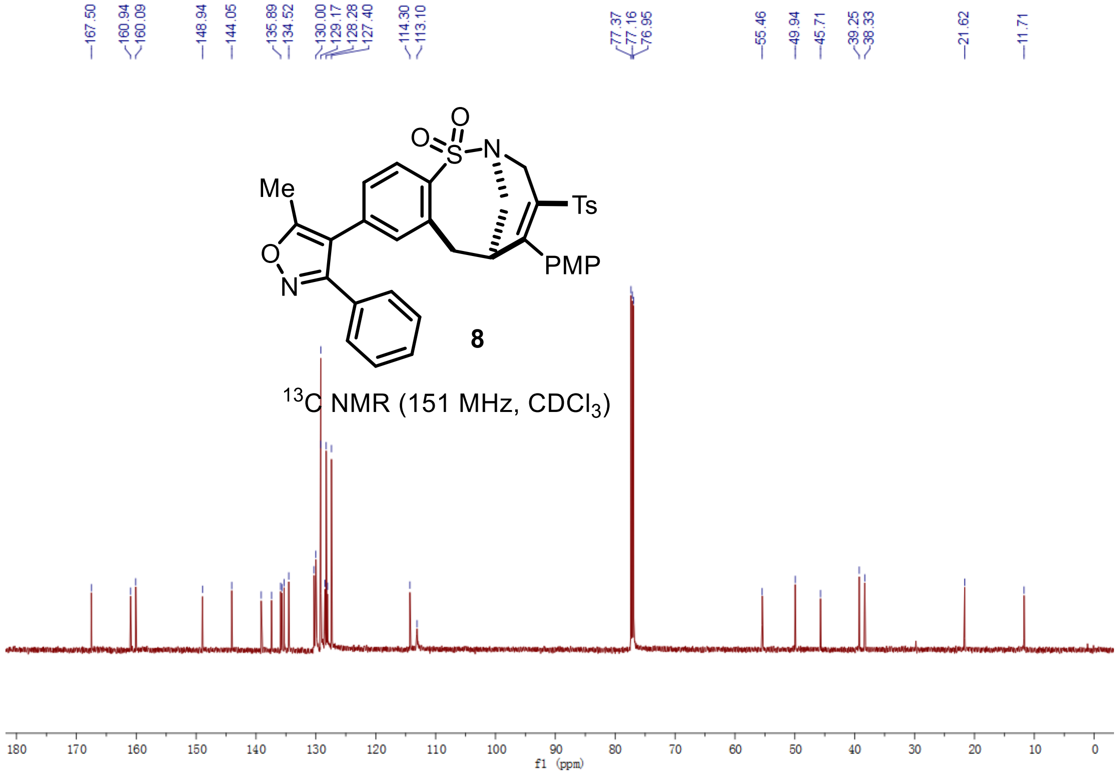
167.50
160.94
160.09
148.94
144.05
135.89
134.52
130.00
129.17
128.28
127.40
114.30
113.10
77.37
77.16
76.95
55.46
49.94
45.71
39.25
38.33
21.62
11.71
Me
O
N
O
O
S
N
Ts
PMP
8
^{13}C NMR (151 MHz, $CDCl_3$)
f1 (ppm)

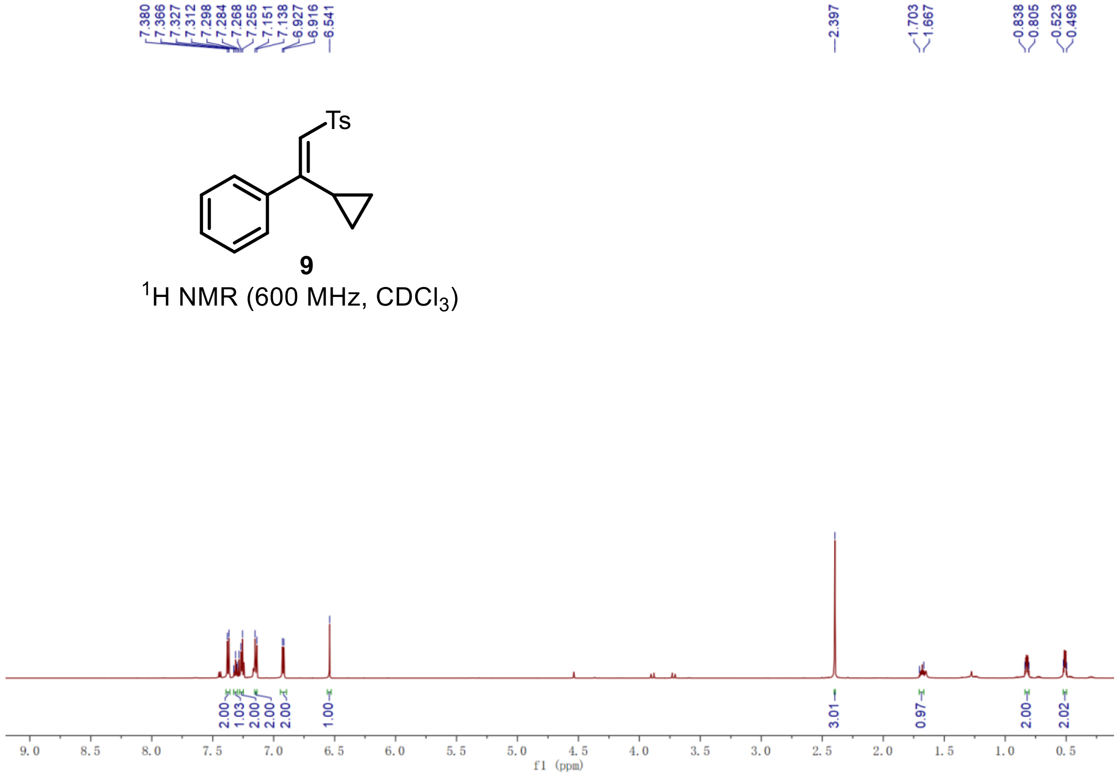
7.380
7.366
7.327
7.312
7.298
7.284
7.268
7.255
7.151
7.138
6.927
6.916
6.541
2.397
1.703
1.667
0.838
0.805
0.523
0.496
Ts
9
^{1}H NMR (600 MHz, $CDCl_3$)
2.00
1.03
2.00
2.00
2.00
1.00
3.01
0.97
2.00
2.02
f1 (ppm)

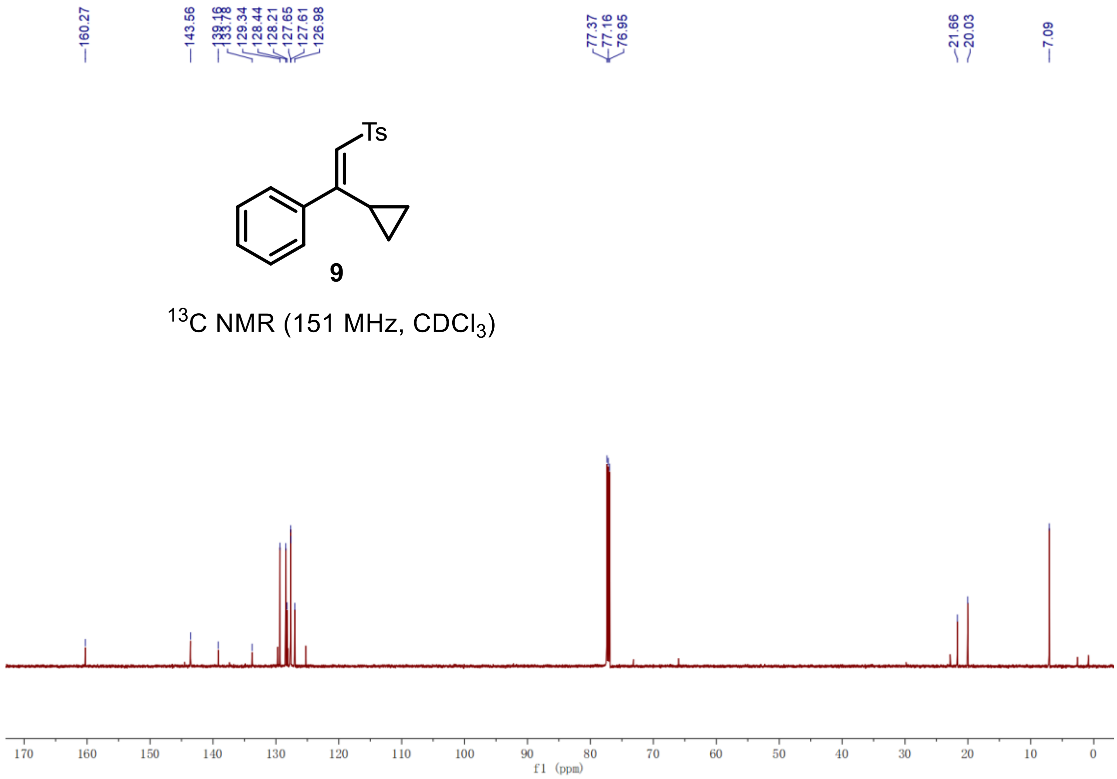

9

^{13}C NMR (151 MHz, $CDCl_3$)
